# Noncanonical IRF3 function mediates STING-dependent pro-inflammatory cytokine production in macrophages

**DOI:** 10.1038/s44319-026-00793-6

**Published:** 2026-05-08

**Authors:** Katherine R Balka, Olivia R Lamb, Rajan Venkatraman, Zoe Magill, Le T Hoang, Ryker Dumbrill, Kate McArthur, Nicole A de Weerd, Paul J Hertzog, Peter J Crack, Linda M Wakim, Kim L Good-Jacobson, Mireille H Lahoud, Meredith O’Keeffe, Dominic De Nardo

**Affiliations:** 1 Department of Biochemistry and Molecular Biology, Immunity Program, Monash Biomedicine Discovery Institute, Monash University, Clayton, VIC Australia; 2 Centre for Innate Immunity and Infectious Diseases, Hudson Institute of Medical Research, Clayton, VIC Australia; 3 Department of Molecular and Translational Science, Monash University, Clayton, VIC Australia; 4 Department of Biochemistry and Pharmacology, The University of Melbourne, Parkville, VIC Australia; 5 Department of Microbiology and Immunology, The University of Melbourne, Peter Doherty Institute for Infection and Immunity, Parkville, VIC Australia; 6 Present Address: Department of Systems Immunology and Proteomics, Institute of Innate Immunity, Medical Faculty, University of Bonn, Bonn, Germany

**Keywords:** Immunology, Microbiology, Virology & Host Pathogen Interaction, Signal Transduction

## Abstract

STING is an important component in the host innate immune system where its activation by cyclic dinucleotides culminates in the production of interferons and pro-inflammatory cytokines that mediate host defence against infection. While the mechanisms that govern STING-induced interferon production have been comprehensively characterised, how pro-inflammatory cytokines are produced downstream of STING remains less understood. Here we discover that IRF3 is critical for effective STING-mediated inflammatory cytokine production from macrophages as those lacking IRF3 display significant defects. Interestingly, the loss of IRF3 does not impact the activation of the prominent pro-inflammatory transcription factor, NF-κB, but rather affects the AP-1 transcriptional complex. We further discover the role of IRF3 in STING inflammatory responses is independent of its phosphorylation and distinct from its role as a transcription factor for induction of type I interferons. This additional activity of IRF3 is dependent on its recruitment to the previously defined IRF3 binding motif within the C-terminal tail of STING. Hence, our findings reveal an unexpected noncanonical function of IRF3 that is critical for mediating STING-induced pro-inflammatory cytokines from macrophages.

## Synopsis

In addition to its canonical role as a transcription factor regulating type I interferons, IRF3 also functions non-canonically downstream of STING to drive pro-inflammatory cytokine production.

- Loss of IRF3 in macrophages leads to a STING-specific defect in pro-inflammatory cytokine production.
- IRF3 drives STING-mediated pro-inflammatory cytokine production independently of global IFN signalling and canonical NF-κB activity.
- IRF3’s role in non-interferon STING responses is independent of its transcriptional activity.
- IRF3 links STING signalling to AP-1 transcription factors to mediate pro-inflammatory cytokine production.

## Abstract (web summary)

In addition to its canonical role as a transcription factor regulating type I interferons, IRF3 also functions non-canonically downstream of STING to drive pro-inflammatory cytokine production.

## Graphical abstract

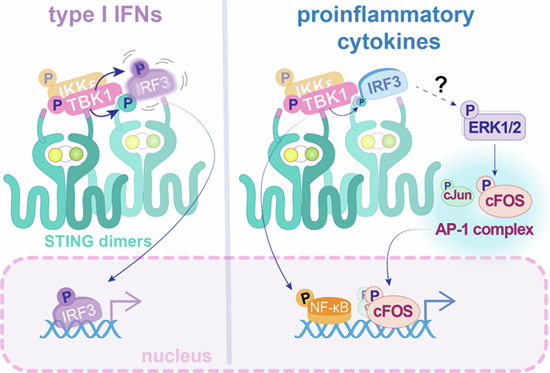

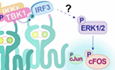

## Introduction

Stimulator of Interferon Genes (STING) is a highly conserved sensor of cyclic dinucleotides (CDNs) originating in bacteria (Morehouse et al, 2020). Mammalian STING binds CDNs produced by bacteria or endogenously upon cyclic GMP-AMP synthase (cGAS) recognition of double-stranded (ds)DNA in the cytosol (Burdette et al, 2011; Sun et al, 2013; Wu et al, 2013). At the steady state, STING dimers remain anchored within endoplasmic reticulum (ER) membranes, however upon activation conformational changes provoke trafficking to the Golgi. Localisation on Golgi membranes mediates the formation of higher order STING oligomers, facilitating the recruitment of effector kinases, TANK-binding kinase 1 (TBK1) and IκBα kinase epsilon (IKKε), and the formation of an active immune signalling platform (Balka and De Nardo, 2021). This culminates in the robust production of type I interferons (IFNα/β) and pro-inflammatory cytokines (e.g. TNF, IL-6) that orchestrate an effective inflammatory response. The transmembrane domains of CDN-bound STING dimers also form an active channel that facilitate proton efflux from the Golgi lumen initiating noncanonical light-chain 3B (LC3B) lipidation and a number of primitive cell defence mechanisms including noncanonical autophagy and lysosomal biogenesis (Gui et al, 2019; Huang et al, 2025; Liu et al, 2023; Lv et al, 2024; Tang et al, 2025; Tapia et al, 2025; Xu et al, 2024; Xun et al, 2024). Interestingly, STING trafficking and these more ancient functions appear to occur independently from TBK1 and IKKε activity. STING protein complexes then bud off the Golgi on endosomal membranes where they continue to signal. Termination of STING signalling is mediated through endosomal sorting complex required for transport (ESCRT)-dependent internalisation of STING vesicles into multivesicular bodies allowing lysosomal fusion and effective degradation (Balka et al, 2023; Gentili et al, 2023; Gonugunta et al, 2017; Kuchitsu et al, 2023).

Immune responses generated through STING are dependent on TBK1 and IKKε (Balka et al, 2020). The recruitment of these key kinases is mediated through a conserved motif within the C-terminal tail of mammalian STING (Venkatraman et al, 2024; Zhang et al, 2019; Zhao et al, 2019). Mutation of a critical lysine residue (L373) to an alanine within this motif renders *Sting*^*L373A*^ cells and mice unable to mount effective immune responses (Yum et al, 2021). Upon binding, active TBK1, and to a lesser extent, IKKε, phosphorylate STING at serine residue 366 (S365 in mouse), leading to the recruitment and activation of Interferon Regulator Factor 3 (IRF3) (Liu et al, 2015; Tanaka and Chen, 2012). Once phosphorylated, IRF3 dimerises and exits the STING complex, translocating to the nucleus to act as a key transcription factor for type I IFN production. In dendritic cells (DCs), IRF3 also likely drives the robust type III IFN (i.e. IFNλ) secretion observed in response to STING activation (Pang et al, 2022). Whilst TBK1 is essential for effective STING-induced IFN responses, both TBK1 and IKKε act redundantly to drive the production of pro-inflammatory cytokines downstream of STING, largely via the activity of nuclear factor kappa B (NF-κB) (Balka et al, 2020). STING activation also triggers the activity of several mitogen-activated protein kinases (MAPKs), which likely contribute to the production of pro-inflammatory cytokines (Abe and Barber, 2014; Venkatraman et al, 2024). Whilst STING-induced IFN responses are well defined, the precise culmination of molecular events that effectively drive pro-inflammatory cytokine production following STING activation remain to be fully determined.

Here, we discover that in addition to its critical role as a potent transcription factor for type I IFN, IRF3 also plays an unexpected but key role in the production of STING-induced pro-inflammatory cytokines. We observed that murine macrophages lacking IRF3, in addition to the expected loss of type I IFNs, also display a significantly blunted ability to transcribe and secrete pro-inflammatory cytokines specifically in response to STING activation. Of note, loss of IRF3 had no impact on the activation of the major cytokine transcription factor, NF-κB. We further report that IRF3 is still able to bind to STING in the absence of TBK1 and robust STING phosphorylation. We determined that recruitment of IRF3 for the induction of pro-inflammatory cytokines is mediated through the described IRF3 binding motif and phosphorylation at serine 365 within murine STING. Finally, we identify that STING may require a unique IRF3–ERK1/2–cFos signalling module in order to produce pro-inflammatory cytokines. Our work identifies an additional and unexpected role for IRF3 within the STING signalling pathway in macrophages.

## Results and discussion

### IRF3 is essential for STING-induced cytokine production in macrophages

**Figure 1:**
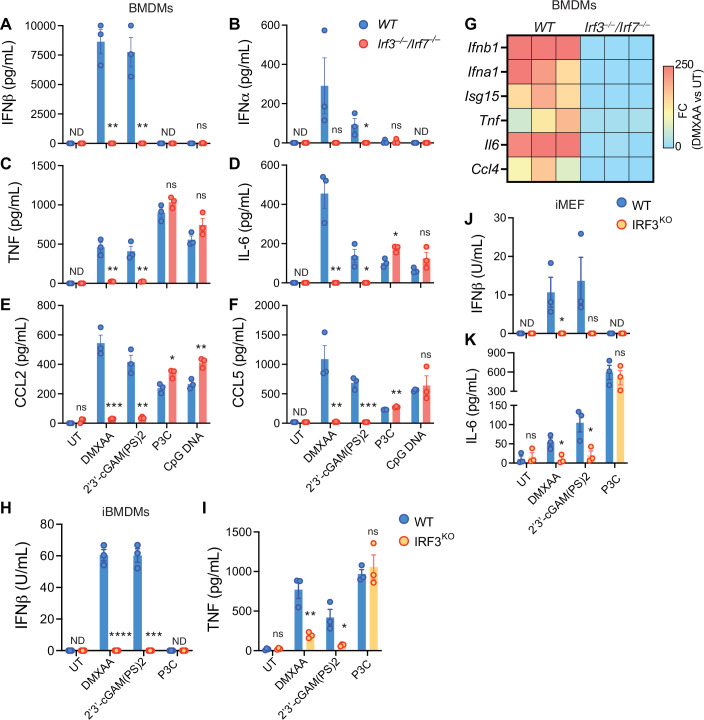
Macrophages lacking IRF3 display reduced inflammatory cytokine production. (**A**–**F**) Wild-type (WT) and *Irf3*^*–/–*^*/Irf7*^*–/–*^ primary BMDMs were left untreated (UT) or stimulated with 50 µg/mL DMXAA, 10 µg/mL 2’3’-cGAM(PS)2, 200 ng/mL Pam3CysK4 (P3C) or 0.5 µM CpG DNA for 4 h before cell supernatants were collected to measure levels of secreted cytokines by LEGENDplex™ assay. Data shown as mean ± SEM from three combined biological experiments. Statistical analysis between genotypes for each condition group was performed using unpaired Student’s *t* test. *P < 0.05 (**B** *P* = 0.045; **D** *P* = 0.0155, *P* = 0.0110; **E** *P* = 0.0166; **F** *P* = 0.002), **P < 0.01 (**A** *P* = 0.0011, *P* = 0.0035; **C** *P* = 0.00167, *P* = 0.00372; **D** *P* = 0.00380; **E** *P* = 0.0013, *P* = 0.0057; **F** *P* = 0.0094, *P* = 0.0023), ***P < 0.001 (**E** *P* = 0.0007; **F** *P* = 0.0004), ns= non-significant (**A** *P* = 0.374; **B** *P* = 0.111, *P* = 0.879: **C** *P* = 0.136, *P* = 0.122; **D** *P* = 0.130; **E** *P* = 0.1169), ND= not determined. (**G**) Wild-type (WT) and *Irf3*^*–/–*^*/Irf7*^*–/–*^ primary BMDMs were left untreated (UT) or stimulated with 50 µg/mL DMXAA for 4 h before cells were lysed for RNA isolation and the expression of type I IFNs, ISN-stimulated genes (ISGs) and pro-inflammatory genes was analysed by qPCR. Data are displayed as a heat map from three biological experiments showing fold change (FC) of DMXAA-treated cells compared to the UT controls. (**H**, **I**) Wild-type (WT) and IRF3^KO^ iBMDMs were left untreated (UT) or stimulated with 50 µg/mL DMXAA, 10 µg/mL 2’3’-cGAM(PS)2 or 200 ng/mL Pam3CysK4 (P3C) for 4 h before cell supernatants were collected and levels of secreted IFNβ and TNF were measured by ELISA. Data shown as mean ± SEM from 3 biological experiments combined. Statistical analysis between genotypes for each condition group was performed using unpaired Student’s *t* test. *P < 0.05 (**I** *P* = 0.0259), **P < 0.01 (**I** *P* = 0.007), ***P < 0.001 (**H** *P* = 0.00015), ****P < 0.0001 (**H** *P* = 0.000075), ns= non-significant (**I** *P* = 0.578, p = 0.61), ND= not determined. (**J**, **K**) Wild-type (WT) and IRF3^KO^ iMEFs were left untreated (UT) or stimulated with 50 µg/mL DMXAA, 10 µg/mL 2’3’-cGAM(PS)2 or 200 ng/mL Pam3CysK4 (P3C) for 4 (**A**) or 18 (**B**) h before cell supernatants were collected and levels of secreted IFNβ or IL-6 were measured by ELISA. Data shown as mean ± SEM from 3 biological experiments combined. Statistical analysis between genotypes for each condition group was performed using unpaired Student’s *t* test. *P < 0.05 (**J** *P* = 0.05; **K** *P* = 0.021, *P* = 0.033), ns= non-significant (**J** *P* = 0.09; **K** *P* = 0.681, *P* = 0.621), ND= not determined. Source data are available online for this figure.

**Figure EV1:**
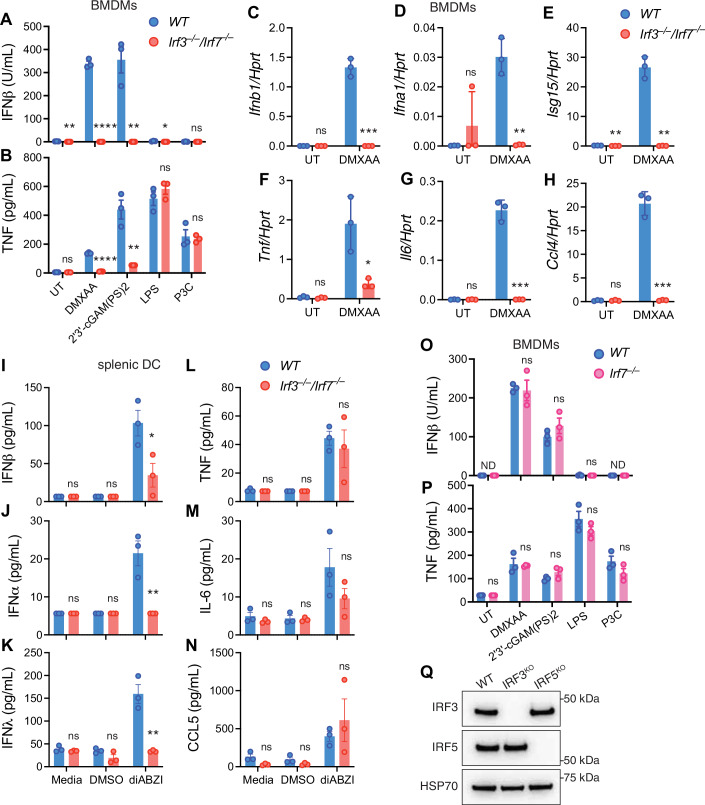
Lack of IRF3, but not IRF7, impacts the ability of macrophages to produce IFNs and inflammatory cytokines. (**A**, **B**) Wild-type (WT) and *Irf3*^*–/–*^*/Irf7*^*–/–*^ primary BMDMs were left untreated (UT) or stimulated with 50 µg/mL DMXAA, 10 µg/mL 2’3’-cGAM(PS)2, 200 ng/mL LPS or 200 ng/mL Pam3CysK4 (P3C) for 4 h before cell supernatants were collected and levels of secreted IFNβ and TNF were measured by ELISA. Data shown as mean ± SEM from three combined biological experiments. Statistical analysis between genotypes for each condition group was performed using unpaired Student’s *t* test. *P < 0.05 (**A** *P* = 0.027), **P < 0.01 (**A** *P* = 0.0025, *P* = 0.0034; **B** *P* = 0.0042), ****P < 0.0001 (**B** *P* = 2.4839E-06). ns= non-significant (**A** *P* = 0.37; **B** *P* = 0.28, *P* = 0.32, *P* = 0.76). (**C**–**H**) Wild-type (WT) and *Irf3*^*–/–*^*/Irf7*^*–/–*^ primary BMDMs were left untreated (UT) or stimulated with 50 µg/mL DMXAA for 4 h before cells for RNA isolation and the expression of IFN and pro-inflammatory genes were analysed by qPCR as indicated. Data shown as mean ± SEM from three combined biological experiments. Statistical analysis between genotypes for each condition group was performed using unpaired Student’s *t* test. *P < 0.05 (**F** *P* = 0.02), **P < 0.01 (**D** *P* = 0.0011), ***P < 0.001 (**C** *P* = 0.0001, **E** *P* = 0.0.0005, *P* = 0.00022; **G** *P* = 0.00011; **H** *P* = 0.00015). ns= non-significant (**C** *P* = 0.66; **D** *P* = 0.37; **F** *P* = 0.3; **G** *P* = 0.86; **H** *P* = 0.92). (**I**–**N**) Primary bulk splenic DCs from wild-type (WT) and *Irf3*^*–/–*^*/Irf7*^*–/–*^ mice were left in fresh media or stimulated with DMSO control or 0.2 µM diABZI for 18 h before cell supernatants were collected to measure levels of secreted cytokines by LEGENDplex™ assay. Data shown as mean ± SEM from three combined biological experiments. Statistical analysis between genotypes for each condition group was performed using unpaired Student’s *t* test. *P < 0.05 (**I** *P* = 0.04), **P < 0.01 (**J** *P* = 0.008; **K** *P* = 0.004). ns= non-significant (**K** *P* = 0.31, *P* = 0.11; **L** *P* = 0.37; **M** *P* = 0.28, *P* = 0.76, *P* = 0.22; **N** *P* = 0.12, *P* = 0.5). (**O**, **P**) Wild-type (WT) and *Irf7*^*–/–*^ primary BMDMs were left untreated (UT) or stimulated with 50 µg/mL DMXAA, 10 µg/mL 2’3’-cGAM(PS)2, 200 ng/mL LPS or 200 ng/mL Pam3CysK4 (P3C) for 4 h before cell supernatants were collected and levels of secreted IFNβ and TNF were measured by ELISA. Data shown as mean ± SEM from three combined biological experiments. Statistical analysis between genotypes for each condition group was performed using unpaired Student’s *t* test. ns= non-significant (**O** *P* = 0.83, *P* = 0.27, *P *= 0.37; **P** *P* = 0.15, *P* = 0.79, *P* = 0.14, *P* = 0.24, *P* = 0.16). ND not determined. (**Q**) Wild-type (WT), IRF3^KO^ and IRF5^KO^ iBMDMs were lysed for immunoblot with the indicated antibodies. Data shown is representative of three biological experiments.

Interestingly, in a report describing a case of human IRF3 deficiency, it was noted that in response to cGAS-STING activation with dsDNA, IRF3-deficient patient peripheral blood mononuclear cells (PBMCs) exhibited partially blunted TNF gene expression in addition to the expected loss of IFN responses (Andersen et al, 2015). However, the mechanisms for this defect were not investigated. We therefore set out to further examine the role of IRF3 in STING-induced production of pro-inflammatory cytokines by challenging primary bone marrow-derived macrophages (BMDMs) from *Irf3*^*–/–*^*/Irf7*^*–/–*^ mice with STING ligands, DMXAA and 2’3’-cGAM(PS)2, comparing responses to macrophages from wild-type mice. As expected, we saw a complete loss of type I IFN production (Figs. 1A,B and  EV1A), however in addition, we also observed significantly reduced secretion of TNF, IL-6, CCL2 and CCL5 inflammatory cytokines from *Irf3*^*–/–*^*/Irf7*^*–/–*^ BMDMs (Figs. 1C–F and  EV1B). This defect appeared to be specific to STING responses as inflammatory cytokine production induced upon activation of multiple TLRs remained intact or, in some cases, even increased (Figs. 1A–F and  EV1A,B). We further observed a significant reduction in the gene expression of type I IFNs (*Ifnb1*, *Ifna1*), IFN-stimulated genes (*Isg15*) and pro-inflammatory cytokines/chemokines (*Tnf*, *Il6*, *Ccl4*) in *Irf3*^*–/–*^*/Irf7*^*–/–*^ BMDMs (Figs. 1G and  EV1C-H). Interestingly, whilst bulk DCs isolated from spleens of *Irf3*^*–/–*^*/Irf7*^*–/–*^ mice produced significantly reduced levels of type I and III IFN compared to WT DCs, there was no difference in their secretion of pro-inflammatory cytokines (Fig. EV1I-N). To determine the potential contribution of IRF7 to this phenotype, we next examined STING-induced IFNβ and TNF levels secreted from *Irf7*^*–/–*^ BMDMs and found no differences compared to WT BMDMs (Fig. EV1O-P), confirming that the defects observed in *Irf3*^*–/–*^*/Irf7*^*–/–*^ macrophages are solely attributable to IRF3 loss. To test the role of IRF3 directly, we generated IRF3-deficient immortalised BMDMs (IRF3^KO^ iBMDMs) using CRISPR/Cas9 gene editing (Fig. EV1Q). The loss of IRF3 alone was sufficient to reduce STING-induced IFNβ (Fig. 1H), as well as TNF cytokine secretion (Fig. 1I). While STING-induced TNF production was significantly reduced in IRF3^KO^ iBMDMs, TNF levels were comparable to those from WT iBMDMs in response to TLR1/2 activation with Pam3Cysk4 (P3C) (Fig. 1I). Similar results were obtained from IRF3-deficient immortalised mouse embryonic fibroblasts (IRF3^KO^ iMEFs) (Fig. 1J,K). Taken together, these data demonstrate that IRF3 plays a key role in mediating pro-inflammatory cytokine production downstream of STING activation.

### IRF3 drives STING-mediated pro-inflammatory cytokine production independently of IFN signalling and canonical NF-κB activity

**Figure 2:**
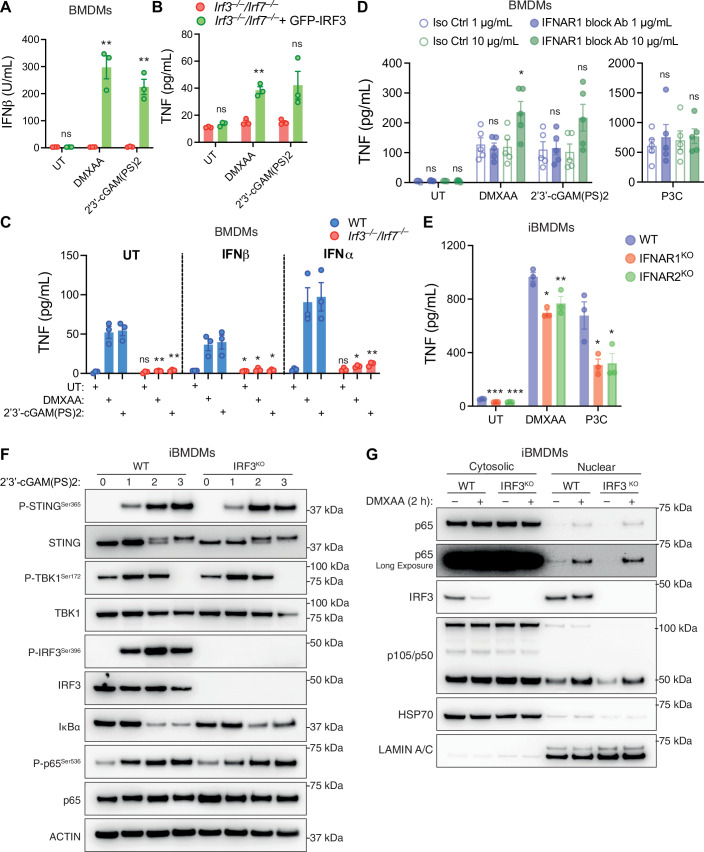
IRF3 drives STING-mediated cytokines independently of type I IFN responses and NF-κB activity. (**A**, **B**) *Irf3*^*–/–*^*/Irf7*^*–/–*^ primary BMDMs and those expressing GFP-IRF3 were left untreated (UT) or stimulated with 50 µg/mL DMXAA or 10 µg/mL 2’3’-cGAM(PS)2 for 4 h before cell supernatants were collected and levels of secreted IFNβ and TNF were measured by ELISA. Data shown as mean ± SEM from 3 combined biological experiments. Statistical analysis between genotypes for each condition group was performed using unpaired Student’s *t* test. **P < 0.01 (**A** *P* = 0.002, *P* = 0.0014; **B** *P* = 0.0013), ns= non-significant (**A** *P* = 0.614; **B** *P* = 0.077, *P* = 0.56). (**C**) Wild-type (WT) and *Irf3*^*–/–*^*/Irf7*^*–/–*^ primary BMDMs were left untreated (UT) or treated with 100 U/mL IFNβ or IFNα for 16 h. Cells were then stimulated with 50 µg/mL DMXAA or 10 µg/mL 2’3’-cGAM(PS)2 for 4 h before cell supernatants were collected and levels of secreted TNF were measured by ELISA. Data shown as mean ± SEM from three combined biological experiments. Statistical analysis between genotypes for each condition group was performed using unpaired Student’s *t* test. *P < 0.05 (*P* = 0.048, *P* = 0.014, *P* = 0.016, *P* = 0.011), **P < 0.01 (*P* = 0.003, *P* = 0.0016, *P* = 0.0092), ns= non-significant (*P* = 0.934, *P* = 0.953). (**D**) Wild-type (WT) primary BMDMs were pre-treated for 1 h with an isotype control antibody or an IFNAR blocking antibody as indicated. BMDMs were then either left untreated (UT) or stimulated with 50 µg/mL DMXAA, 10 µg/mL 2’3’-cGAM(PS)2 or 200 ng/mL Pam3CysK4 (P3C) for 4 h before cell supernatants were collected and levels of secreted TNF were measured by ELISA. Data shown as mean ± SEM from 5 biological experiments combined. Statistical analysis between antibody treatments for each condition group was performed using unpaired Student’s *t* test. *P < 0.05 (*P* = 0.029), ns= non-significant (*P* = 0.26, *P* = 0.66, *P* = 0.88, *P* = 0.56, *P* = 0.86, *P* = 0.06, *P* = 0.75). (**E**) Wild-type (WT), IFNAR1^KO^ or IFNAR2^KO^ iBMDMs were left untreated (UT) or stimulated with 50 µg/mL DMXAA or 100 ng/mL Pam3CysK4 (P3C) for 4 h before cell supernatants were collected and levels of secreted TNF were measured by ELISA. Data shown as mean ± SEM from three biological experiments combined. Statistical analysis between WT iBMDMs and either IFNAR1^KO^ or IFNAR2^KO^ iBMDMs for each condition group was performed using unpaired Student’s *t* test. *P < 0.05 (*P* = 0.011, *P* = 0.03, *P *= 0.0465), **P < 0.01 (*P* = 0.006), ***P < 0.001 (*P* = 0.0007, *P* = 0.0009). (**F**) Wild-type (WT) and IRF3^KO^ iBMDMs were left untreated (0) or stimulated for 1, 2 or 3 h with 10 µg/mL 2’3’-cGAM(PS)2 before cells were lysed for immunoblot with the indicated antibodies. Data shown is representative of four biological experiments. (**G**) Wild-type (WT) and IRF3^KO^ iBMDMs were left untreated (–) or stimulated with 50 µg/mL DMXAA for 2 h (+) before preparation of nuclear and cytoplasmic cell fractions and immunoblotting with the indicated antibodies. Data shown is representative of three biological experiments. Source data are available online for this figure.

**Figure EV2:**
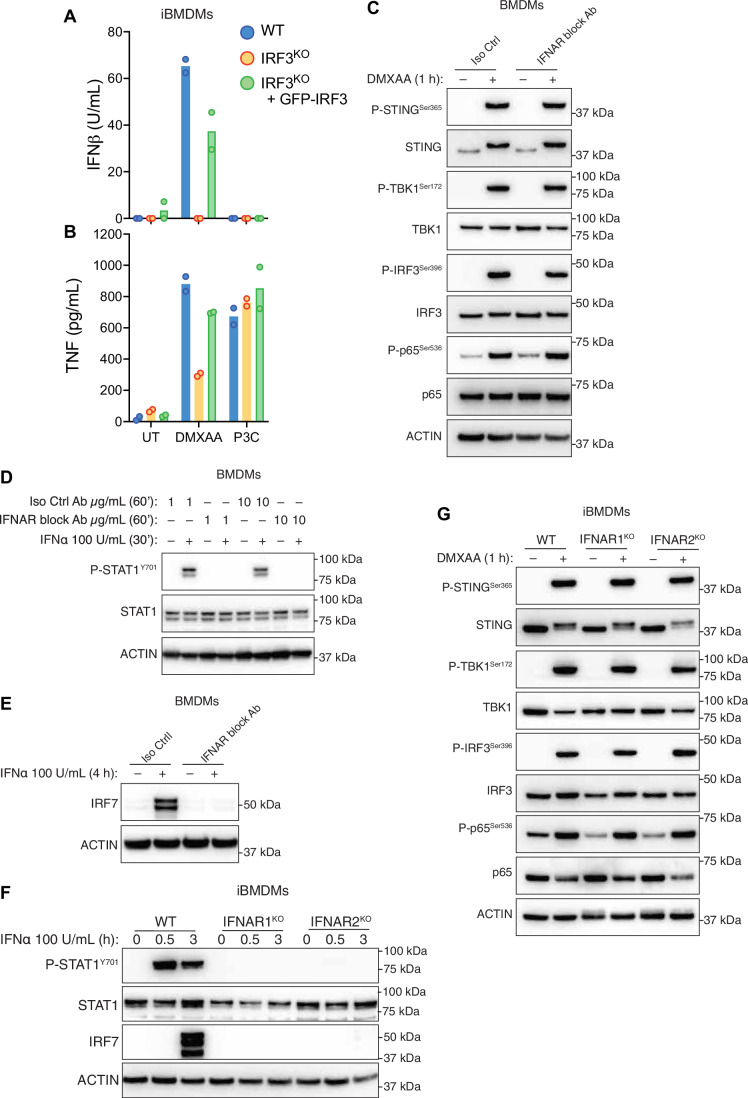
Blocking or deletion of IFNAR abolishes type I IFN responses. (**A**-**B**) Wild-type (WT), IRF3^KO^ and IRF3^KO^ expressing GFP-IRF3 were left untreated (UT) or stimulated with 50 µg/mL DMXAA or 200 ng/mL Pam3CysK4 (P3C) for 4 h before cell supernatants were collected and levels of secreted IFNβ and TNF were measured by ELISA. Data shown as mean from two biological experiments. (**C**) Wild-type (WT) primary BMDMs were pre-treated for 1 h with 10 µg/mL isotype control antibody or IFNAR blocking antibody before being left untreated (–) or stimulated for 1 h with 50 µg/mL DMXAA (+). Cells were then lysed for immunoblot with the indicated antibodies. Data shown is representative of three biological experiments. (**D**) Wild-type (WT) primary BMDMs were pre-treated for 1 h with an isotype control antibody or IFNAR blocking antibody as indicated. Cells were then left untreated (–) or stimulated for 30 min with 100 U/mL IFNα ( + ). Cells were then lysed for immunoblot with the indicated antibodies. Data shown is representative of 3 biological experiments. (**E**) Wild-type (WT) primary BMDMs were pre-treated for 1 h with 10 µg/mL isotype control antibody or IFNAR blocking antibody. Cells were then left untreated (–) or stimulated for 4 h with 100 U/mL IFNα ( + ) before they were lysed for immunoblot with the indicated antibodies. Data shown is representative of three biological experiments. (**F**) Wild-type (WT), IFNAR1^KO^ and IFNAR2^KO^ iBMDMs were left untreated (0) or stimulated for 0.5 or 3 h with 100 U/mL IFNα before cells were lysed for immunoblot with the indicated antibodies. Data shown is representative of three biological experiments. (**G**) Wild-type (WT), IFNAR1^KO^ and IFNAR2^KO^ iBMDMs were left untreated (–) or stimulated for 1 h with 50 µg/mL DMXAA (+). Cells were then lysed for immunoblot with the indicated antibodies. Data shown is representative of three biological experiments.

To determine the mechanisms by which IRF3 regulates STING-induced inflammatory cytokine responses, we utilised lentiviral transduction to reconstitute primary *Irf3*^*–/–*^*/Irf7*^*–/–*^ BMDMs or IRF3^KO^ iBMDMs with a GFP fused version of human IRF3 (GFP-IRF3). When we subsequently activated STING in these cells, we observed a significant rescue of both IFNβ and TNF production compared to *Irf3*^*–/–*^*/Irf7*^*–/–*^ BMDMs (Fig. 2A,B) or IRF3^KO^ iBMDMs alone (Fig. EV2A,B). To rule out a potential indirect defect caused by altered type I IFN signalling in cells lacking IRF3/IRF7, we first pre-treated WT and *Irf3*^*–/–*^*/Irf7*^*–/–*^ BMDMs for 16 h with either IFNβ or IFNα prior to the activation of STING. Neither IFNβ nor IFNα pre-treatment led to a rescue of STING-induced TNF production (Fig. 2C). To examine this further we next treated primary BMDMs with a well characterised interferon-α/β receptor (IFNAR)1 blocking antibody (Sheehan et al, 2006) or an isotype control counterpart prior to activation of STING. In the presence of IFNAR1 blocking we observed no reduction in STING- nor TLR2-induced TNF secretion (Fig. 2D). We further observed no differences in DMXAA-induced STING signalling responses with IFNAR1 blocking antibody treatment (Fig. EV2C). Importantly, we saw that IFNα-induced STAT1 phosphorylation and ISG induction (shown by IRF7 expression) were completely abolished by IFNAR1 blocking antibody treatment, demonstrating efficient inhibition of type I IFN responses under these conditions (Fig. EV2D,E). Finally, we examined the role of IFN signalling genetically, by deleting either IFNAR1 or IFNAR2 in iBMDMs. The efficacy of gene deletion was indicated by an absence of both STAT1 phosphorylation and IRF7 upregulation in response to IFNα stimulation from both IFNAR1^KO^ and IFNAR2^KO^ iBMDMs (Fig. EV2F). While we observed no differences in STING signalling responses between WT, IFNAR1^KO^ and IFNAR2^KO^ iBMDMs (Fig. EV2G), we observed that iBMDMs lacking either IFNAR1 or IFNAR2 displayed small yet significant reductions in STING-induced TNF production (Fig. 2E). Of note, significant reductions were also observed in basal TNF levels and TNF induced by TLR1/2 activation (Fig. 2E). This differs to our findings from both IRF3^KO^ iBMDMs and primary BMDMs activated in the presence of IFNAR1 blocking antibody, where TLR1/2-induced TNF remained unchanged (Figs. 1I and  2D). This strongly suggests that the reductions seen in PRR-driven TNF are likely due to intrinsic differences in cells lacking IFNARs, which are known to have altered macrophage homeostasis and immune responses (Gough et al, 2012).

**Figure EV3:**
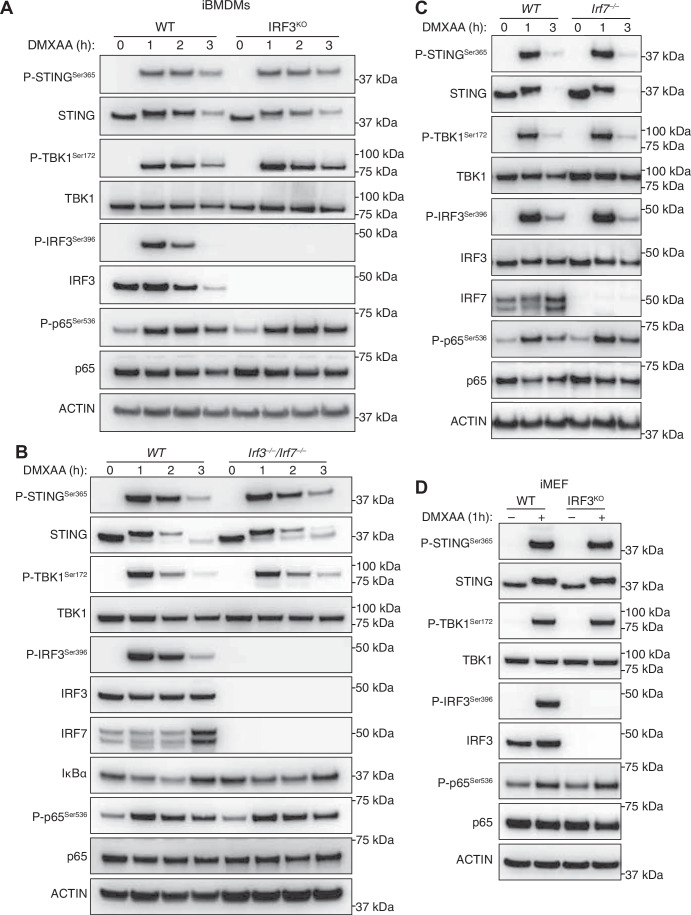
STING signalling is unchanged in the absence of IRF3 and/or IRF7. (**A**) Wild-type (WT) and IRF3^KO^ iBMDMs were left untreated (0) or stimulated for 1, 2 or 3 h with 50 µg/mL DMXAA. Cells were then lysed for immunoblot with the indicated antibodies. Data shown is representative of 3 biological experiments. (**B**) Wild-type (WT) and *Irf3*^*–/–*^*/Irf7*^*–/–*^ primary BMDMs were left untreated (0) or stimulated for 1, 2 or 3 h with 50 µg/mL DMXAA. Cells were then lysed for immunoblot with the indicated antibodies. Data shown is representative of three biological experiments. (**C**) Wild-type (*WT*) and *Irf7*^*–/–*^ primary BMDMs were left untreated (0) or stimulated for 1 and 3 h with 50 µg/mL DMXAA. Cells were then lysed for immunoblot with the indicated antibodies. Data shown is representative of three biological experiments. (**D**) Wild-type (WT) and IRF3^KO^ iMEFs were left untreated (–) or stimulated (+) with 50 µg/mL DMXAA for 1 h. Cells were then lysed for immunoblot with the indicated antibodies. Data shown is representative of three biological experiments.

We next examined key STING signalling responses in the absence of IRF3. Interestingly, in IRF3^KO^ iBMDMs we observed no defects in the phosphorylation of STING, TBK1 nor NF-κB p65, as well as no discernible difference in IκBα degradation – another marker of NF-κB activation (Figs. 2F and  EV3A). Similarly, in *Irf3*^*–/–*^*/Irf7*^*–/–*^, *Irf7*^*–/–*^ BMDMs and IRF3^KO^ iMEFs, we also observed no obvious defects in STING signalling when compared to WT controls (Fig. EV3B–D). Given pro-inflammatory cytokine production is greatly dependent on the transcription activity of NF-κB, we further examined its nuclear translocation in macrophages lacking IRF3. Consistent with comparable phosphorylation of the NF-κB p65 subunit observed in cell lysates from IRF3^KO^ iBMDMs (Figs. 2F and  EV3A), we observed no difference in the amount of p65 that translocated to the nucleus in IRF3^KO^ iBMDMs following STING activation (Fig. 2G). Similarly, the nuclear translocation of the NF-κB p50 subunit (generated from the cleavage of p105 in the cytoplasm) also appeared normal in cells lacking IRF3 (Fig. 2G). Together, these data therefore suggest that the role of IRF3 in STING-induced cytokine production is independent of both global IFN responses and canonical NF-κB signalling.

### STING interacts with IRF3 in the absence of TBK1

**Figure 3:**
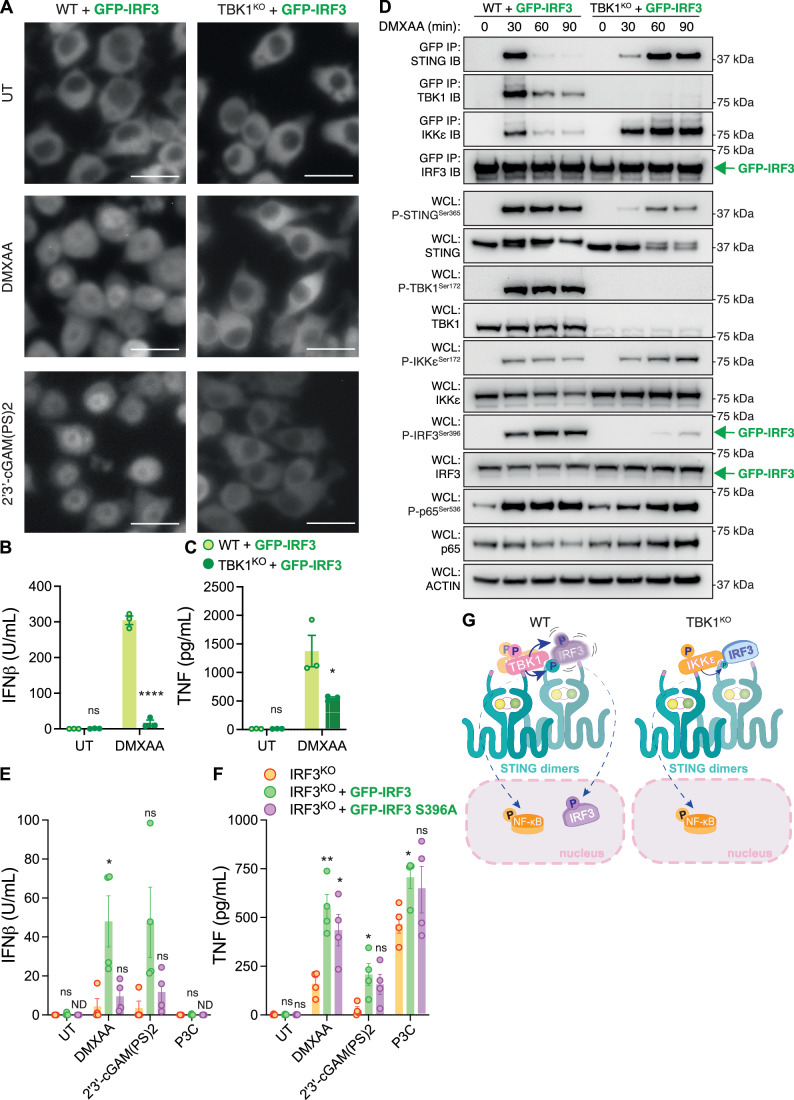
IRF3 interacts with STING in the absence of TBK1. (**A**) Wild-type (WT) iBMDMs expressing GFP-IRF3 and TBK1^KO^ iBMDMs expressing GFP-IRF3 were left untreated (UT) or stimulated with 50 µg/mL DMXAA or 10 µg/mL 2’3’-cGAM(PS)2 for 4 h before IRF3 localisation was examined by fluorescence microscopy. Scale bar = 20 µm. Data shown is representative of 3 biological experiments. (**B**, **C**) Wild-type (WT) iBMDMs expressing GFP-IRF3 and TBK1^KO^ iBMDMs expressing GFP-IRF3 were left untreated (0) or stimulated with 50 µg/mL DMXAA for 4 h before cell supernatants were collected and levels of secreted IFNβ and TNF were measured by ELISA. Data shown as mean ± SEM from three biological experiments combined. Statistical analysis between genotypes for each condition group was performed using unpaired Student’s *t* test. **P < 0.01 (**C** *P* = 0.04), ****P < 0.0001 (**B** *P* = 0.00003), ns= non-significant (**B** *P* = 0.216; **C** *P* = 0.812). (**D**) Wild-type (WT) iBMDMs expressing GFP-IRF3 and TBK1^KO^ iBMDMs expressing GFP-IRF3 were left untreated (0) or stimulated with 50 µg/mL DMXAA for 30, 60 or 90 min. Cells were lysed and a portion of the whole cell lysate (WCL) underwent immunoblot with the indicated antibodies. The remaining lysate underwent GFP immunoprecipitation (IP) before immunoblotting with the indicated antibodies. Data representative of three biological experiments. (**E**, **F**) IRF3^KO^ iBMDMs and IRF3^KO^ iBMDMs expressing GFP-IRF3 or GFP-IRF3 S396A were left untreated (UT) or stimulated with 50 µg/mL DMXAA, 10 µg/mL 2’3’-cGAM(PS)2 or 100 ng/mL Pam3CysK4 (P3C) for 4 h before cell supernatants were collected and levels of secreted IFNβ and TNF were measured by ELISA. Data shown as mean ± SEM from four biological experiments combined. Statistical analysis between IRF3^KO^ and those expressing either IRF3 or IRF3 S396A for each condition group was performed using unpaired Student’s *t* test. *P < 0.05 (**E** *P* = 0.019; **F** *P* = 0.194, *P* = 0.02, *P* = 0.018), **P < 0.001 (**E** *P* = 0.0022), ns= non-significant (**E** *P* = 0.36, *P* = 0.39, *P* = 0.24, *P* = 0.36; **F** *P* = 0.92, *P* = 0.9, *P* = 0.052, *P* = 0.24). ND not determined. (**G**) A proposed model of IRF3 recruitment to phosphorylated STING in the presence (left) or absence (right) of TBK1. In the absence of TBK1, IRF3 is not effectively activated and fails to translocate the nucleus. Source data are available online for this figure.

We have previously found that TBK1 is essential for IRF3 activation and production of type I IFN, whilst IKKε is dispensable for IFNβ and instead can mediate STING-induced NF-κB activation redundantly with TBK1 in BMDMs (Balka et al, 2020). We therefore examined if IRF3 can interact with STING in the absence of TBK1. In cells lacking TBK1, IRF3 is poorly activated, i.e. little to no phosphorylation at S396 (Servant et al, 2003), and upon stimulation of STING, IRF3 does not translocate efficiently to the nucleus to drive IFN production (Abe and Barber, 2014). Consistent with this, we observed no nuclear localisation of GFP-IRF3 when expressed in TBK1^KO^ iBMDMs following STING activation, which contrasted with GFP-IRF3 expressed in WT iBMDMs (Fig. 3A). Furthermore, TBK1^KO^ iBMDMs expressing GFP-IRF3 produced very little IFNβ and reduced TNF in response to STING activation compared to their WT counterparts (Fig. 3B,C). We next examined the temporal interactions of GFP-IRF3 in these cells with STING–TBK1–IKKε complexes by performing GFP immunoprecipitations. In WT BMDMs expressing GFP-IRF3, IRF3 interacted with STING complexes maximally at 30 min before a large reduction (Fig. 3D), presumably as the majority of GFP-IRF3 leaves the STING complex and translocates to the nucleus. Notably, a small amount of IRF3 continues to interact with STING, TBK1 and IKKε from 60 to 90 min in WT iBMDMs (Fig. 3D). In contrast, in TBK1-deficient iBMDMs a more stable interaction between GFP-IRF3 and STING–IKKε was observed over time (Fig. 3D). Of note and consistent with our previously published findings (Balka et al, 2020; Venkatraman et al, 2024), in murine macrophages lacking TBK1, we still observe the phosphorylation of STING at S365, albeit greatly reduced (Fig. 3D), which is mediated via IKKε activity (Balka et al, 2020).

**Figure EV4:**
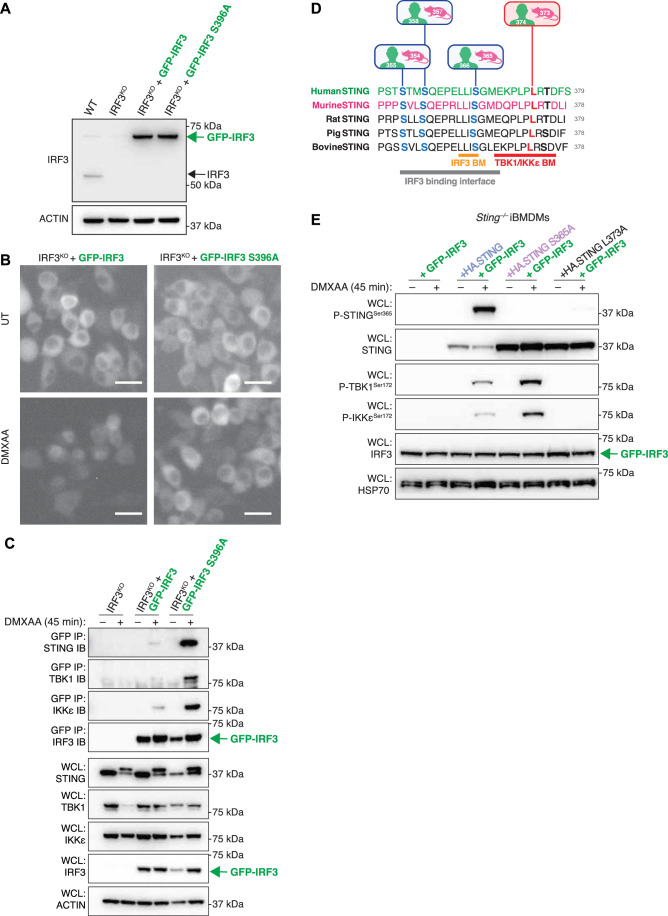
The role of IRF3 in non-IFN STING responses is independent of its canonical function as a transcription factor for IFNs. (**A**) Wild-type (WT) iBMDMs, IRF3^KO^ iBMDMs and those expressing either GFP-IRF3 or GFP-IRF3 S396A were lysed for immunoblot with the indicated antibodies. Data shown is representative of three biological experiments. (**B**) IRF3^KO^ iBMDMs expressing GFP-IRF3 or GFP-IRF3 S396A were left untreated (UT) or stimulated with 50 µg/mL DMXAA for 4 h before IRF3 localisation was examined by fluorescence microscopy. Scale bar = 40 µm. Data shown is representative of three biological experiments. (**C**) IRF3^KO^ iBMDMs expressing GFP-IRF3 or GFP-IRF3 S396A were left untreated (–) or stimulated for 45 min with 50 µg/mL DMXAA (+). Cells were lysed and a portion of the whole cell lysate (WCL) underwent immunoblot with the indicated antibodies. The remaining lysate underwent GFP immunoprecipitation (IP) before immunoblotting with the indicated antibodies. Data representative of 2 biological experiments. (**D**) Multiple sequence alignment of part of the CTT from human (green), murine (pink), rat (black), pig (black) and bovine (black) STING. Conserved phospho-serine (S) residues are highlighted in blue, the conserved lysine (L) residue at position 374 (human STING) is highlighted in red, the threonine (T) residue at position 376 (human STING) previously identified to be phosphorylated is highlighted in black (serine residues in pig and bovine). Red line indicates the TBK1/IKKε binding motif (BM); orange line indicates the IRF3 BM; grey line indicates the IRF3 binding interface. (**E**) *Sting1*^*–/–*^ iBMDMs expressing GFP-IRF3 as well as either HA.STING, HA.STING S365A or HA.STING L373A were left untreated (–) or stimulated for 45 min with 50 µg/mL DMXAA (+). Cells were lysed and a portion of the whole cell lysate (WCL) underwent immunoblot with the indicated antibodies. Data representative of three biological experiments.

Finally, we examined if the phosphorylation of IRF3, and henceforth its dimerization and transcriptional activity, is required for STING-induced pro-inflammatory cytokine production by reconstituting IRF3^KO^ iBMDMs with either GFP-IRF3 or a GFP-IRF3 S396A mutant (Fig. EV4A). As expected, GFP-IRF3 was able to translocate from the cytoplasm to the nucleus, while GFP-IRF3 S396A remained cytoplasmic upon STING activation (Fig. EV4B). When we activated STING in macrophages expressing GFP-IRF3 they were able to induce IFNβ compared to IRF3^KO^ iBMDMs, whilst cells expressing GFP-IRF3 S396A only induced very low levels of IFNβ (Fig. 3E). In contrast, macrophages expressing either GFP-IRF3 or GFP-IRF3 S396A were able to produce significant amounts of TNF (Fig. 3F). Of note, cells expressing GFP-IRF3 S396A were still able to form active STING signalling complexes containing STING, TBK1, IKKε and IRF3, which, similar to our findings from TBK1^KO^ iBMDMs, appeared more stable when compared to interactions observed in cells expressing GFP-IRF3 WT (Fig. EV4C).

Taken together these data demonstrate that, even in the absence of TBK1, IRF3 is recruited to STING to support pro-inflammatory cytokine production, which occurs concurrently to NF-κB activation but independently of IRF3 nuclear translocation and transcriptional activity (Fig. 3G).

### STING cytokine responses are dependent on phosphorylation of serine 365

**Figure 4:**
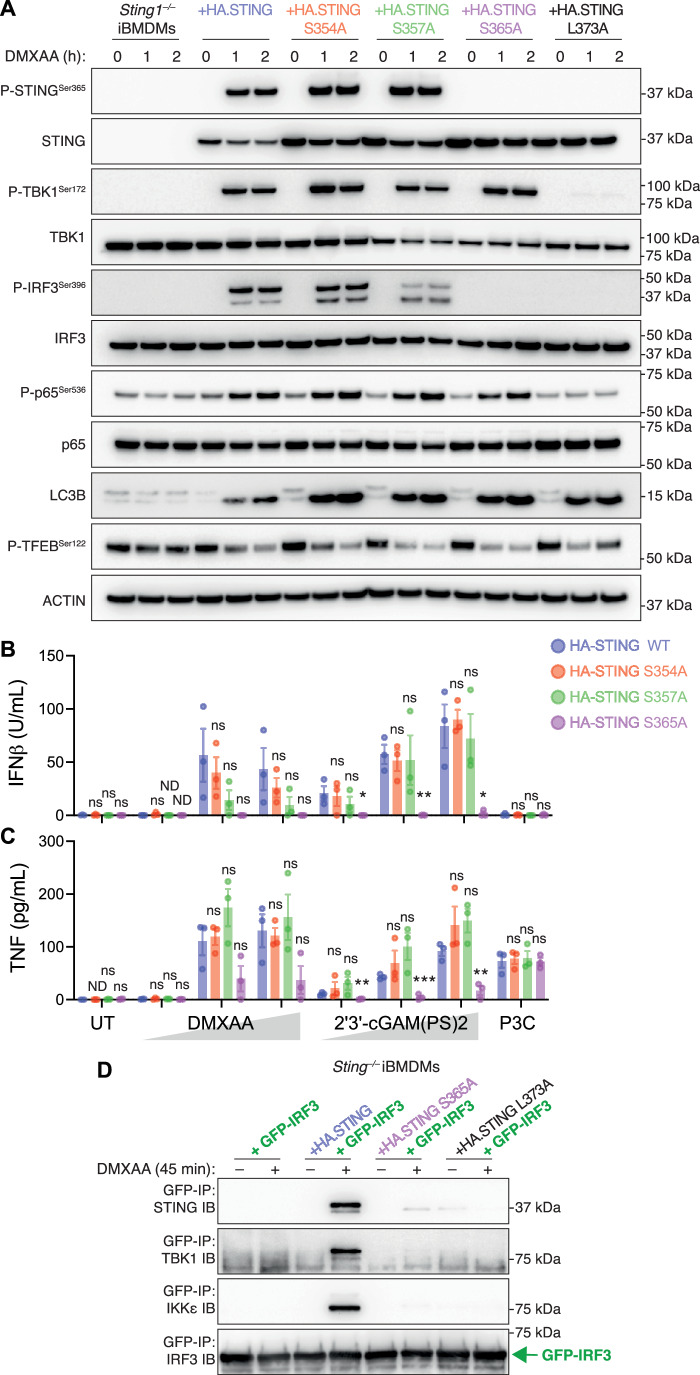
STING S365A macrophages exhibit a defect in TNF production. (**A**) *Sting1*^*–/–*^ iBMDMs and *Sting1*^*–/–*^ iBMDMs expressing HA.STING, HA.STING S354A, HA.STING S357A, HA.STING S365A or HA.STING L373A were left untreated (0) or stimulated for 1 or 2 h with 50 µg/mL DMXAA. Cells were then lysed for immunoblot with the indicated antibodies. Data shown is representative of three biological experiments. (**B**, **C**) *Sting1*^*–/–*^ iBMDMs expressing HA.STING, HA.STING S354A, HA.STING S357A, HA.STING S365A were left untreated (UT) or stimulated with DMXAA [12.5, 25 and 50 µg/mL], 2’3’-cGAM(PS)2 [2.5, 5 and 10 µg/mL], or 200 ng/mL Pam3CysK4 (P3C) for 4 h before cell supernatants were collected and levels of secreted IFNβ and TNF were measured by ELISA. Data shown as mean ± SEM from 3 biological experiments combined. Statistical analysis between HA-STING WT iBMDMs and variant STING iBMDMs for each condition group was performed using unpaired Student’s *t* test. *P < 0.05 (**B** *P* = 0.0369, *P *= 0.016), **P < 0.01 (**B** *P* = 0.0032; **C** *P* = 0.0059, *P* = 0.0044), ***P < 0.001 (**C** *P* = 0.00076), ns= non-significant (**B** *P* = 0.85, *P* = 0.38, *P* = 0.37, *P* = 0.24, *P* = 0.6, *P* = 0.19, *P* = 0.09, *P* = 0.47, *P* = 0.18, *P* = 0.09, *P* = 0.83, *P* = 0.36, *P* = 0.68, *P* = 0.84, *P* = 0.79, *P* = 0.72, *P* = 0.67, *P* = 0.37, *P* = 0.77; **C** *P* = 0.17, *P* = 0.12, *P* = 0.62, *P* = 0.1, *P* = 0.23, *P* = 0.79, *P* = 0.22, *P* = 0.12, *P* = 0.79, *P* = 0.65, *P* = 0.08, *P* = 0.45, *P* = 18, *P* = 0.36, *P* = 0.09, *P* = 0.25, *P* = 0.08, *P* = 0.8, *P* = 0.76, *P* = 0.91). ND not determined. (**D**) *Sting1*^*–/–*^ iBMDMs expressing HA.STING, HA.STING S365A or HA.STING L373A and expressing GFP-IRF3 were left untreated (–) or stimulated for 45 min with 50 µg/mL DMXAA (+). Cells were lysed and a portion of the whole cell lysate (WCL) underwent GFP immunoprecipitation (IP) before immunoblotting with the indicated antibodies. Data representative of three biological experiments. Source data are available online for this figure.

To mediate IFN responses, IRF3 must be recruited to the phosphorylated serine residue at position 365 (S366 in human STING) within the STING C-terminal tail (CTT), which occurs prior to its own phosphorylation and activation by TBK1 (Tanaka and Chen, 2012). Given that we observed IRF3 still associates with STING and IKKε in the absence of TBK1, we next wanted to determine how this occurs. We postulated that this may involve alternative serine phospho-sites in the STING CTT. Indeed, using mass spectrometry approaches, we and others have identified several additional residues to S365 within the STING CTT that become phosphorylated upon activation (Balka et al, 2023; Konno et al, 2013). Of these STING phospho-sites, residues S354 (S355 in human) and S357 (S358 in human) are located within the described IRF3 binding interface (Zhao et al, 2016) and are highly conserved in mammals together with the critical L373 (L374 in human) residue located within the TBK1/IKKε binding motif (Fig. EV4D). To test this, we reconstituted *Sting1*^*–/–*^ iBMDMs with HA-tagged versions of WT, S354 A, S357A, S365A and L373A STING and tested their signalling and cytokine responses following STING activation. As expected, we observed the loss of STING S365 phosphorylation in cells expressing the HA.STING S365A variant, corresponding with a complete lack of IRF3 phosphorylation (Fig. 4A). We also saw a slight but consistent reduction in IRF3 phosphorylation in macrophages expressing HA.STING S357A. Meanwhile, phosphorylation of both TBK1 and NF-κB p65 was unaffected between the WT and phospho-mutants of STING. As expected, in cells expressing STING L373A, phosphorylation of STING, TBK1, IRF3 and NF-κB p65 were all ablated. Of note, STING functions stemming from its proton channel activity remained intact across all STING variants, as observed by comparable lipidation of LC3B and reductions in TFEB phosphorylation upon STING activation (Fig. 4A). We next examined the ability of the STING serine mutants to produce cytokines upon activation, observing a complete loss of IFNβ secretion from HA.STING S365A macrophages, as expected (Fig. 4B). Interestingly, the production of STING-induced TNF was also significantly reduced in cells expressing HA.STING S365A (Fig. 4C). Of note, TNF production in response to TLR1/2 activation was consistent across all STING variants (Fig. 4C).

The signalling and cytokine data we obtained strongly suggested that STING S365 was the critical residue mediating pro-inflammatory cytokine responses via IRF3. We therefore expressed GFP-IRF3 in *Sting*^*–/–*^ iBMDMs, as well as those expressing WT, S365 A or L373A versions of STING. While IRF3, STING, TBK1 and IKKɛ interacted in cells expressing WT STING, no IRF3 interaction was observed in those expressing STING S365A (Fig. 4D). As expected, we did not observe any interaction of IRF3 in *Sting*^*–/–*^ iBMDMs expressing STING L373A (Fig. 4D), nor STING phosphorylation at S365 (Fig. EV4E). Together this strongly suggests that IRF3 recruitment to STING is indeed primarily mediated via the phosphorylation of S365 (S366 in humans), which is subsequently crucial for driving type I IFNs responses as well as mediating the production of pro-inflammatory cytokines.

### STING-induced pro-inflammatory cytokines are independent of IRF5 but require IRF3-dependent AP-1 transcription factors

**Figure 5:**
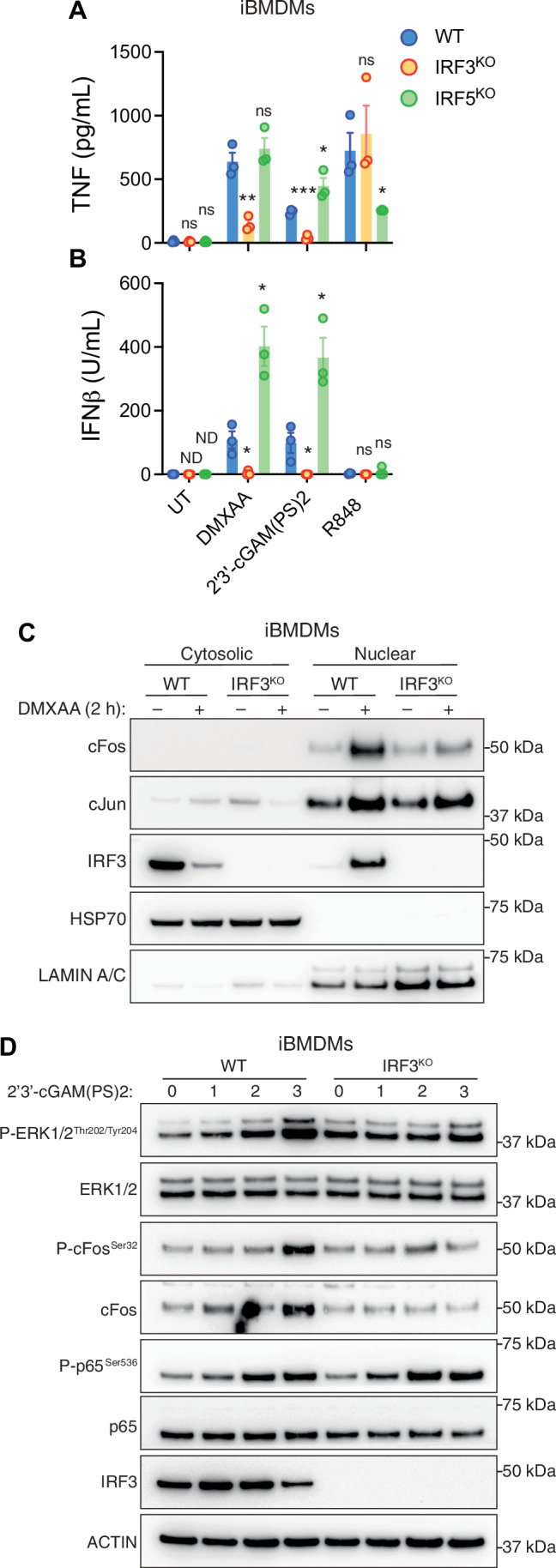
STING-induced pro-inflammatory cytokines are independent of IRF5. (**A**, **B**) Wild-type (WT), IRF3 ^KO^ and IRF5^KO^ iBMDMs were left untreated (UT) or stimulated with 50 µg/mL DMXAA, 10 µg/mL 2’3’-cGAM(PS)2 or 10 ng/mL R848 for 4 h before cell supernatants were collected and levels of secreted TNF and IFNβ were measured by ELISA. Data shown as mean ± SEM from three biological experiments combined. Statistical analysis between WT iBMDMs and IRF3 ^KO^ or IRF5^KO^ iBMDMs for each condition group was performed using unpaired Student’s *t* test. *P < 0.05 (**A** *P* = 0.036, *P* = 0.0296; **B** *P* = 0.019, *P* = 0.012, *P* = 0.037, *P* = 0.0188), **P < 0.01 (**A** *P* = 0.003), ***P < 0.001 (**A** *P* = 0.0003), ns= non-significant (**A** *P* = 0.926, *P* = 0.85, *P* = 0.418, *P* = 0.689; **B** *P* = 0.128, *P* = 0.5), ND= not determined. (**C**) Wild-type (WT) and IRF3^KO^ iBMDMs were left untreated (–) or stimulated with 50 µg/mL DMXAA for 2 h (+) before preparation of nuclear and cytoplasmic cell fractions and immunoblotting with the indicated antibodies. Data shown is representative of three biological experiments. (**D**) Wild-type (WT) and IRF3^KO^ iBMDMs were left untreated (0) or stimulated for 1, 2 or 3 h with 10 µg/mL 2’3’-cGAM(PS)2 before cells were lysed for immunoblot with the indicated antibodies. Data shown is representative of three biological experiments. Source data are available online for this figure.

Our data thus far suggests the role of IRF3 in pro-inflammatory cytokine production downstream of STING is independent of NF-κB activity. IRF5 has previously been shown to cooperate with other transcription factors downstream of nucleic acid-sensing TLRs to drive pro-inflammatory cytokines in monocytes and macrophages (Heinz et al, 2020). The role of IRF5 in STING-mediated cytokine production is currently unknown. We therefore generated IRF5^KO^ iBMDMs (Fig. EV1Q) and examined TNF and IFNβ secretion from WT, IRF3^KO^ and IRF5^KO^ iBMDMs following STING and TLR7 activation (using R848). Consistent with our previous data, IRF3^KO^ iBMDMs displayed a significant reduction in TNF levels (Fig. 5A), as well as a complete loss of IFNβ production (Fig. 5B). In contrast, we observed no reduction in STING-induced TNF nor IFNβ from macrophages lacking IRF5. Importantly, TNF production following TLR7 activation was greatly reduced in IRF5^KO^ iBMDMs, but not those lacking IRF3 further demonstrating IRF5 functional deficiency. This data clearly demonstrates that IRF5 is not involved in cytokine production from macrophages downstream of STING.

**Figure EV5:**
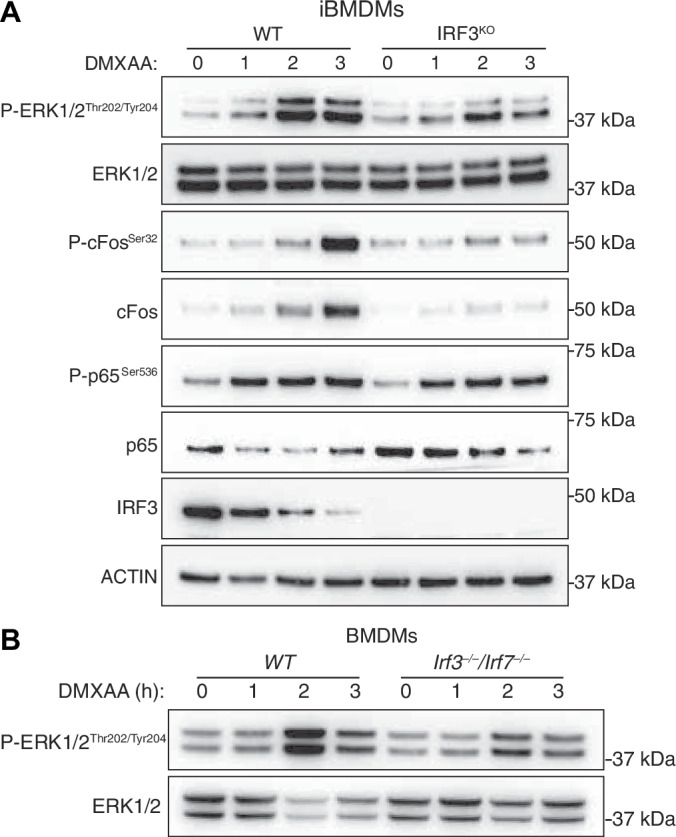
Loss of IRF3 affects ERK1/2 and cFOS activity. (**A**) Wild-type (WT) and IRF3^KO^ iBMDMs were left untreated (0) or stimulated for 1, 2 or 3 h with 50 µg/mL DMXAA before cells were lysed for immunoblot with the indicated antibodies. Data shown is representative of two biological experiments. (**B**) Wild-type (WT) and *Irf3*^*–/–*^*/Irf7*^*–/–*^ primary BMDMs were left untreated (0) or stimulated for 1, 2 and 3 h with 50 µg/mL DMXAA. Cells were then lysed for immunoblot with the indicated antibodies. Data shown is representative of three biological experiments.

We have previously found that STING induces the activation of members of the mitogen-activated protein kinase (MAPK) family (Venkatraman et al, 2024). MAPKs control the activity of components of the activating protein (AP)-1 transcriptional complex (e.g. cFos and cJun) that work in concert with NF-κB to drive pro-inflammatory cytokines. We therefore examined nuclear translocation of cFos and cJun in response to STING activation. Of note, while cJun translocation was comparable between WT and IRF3^KO^ iBMDMs, we observed a clear defect in the ability of cFos to accumulate in the nucleus of IRF3^KO^ iBMDMs following STING activation (Fig. 5C). In the absence of activation cFos is highly unstable, however phosphorylation enhances both its protein stability and nuclear localization (Murphy et al, 2002). Consistent with defective cFos translocation, we also observed reduced STING-mediated cFos phosphorylation in cell lysates from IRF3^KO^ iBMDMs compared to their WT counterparts (Fig. 5D). Similarly, phosphorylation of extracellular signal-regulated kinase (ERK)1/2, which is known to mediate cFos phosphorylation (Gilley et al, 2009), was also greatly reduced in the absence of IRF3 following STING activation, while NF-κB p65 phosphorylation remained intact (Figs. 5D and  EV5). Together these data demonstrate that the reduction in STING pro-inflammatory cytokines observed from IRF3^KO^ iBMDMs may be driven by a defect in the ERK1/2–cFos signalling module.

The importance of cGAS-STING driven immunity is unquestionable and while the molecular mechanisms that induce IFN responses downstream of STING are well defined, how STING drives pro-inflammatory cytokine production remains less clear. Here we describe an unexpected role for IRF3 in mediating STING-dependent pro-inflammatory responses. To our initial surprise, in addition to the expected loss of IFN production, macrophages lacking IRF3 had a significant defect in their ability to produce pro-inflammatory cytokines (e.g. TNF, IL-6). Of note, how IRF3 mediates pro-inflammatory cytokines appeared distinct from STING-induced NF-κB activity, which remained intact in the absence of IRF3. Instead, IRF3 appears to be required for the activity of the AP-1 transcription factor, cFos. We therefore propose a model in which a pool of IRF3 that is recruited to active STING remains unphosphorylated and in a stable complex, facilitating the activation of an Erk1/2–cFos signalling arm that acts in concert with NF-κB to drive maximal production of pro-inflammatory cytokines in macrophages. Of note, during the preparation of our revised manuscript a new research paper was published from the laboratory of Søren Paludan that strongly supports our general findings (Zhang et al, 2025). While some mechanistic details differ between the studies, the major finding that IRF3 plays a central and direct role in STING-mediated pro-inflammatory cytokine responses independently of type I IFN signalling is clearly consistent.

STING is highly conserved throughout evolution and a number of ancestral host defence mechanisms, including noncanonical autophagy and lysosomal biogenesis, remain intact upon activation of mammalian STING. The appearance of the C-terminal tail (CTT) in vertebrates facilitated the ability of STING to induce more ‘modern’ immunity via the production of IFNs and pro-inflammatory cytokines (Margolis et al, 2017). While the CTT of mammalian STING is only ~40 amino acids in length it contains both the IRF3 and TBK1/IKKε binding motifs to elicit downstream immune signalling. Given the close proximity of IRF3 and TBK1/IKKε within a single STING dimer, the active kinase site of TBK1 is too far from the S365/366 residue of STING to enable phosphorylation. Therefore, TBK1 bound to the CTT of one STING dimer is thought to phosphorylate an adjacent STING molecule within a linear oligomeric STING assembly (Zhang et al, 2019). STING clustering at the Golgi and subsequent TBK1 and IRF3 binding is dependent on both palmitoylation and cholesterol accumulation into specific lipid membrane microdomains (Kemmoku et al, 2024). While the predominant role of IRF3 within the mammalian STING pathway is as a transcriptional activator of IFNs, our finding that IRF3 has the additional ability to facilitate a more robust induction of pro-inflammatory cytokines may represent an adaptation in response to the spatial limitations imposed by the CTT. Indeed, in zebrafish, STING has acquired an extended CTT with additional residues containing a putative TRAF6 binding motif that enables exceptionally high NF-κB activity (de Oliveira Mann et al, 2019). Of note, IRF3 has also been implicated in the regulation of cell death through mechanisms independent of its transcriptional activity, further underscoring the existence of IRF3 functions that extend beyond IFN production (Chattopadhyay et al, 2010).

Our findings provide important answers to some previously unexplained observations in the field. Indeed, the defects we observed in STING-induced pro-inflammatory cytokines from macrophages lacking IRF3 are consistent with those seen from IRF3-deficient patient cells (Andersen et al, 2015), as well as, the ablation of TNF seen from *Irf3*^*–/–*^ peritoneal macrophages stimulated with the murine STING agonist, DMXAA (Roberts et al, 2007). However, in both cases how IRF3 may be driving non-IFN cytokine responses was not investigated further. Here we found that in order for IRF3 to mediate pro-inflammatory cytokine production it requires recruitment to the previously described IRF3 binding motif within the CTT and phosphorylation of Serine 365 (S366 in humans) of STING (Liu et al, 2015; Tanaka and Chen, 2012). Several years ago, two seminal studies generated mice expressing the S365A mutant form of STING (*Sting1*^*S365A*^) to selectively ablate STING-induced IFN production while maintaining pro-inflammatory cytokine and other non-IFN responses (Wu et al, 2020; Yamashiro et al, 2020). However, consistent with our findings, in both studies the authors observed that BMDMs from *Sting1*^*S365A*^ mice exhibited significantly reduced secretion of key pro-inflammatory cytokines, including TNF. Thus, together with our data, these findings further support a model in which IRF3 recruitment via S365 is essential for achieving maximal inflammatory cytokine production from macrophages. These observations raise the possibility that IRF3 may also function as a scaffold to facilitate STING-driven pro-inflammatory cytokine production by promoting ERK1/2-cFos activity, independently of canonical NF-κB. However, this model requires further investigation.

Different cell populations are known to induce TLR-mediated inflammatory cytokines via alternative transcription factor usage (Cushing et al, 2014; Kawai et al, 2004). An important finding in this study is that, unlike macrophages, splenic DCs lacking IRF3/7 only show a defect in IFN production, while inflammatory cytokine responses remain intact (Fig. EV1D). These data suggest that some immune cell types can induce STING-mediated pro-inflammatory cytokines in a manner independent of IRF3 binding. Indeed, Yamashiro et al, noted that while *Sting1*^*S365A*^ macrophages displayed a robust defect in STING-induced TNF production, challenged *Sting1*^*S365A*^ mice had only a mild reduction in serum TNF levels (Yamashiro et al, 2020). This may further explain why other studies have not observed a significant role for IRF3 in murine models of STING-associated vasculopathy with onset in infancy (SAVI) nor in autoinflammatory polyarthritis caused by DNase2-deficiency (Li et al, 2022; Warner et al, 2017). In order to understand this further, it would be of interest to examine IRF3 dependence for STING pro-inflammatory cytokine production across a wider array of murine and human cell populations.

In summary, we have identified a noncanonical role of IRF3 for effective macrophage STING pro-inflammatory cytokine production that is distinct from its well-defined transcriptional activity for driving type I and III IFN responses and of STING-induced NF-κB activation. Our findings, together with those of the Paludan group (Zhang et al, 2025), provide additional understanding of the complexity of STING signalling and its associated immune responses and suggests multiple mechanisms exist to drive pro-inflammatory responses following STING activation.

## Methods

**Table:** Reagents and tools table

Reagent/resource	Reference or source	Identifier or catalog number
**Experimental models**		
C57BL/6J mice	Jackson Laboratory	Cat# 000664
*Irf3*^*–/–*^*/Irf7*^*–/–*^ mice	Wakim Laboratory, University of Melbourne, Australia.	N/A
*Irf7*^*–/–*^ mice	Crack Laboratory, University of Melbourne, Australia.	N/A
WT immortalized mouse embryonic fibroblasts (iMEF) expressing Cas9-mCherry	McArthur Laboratory, Monash University, Australia.	White et al, 2014
IRF3^KO^ iMEF cells	McArthur Laboratory, Monash University, Australia.	White et al, 2014
WT immortalized bone marrow-derived macrophages (iBMDMs) expressing Cas9-Blast^R^	This paper	N/A
IRF3^KO^ immortalized bone marrow-derived macrophages (iBMDMs)	This paper	N/A
IFNAR1^KO^ immortalized bone marrow-derived macrophages (iBMDMs)	This paper	N/A
IFNAR2^KO^ immortalized bone marrow-derived macrophages (iBMDMs)	This paper	N/A
TBK1^KO^ immortalized bone marrow-derived macrophages (iBMDMs)	This paper	N/A
IRF5^KO^ immortalized bone marrow-derived macrophages (iBMDMs)	This paper	N/A
WT immortalized bone marrow-derived macrophages (iBMDMs) expressing Cas9-Blast^R^ and GFP-IRF3	This paper	N/A
TBK1^KO^ immortalized bone marrow-derived macrophages (iBMDMs) expressing GFP-IRF3	This paper	N/A
*Sting1*^*–/–*^ immortalized bone marrow-derived macrophages (iBMDMs)	De Nardo Laboratory, Monash University, Australia.	Balka et al, 2020
*Sting1*^*–/–*^ immortalized bone marrow-derived macrophages (iBMDMs) expressing HA.STING	De Nardo Laboratory, Monash University, Australia.	Balka et al, 2023
*Sting1*^*–/–*^ immortalized bone marrow-derived macrophages (iBMDMs) expressing HA.STING L373A	De Nardo Laboratory, Monash University, Australia.	Venkatraman et al, 2024
*Sting1*^*–/–*^ immortalized bone marrow-derived macrophages (iBMDMs) expressing HA.STING S354A	This paper	N/A
*Sting1*^*–/–*^ immortalized bone marrow-derived macrophages (iBMDMs) expressing HA.STING S357A	This paper	N/A
*Sting1*^*–/–*^ immortalized bone marrow-derived macrophages (iBMDMs) expressing HA.STING S365A	This paper	N/A
*Sting1*^*–/–*^ immortalized bone marrow-derived macrophages (iBMDMs) expressing GFP-IRF3	This paper	N/A
*Sting1*^*–/–*^ immortalized bone marrow-derived macrophages (iBMDMs) expressing HA.STING and GFP-IRF3	This paper	N/A
*Sting1*^*–/–*^ immortalized bone marrow-derived macrophages (iBMDMs) expressing HA.STING S365A and GFP-IRF3	This paper	N/A
*Sting1*^*–/–*^ immortalized bone marrow-derived macrophages (iBMDMs) expressing HA.STING L373A and GFP-IRF3	This paper	N/A
**Recombinant DNA**		
pTRIP-GFP-IRF3	Addgene	Plasmid# 127663
pTRIP-GFP-IRF3 S396A	This paper	N/A
pLVX-HA-mSTING	De Nardo Laboratory, Monash University, Australia.	Balka et al, 2023
pLVX-HA-mSTING L373A	De Nardo Laboratory, Monash University, Australia.	Venkatraman et al, 2024
pLVX-HA-mSTING S354A	This paper	N/A
pLVX-HA-mSTING S357A	This paper	N/A
pLVX-HA-mSTING S365A	This paper	N/A
pMD2.G	Addgene	Plasmid #12259
psPAX2	Addgene	Plasmid #12260
pRSV-Rev	Addgene	Plasmid #12253
pMDLg/pRRE	Addgene	Plasmid #12251
pLenti-Cas9-2A-Blast	Addgene	Plasmid #73310
pXPR_BRD003	Broad Institute, USA.	https://portals.broadinstitute.org/gpp/public/vector/details?vector=pXPR_003
pXPR_BRD003-*Irf3* targeting sgRNA	This paper	N/A
pXPR_BRD003-*Ifnar1* targeting sgRNA	This paper	N/A
pXPR_BRD003-*Ifnar2* targeting sgRNA	This paper	N/A
pXPR_BRD003-*Tbk1* targeting sgRNA	This paper	N/A
pXPR_BRD003-*Irf5* targeting sgRNA	This paper	N/A
**Antibodies**		
Rabbit monoclonal anti-STING (D2P2F) antibody	Cell Signaling Technology	Cat# 13647; RRID:AB_2732796
Rabbit monoclonal anti-STING (D1V5L) antibody (Rodent preferred)	Cell Signaling Technology	Cat# 50494, RRID:AB_2799375
Rabbit monoclonal anti-P-STING Ser^365^ (D8F4W) antibody	Cell Signaling Technology	Cat# 72971, RRID:AB_2799831
Rabbit monoclonal anti-NF-κB p65 (C22B4) antibody	Cell Signaling Technology	Cat# 4764; RRID:AB_823578
Rabbit monoclonal anti-NF-κB P-p65 Ser^536^ (93H1) antibody	Cell Signaling Technology	Cat# 3033; RRID:AB_331284
Rabbit polyclonal anti-IκBα antibody	Cell Signaling Technology	Cat# 9242;RRID:AB_331623
Rabbit monoclonal anti-NF-kappaB1 p105/p50 (D4P4D) antibody	Cell Signaling Technology	Cat# 13586RRID:AB_2665516
Rabbit monoclonal anti-P-NF-kappa-B p105 Ser^932^ (18E6) antibody	Cell Signaling Technology	Cat# 4806RRID:AB_2282911
Rabbit polyclonal anti-TBK1 antibody	Cell Signaling Technology	Cat# 3013; RRID:AB_2199749
Rabbit monoclonal anti-P-TBK1 Ser^172^ (D52C2) antibody	Cell Signaling Technology	Cat# 5483; RRID:AB_10693472
Rabbit monoclonal anti-IKKε antibody	Cell Signaling Technology	Cat# 2690; RRID: AB_915926
Rabbit monoclonal anti-P-IKKε Ser^172^ (D1B7) antibody	Cell Signaling Technology	Cat# 8766; RRID:AB_2737061
Rabbit monoclonal anti-IRF3 (D83B9) antibody	Cell Signaling Technology	Cat# 4302; RRID:AB_1904036
Rabbit monoclonal anti-P-IRF3 Ser^396^ (4D4G) antibody	Cell Signaling Technology	Cat# 4947; RRID:AB_823547
Rabbit monoclonal anti-IRF-7 (D8V1J) antibody	Cell Signaling Technology	Cat# 72073 RRID:AB_3073735
Rabbit monoclonal anti-IRF5 (EPR17067) antibody	Abcam	Cat# ab181553 RRID:AB_2801301
Rabbit monoclonal anti-STAT1 (D4Y6Z) antibody	Cell Signaling Technology	Cat# 14995 RRID:AB_2716280
Rabbit monoclonal anti-P-STAT1 Tyr^701^ (D4A7) antibody	Cell Signaling Technology	Cat# 7649 RRID:AB_10950970
Rabbit monoclonal anti-cFos (9F6) antibody	Cell Signaling Technology	Cat# 2250RRID:AB_2247211
Rabbit monoclonal anti-P-cFos Ser^32^ (D82C12) antibody	Cell Signaling Technology	Cat# 5348 RRID:AB_10557109
Rabbit monoclonal anti-cJun (60A8) antibody	Cell Signaling Technology	Cat# 9165 RRID:AB_2130165
Rabbit monoclonal anti-p44/42 MAPK (Erk1/2) (137F5) antibody	Cell Signaling Technology	Cat# 4695; RRID: AB_390779
Rabbit monoclonal anti-P-p44/42 MAPK (Erk1/2) Thr^202^/Tyr^204^ (D13.14.4E) antibody	Cell Signaling Technology	Cat# 4370; RRID: AB_2315112
Rabbit monoclonal LC3B (D11) antibody	Cell Signaling Technology	Cat# 3868; RRID:AB_2137707
Rabbit monoclonal anti-P-TFEB Ser^122^ (E9M5M) antibody	Cell Signaling Technology	Cat# 87932 RRID:AB_3696733
Mouse 1gG2A monoclonal GFP (E36) antibody	Thermo Fisher Scientific	Cat# A-11120; RRID:AB_221568
Mouse monoclonal anti-beta ACTIN HRP (C4) antibody	Santa Cruz Biotechnology	Cat# sc-47778 HRP; RRID:AB_2714189
Mouse monoclonal anti-Lamin A/C (4C11) antibody	Cell Signaling Technology	Cat# 4777 RRID:AB_10545756
Mouse monoclonal anti-Hsp70 (5A5) antibody	Abcam	Cat# ab2787 RRID:AB_303300
Rat anti-mouse IFNβ (IFN-beta, IFNb, IFB, IFF, IFNB1, Fibroblast Interferon, MGC96956) antibody	USBiological Life Sciences	Cat # 138027
Rabbit polyclonal anti-Mouse IFNβ antibody	PBL Assay Science	Cat# 32400-1; RRID:AB_387872
Peroxidase-AffiniPure Goat Anti-Rabbit IgG (H + L) antibody	Jackson ImmunoResearch Labs	Cat# 111-035-003; RRID:AB_2313567
Anti-Mouse IFNAR1(MAR1-5A3) antibody	Leinco Technologies	Cat# I-401; RRID:AB_2491621
Mouse IgG1 Isotype Control (HKSP84) antibody	Leinco Technologies	Cat# I-117 RRID:AB_2830510
**Oligonucleotides and other sequence-based reagents**		
Mouse *Hprt* forward: TGAAGTACTCATTATAGTCAAGGGCA	De Nardo Laboratory, Monash University, Australia.	Balka et al, 2020
Mouse *Hprt* reverse: CTGGTGAAAAGGACCTCTCG	De Nardo Laboratory, Monash University, Australia.	Balka et al, 2020
Mouse *Ifnb1* forward: CCAGCTCCAAGAAAGGACGA	De Nardo Laboratory, Monash University, Australia.	Balka et al, 2020
Mouse *Ifnb1* reverse: TGGATGGCAAAGGCAGTGTA	De Nardo Laboratory, Monash University, Australia.	Balka et al, 2020
Mouse *Ifna1* reverse: CTACTGGCCAACCTGCTCTC	De Nardo Laboratory, Monash University, Australia.	Balka et al, 2020
Mouse *Ifna1* reverse: CCTTCTTGATCTGCTGGGCA	De Nardo Laboratory, Monash University, Australia.	Balka et al, 2020
Mouse *Isg15* forward: TGTGAGAGCAAGCAGCCAGA	De Nardo Laboratory, Monash University, Australia.	Balka et al, 2020
Mouse *Isg15* reverse: CCCCCAGCATCTTCACCTTT	De Nardo Laboratory, Monash University, Australia.	Balka et al, 2020
Mouse *Tnf* forward: CCAAATGGCCTCCCTCTCAT	De Nardo Laboratory, Monash University, Australia.	Balka et al, 2020
Mouse *Tnf* reverse: TGGTGGTTTGCTACGACGTG	De Nardo Laboratory, Monash University, Australia.	Balka et al, 2020
Mouse *Il6* forward: CCAGAAACCGCTATGAAGTTCC	De Nardo Laboratory, Monash University, Australia.	Balka et al, 2020
Mouse *Il6* reverse: CGGACTTGTGAAGTAGGGAAGG	De Nardo Laboratory, Monash University, Australia.	Balka et al, 2020
Mouse *Ccl4* forward: ACCTAACCCCGAGCAACACC	De Nardo Laboratory, Monash University, Australia.	Balka et al, 2020
Mouse *Ccl4* reverse: GAGCCCATTGGTGCTGAGAA	De Nardo Laboratory, Monash University, Australia.	Balka et al, 2020
*Irf3* targeting sgRNA: CACCGGTGGAAGCATGGCCTACGGC	This paper	N/A
*Ifnar1* targeting sgRNA: CACCGTCAGTTACACCATACGAATC	This paper	N/A
*Ifnar2* targeting sgRNA: CACCGGCCATCGTCATAGTGCACAG	This paper	N/A
*Tbk1* targeting sgRNA: ACGGGGCTACCGTTGATCTG	This paper	N/A
*Irf5* targeting sgRNA: CACCGTGAACAGCTGCCAGTACCCA	This paper	N/A
hsIRF3 S396A mutagenesis forward primer: CCTGCACATTgcgAACAGCCACC	This paper	N/A
hsIRF3 S396A mutagenesis reverse primer: TCCACAGTATTCTCCAGG	This paper	N/A
mmSTING S354A mutagenesis forward primer: ACCTCCTCCCgCCGTACTGTC	This paper	N/A
mmSTING S354A mutagenesis reverse primer: GCCACTGAGGTCATGGGG	This paper	N/A
mmSTING S357A mutagenesis forward primer: CTCCGTACTGgCCCAAGAGCC	This paper	N/A
mmSTING S357A mutagenesis reverse primer: GGAGGAGGTGCCACTGAG	This paper	N/A
mmSTING S365A mutagenesis forward primer: ACTCCTCATCgctGGTATGGATCAGCCTC	This paper	N/A
mmSTING S365A mutagenesis reverse primer: CTTGGCTCTTGGGACAGT	This paper	N/A
**Chemicals, enzymes and other reagents**		
Lipofectamine 2000	Thermo Fisher Scientific	Cat# 11668019
Mouse IFN-beta	PBL Assay Science	Cat# 12401-1
Mouse IFN-alpha A	PBL Assay Science	Cat# 12100-1
2’3’-cGAM(PS)2 (Rp/Sp)	InvivoGen	Cat# tlrl-nacga2srs-05
DMXAA (5,6-Dimethylxanthenone-4-acetic acid)	InvivoGen	Cat# tlrl-dmx
diABZI	InvivoGen	Cat# tlrl-diabzi-2
Ultrapure LPS from E. coli 055:B5	InvivoGen	Cat# tlrl-pb5lps
CpG DNA (1826)	InvivoGen	Cat# tlrl-1826
P3C (Pam3CysK4)	InvivoGen	Cat# tlrl-pms
Puromycin	InvivoGen	Cat# ant-pr-1
Blasticidin	InvivoGen	Cat# ant-bl-05
DMSO (Dimethyl sulfoxide)	Sigma-Aldrich	Cat# D2650
*BsmBI*-v2 restriction enzyme	New England Biolabs	Cat# R0739
NEBuffer™ r3.1	New England Biolabs	Cat# B6003
T4 DNA Ligase	New England Biolabs	Cat# M0202
T4 DNA Ligase Reaction Buffer	New England Biolabs	Cat# B0202
GeneJET Plasmid Maxiprep Kit	Thermo Fisher Scientific	Cat# K0492
QIAprep Spin Miniprep Kit	QIAGEN	Cat# 27104
RNeasy Kit	QIAGEN	Cat# 74104
RNase-Free DNase Set	QIAGEN	Cat# 79254
NE-PER Nuclear and Cytoplasmic Extraction kit	Thermo Fisher Scientific	Cat# 78833
**Software**		
Image Lab	Bio-Rad Laboratories	RRID:SCR_014210
Prism	GraphPad Software, LLC.	http://www.graphpad.com/ RRID:SCR_002798
Illustrator	Adobe	http://www.adobe.com/products/illustrator.html RRID:SCR_010279
FIJI	ImageJ	https://fiji.sc RRID:SCR_002285
UniProt	The UniProt Consortium	https://www.uniprot.org/ RRID:SCR_002380
**Other**		
NuPAGE™ 4–12% Bis-Tris Protein Gels, 1.5 mm, 10-well	Thermo Fisher Scientific	Cat# NP0335BOX
NuPAGE™ 4–12% Bis-Tris Midi Protein Gels, 20-well	Thermo Fisher Scientific	Cat# WG1402A
NuPAGE™ MES SDS Running Buffer (20X)	Thermo Fisher Scientific	Cat# NP000202
Immobilon-P PVDF Membrane, 0.45 µm pore size, Hydrophobic PVDF	Millipore (Merck)	Cat# IPVH00005
cOmplete™ Protease Inhibitor Cocktail	Roche Biochemicals (Merck)	Cat# 11836145001
Immobilon Forte Western HRP substrate	Merck Millipore	Cat# WBLUF0500
Dynabeads™ Protein G	Thermo Fisher Scientific	Cat# 10003D
DynaMag™-2 Magnet	Thermo Fisher Scientific	Cat# 12321D
Mouse TNF Uncoated ELISA Kit	Thermo Fisher Scientific	Cat# 88-7324-88
Mouse IL-6 Uncoated ELISA Kit	Thermo Fisher Scientific	Cat# 88-7064-88
Nunc MaxiSorp™ flat-bottom ELISA plates	Thermo Fisher Scientific	Cat# 442404
LEGENDplex™ Mouse Anti-Virus Response Panel kit	BioLegend	Cat# 740622
μ–Slide 8 well	Ibidi	Cat# 80826
Q5^®^ Site-Directed Mutagenesis Kit	New England Biolabs	Cat# E0552S
ChemiDoc™ Imaging System	Bio-Rad	Cat# 12003153
EVOS™ XL Core Imaging System	Thermo Fisher Scientific	Cat# AMEX1000

### Animals

Six- to eight-week-old male or female C57BL/6 wild-type, *Irf3*^*–/–*^*/Irf7*^*–/–*^ or *Irf7*^*–/–*^ mice were euthanized using CO_2_ asphyxiation in accordance with ethics guidelines approved by Monash University (#40728) and University of Melbourne (#26956, #28210) Animal Ethics Committees. All mice were housed under specific pathogen free conditions.

### Generation of primary bone marrow-derived macrophages

To generate primary bone marrow-derived macrophages (BMDMs), mouse femurs and tibias were harvested before bone marrow cells were flushed and cultured in complete Dulbecco’s Modified Eagle Medium (DMEM [made in-house] with 0.1% (w/v) streptomycin, 100 U/mL penicillin and 10% fetal bovine serum [FBS]) supplemented with 20% L929-conditioned media. Cells were incubated for 6 days at 37 °C in a humidified incubator with 10% CO_2_ to generate primary BMDMs.

### Isolation of primary dendritic cells

Isolation of primary dendritic cells (DCs) was performed as previously described (Pang et al, 2022). Mouse spleens were digested using DNase/Collagenase (Roche) at room temperature for 20 min and filtered to create a single cell suspension. Light density cells were isolated by centrifugation in 1.077 g/cm^3^ NycoPrep™ medium (AXIS Shield). Negative selection using a rat monoclonal Ab cocktail against CD3-ε (KT3-1.1), Thy-1 (T24/31.7), Ly6G/Ly6C (1A8), CD19 (ID3) and erythrocytes (TER-119) together with anti-rat immunoglobulin immunomagnetic beads (Qiagen) was used to isolate ‘bulk’ splenic DCs.

### Cell lines

Immortalised (i)BMDMs, iMEFs and Human Embryonic Kidney (HEK) 293 T cells were cultured in complete DMEM at 37°C in 10% CO_2_ humidified atmosphere and passaged every 2–3 days. WT and *Sting1*^*–/–*^ iBMDMs, as well as IRF3^KO^ iMEFs were generated previously (Balka et al, 2020; White et al, 2014). WT iBMDMs expressing Cas9 with blasticidin selection and TBK1, IRF3, IRF5, IFNAR1 and IFNAR2 KO iBMDMs were generated as described below. All cell lines tested negative for mycoplasma contamination prior to use in this study.

### Generation of lentiviral plasmids

The second-generation lentiviral plasmids pLVX-HA.mSTING and pLVX-HA.mSTING L373A were generated previously (Balka et al, 2023; Venkatraman et al, 2024). pLVX-HA.mSTING was used to perform mutagenesis to generate the pLVX-HA.mSTING S354A, pLVX-HA.mSTING S357A and pLVX-HA.mSTING S365A plasmids, while pTRIP-GFP-IRF3 was used to generate pTRIP-GFP-IRF3 S396A. The NEBasechanger online tool was used to design primers for Q5 mutagenesis (New England Biolabs) using the Q5® Site-Directed Mutagenesis Kit (New England Biolabs # E0554S). Third-generation sgRNA plasmids for targeting murine TBK1, IRF3, IRF5, IFNAR1 and IFNAR2 were generated by annealing sgRNA oligos before ligation into the *BsmBI* digested pXPR_BRD003 plasmid. 5-α Competent *E.coli* (high efficiency) (New England Biolabs, C2987U) was used to prepare single colony preparations of plasmids. DNA was extracted and plasmids were verified using Sanger sequencing by Monash Micromon Genomics prior to expansion. All primers were ordered from Integrated DNA Technologies (IDT) and sequences are listed in the Reagents and Tools Table.

### Lentiviral transduction

Third-generation lentivirus was generated by transient transfection of HEK293T cells with 3rd generation lentiviral plasmids (pLenti, pXPR_BRD003), pMDL (packaging), RSV-REV (packaging) and VSVg (envelope) plasmids, while second-generation lentivirus was generated via transient transfection of HEK293T cells with a 2nd generation lentiviral plasmid (pTRIP, pLVX), pPAX2 (packaging) and pMD2.G (envelope) plasmids. In both cases, DNA was complexed into liposomes using Lipofectamine 2000 (Thermo Scientific) diluted in OptimMEM (Thermo Scientific). Lentiviruses were harvested 48 h later, filtered through 0.45 µm filters and used to infect target cell lines. Cells were subsequently enriched for lentiviral plasmid expression via mammalian antibiotic selection (5 µg/ml blasticidin, 2.5 µg/ml puromycin). All plasmids used for lentiviral transduction are listed in the Reagents and Tools Table.

### CRISPR/Cas9 gene editing

We utilized CRISPR/Cas9 gene editing to generate IRF3^KO^, IFNAR1^KO^, IFNAR2^KO^, TBK1^KO^ and IRF5^KO^ iBMDMs. Third generation lentiviral transduction (described above) was used to generate iBMDMs expressing Cas9 (pLenti-Cas9-2 A-Blast), which were subsequently enriched by blasticidin antibiotic selection. CRISPR sgRNA guides targeting murine genes were designed using the Broad Institute CRISPick online tool (https://portals.broadinstitute.org/gppx/crispick/public) and then cloned into a constitutive sgRNA plasmid (pXPR_BRD003), which was subsequently introduced into Cas9-Blast expressing iBMDMs via third generation lentiviral transduction. Cells expressing sgRNA plasmids were then enriched with puromycin. Gene disruption was confirmed by immunoblot and/or functional analysis. The plasmids and sgRNA targeting sequences used are provided in the Reagents and Tools Table.

### Preparation of whole-cell lysates (WCLs)

For immunoblot experiments, ∼5 × 10^5^ iBMDMs, iMEFs or ∼1 × 10^6^ BMDMs were seeded per well in 12-well plates the day prior to stimulation. Following stimulation, the cells were lysed on ice with 120 µL of Radioimmune precipitation assay (RIPA) buffer [20 mM Tris-HCl pH 7.4, 150 mM NaCl, 1 mM EDTA, 1% Triton X-100, 10% glycerol, 0.1% SDS and 0.5% deoxycholate, 5 mM NaF, 10 mM NaPP_i_, 1 mM Na_3_VO_4_] supplemented with 1 mM phenylmethylsulphonyl fluoride (PMSF) and 1× c*O*mplete protease inhibitors (Roche Biochemicals). WCLs were clarified by centrifugation at 17,000×*g* for 1 min through Pierce centrifuge columns (Thermo Scientific) and diluted with 4× reducing LDS sample buffer (Thermo Scientific) supplemented with 5% β-mercaptoethanol (Sigma-Aldrich) and heated to 95 °C for 10 min. Samples were centrifuged at 17,000×*g* for 1 min prior to SDS-polyacrylamide gel electrophoresis (SDS-PAGE) and immunoblotting.

### Immunoprecipitation assay

Immunoprecipitation (IP) experiments were performed similarly as described (De Nardo et al, 2018; Ullah et al, 2023). Approximately 2 × 10^7^ iBMDMs expressing GFP-IRF3 were lysed on ice for 30 min with 1 ml of 1× NP-40 buffer (1% Nonidet-P40, 20 mM Tris-HCl pH 7.4, 150 mM NaCl, 1 mM EGTA, 10% glycerol, 10 mM NaPPi, 5 mM NaF and 1 mM Na_3_VO_4_) supplemented with 1 mM PMSF and c*O*mplete protease inhibitors (Roche Biochemicals). Whole cell lysates were subsequently clarified by centrifugation at 13,000×*g* for 10 min at 4 °C. Following preparation of samples for immunoblot, 1 µg of anti-GFP antibody (Thermo Scientific; clone E36, A-11120) was added to the remaining whole cell lysate. Samples were then incubated at 4 °C for 1 h on a rotator before 50 µL of Dynabeads Protein G (Thermo Scientific; 10004D) were added. Samples were then incubated again at 4 °C for 1 h on a rotator before beads were extensively washed with lysis buffer using a DynaMag-2 magnet (Thermo Scientific; 12321D). Proteins were eluted from beads by the addition of 35 µL of 1× reducing LDS sample buffer/NP-40 buffer and heating at 95 °C for 10 min. Samples were centrifuged at 17,000×*g* for 1 min prior to SDS-polyacrylamide gel electrophoresis (SDS-PAGE) and immunoblotting.

### Preparation of nuclear and cytoplasmic cell fractions

Cellular fractionation was performed using the NE-PER Nuclear and Cytoplasmic Extraction kit (Thermo Scientific) in accordance with the manufacturer’s instructions. Briefly, ~2–2.5 × 10^6^ iBMDMs were harvested in 1.5 mL tubes and washed in ice-cold PBS before centrifugation at 400×*g* for 5 min. Following careful removal of PBS, cell pellets were lysed in 200 µL ice-cold CER I buffer supplemented with c*O*mplete protease inhibitors (Roche Biochemicals). Tubes were then vortexed for 30 s to fully suspend the cell pellet before incubating on ice for 15 min. Next, 11 µL of ice-cold CER II buffer was added to the lysate before centrifugation at ~17,000×*g* for 10 min to pellet the insoluble fraction. The supernatant (cytoplasmic extract) was transferred to a new tube and diluted with 4× reducing LDS sample buffer (Thermo Scientific) supplemented with 5% β-mercaptoethanol (Sigma-Aldrich) and heated to 95 °C for 10 min. The remaining insoluble pellet was then washed in 1 mL PBS to remove any residual cytoplasmic extract before the addition of 100 µL of in ice-cold NER buffer supplemented with c*O*mplete protease inhibitors (Roche Biochemicals). Tubes were then vortexed for 30 s every 10 min for a total of 40 min before centrifugation at ~17,000×*g* for 10 min. The supernatant (nuclear extract) was transferred to a new tube and diluted with 4× reducing LDS sample buffer (Thermo Scientific) supplemented with 5% β-mercaptoethanol (Sigma-Aldrich) and heated to 95 °C for 10 min. Samples were centrifuged at 17,000×*g* for 1 min prior to SDS-polyacrylamide gel electrophoresis (SDS-PAGE) and immunoblotting.

### Immunoblotting

WCL or IP samples were run on NuPAGE™ 4–12% Bis-Tris Protein Gels (Thermo Scientific) with MES running buffer (Thermo Scientific). Following activation of Immobilon-P polyvinyl difluoride (PVDF) membrane (Millipore Merk) in methanol, proteins were transferred to membranes using the Trans-Blot Turbo System (Bio-Rad). Membranes were then blocked using 5% skim milk powder in TBS + 0.1% Tween 20 (TBST) at room temperature (RT) for 1 h before incubation overnight in primary antibodies at 4 °C. Membranes were then washed 3× in TBST and incubated with appropriate HRP-conjugated secondary antibodies for 1 h at RT before membranes were again washed 3× in TBST. Chemiluminescence was detected by subjecting membranes to Immobilon Forte Western HRP substrate (Millipore Merk) before imaging using the ChemiDoc Touch Imaging System (Bio-Rad). Images were acquired and converted to tagged image format file (TIFF) using Image Lab software (Bio-Rad). When required, antibodies were removed from membranes using a mild stripping buffer (50 mM glycine + 0.4% SDS pH 2.2) before 3× washes in TBST and re-probing with primary antibodies. Primary and secondary antibodies used are listed in the Reagents and Tools Table.

### Reverse transcription quantitative polymerase chain reaction (RT–qPCR)

RNA was isolated from 1 × 10^6^ primary BMDMs utilising the RNeasy Plus Mini Kit (QIAGEN). Complementary DNA (cDNA) was then generated using SuperScript III Reverse Transcriptase (Thermo Scientific). qPCR was performed with QuantiNova Probe PCR Master Mix SYBR Green (QIAGEN) using the Bio-Rad CFX384™ Real-Time System Thermal Cycler. Expression levels are displayed as normalised to the murine housekeeping gene *Hprt*. Specific primer sequences used are listed in the Reagents and Tools Table.

### Measurements of secreted cytokines

Levels of secreted murine TNF and IL-6 were measured from cell supernatant by Enzyme-Linked Immunosorbent Assay (ELISA) according to the manufacturer’s protocol (eBioscence, Thermo Scientific), while murine IFNβ were measured using a custom-designed ELISA protocol as previously reported (Roberts et al, 2007). The monoclonal rat anti-mouse IFNβ (USBiological Life Sciences; 138027) was used as a coating antibody, while the polyclonal rabbit anti-mouse IFNβ (PBL Assay Science; 32400-1) was used for detection. Recombinant mouse IFNβ (carrier-free) (PBL Assay Science; 12401-1) was used to generate a standard curve in activity units per mL (U/mL), peroxidase-conjugated AffiniPure F(ab’)2 fragment donkey anti-rabbit IgG (H + L) (Jackson Immuno Research; 711-036-152) was used for chemiluminescence, and PBS with 1% bovine serum albumin (BSA) was used as the assay diluent. A panel of cytokines was measured from cell supernatants using a flow cytometric bead-based LEGENDplex™ assay kit (Mouse Anti-Virus Response Panel; 740622) according to manufacturer’s instructions (BioLegend). Of note, the LEGENDplex™ assay kit measures IFNβ in protein amount (pg/mL).

### IRF3 translocation assays

Approximately 0.5–1 × 10^5^ WT or TBK1^KO^ iBMDMs expressing GFP-IRF3 were seeded in 8-well μ-slide ibiTreat chamber slides (iBidi). The following day, cells were stimulated as described in the figure legends before imaging for localisation of GFP (EVOS Light Cube, GFP: Ex: 470/22 nm; Em: 510/42 nm) using the 20x objective of an EVOS™ XL Core Imaging System with integrated EVOS™ software (Thremo Scientific). Images were then exported as TIFF files.

### Multiple sequence alignment of STING

Protein sequences for orthologs from human (*Homo sapiens*), murine (*Mus musculus*), rat (*Rattus norvegicus*), pig (*Sus scrofa*) and bovine (*Bos taurus*) STING were obtained via UniProt (UniProt, 2025) and aligned using the ‘align’ function (https://www.uniprot.org/align).

### Statistical analysis

Statistical analyses were performed with Prism (GraphPad Software) or Excel (Microsoft) and data are typically presented as the mean ± SEM, where a *P* value < 0.05 was considered significant as determined by an unpaired two-tailed Student *t* test and indicated within the specific figure legends. Significance is indicated by asterisks, defined as *P < 0.05, **P < 0.01, ***P < 0.001 and ****P < 0.0001 with the actual *P* values listed in corresponding figure legends.

## Supplementary information

Peer Review File

Source data Fig. 1

Source data Fig. 2

Source data Fig. 3

Source data Fig. 4

Source data Fig. 5

Expanded View Figures

## Data Availability

No primary datasets have been generated and deposited. The source data of this paper are collected in the following database record: biostudies:S-SCDT-10_1038-S44319-026-00793-6.
