# Supplementary figures and images for: Noncanonical IRF3 function mediates STING-dependent pro-inflammatory cytokine production in macrophages
Source: EMBO Rep. 2026 May 8;27(11):2865–92. doi: 10.1038/s44319-026-00793-6 (PMC13260819; doi:10.1038/s44319-026-00793-6)

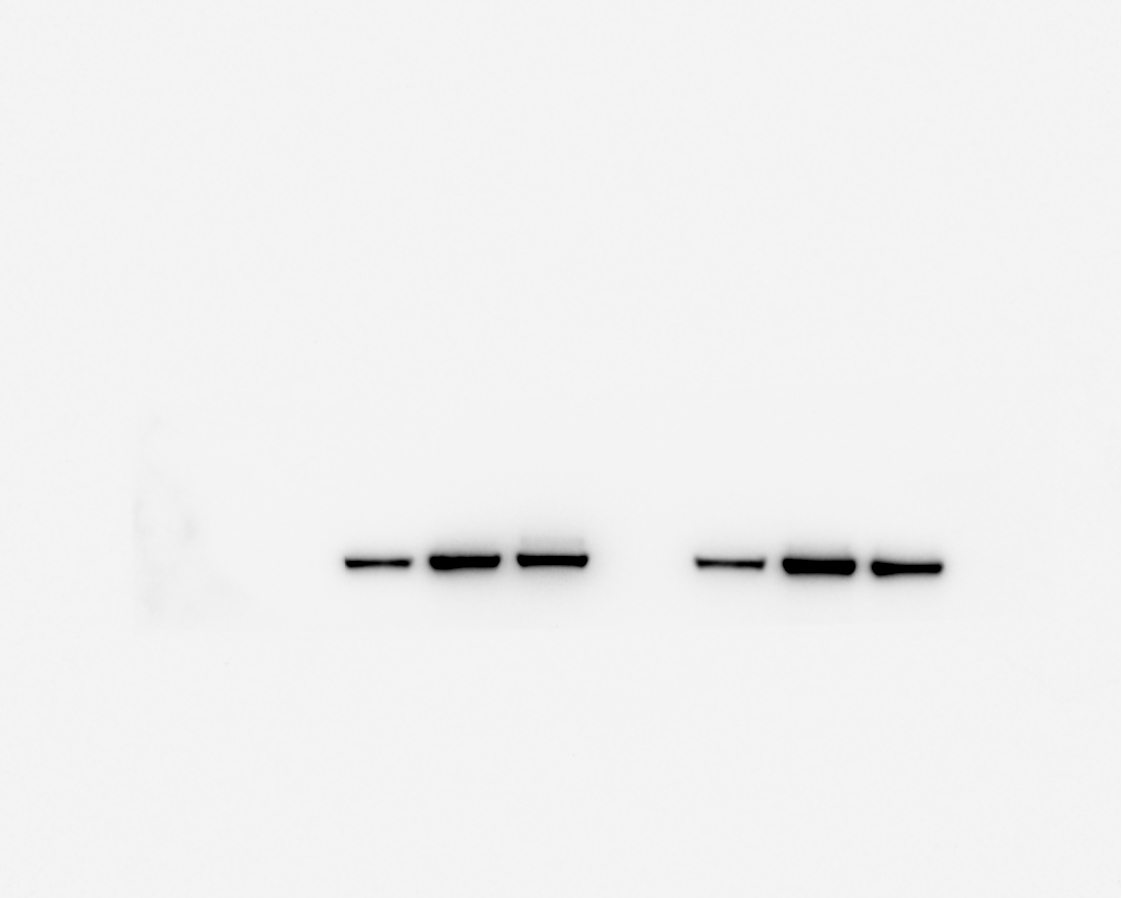

Supplement: Supplementary file 3 — Source data Fig. 2 [file 44319_2026_793_MOESM3_ESM.zip › Figure 2/Fig2F/P-TBK1.tif]

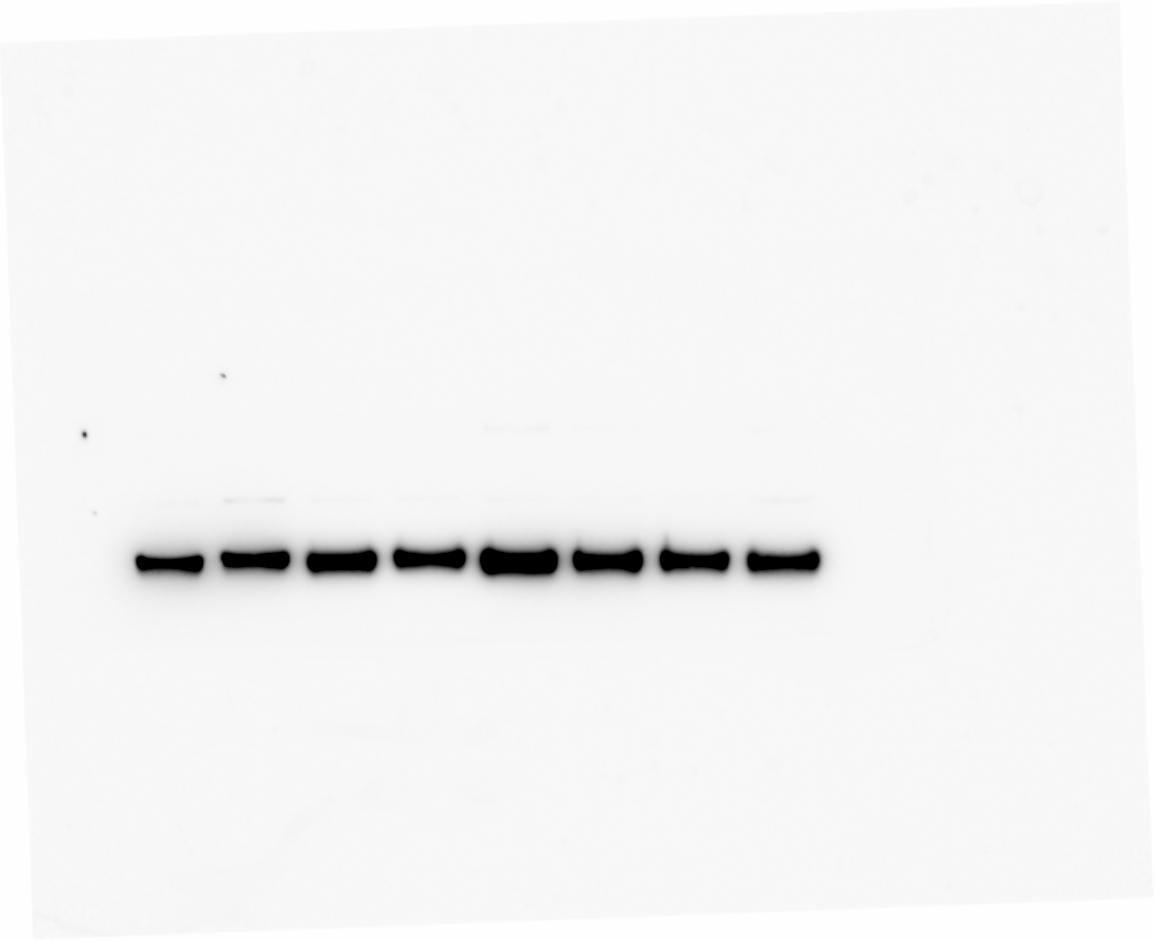

Supplement: Supplementary file 3 — Source data Fig. 2 [file 44319_2026_793_MOESM3_ESM.zip › Figure 2/Fig2F/p65.tif]

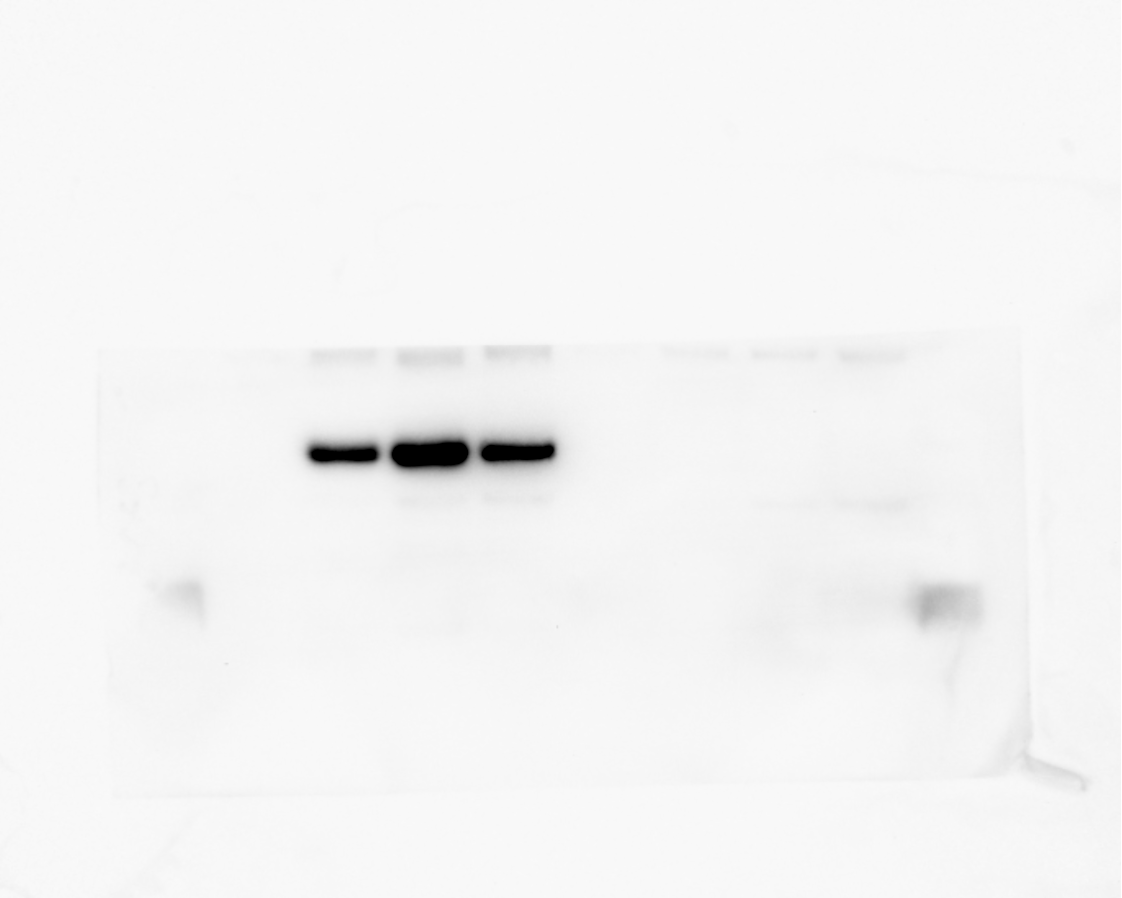

Supplement: Supplementary file 3 — Source data Fig. 2 [file 44319_2026_793_MOESM3_ESM.zip › Figure 2/Fig2F/P-IRF3.tif]

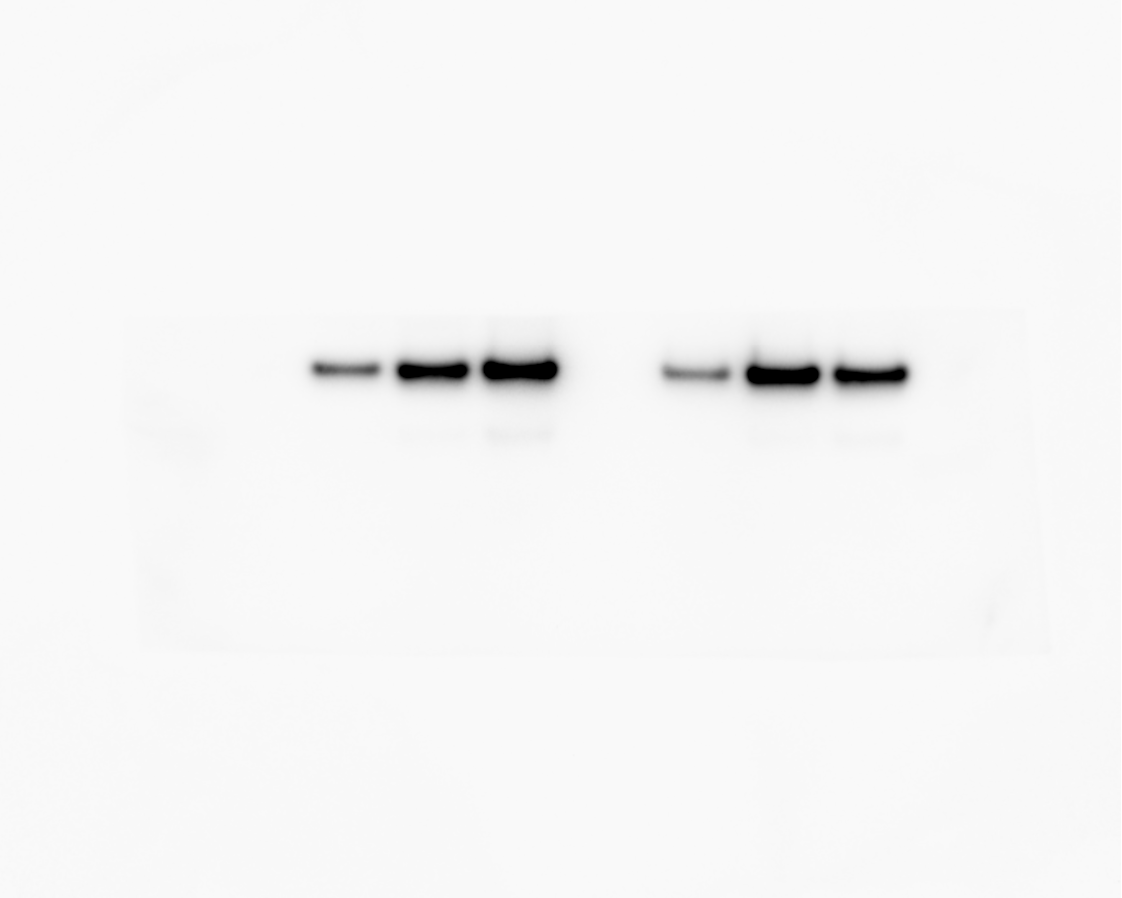

Supplement: Supplementary file 3 — Source data Fig. 2 [file 44319_2026_793_MOESM3_ESM.zip › Figure 2/Fig2F/P-STING.tif]

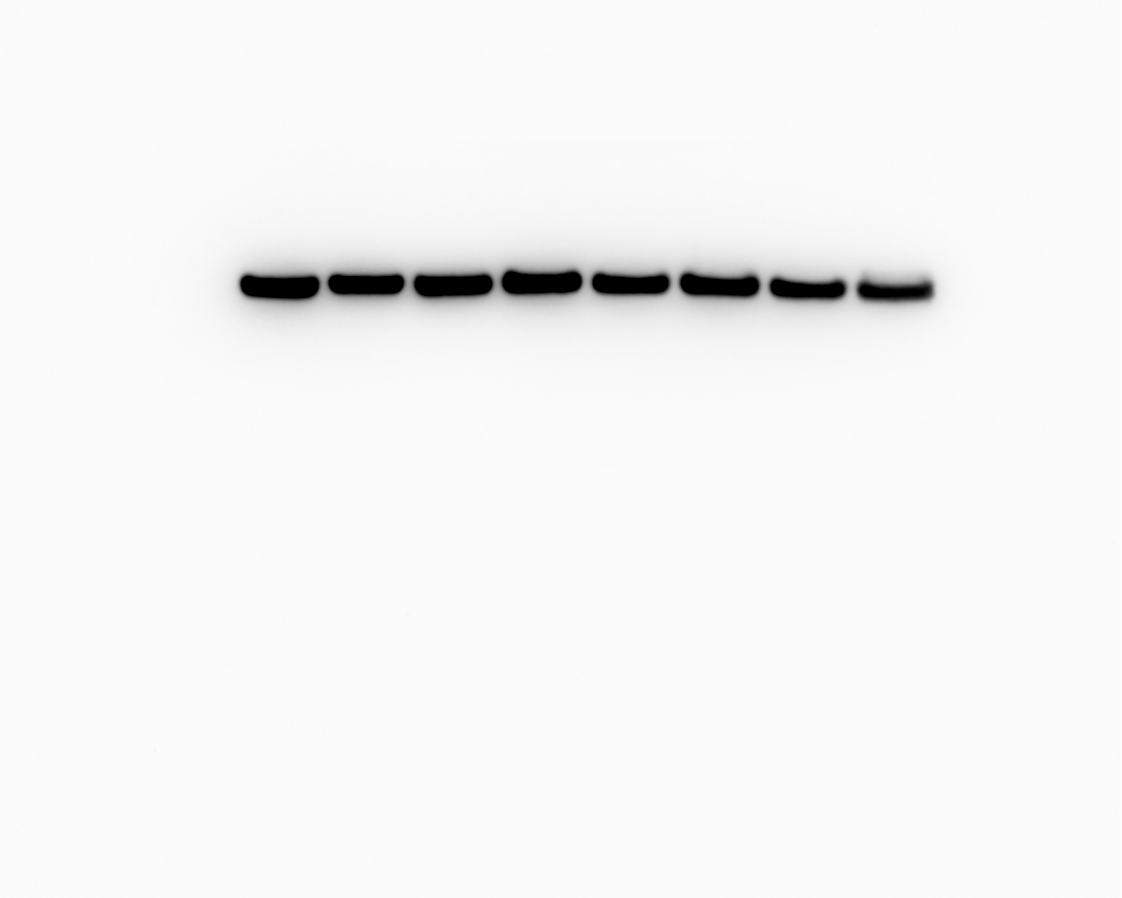

Supplement: Supplementary file 3 — Source data Fig. 2 [file 44319_2026_793_MOESM3_ESM.zip › Figure 2/Fig2F/ACTIN.tif]

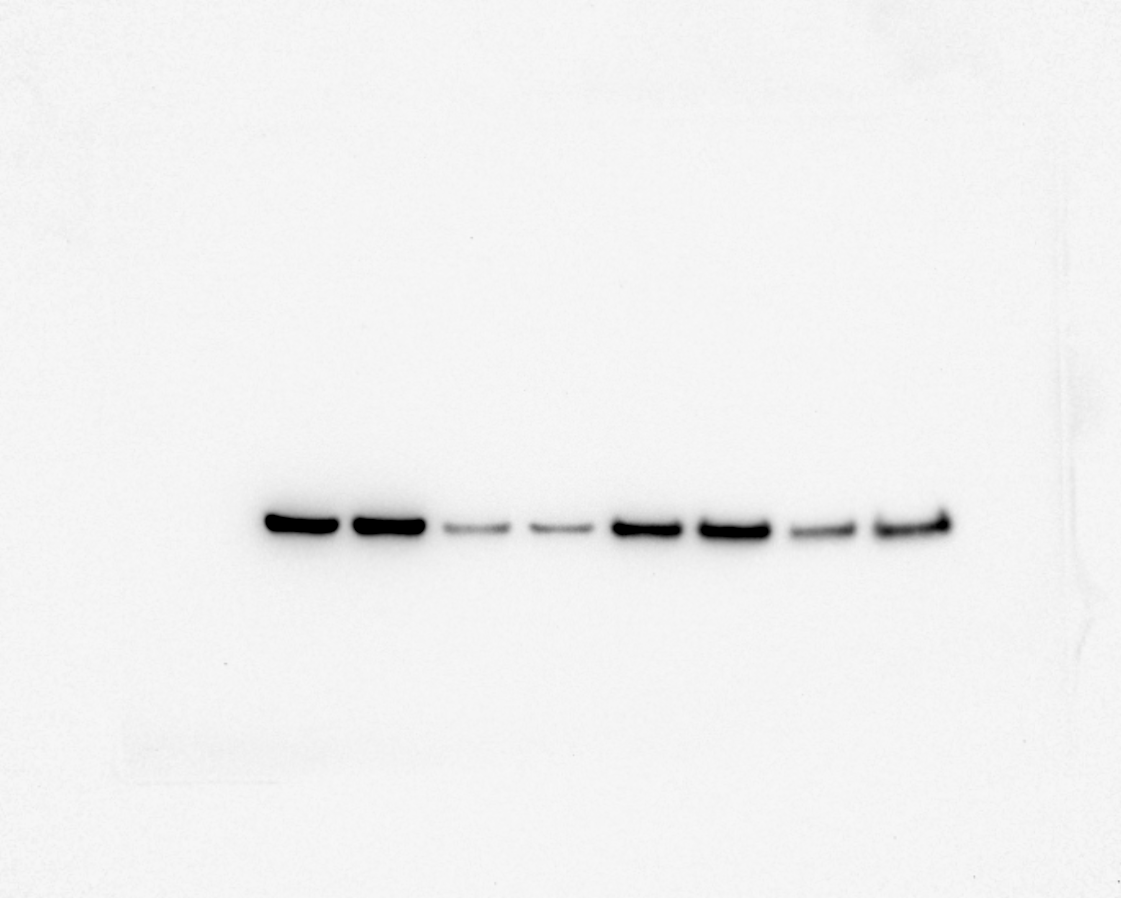

Supplement: Supplementary file 3 — Source data Fig. 2 [file 44319_2026_793_MOESM3_ESM.zip › Figure 2/Fig2F/IkBa.tif]

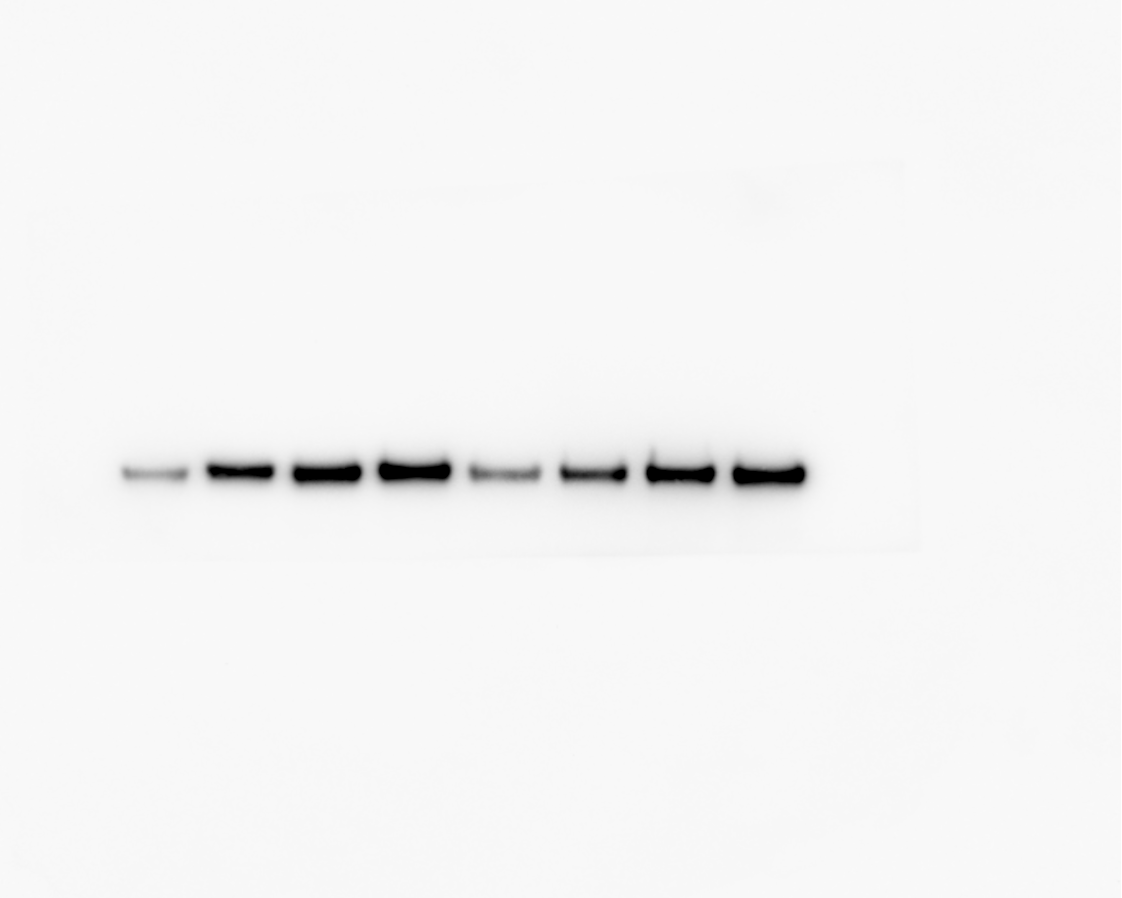

Supplement: Supplementary file 3 — Source data Fig. 2 [file 44319_2026_793_MOESM3_ESM.zip › Figure 2/Fig2F/P-p65.tif]

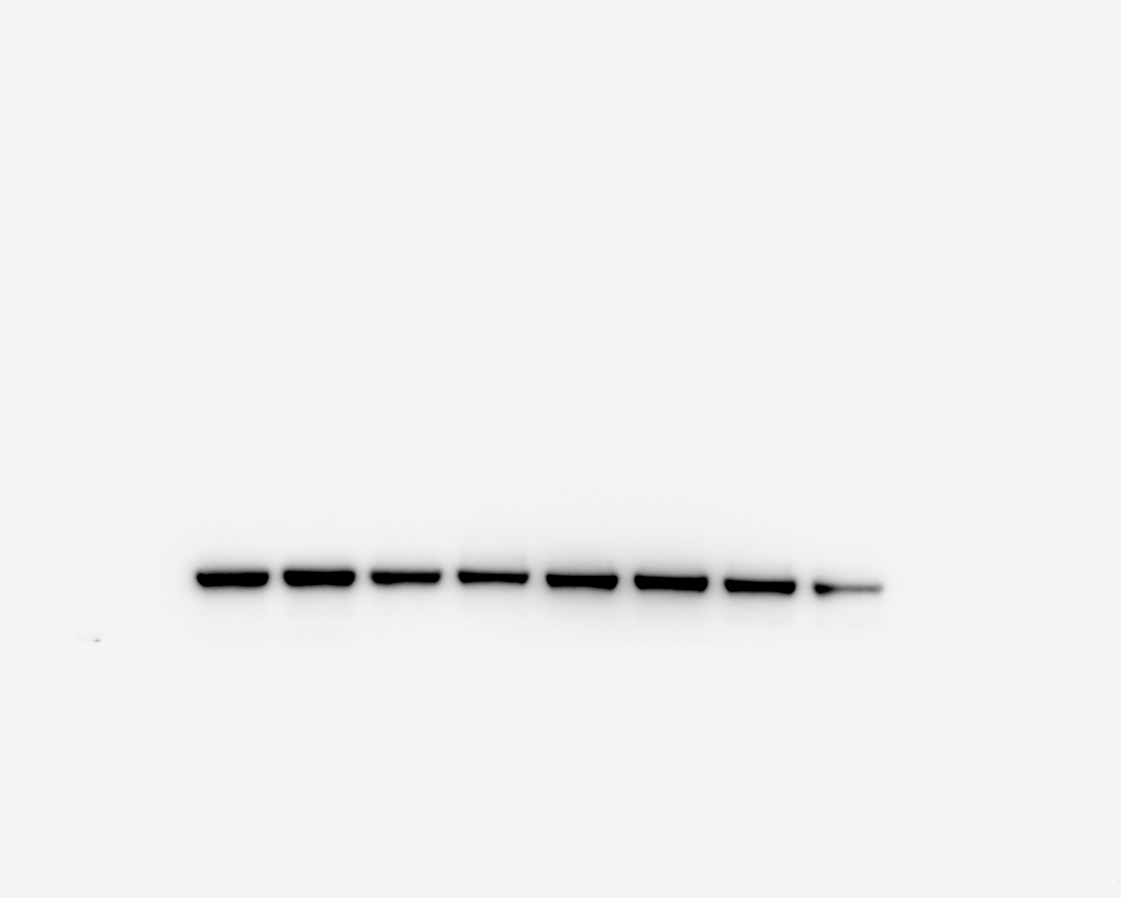

Supplement: Supplementary file 3 — Source data Fig. 2 [file 44319_2026_793_MOESM3_ESM.zip › Figure 2/Fig2F/TBK1.tif]

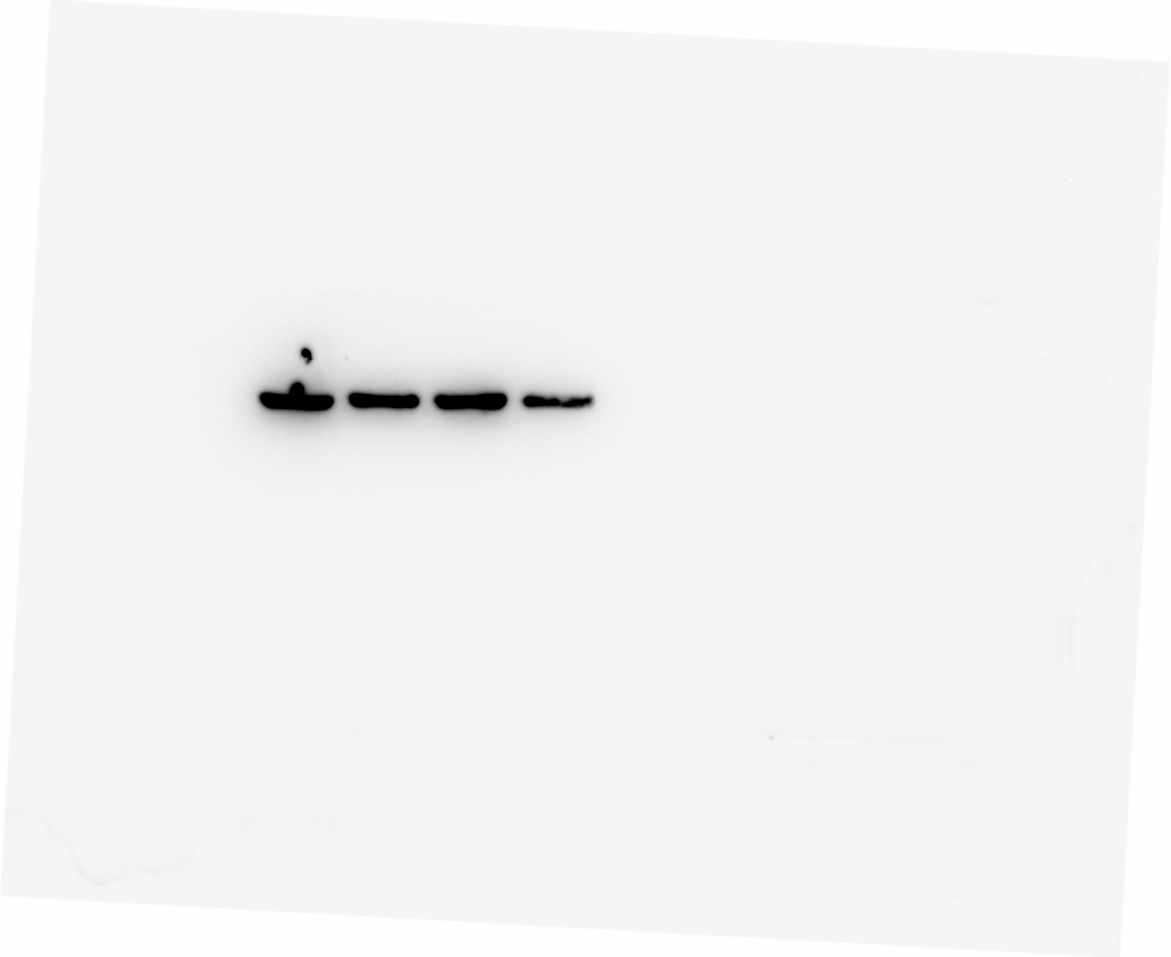

Supplement: Supplementary file 3 — Source data Fig. 2 [file 44319_2026_793_MOESM3_ESM.zip › Figure 2/Fig2F/IRF3.tif]

Figure 2F\_uncropped blots

| 2'3'-cGAM(PS)2: | iBMDMs |   |   |   |                    |   |   |   |
|-----------------|--------|---|---|---|--------------------|---|---|---|
|                 | WT     |   |   |   | IRF3 <sup>ko</sup> |   |   |   |
|                 | 0      | 1 | 2 | 3 | 0                  | 1 | 2 | 3 |
|                 |        |   |   |   |                    |   |   |   |

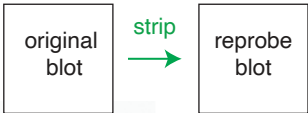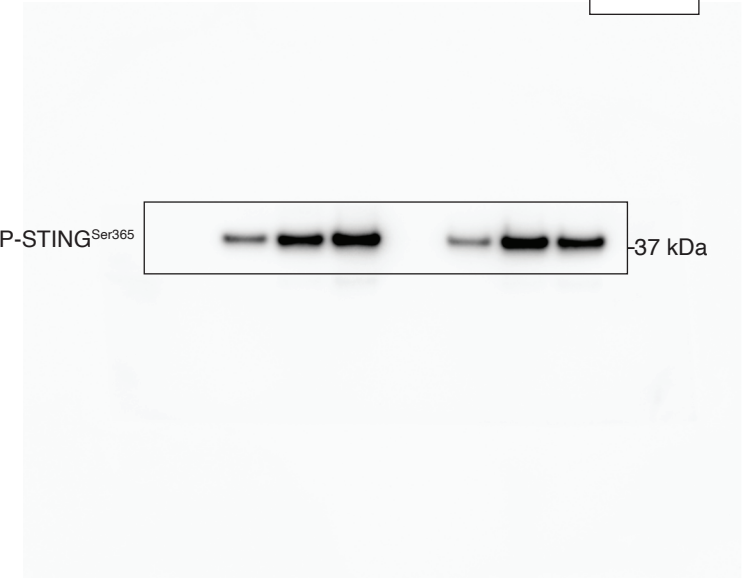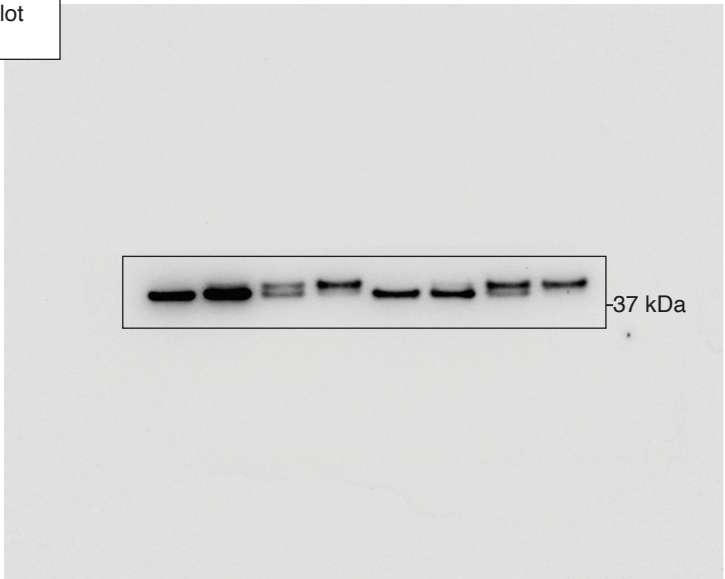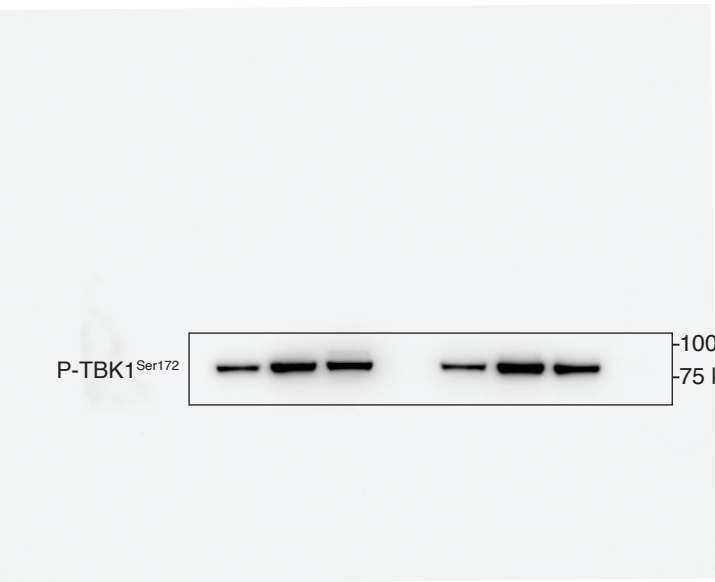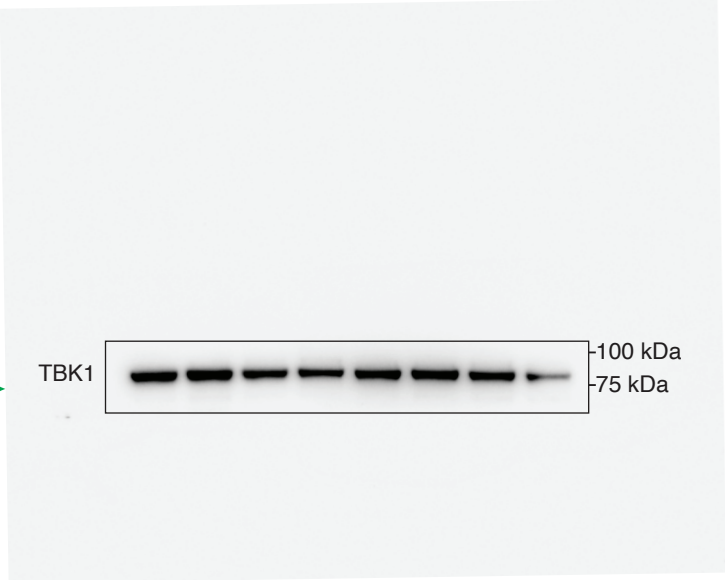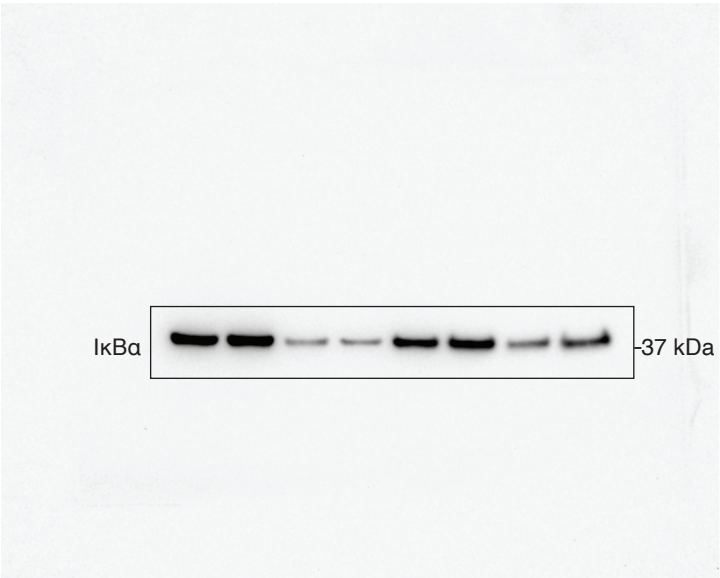

Figure 2F\_uncropped blots

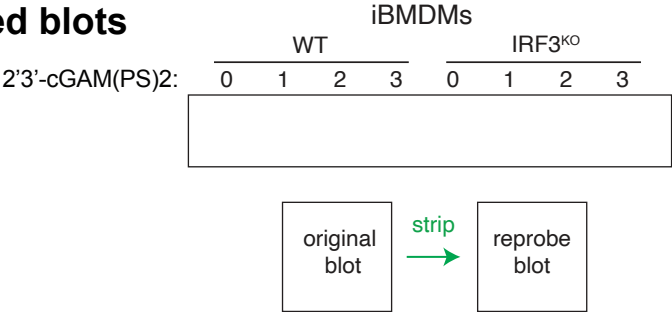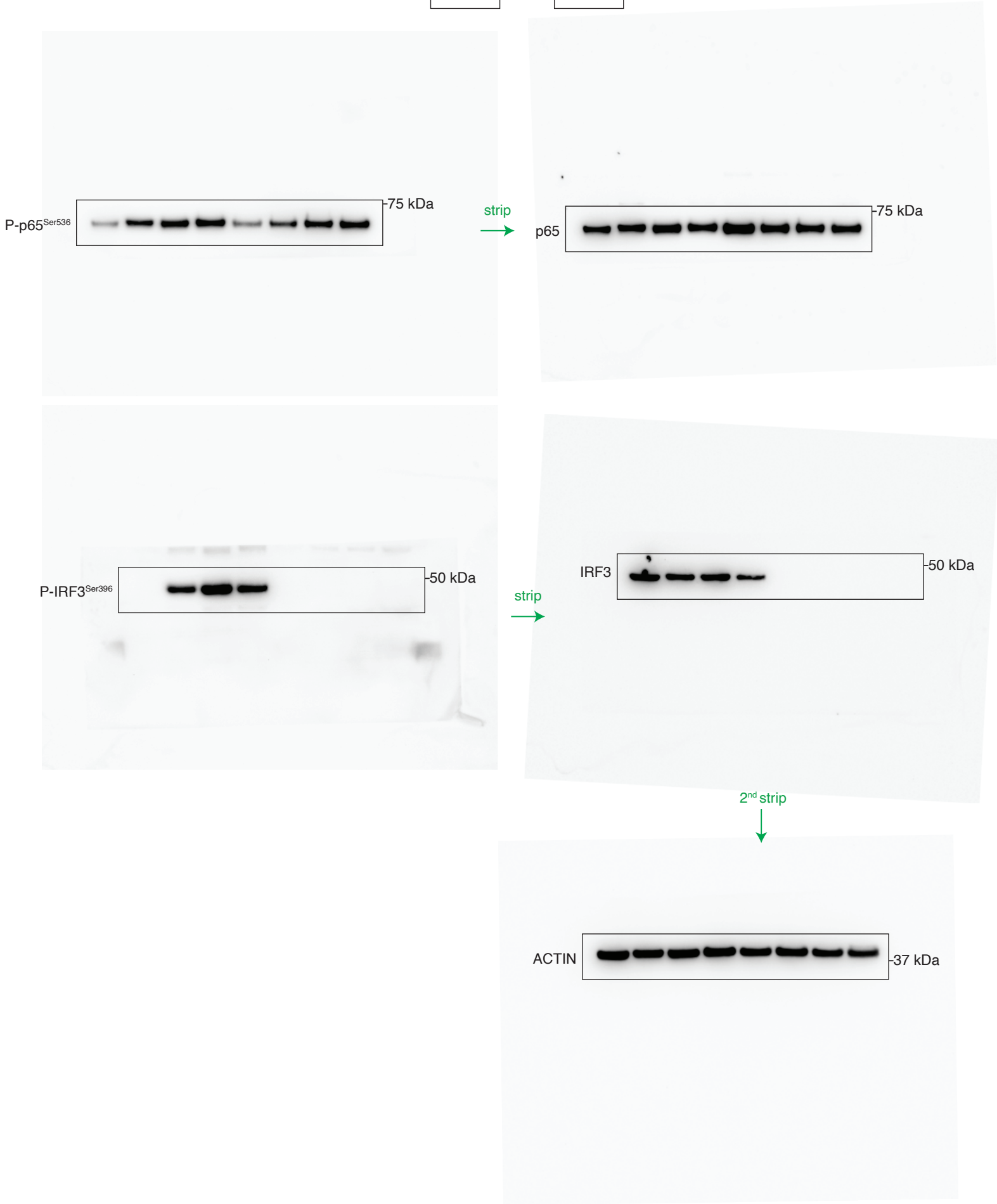

Supplement: Supplementary file 3 — Source data Fig. 2 [file 44319_2026_793_MOESM3_ESM.zip › Figure 2/Fig2F/Fig2F_uncropped blots.pdf]

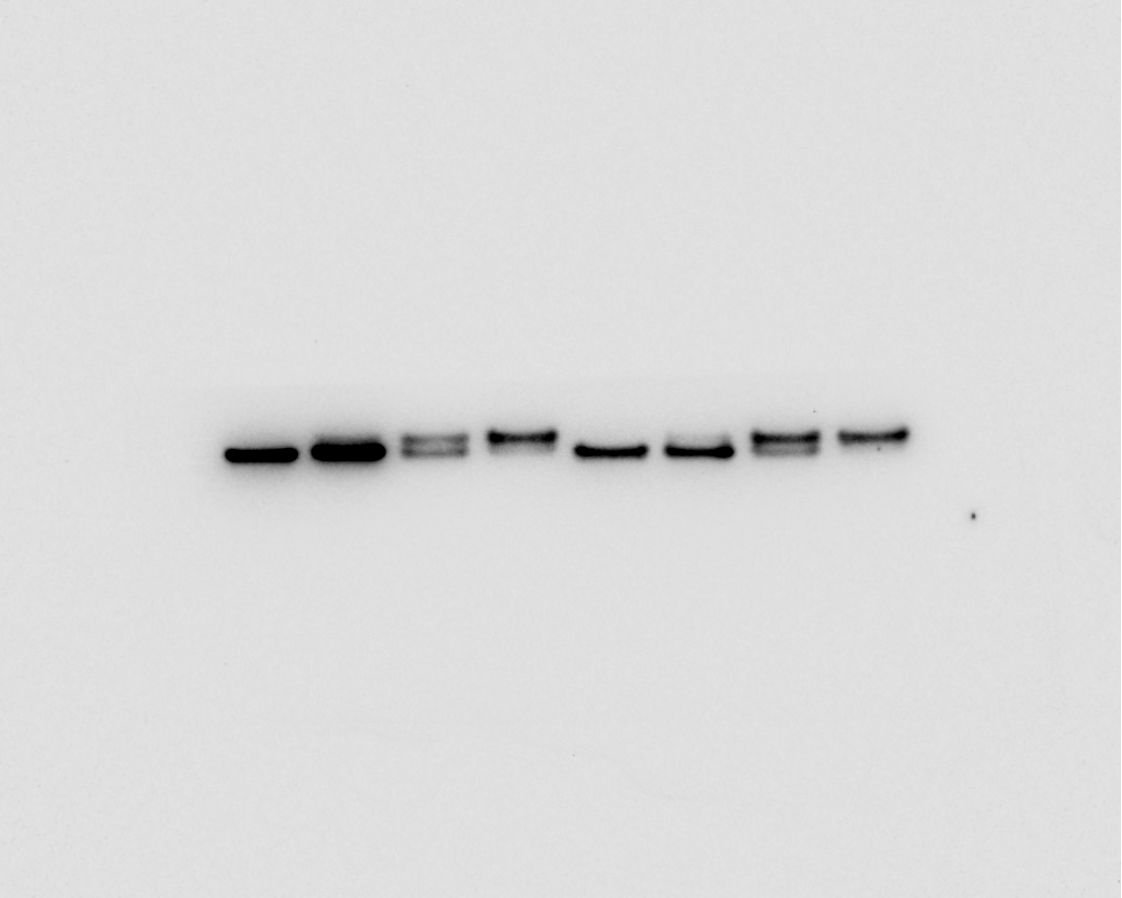

Supplement: Supplementary file 3 — Source data Fig. 2 [file 44319_2026_793_MOESM3_ESM.zip › Figure 2/Fig2F/STING.tif]

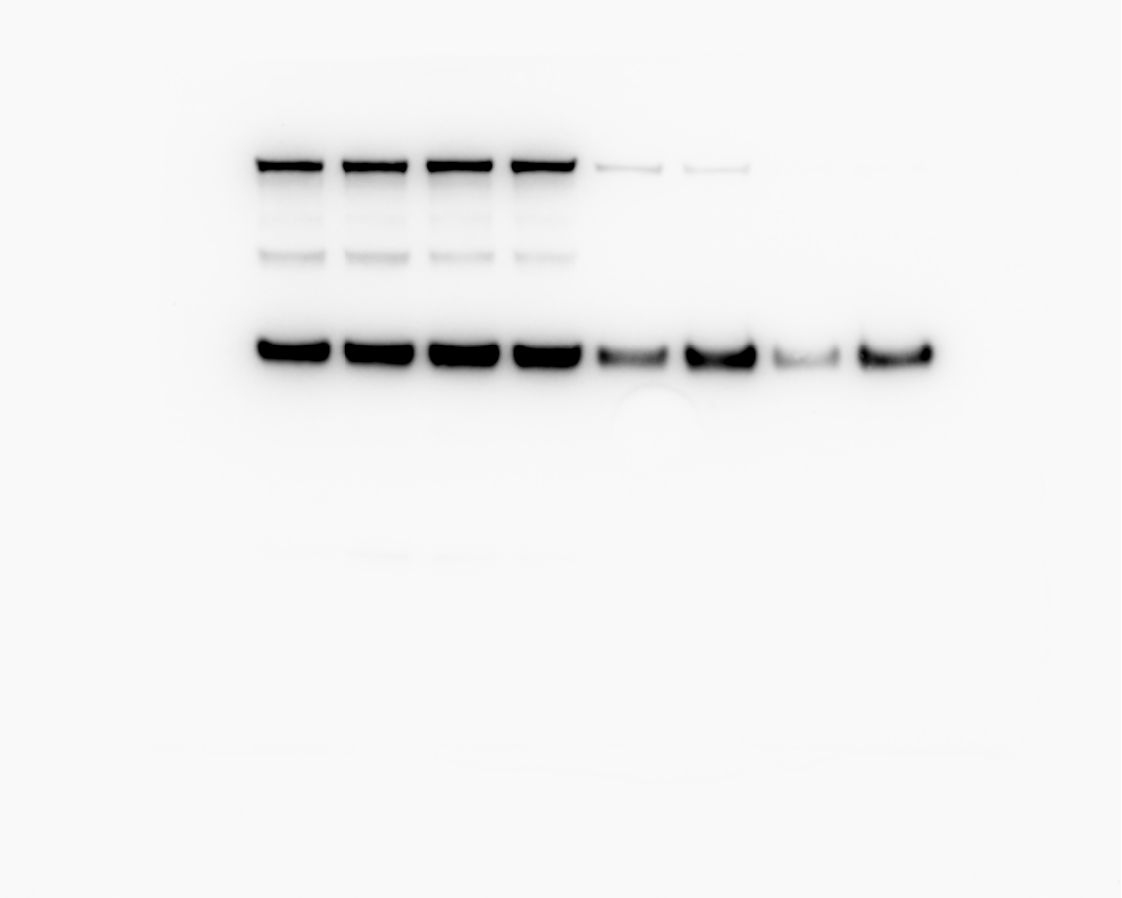

Supplement: Supplementary file 3 — Source data Fig. 2 [file 44319_2026_793_MOESM3_ESM.zip › Figure 2/Fig2G/p105:p50.tif]

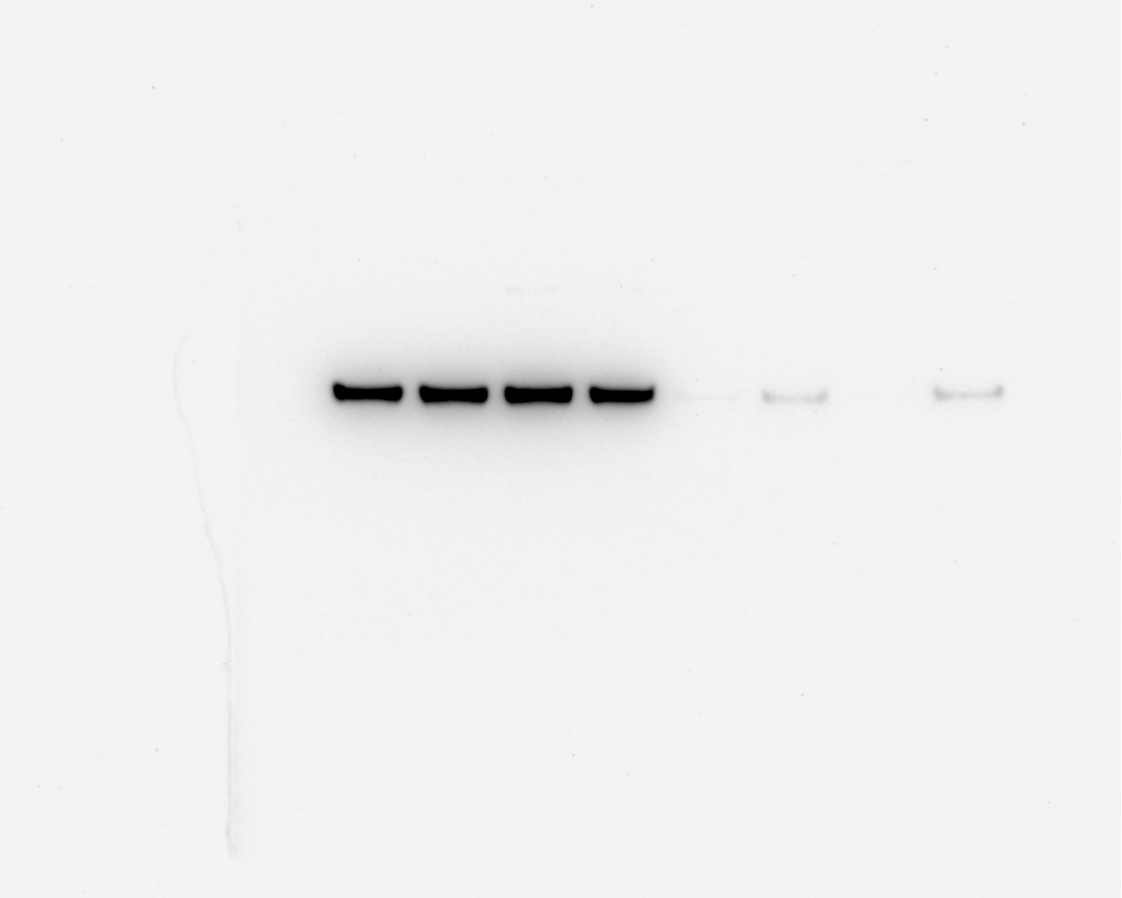

Supplement: Supplementary file 3 — Source data Fig. 2 [file 44319_2026_793_MOESM3_ESM.zip › Figure 2/Fig2G/p65 short exposure.tif]

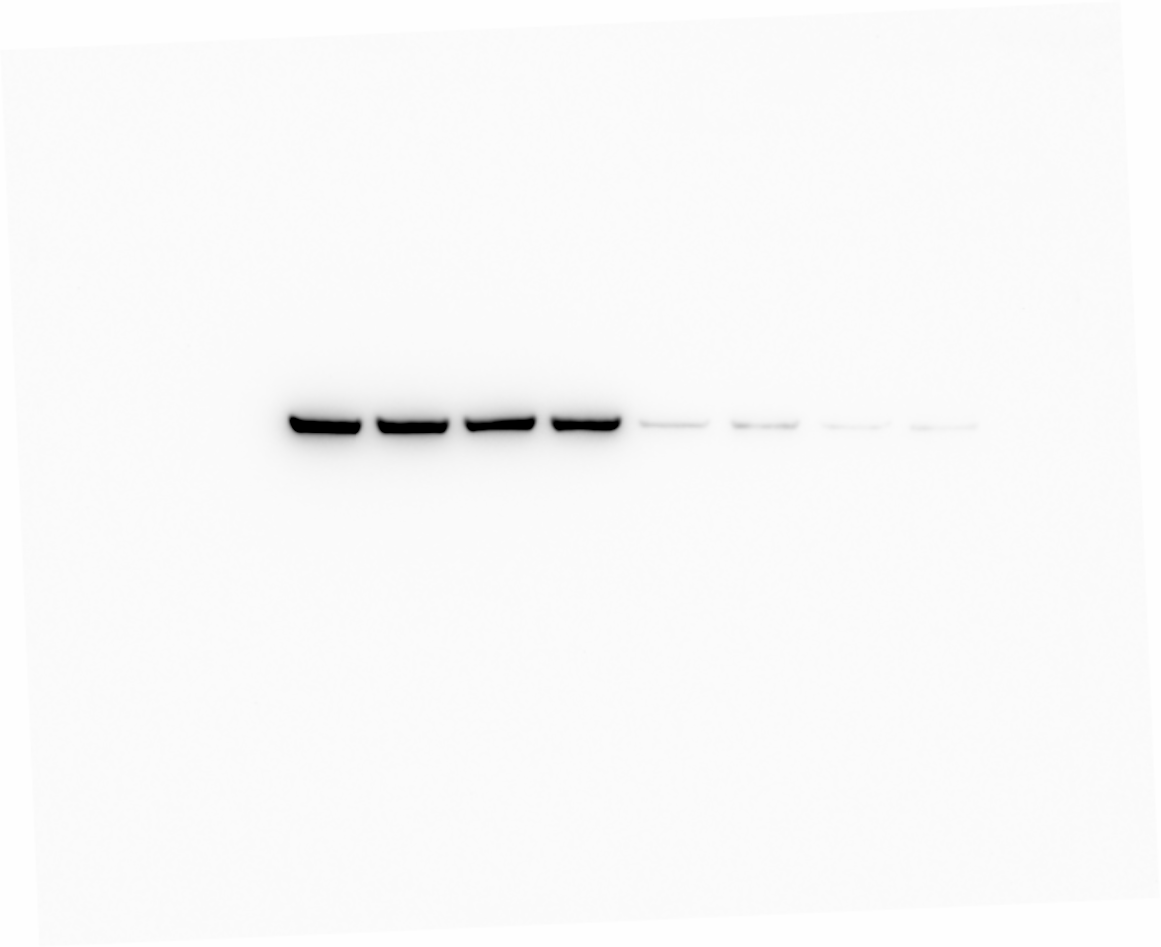

Supplement: Supplementary file 3 — Source data Fig. 2 [file 44319_2026_793_MOESM3_ESM.zip › Figure 2/Fig2G/HSP70.tif]

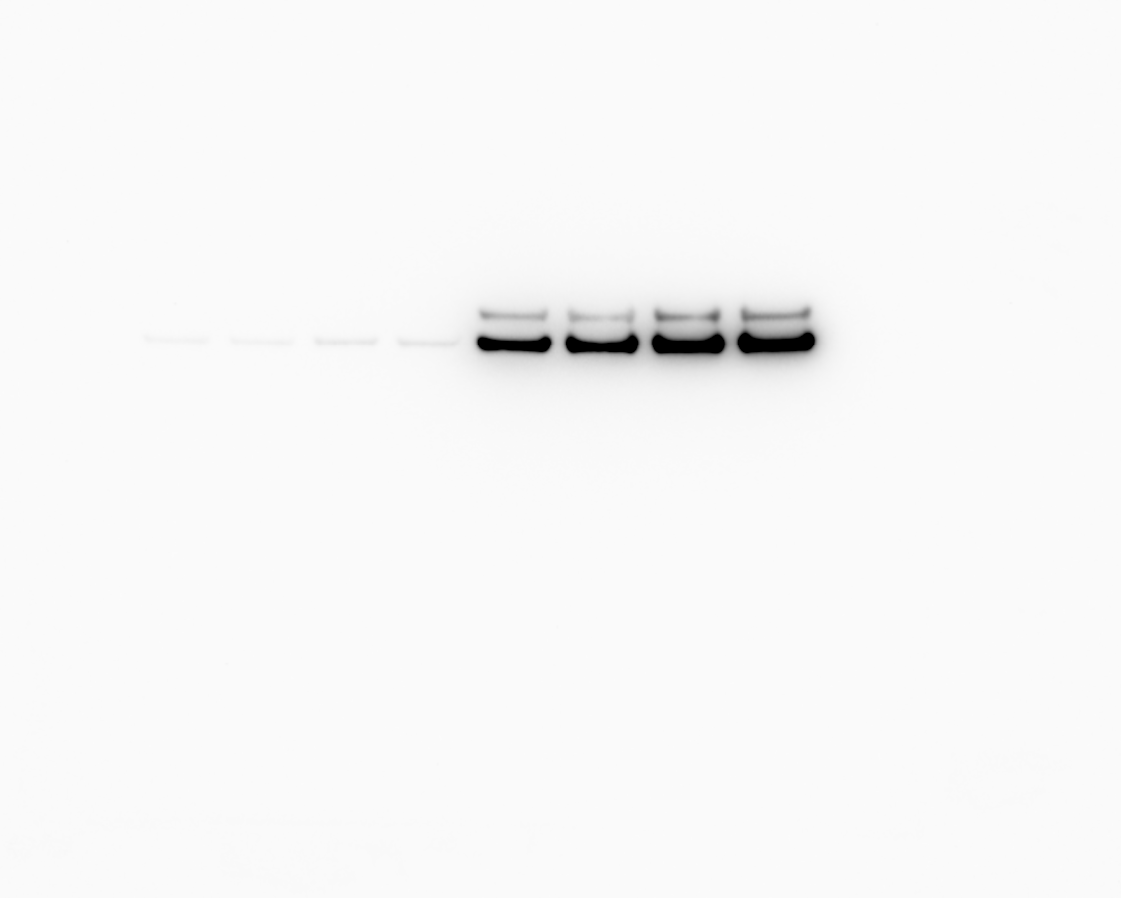

Supplement: Supplementary file 3 — Source data Fig. 2 [file 44319_2026_793_MOESM3_ESM.zip › Figure 2/Fig2G/LAMIN A:C.tif]

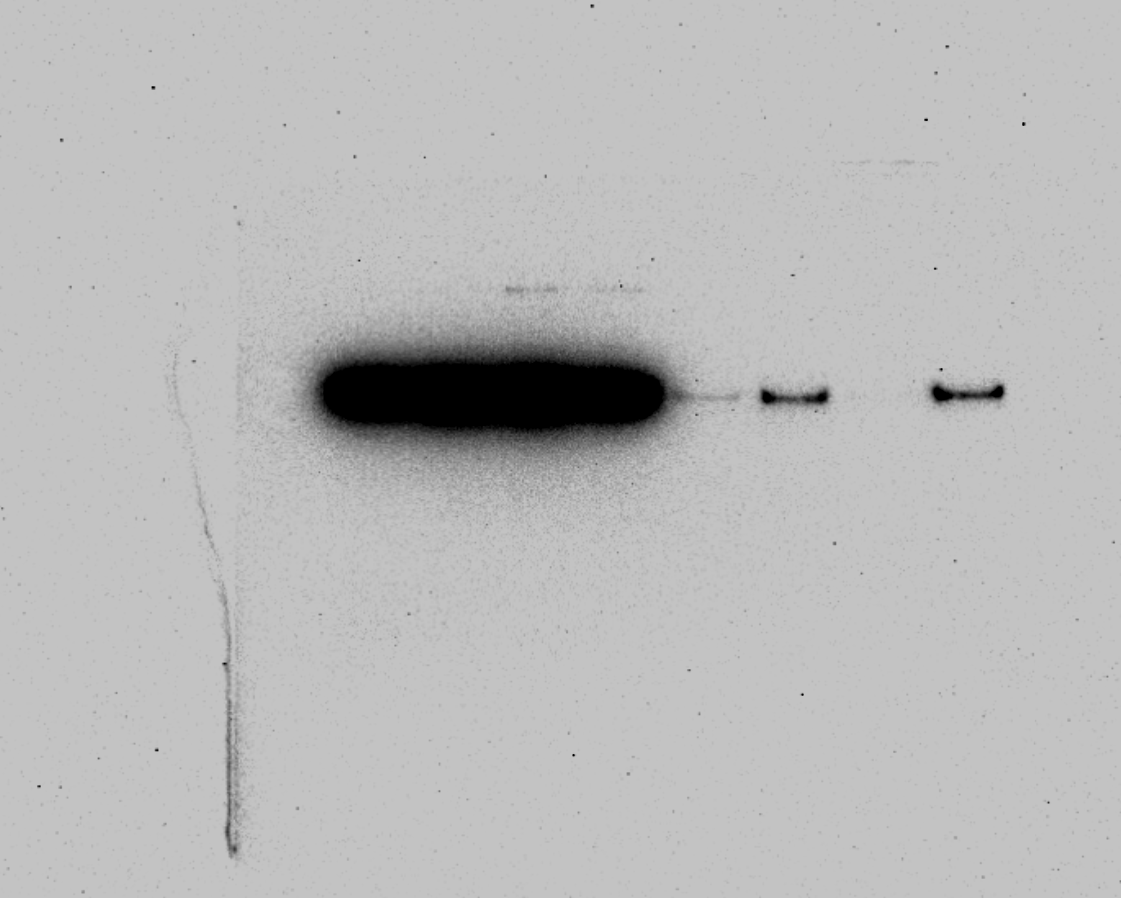

Supplement: Supplementary file 3 — Source data Fig. 2 [file 44319_2026_793_MOESM3_ESM.zip › Figure 2/Fig2G/p65 long exposure.tif]

Figure 2G\_uncropped blots

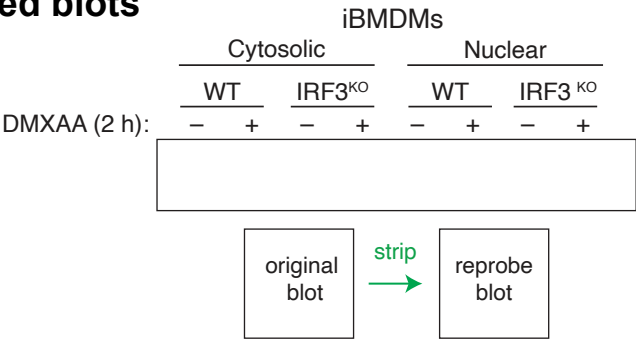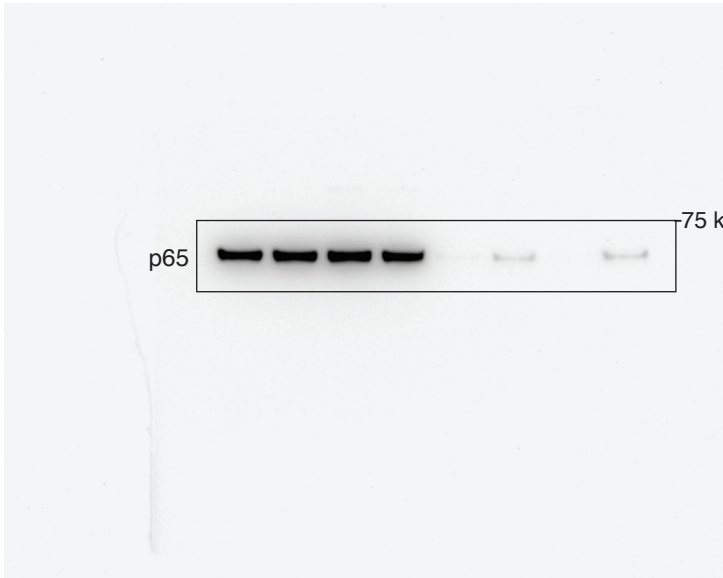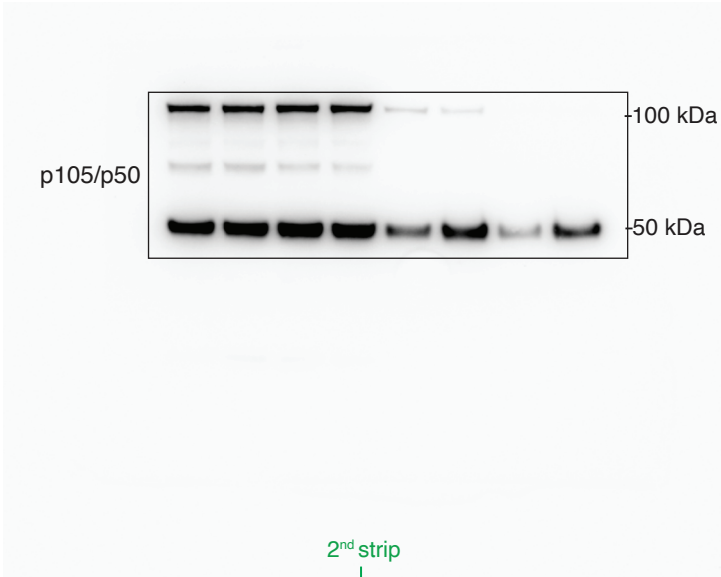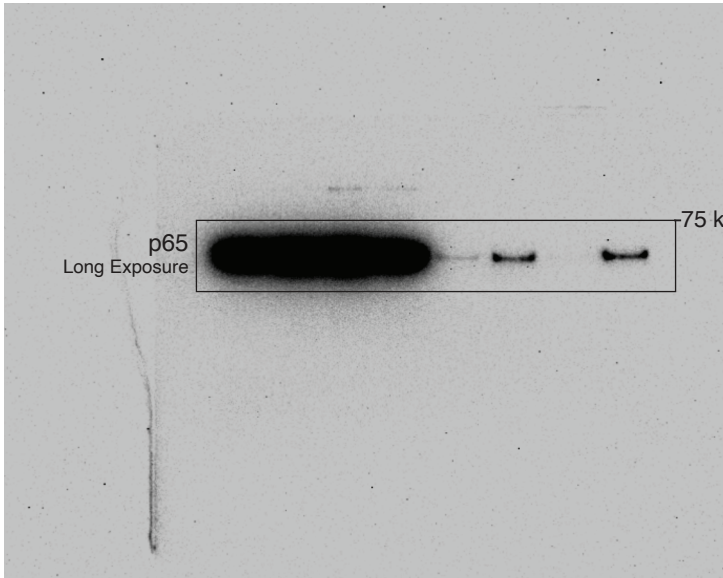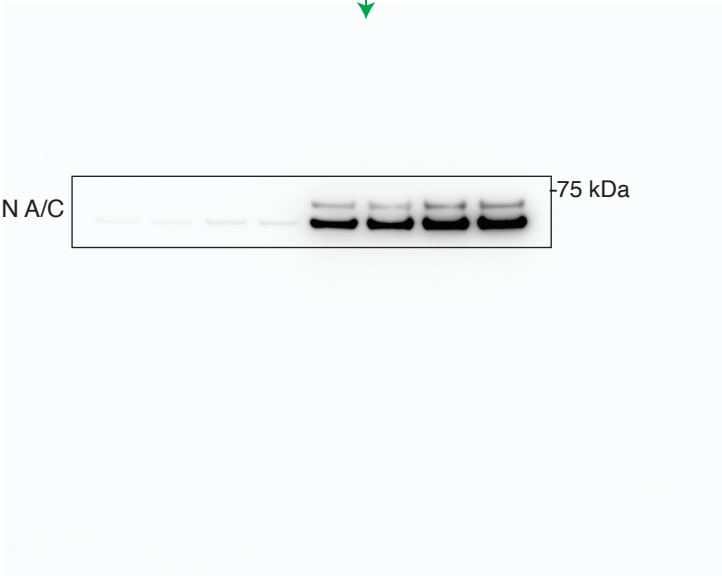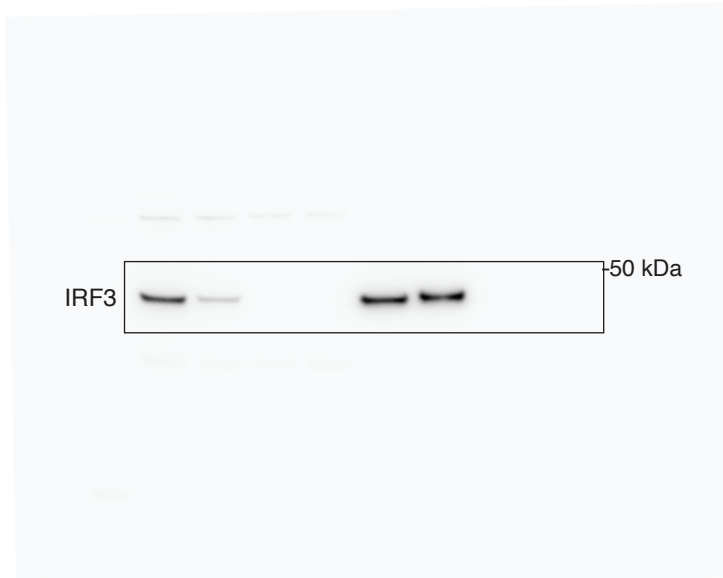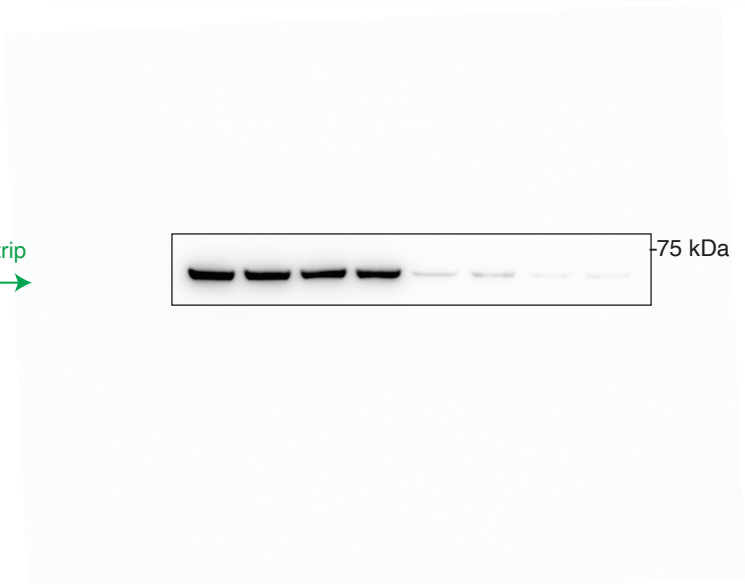

Supplement: Supplementary file 3 — Source data Fig. 2 [file 44319_2026_793_MOESM3_ESM.zip › Figure 2/Fig2G/Fig2G_uncropped blots.pdf]

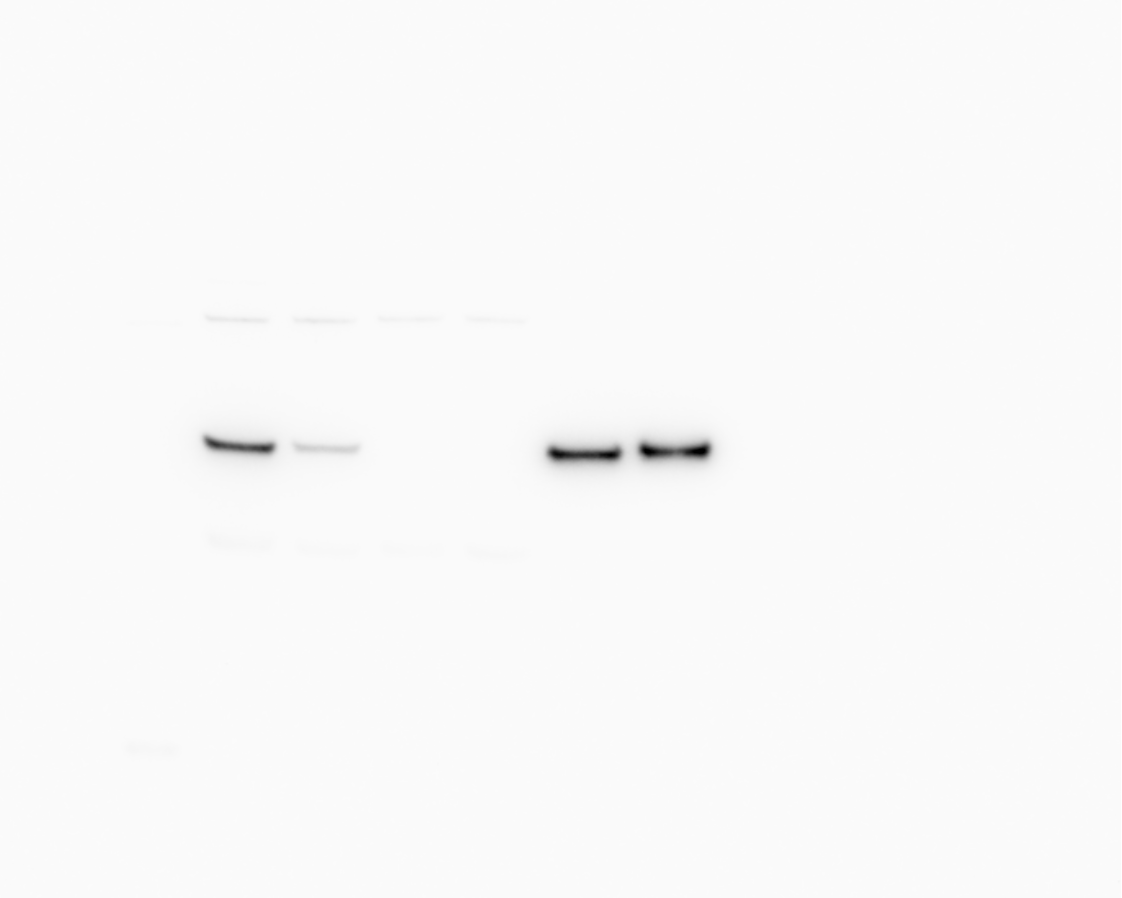

Supplement: Supplementary file 3 — Source data Fig. 2 [file 44319_2026_793_MOESM3_ESM.zip › Figure 2/Fig2G/IRF3.tif]

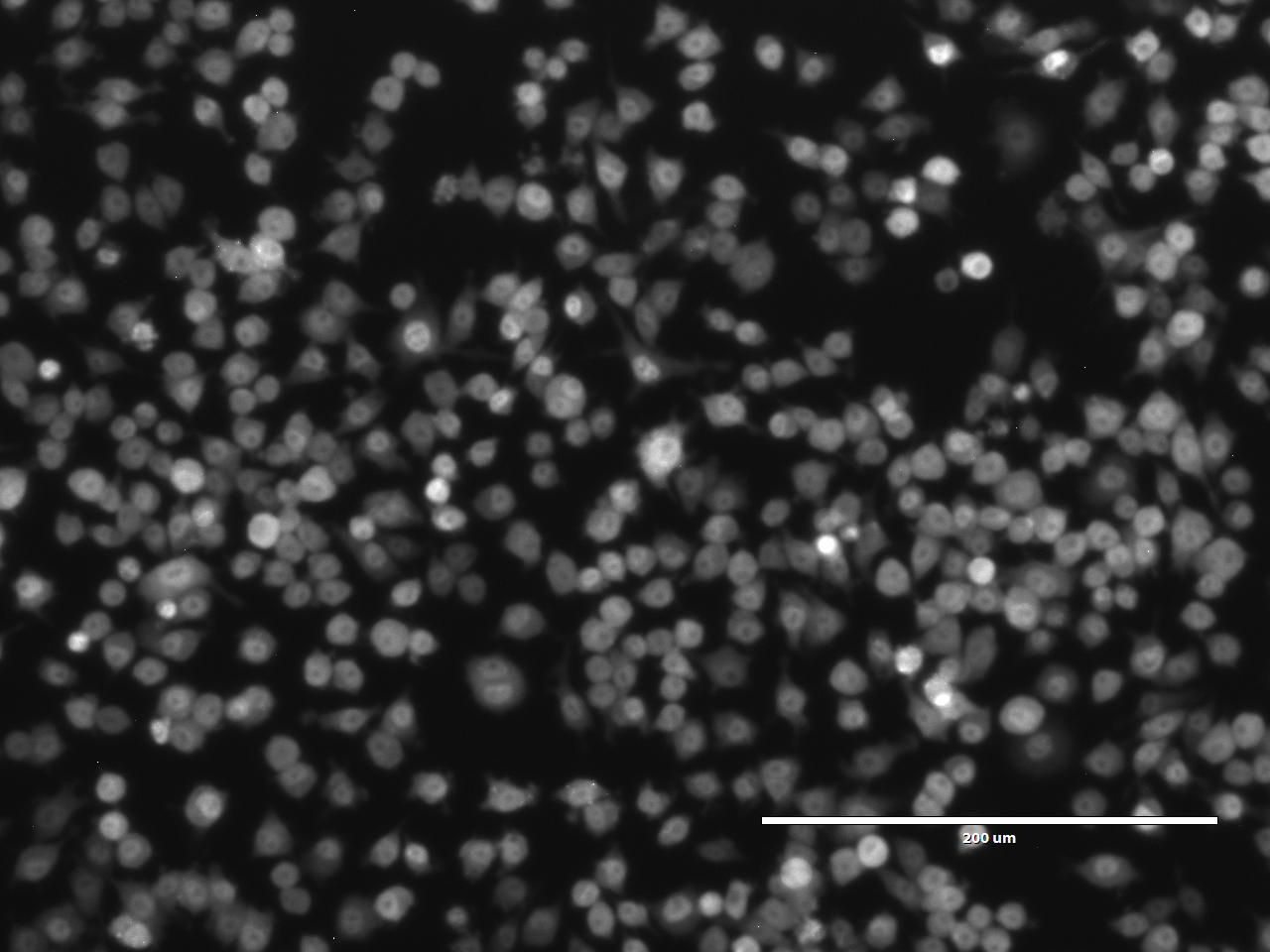

Supplement: Supplementary file 4 — Source data Fig. 3 [file 44319_2026_793_MOESM4_ESM.zip › Figure 3/Fig3A/WT GFP-IRF3 DMXAA.tif]

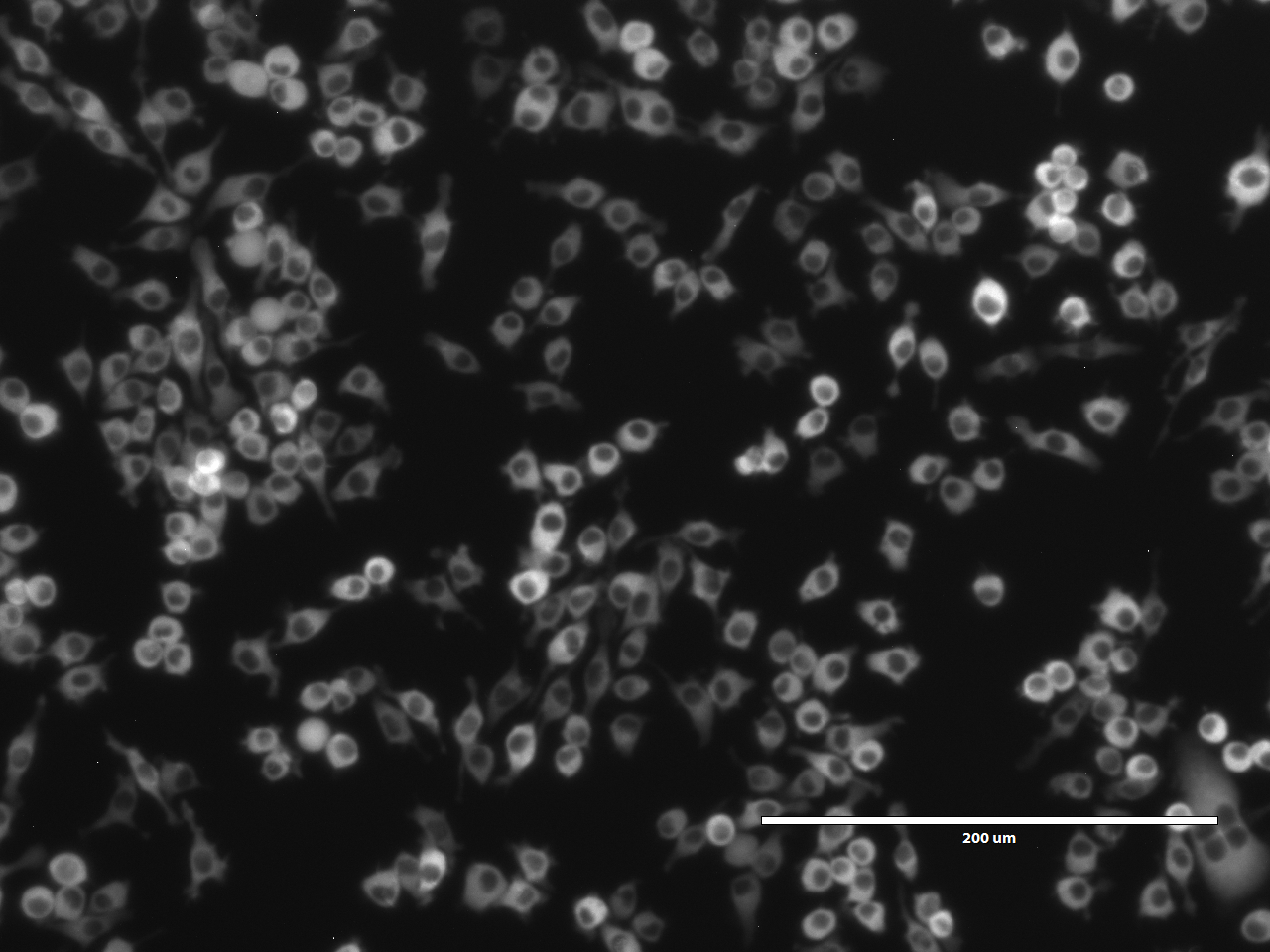

Supplement: Supplementary file 4 — Source data Fig. 3 [file 44319_2026_793_MOESM4_ESM.zip › Figure 3/Fig3A/TBK1 KO GFP-IRF3 UT.tif.tif]

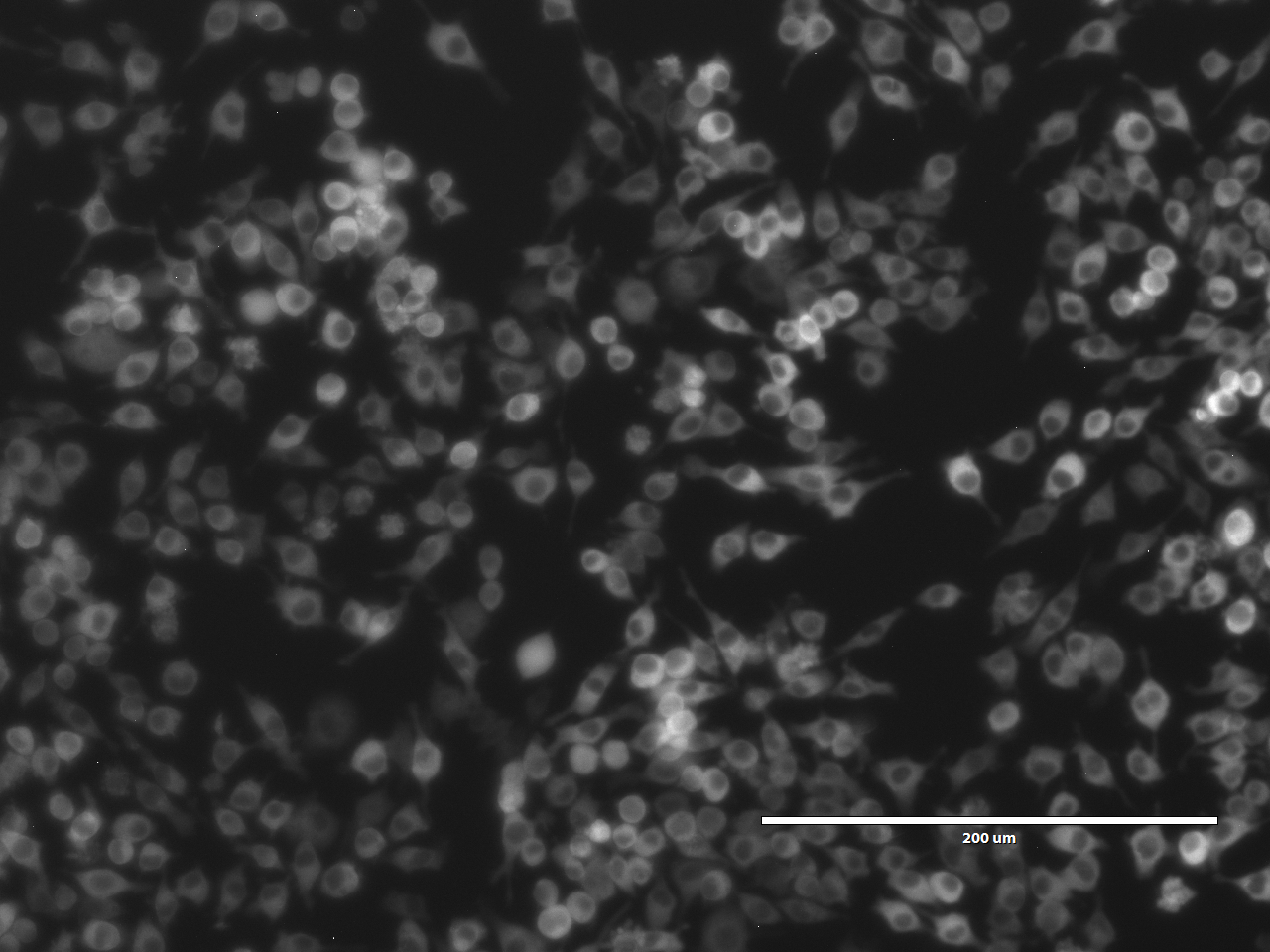

Supplement: Supplementary file 4 — Source data Fig. 3 [file 44319_2026_793_MOESM4_ESM.zip › Figure 3/Fig3A/TBK1 KO GFP-IRF3 cGAMP.tif]

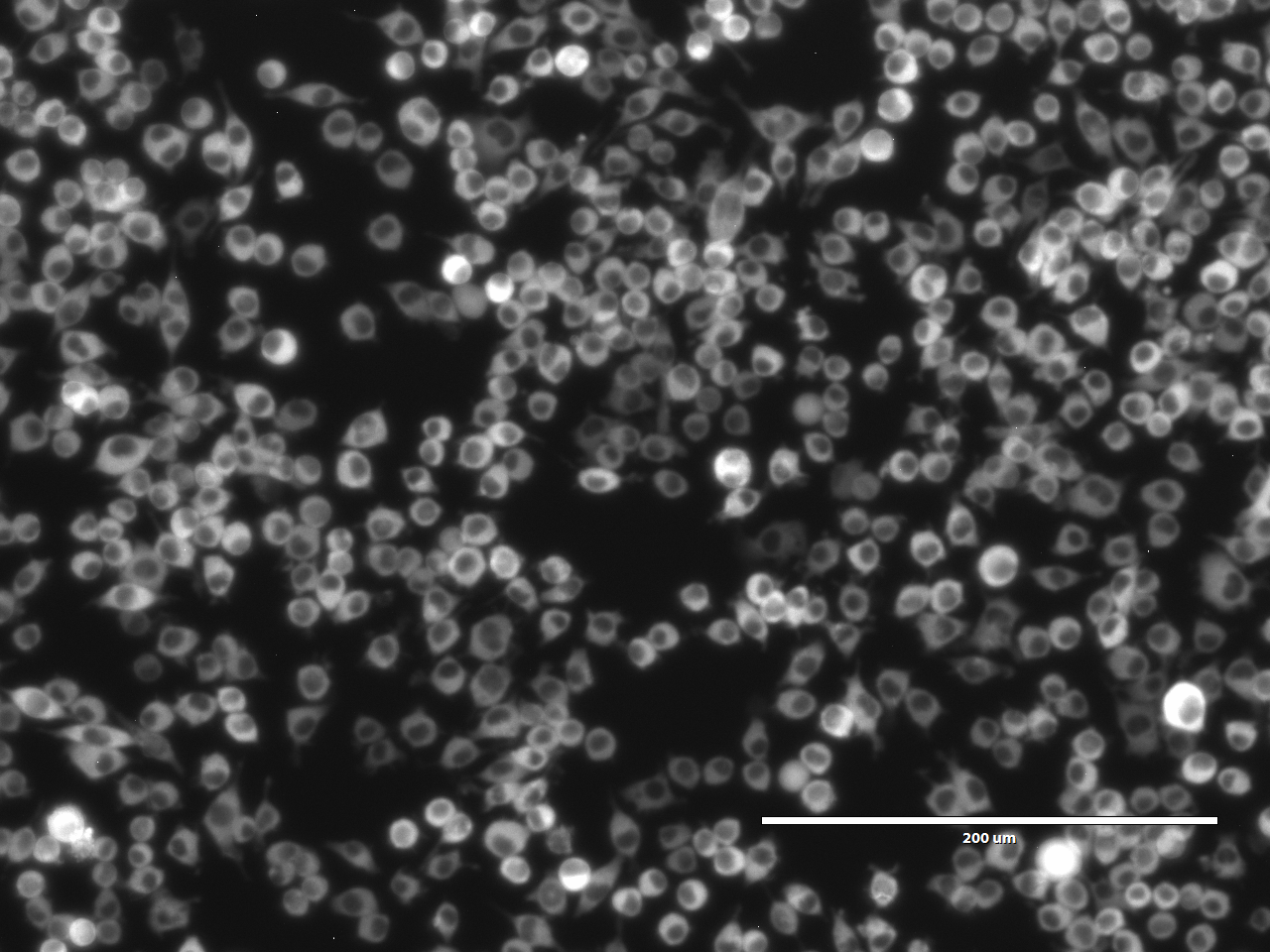

Supplement: Supplementary file 4 — Source data Fig. 3 [file 44319_2026_793_MOESM4_ESM.zip › Figure 3/Fig3A/WT GFP-IRF3 UT.tif]

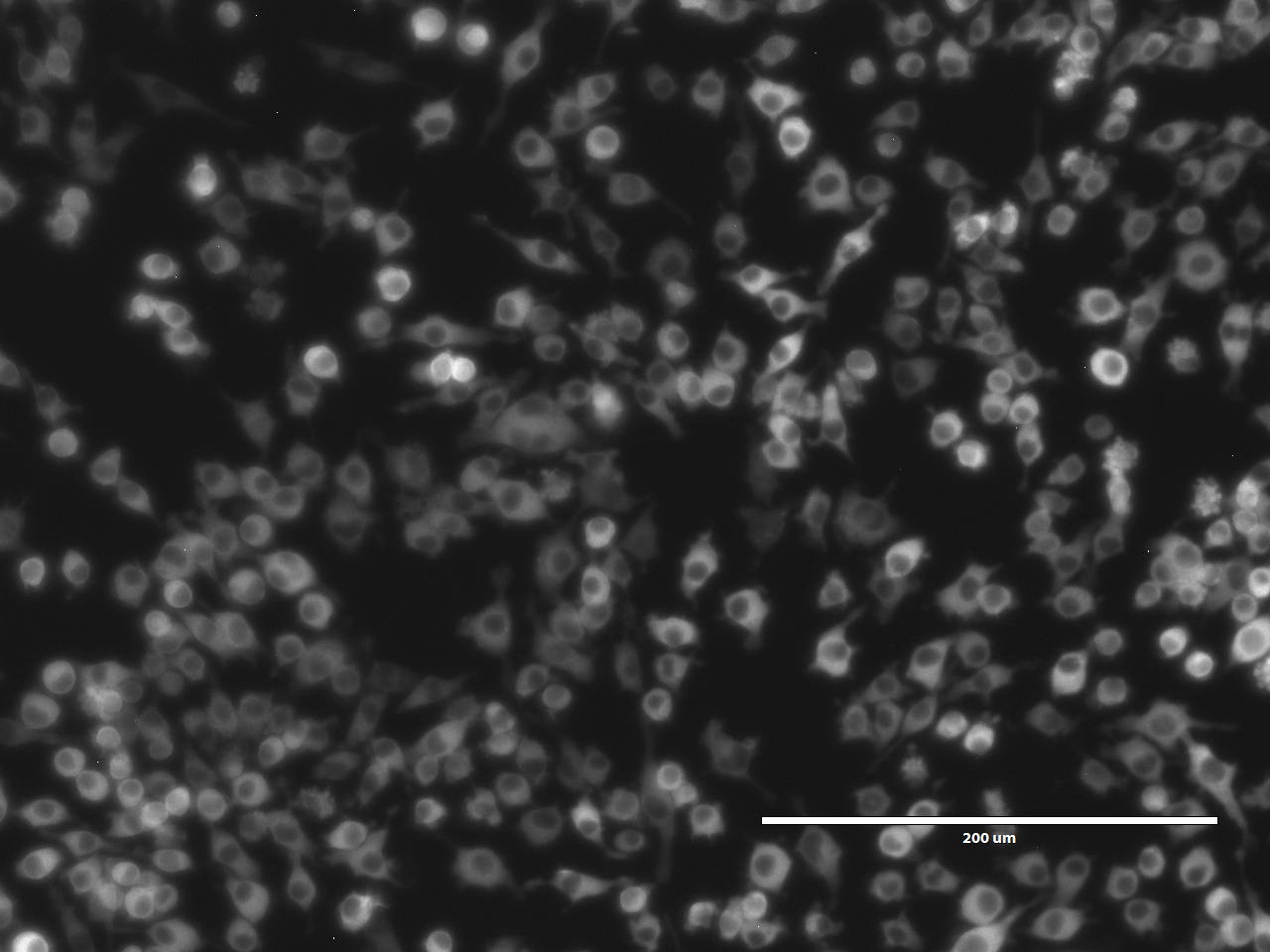

Supplement: Supplementary file 4 — Source data Fig. 3 [file 44319_2026_793_MOESM4_ESM.zip › Figure 3/Fig3A/TBK1 KO GFP-IRF3 DMXAA.tif]

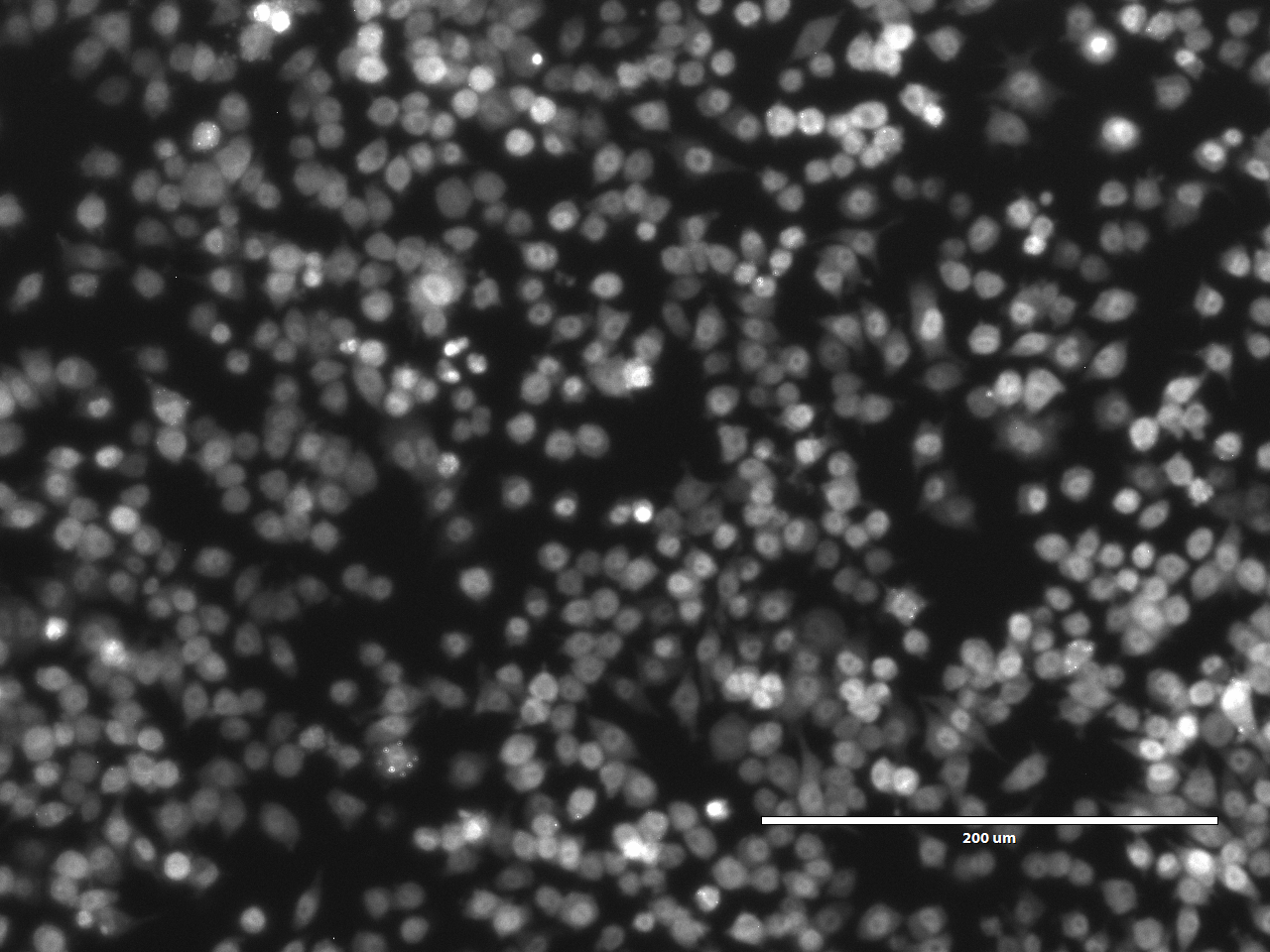

Supplement: Supplementary file 4 — Source data Fig. 3 [file 44319_2026_793_MOESM4_ESM.zip › Figure 3/Fig3A/WT GFP-IRF3 cGAMP.tif]

Figure 3A\_uncropped images

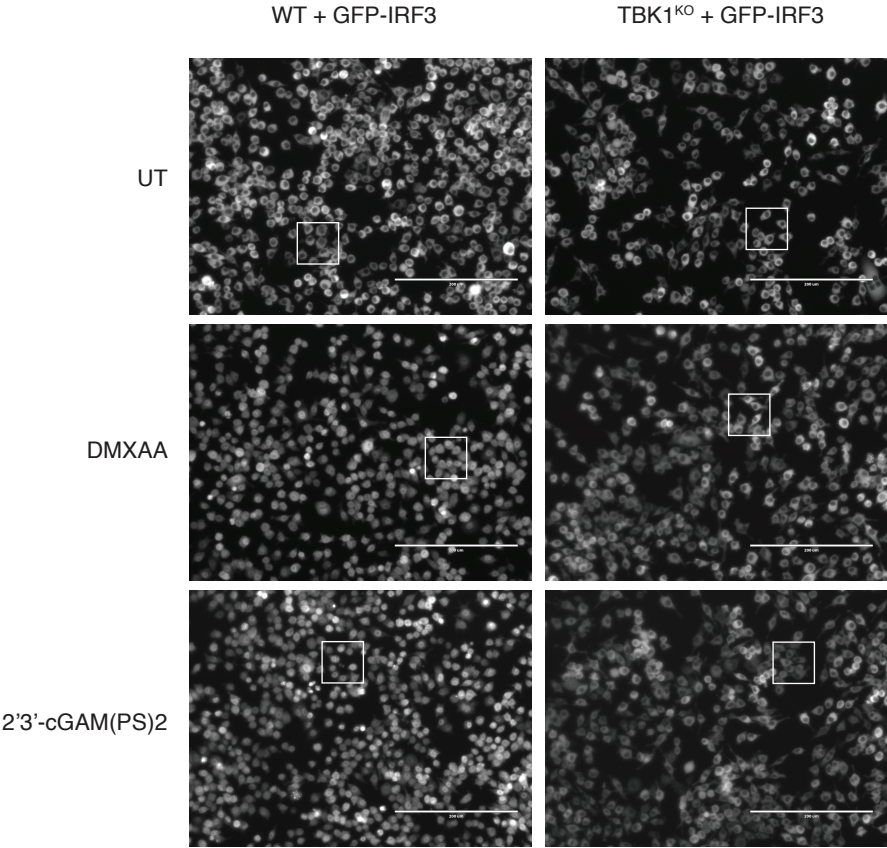

Supplement: Supplementary file 4 — Source data Fig. 3 [file 44319_2026_793_MOESM4_ESM.zip › Figure 3/Fig3A/Fig3A_uncropped images.pdf]

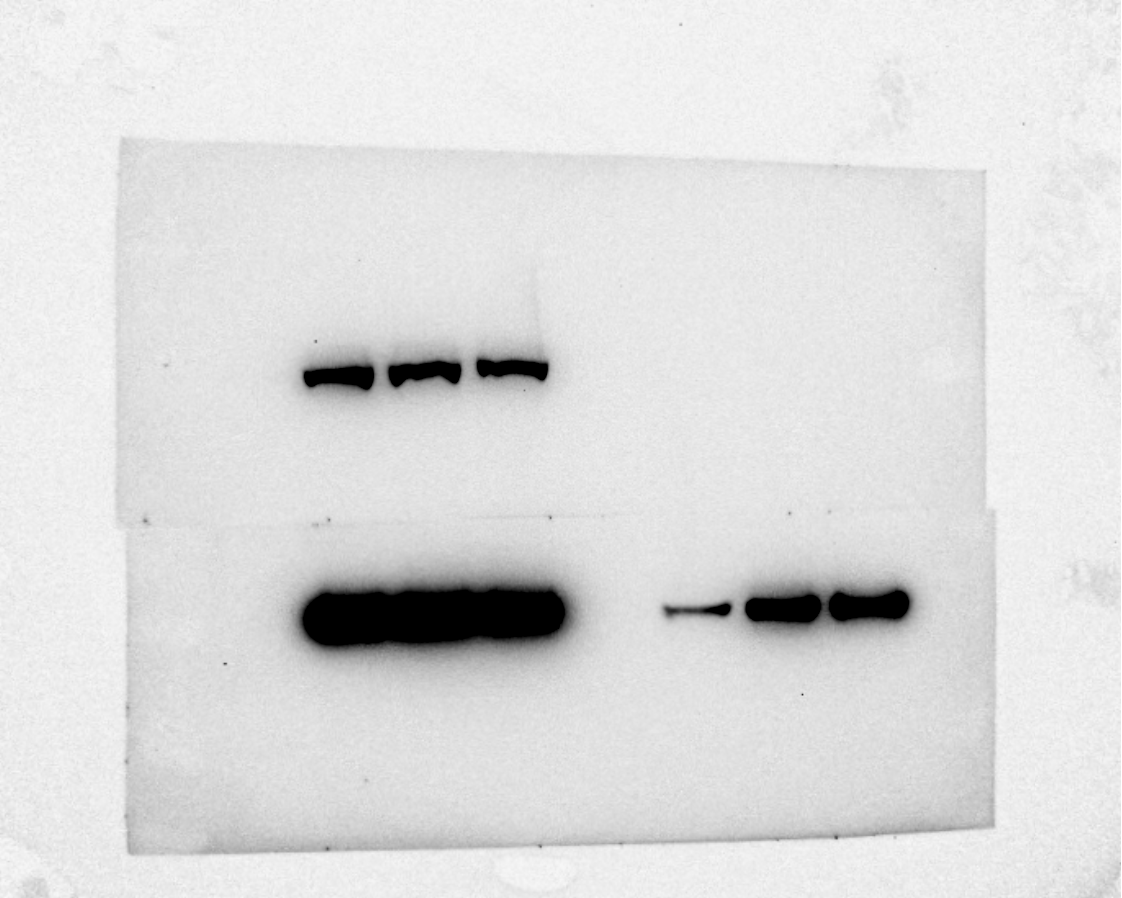

Supplement: Supplementary file 4 — Source data Fig. 3 [file 44319_2026_793_MOESM4_ESM.zip › Figure 3/Fig3D/P-TBK1.tif]

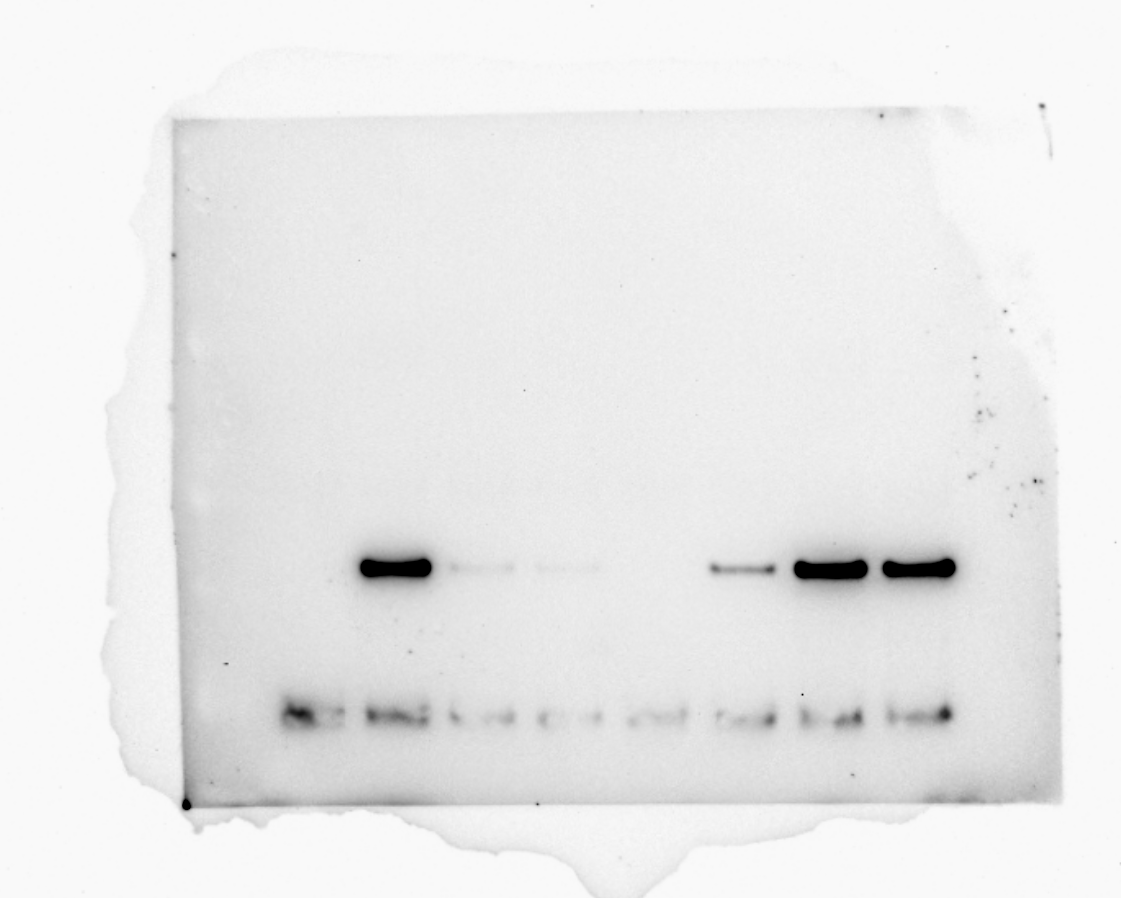

Supplement: Supplementary file 4 — Source data Fig. 3 [file 44319_2026_793_MOESM4_ESM.zip › Figure 3/Fig3D/GFP IP STING.tif]

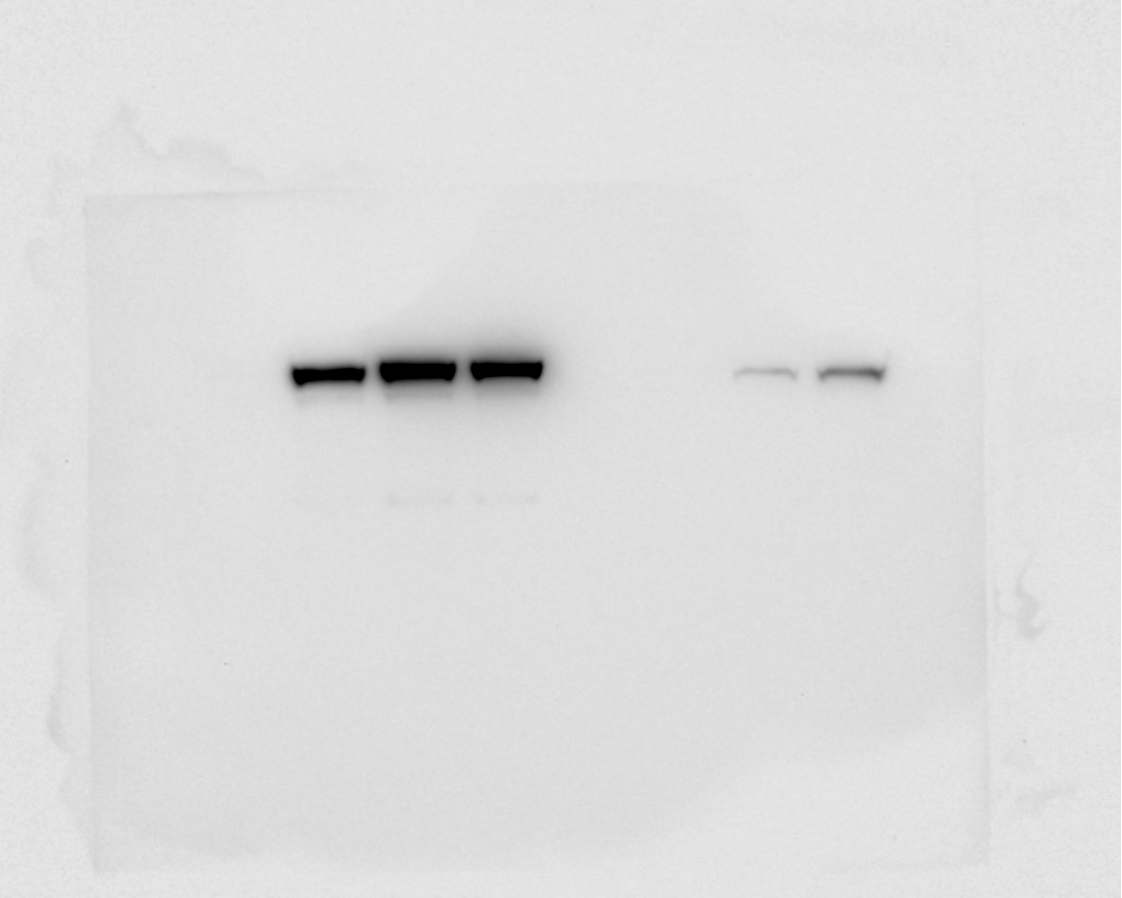

Supplement: Supplementary file 4 — Source data Fig. 3 [file 44319_2026_793_MOESM4_ESM.zip › Figure 3/Fig3D/P-IRF3.tif]

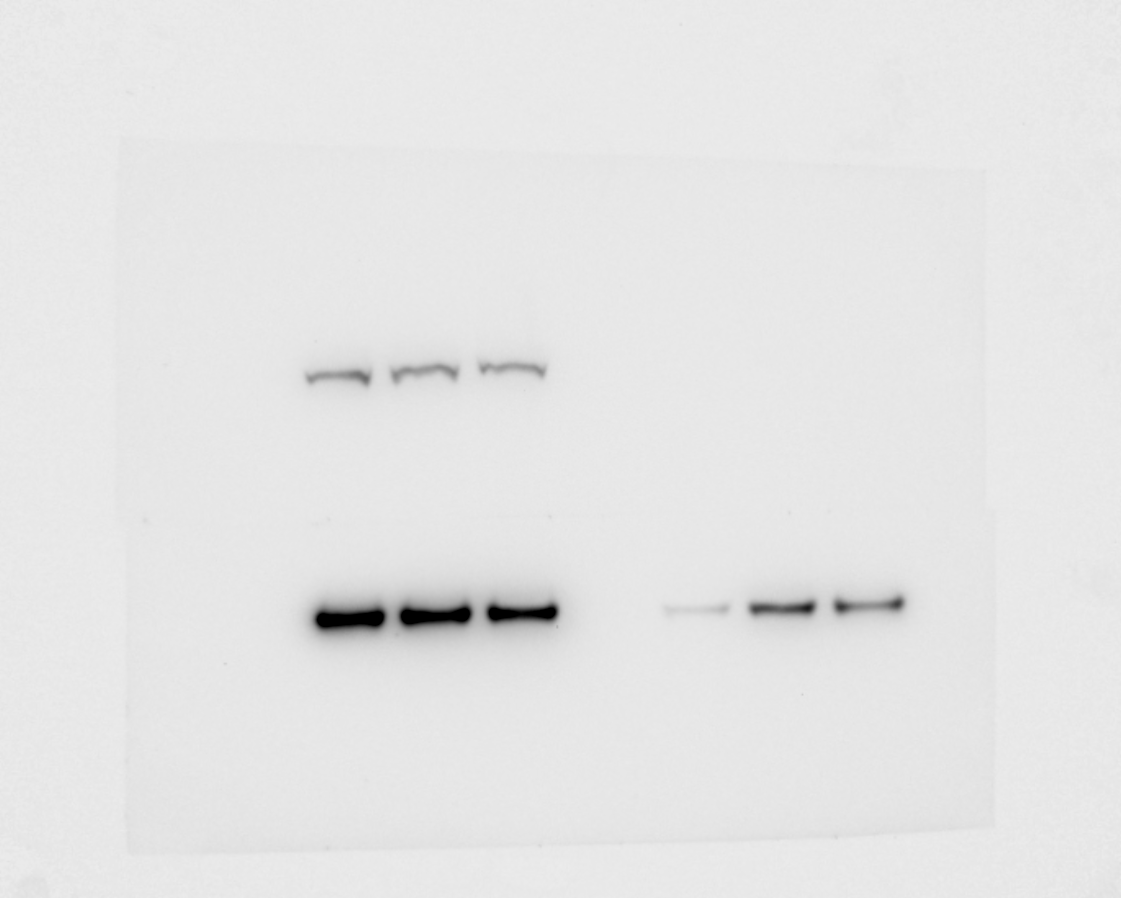

Supplement: Supplementary file 4 — Source data Fig. 3 [file 44319_2026_793_MOESM4_ESM.zip › Figure 3/Fig3D/P-STING.tif]

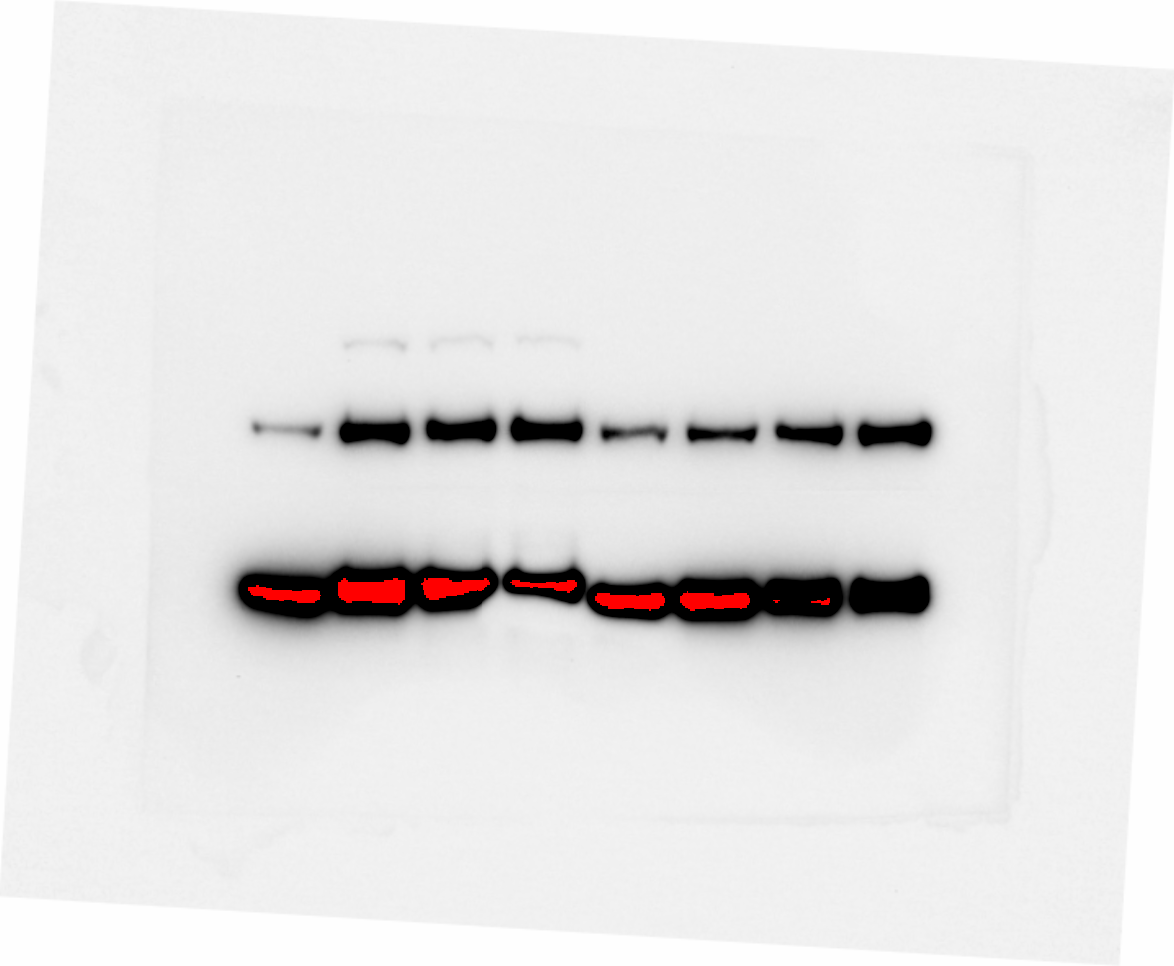

Supplement: Supplementary file 4 — Source data Fig. 3 [file 44319_2026_793_MOESM4_ESM.zip › Figure 3/Fig3D/P-p65.tif]

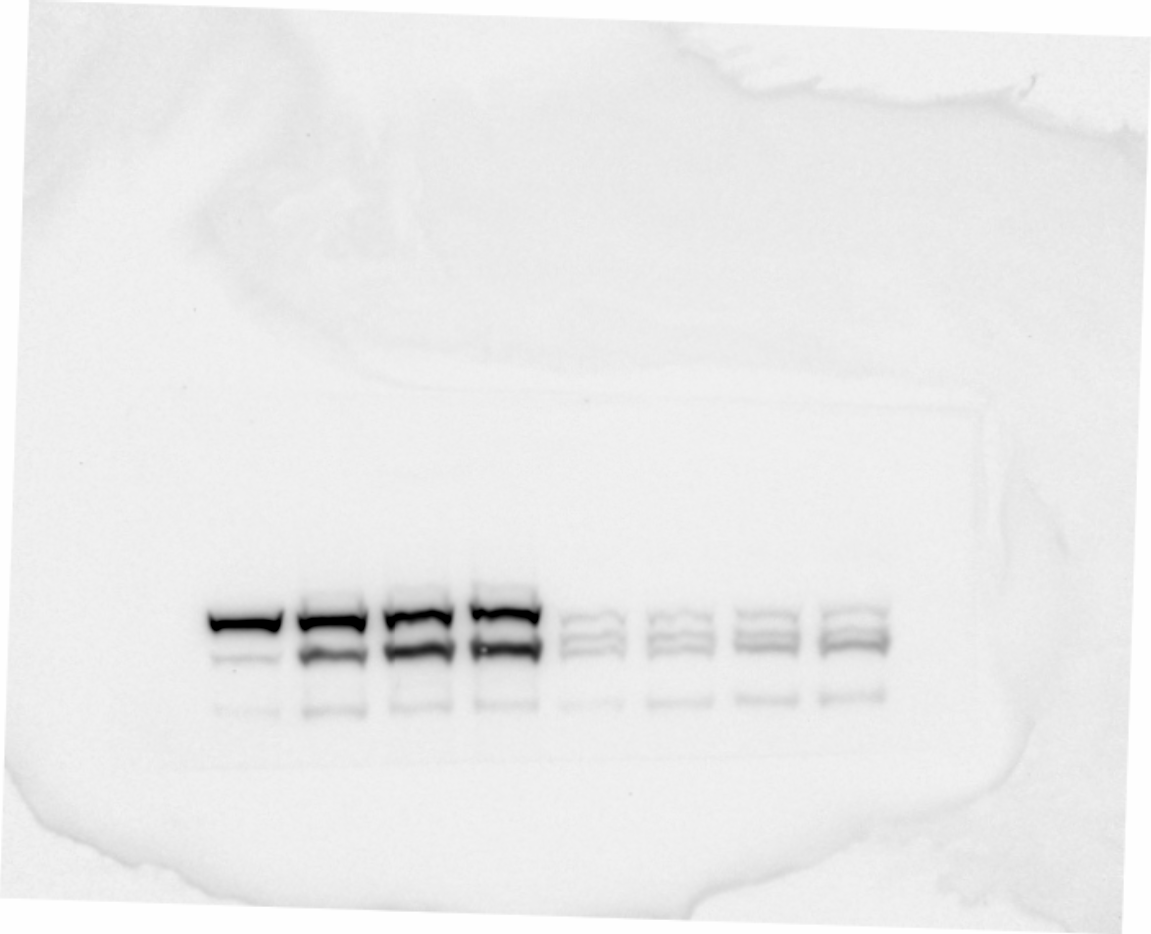

Supplement: Supplementary file 4 — Source data Fig. 3 [file 44319_2026_793_MOESM4_ESM.zip › Figure 3/Fig3D/TBK1.tif]

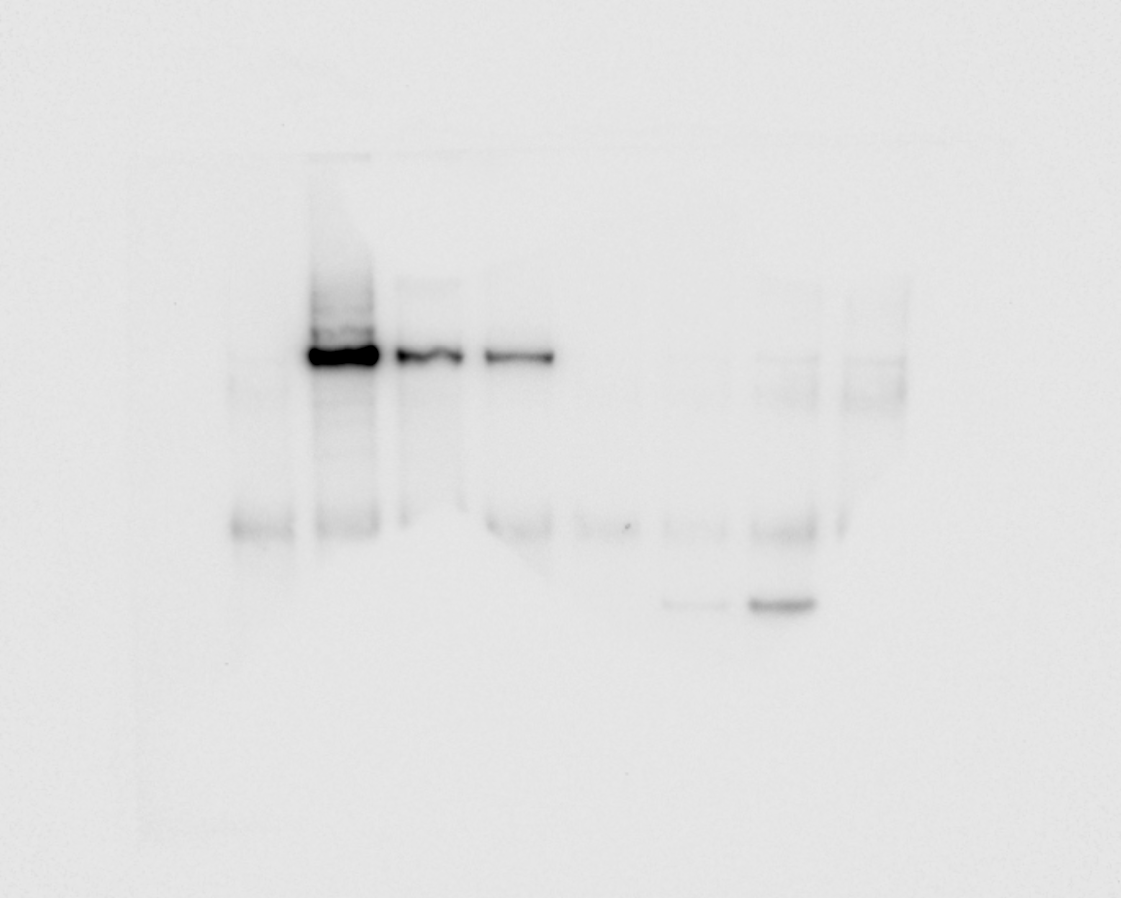

Supplement: Supplementary file 4 — Source data Fig. 3 [file 44319_2026_793_MOESM4_ESM.zip › Figure 3/Fig3D/GFP IP TBK1 IB.tif]

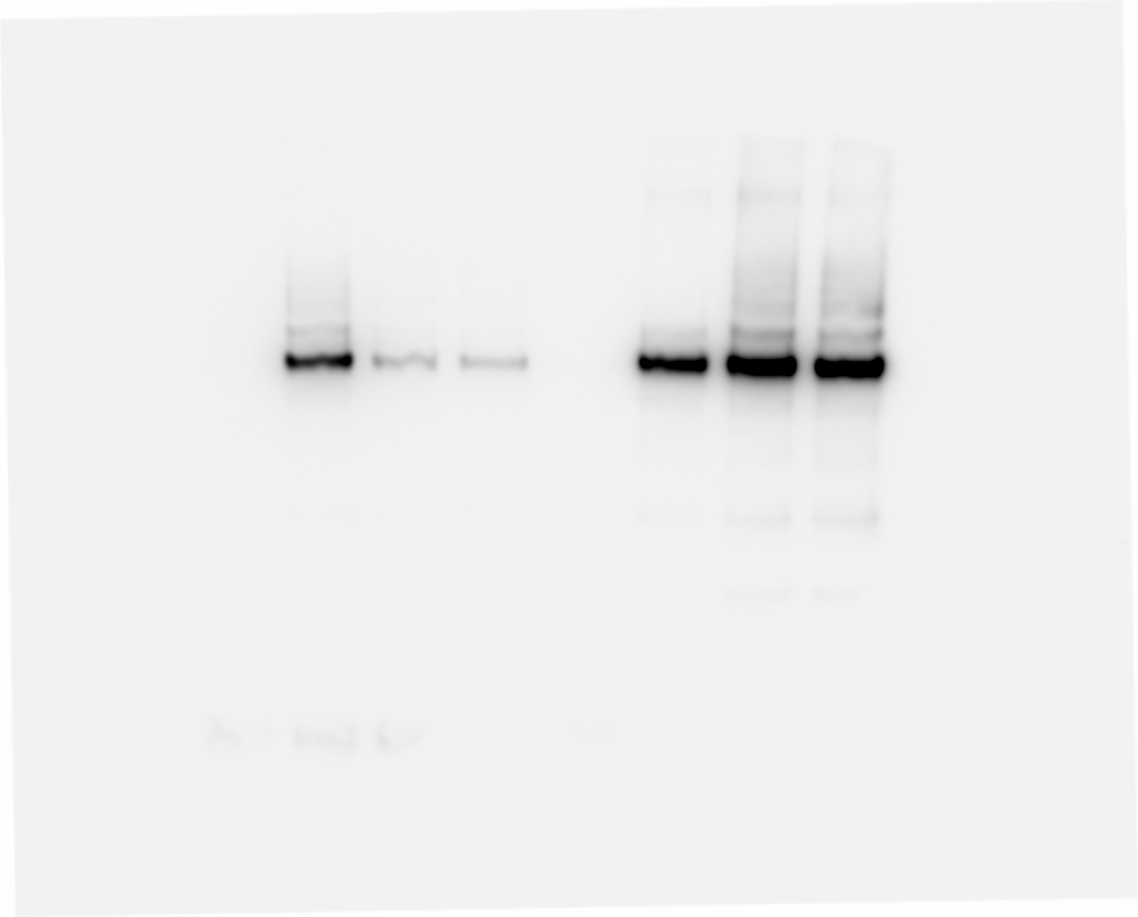

Supplement: Supplementary file 4 — Source data Fig. 3 [file 44319_2026_793_MOESM4_ESM.zip › Figure 3/Fig3D/GFP IP IKKe IB.tif]

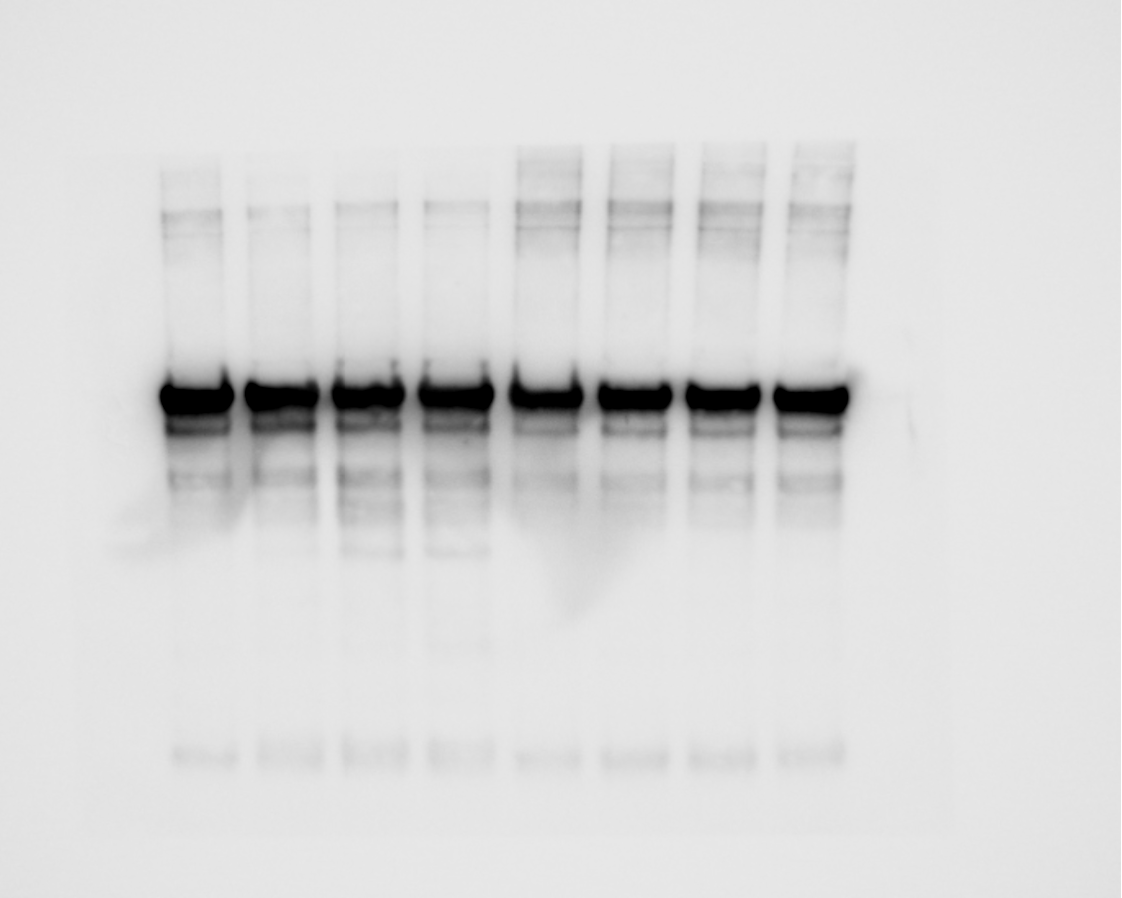

Supplement: Supplementary file 4 — Source data Fig. 3 [file 44319_2026_793_MOESM4_ESM.zip › Figure 3/Fig3D/GFP IP IRF3.tif]

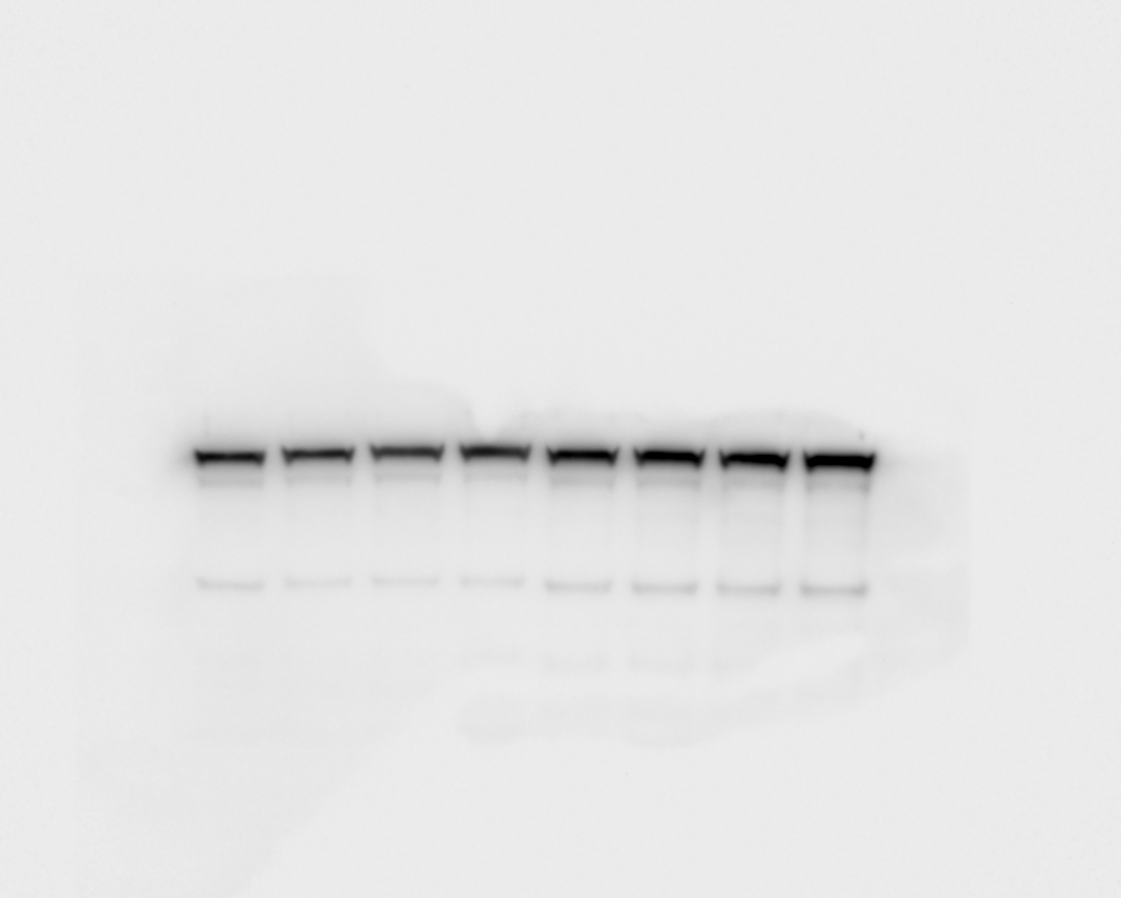

Supplement: Supplementary file 4 — Source data Fig. 3 [file 44319_2026_793_MOESM4_ESM.zip › Figure 3/Fig3D/IRF3.tif]

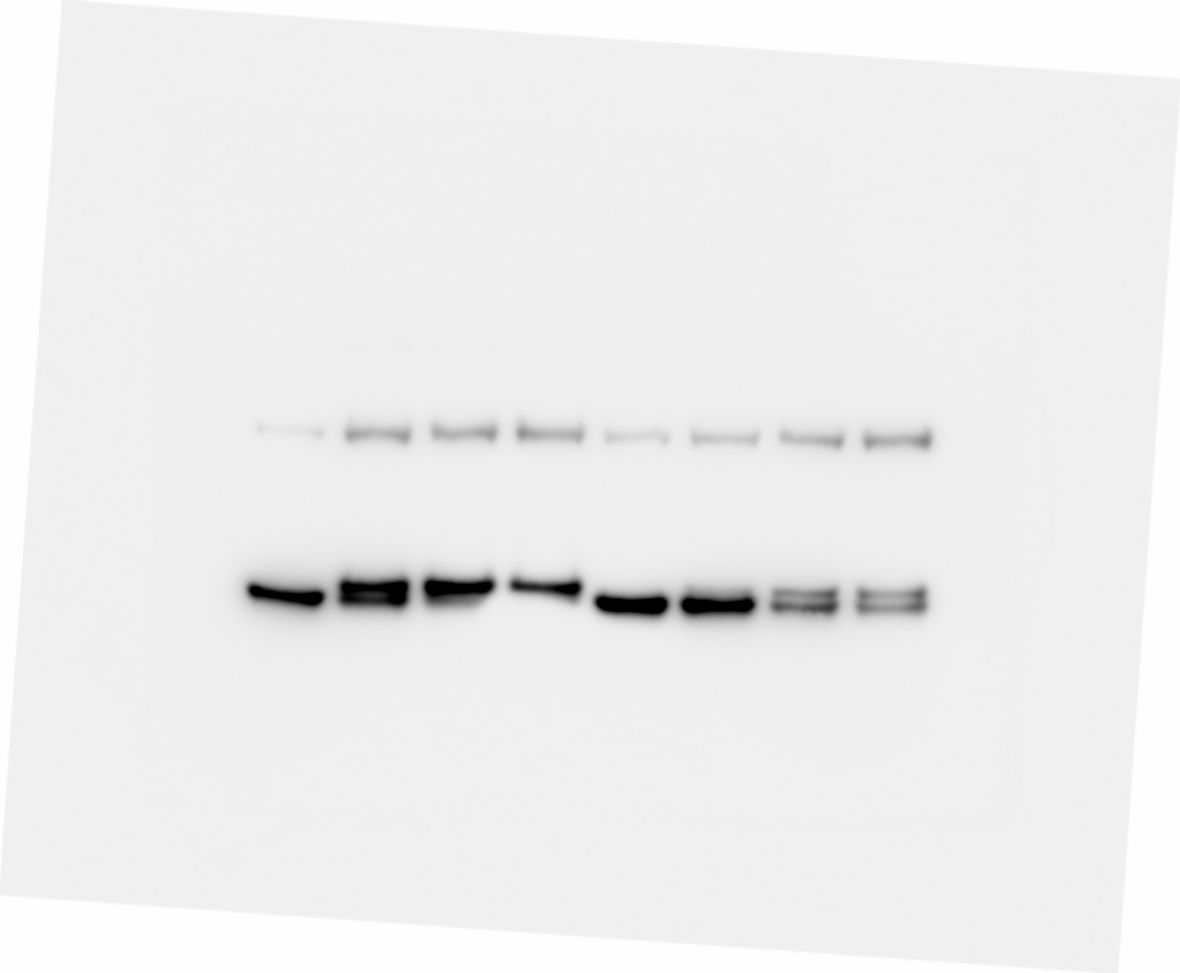

Supplement: Supplementary file 4 — Source data Fig. 3 [file 44319_2026_793_MOESM4_ESM.zip › Figure 3/Fig3D/STING.tif]

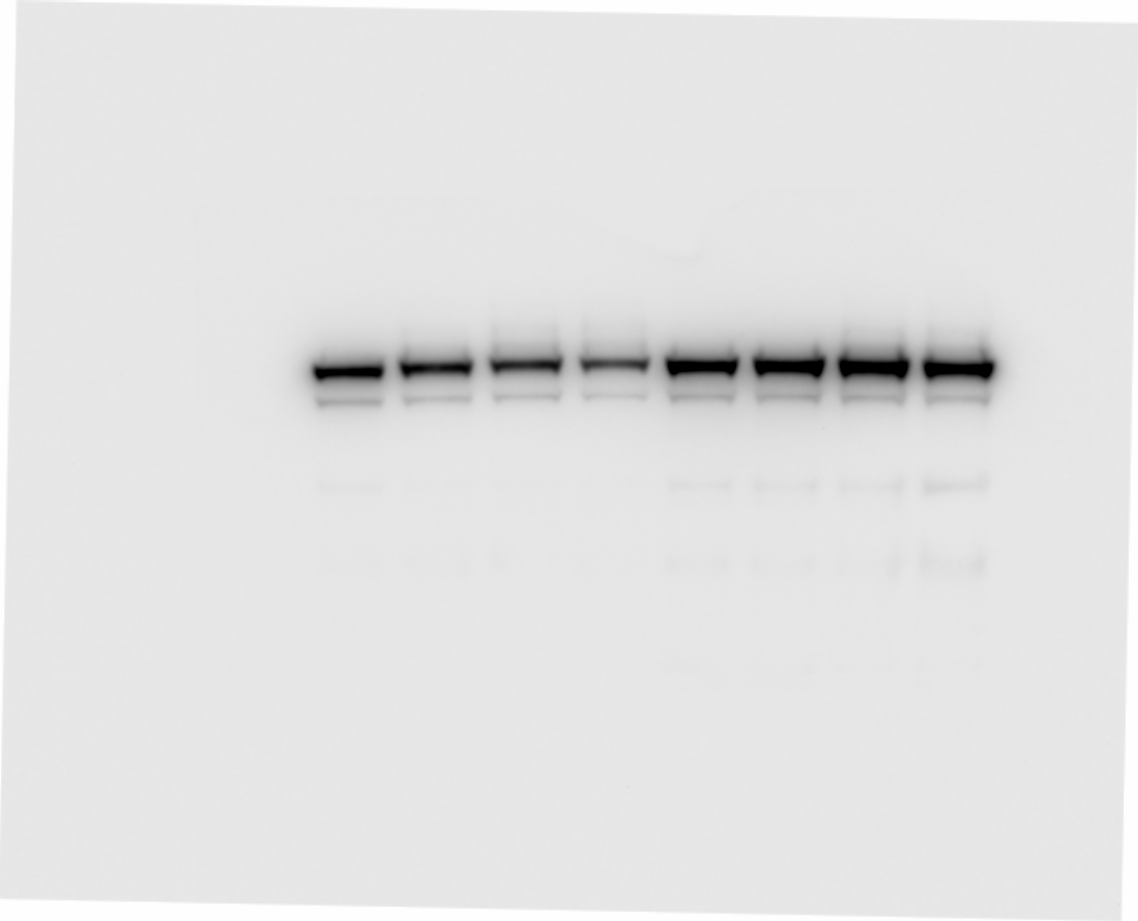

Supplement: Supplementary file 4 — Source data Fig. 3 [file 44319_2026_793_MOESM4_ESM.zip › Figure 3/Fig3D/IKKe.tif]

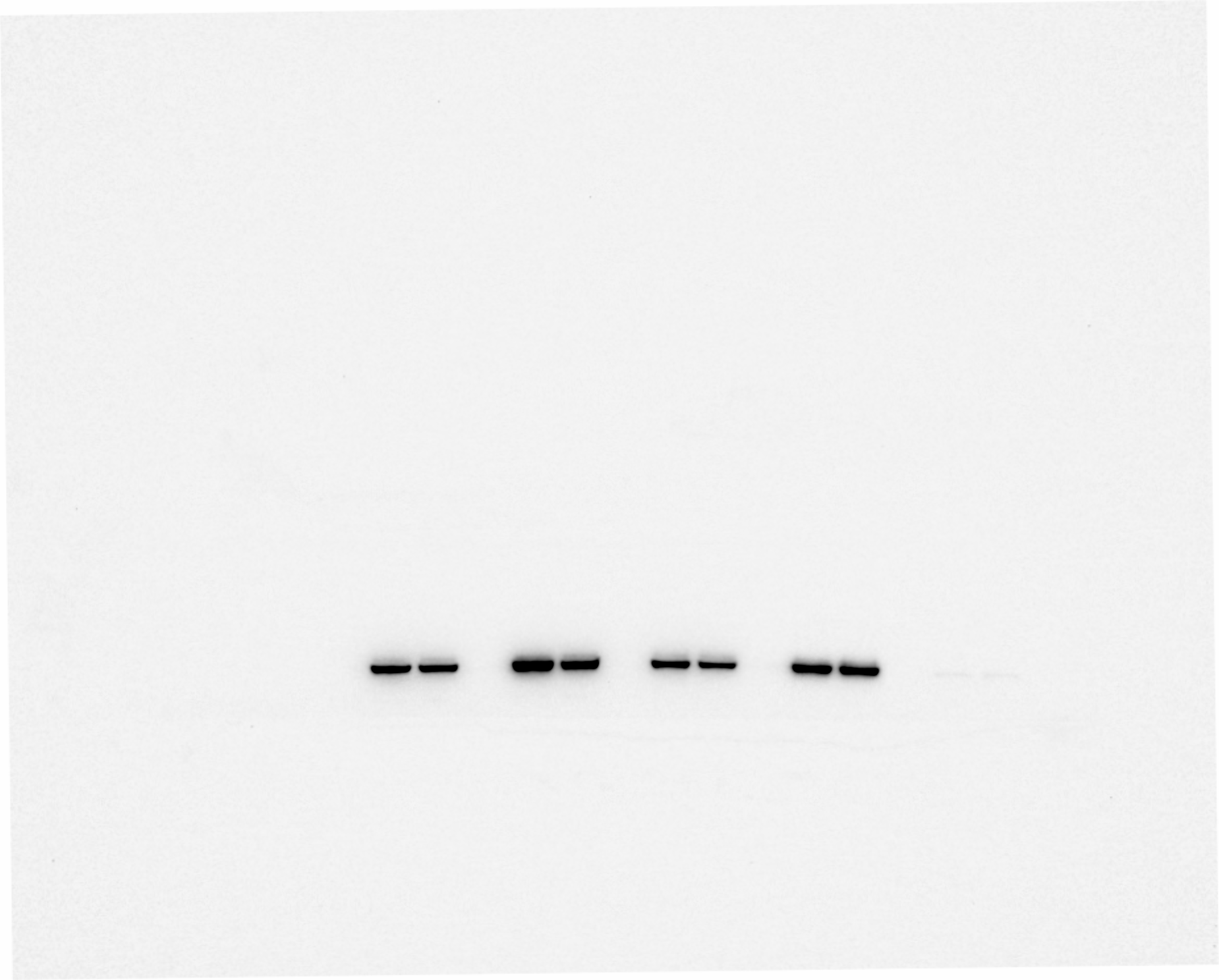

Supplement: Supplementary file 5 — Source data Fig. 4 [file 44319_2026_793_MOESM5_ESM.zip › Figure 4/Fig4A/P-TBK1.tif]

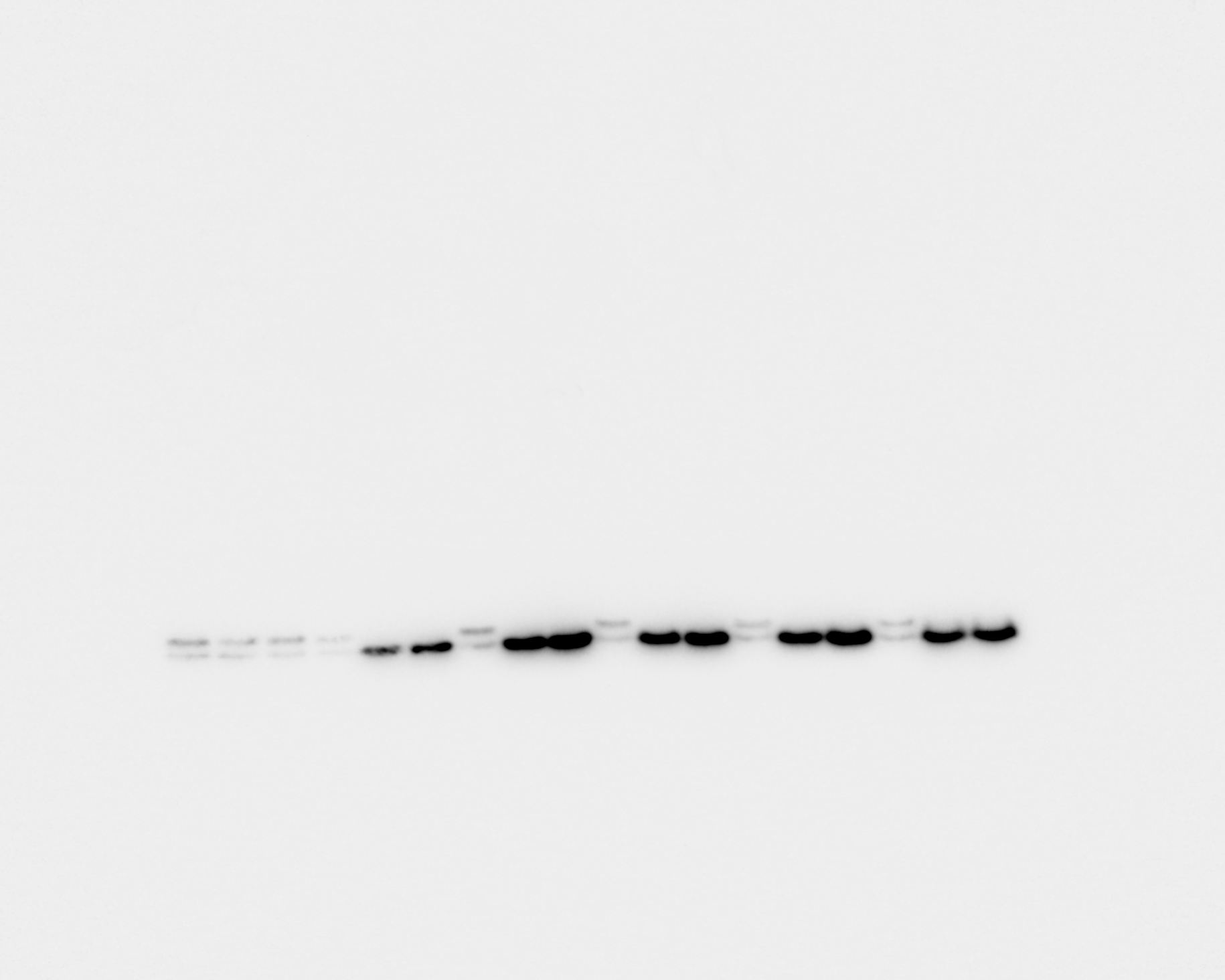

Supplement: Supplementary file 5 — Source data Fig. 4 [file 44319_2026_793_MOESM5_ESM.zip › Figure 4/Fig4A/LC3B.tif]

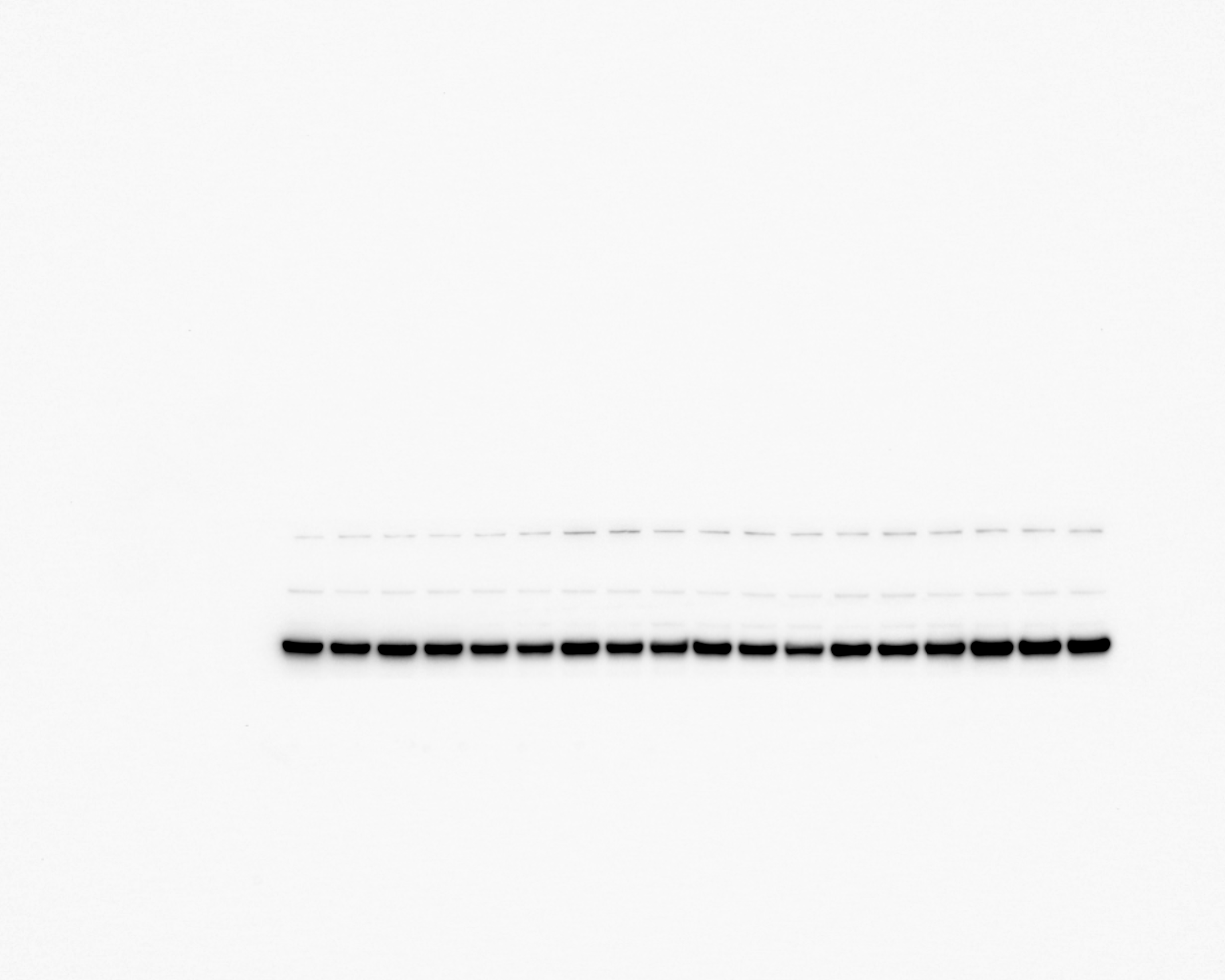

Supplement: Supplementary file 5 — Source data Fig. 4 [file 44319_2026_793_MOESM5_ESM.zip › Figure 4/Fig4A/P65.tif]

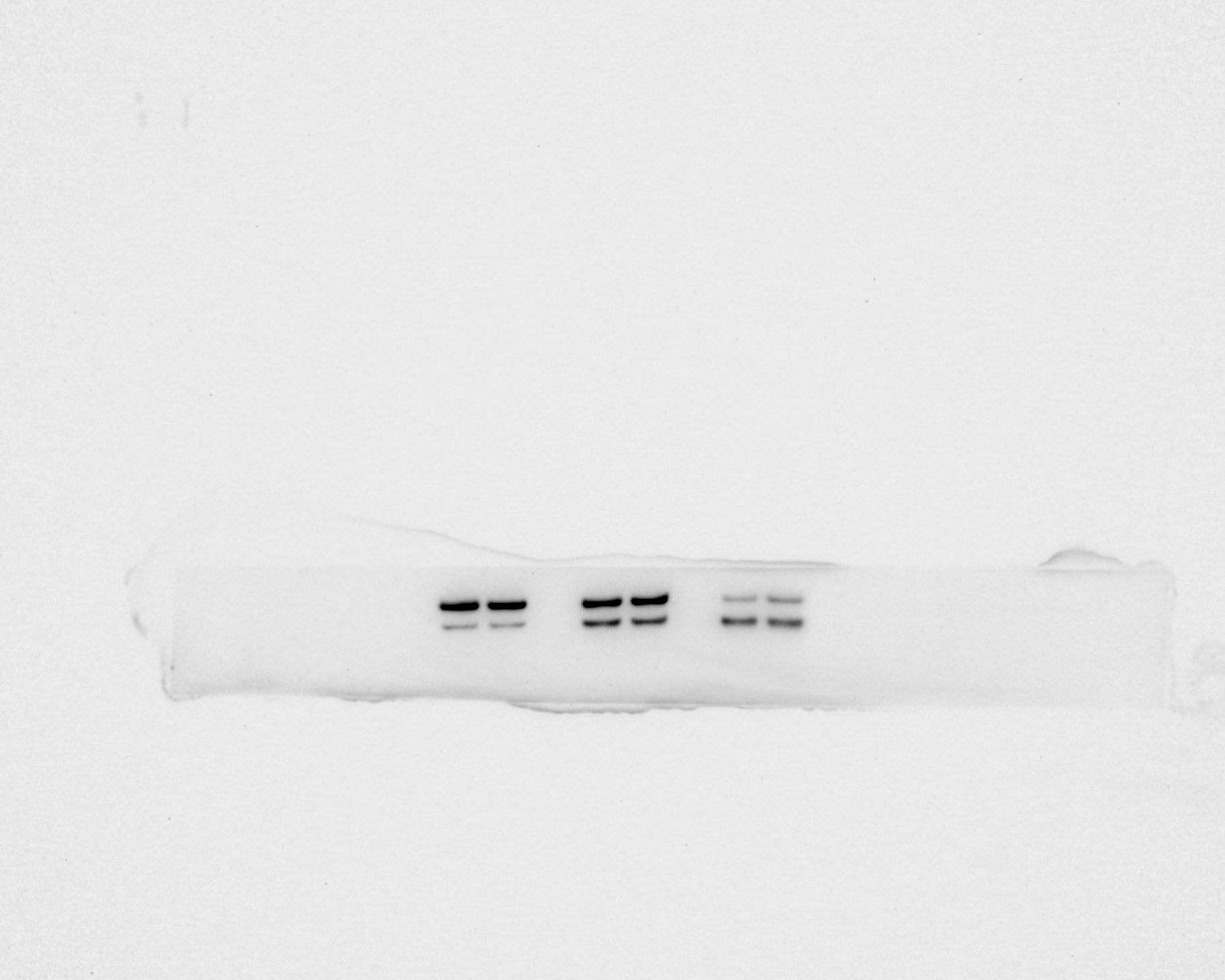

Supplement: Supplementary file 5 — Source data Fig. 4 [file 44319_2026_793_MOESM5_ESM.zip › Figure 4/Fig4A/P-IRF3.tif]

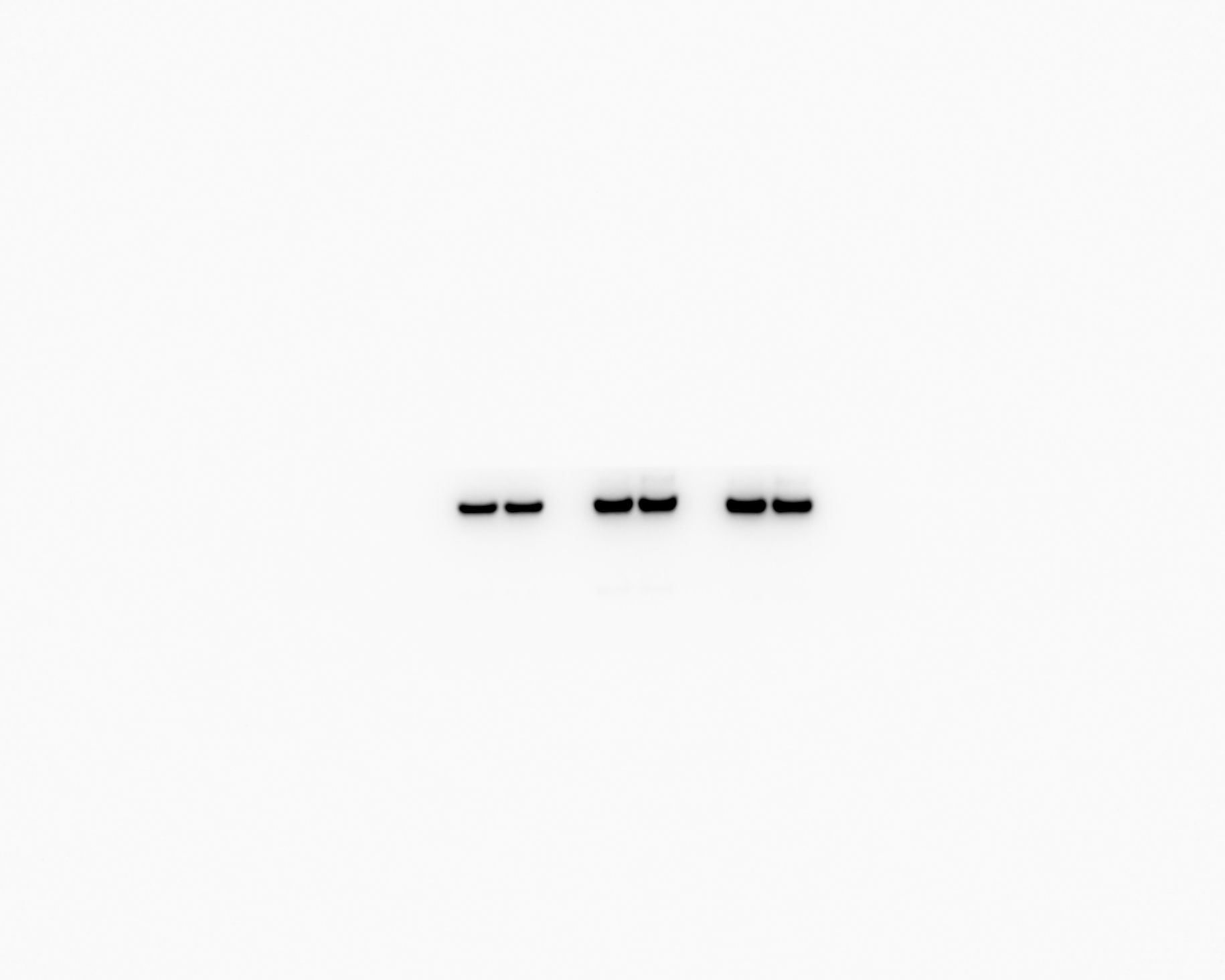

Supplement: Supplementary file 5 — Source data Fig. 4 [file 44319_2026_793_MOESM5_ESM.zip › Figure 4/Fig4A/P-STING.tif]

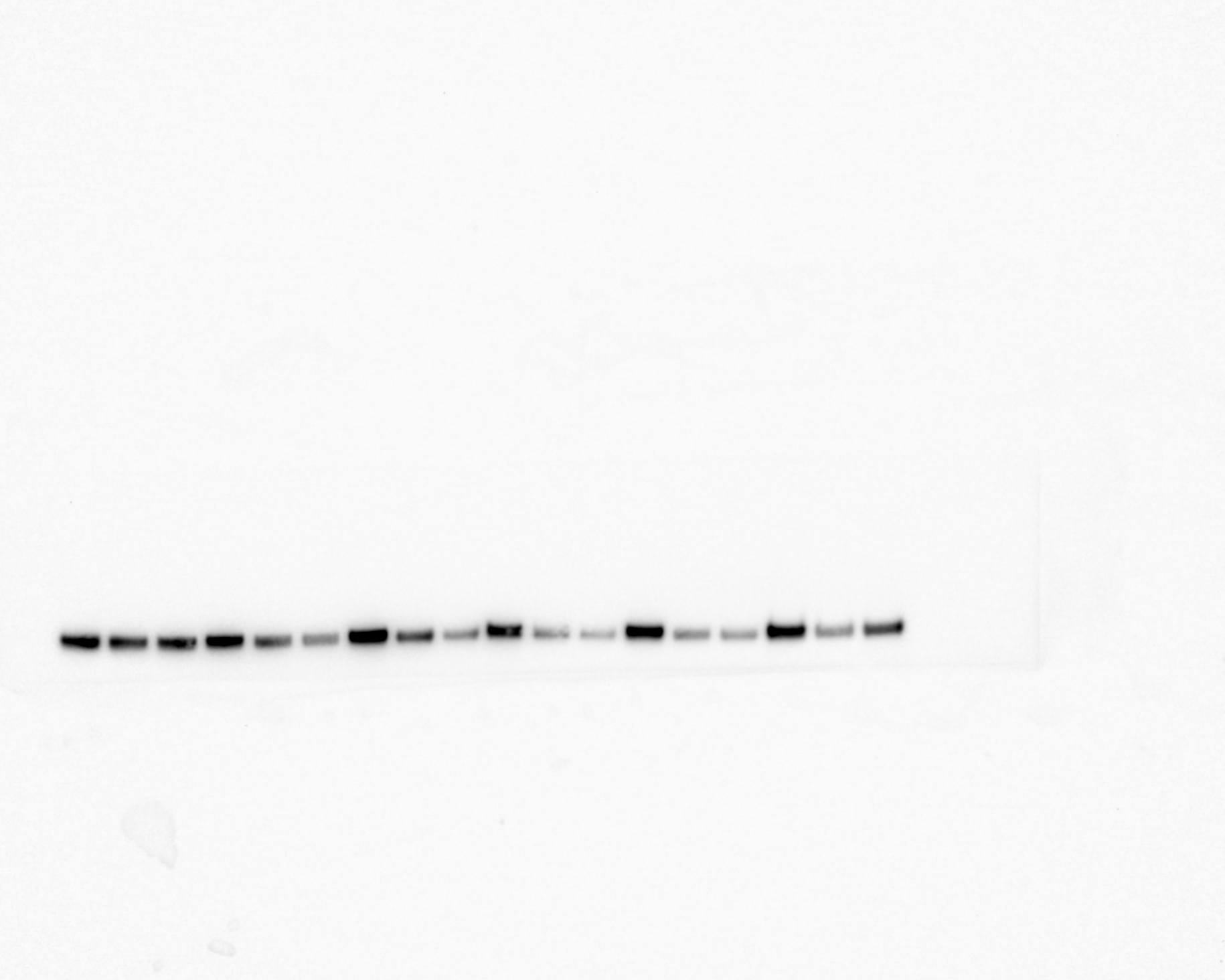

Supplement: Supplementary file 5 — Source data Fig. 4 [file 44319_2026_793_MOESM5_ESM.zip › Figure 4/Fig4A/P-TEFB.tif]

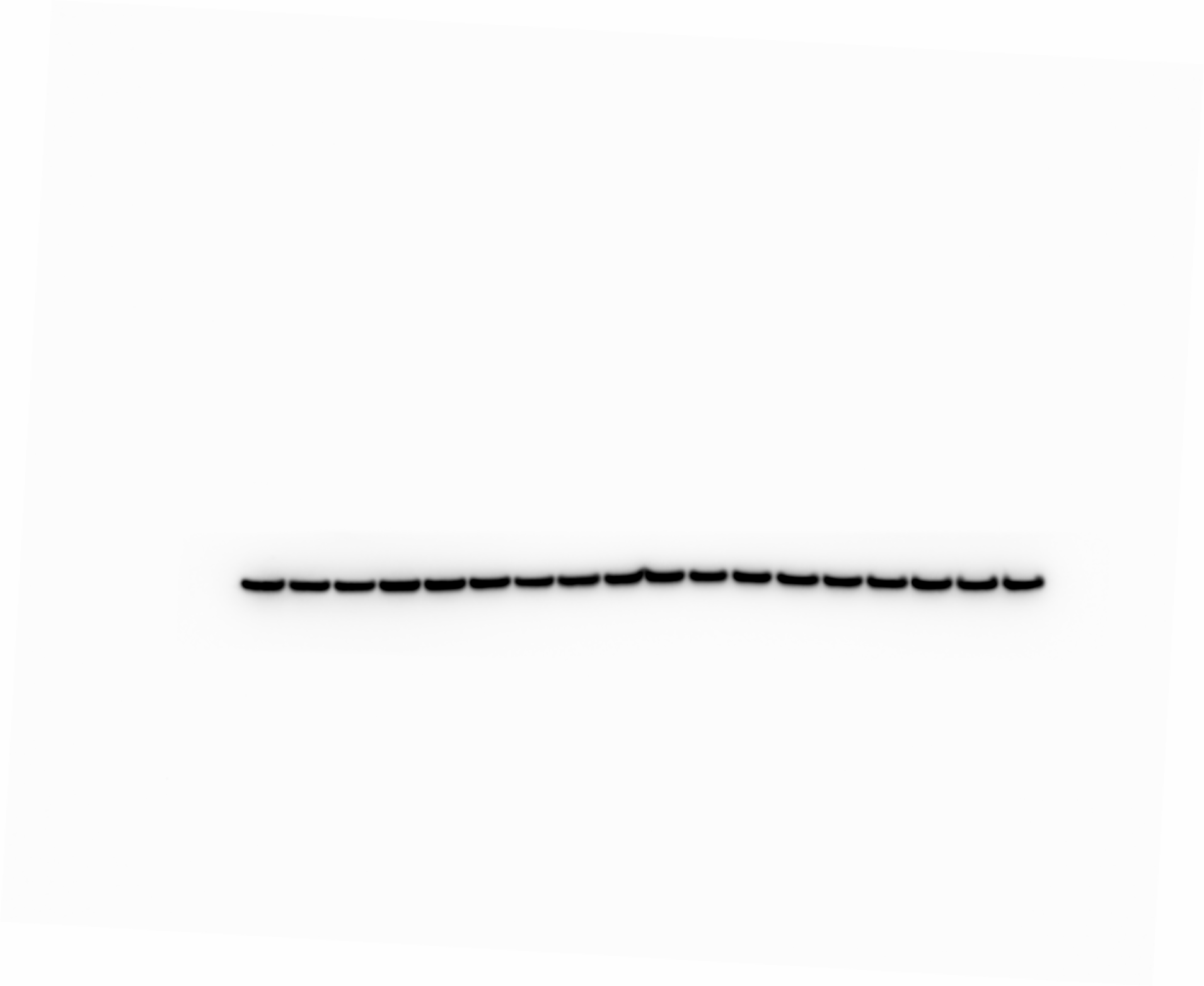

Supplement: Supplementary file 5 — Source data Fig. 4 [file 44319_2026_793_MOESM5_ESM.zip › Figure 4/Fig4A/ACTIN.tif]

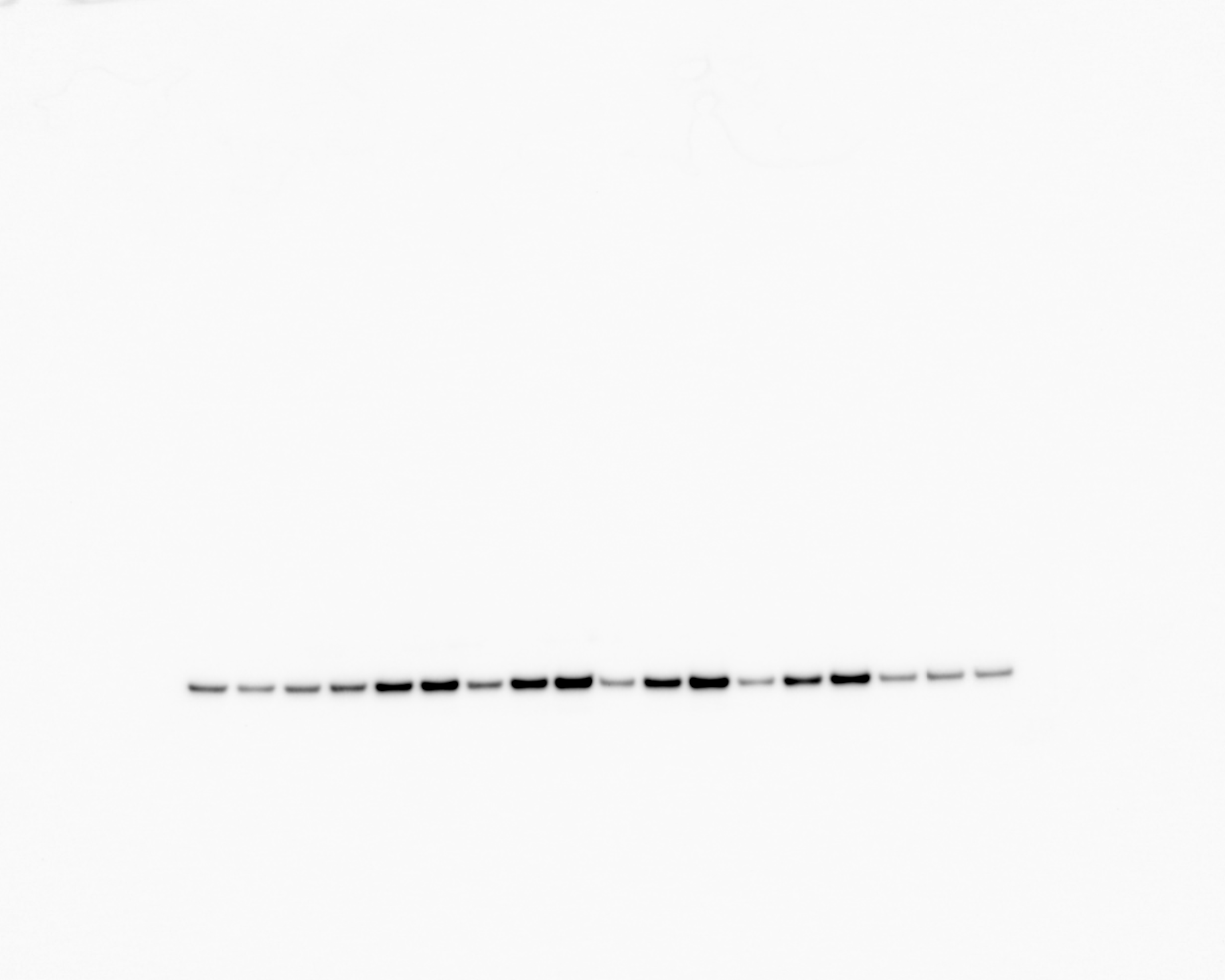

Supplement: Supplementary file 5 — Source data Fig. 4 [file 44319_2026_793_MOESM5_ESM.zip › Figure 4/Fig4A/P-p65.tif]

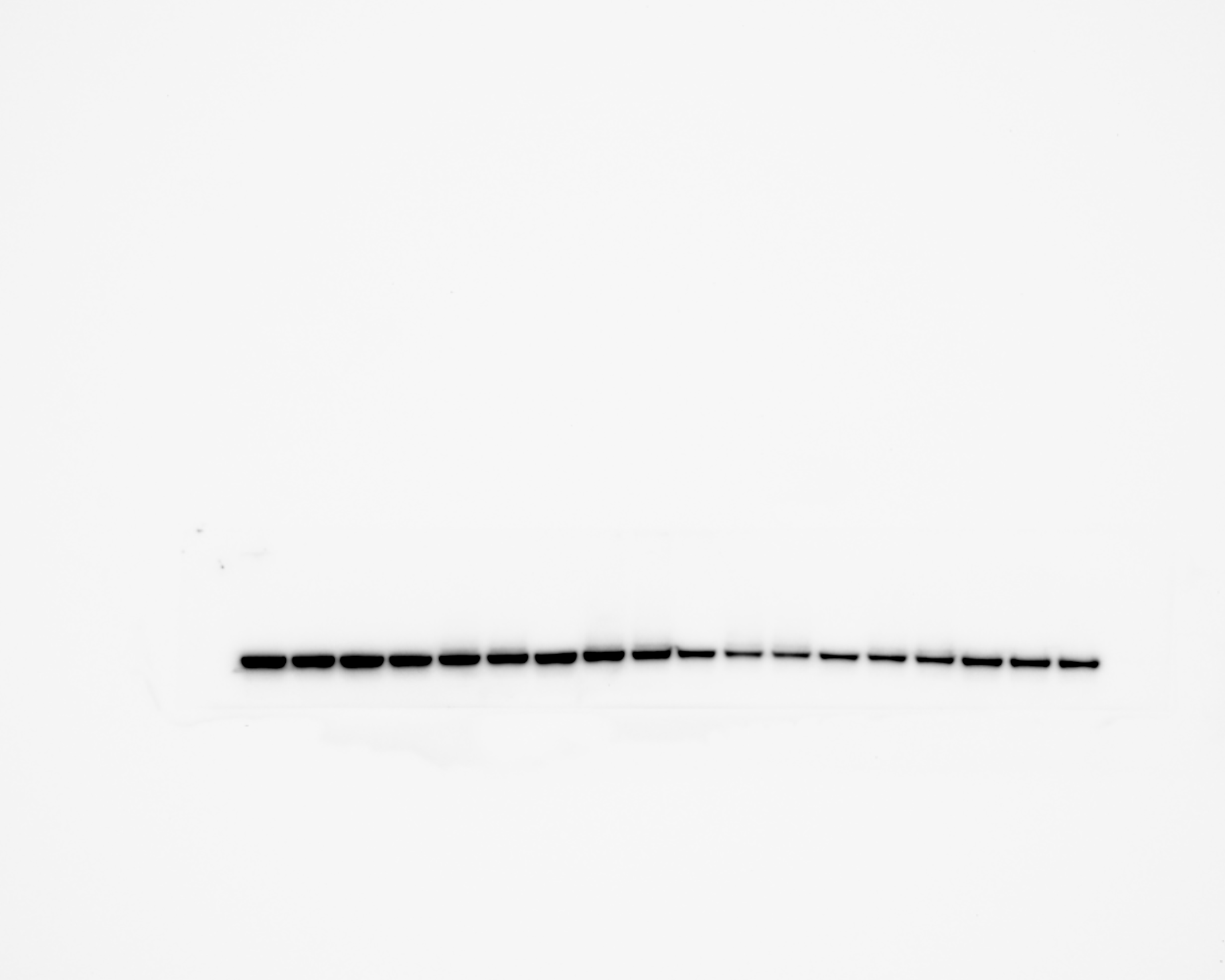

Supplement: Supplementary file 5 — Source data Fig. 4 [file 44319_2026_793_MOESM5_ESM.zip › Figure 4/Fig4A/TBK1.tif]

Figure 4A\_uncropped blots

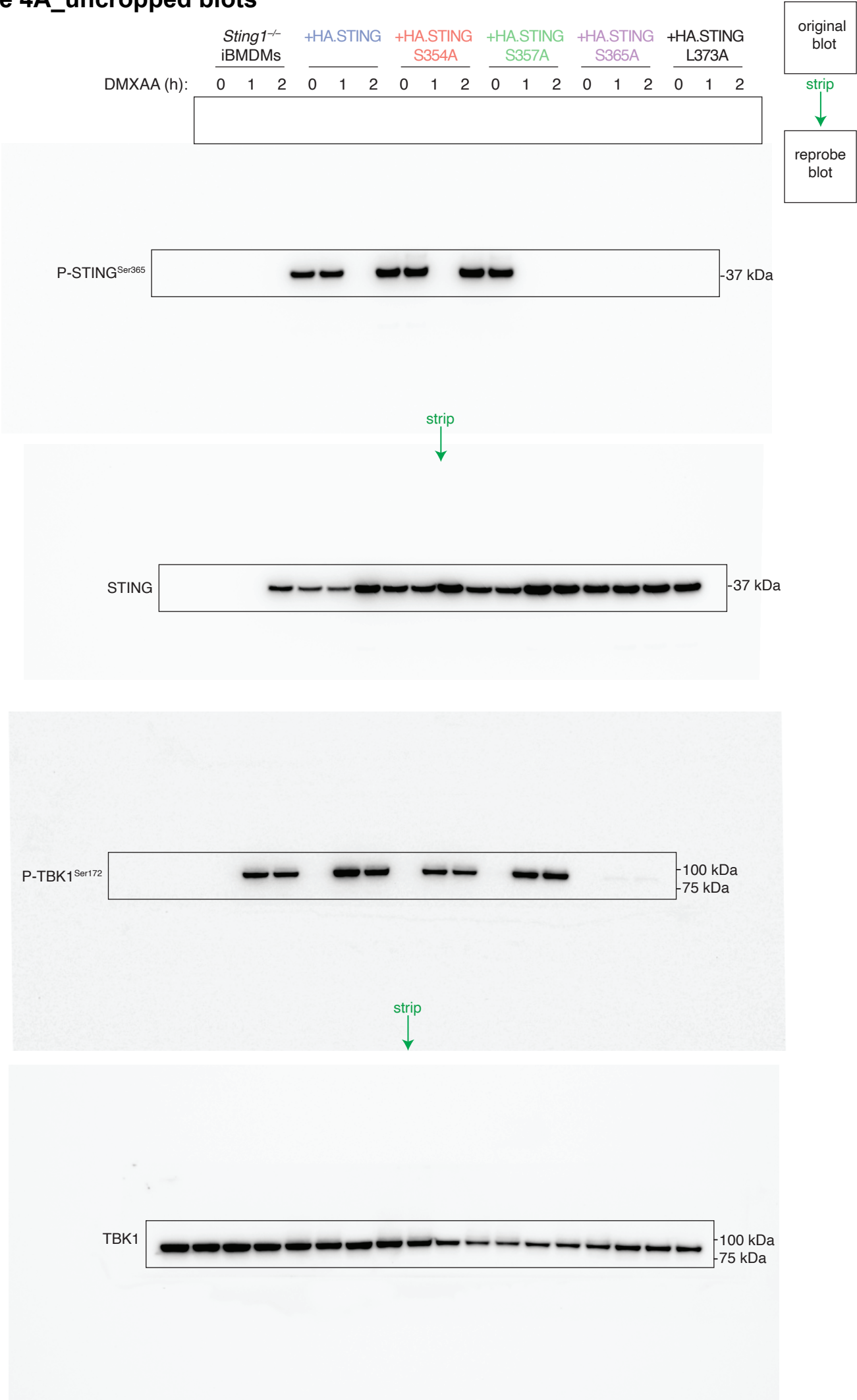

Figure 4A\_uncropped blots

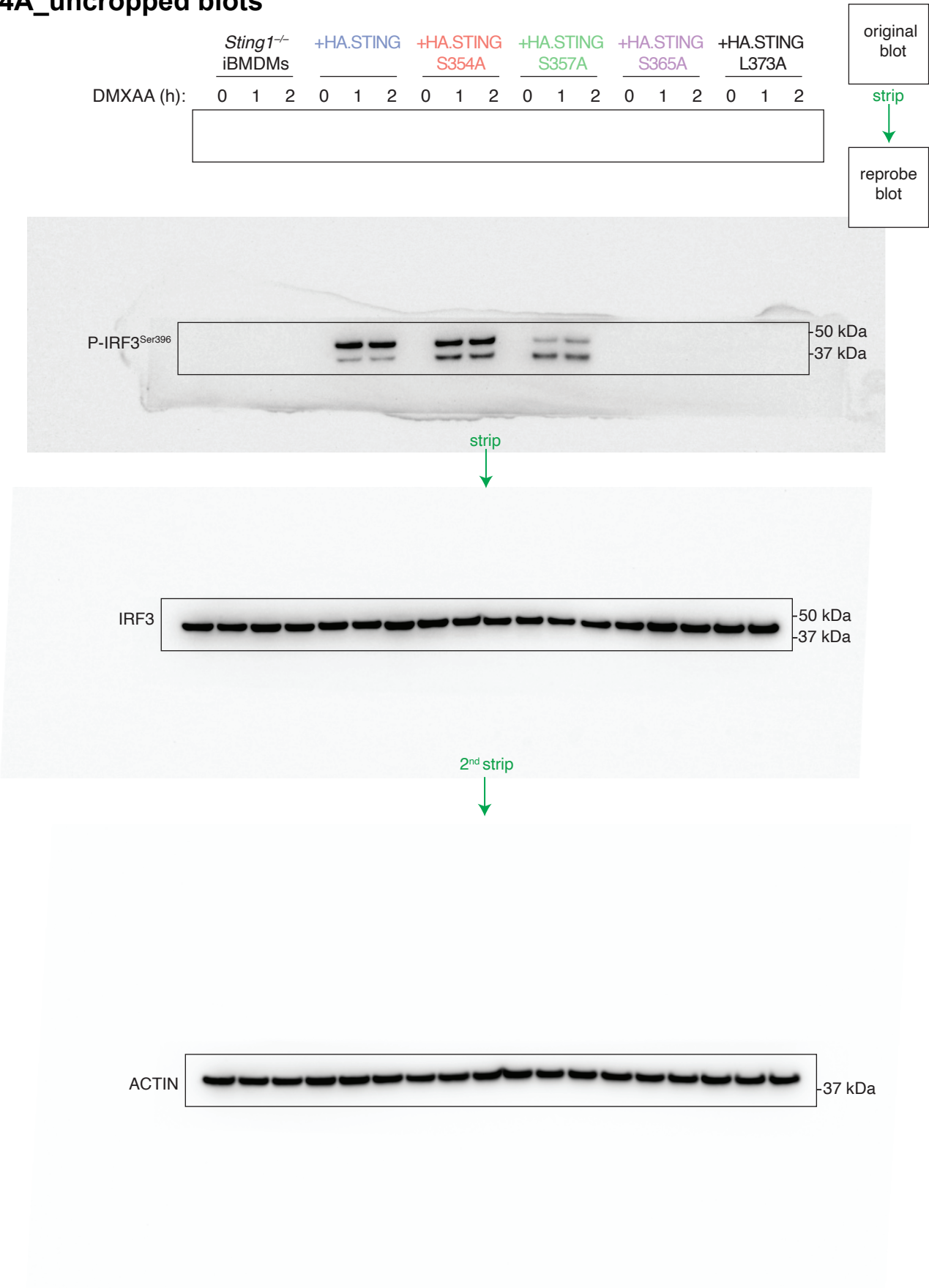

Figure 4A\_uncropped blots

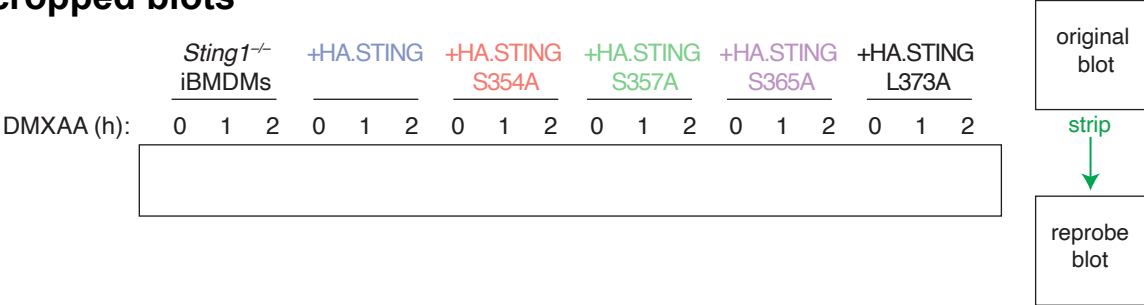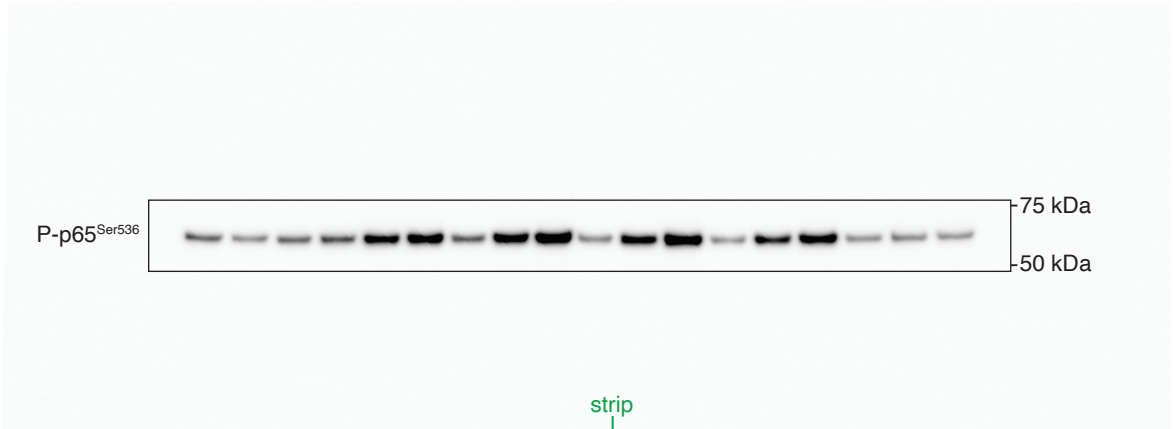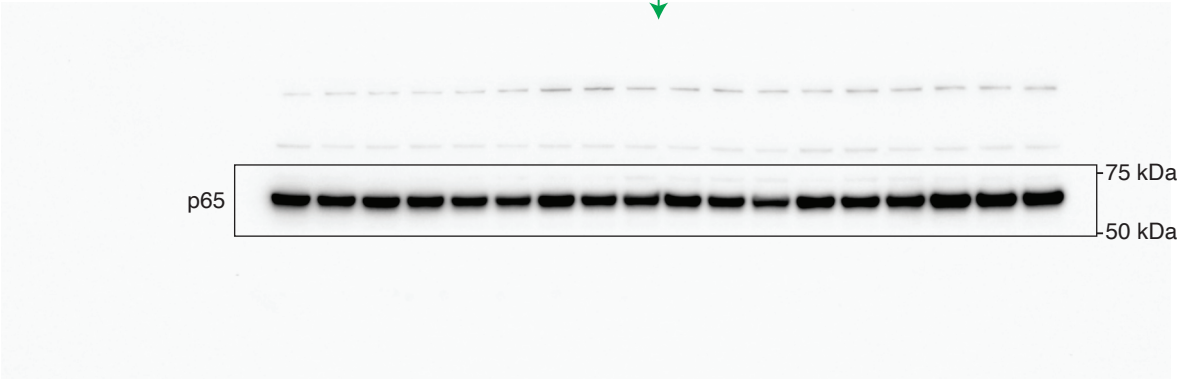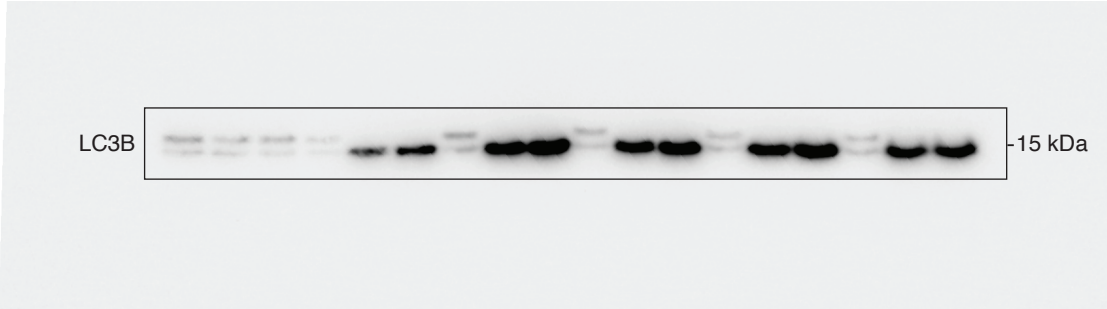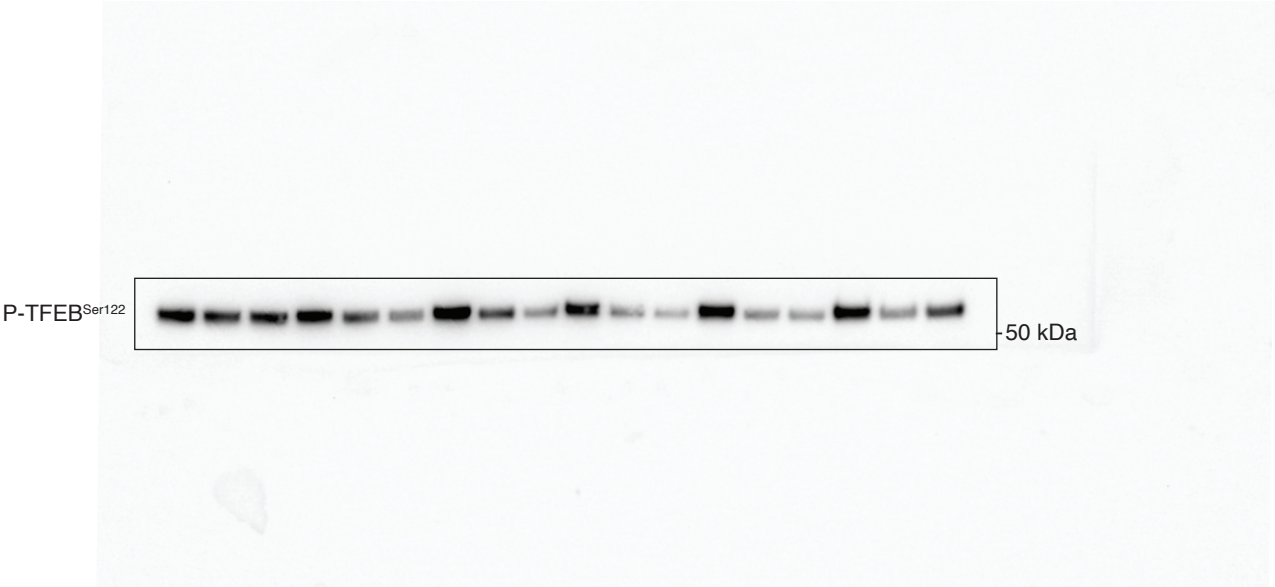

Supplement: Supplementary file 5 — Source data Fig. 4 [file 44319_2026_793_MOESM5_ESM.zip › Figure 4/Fig4A/Fig4A _uncropped blots.pdf]

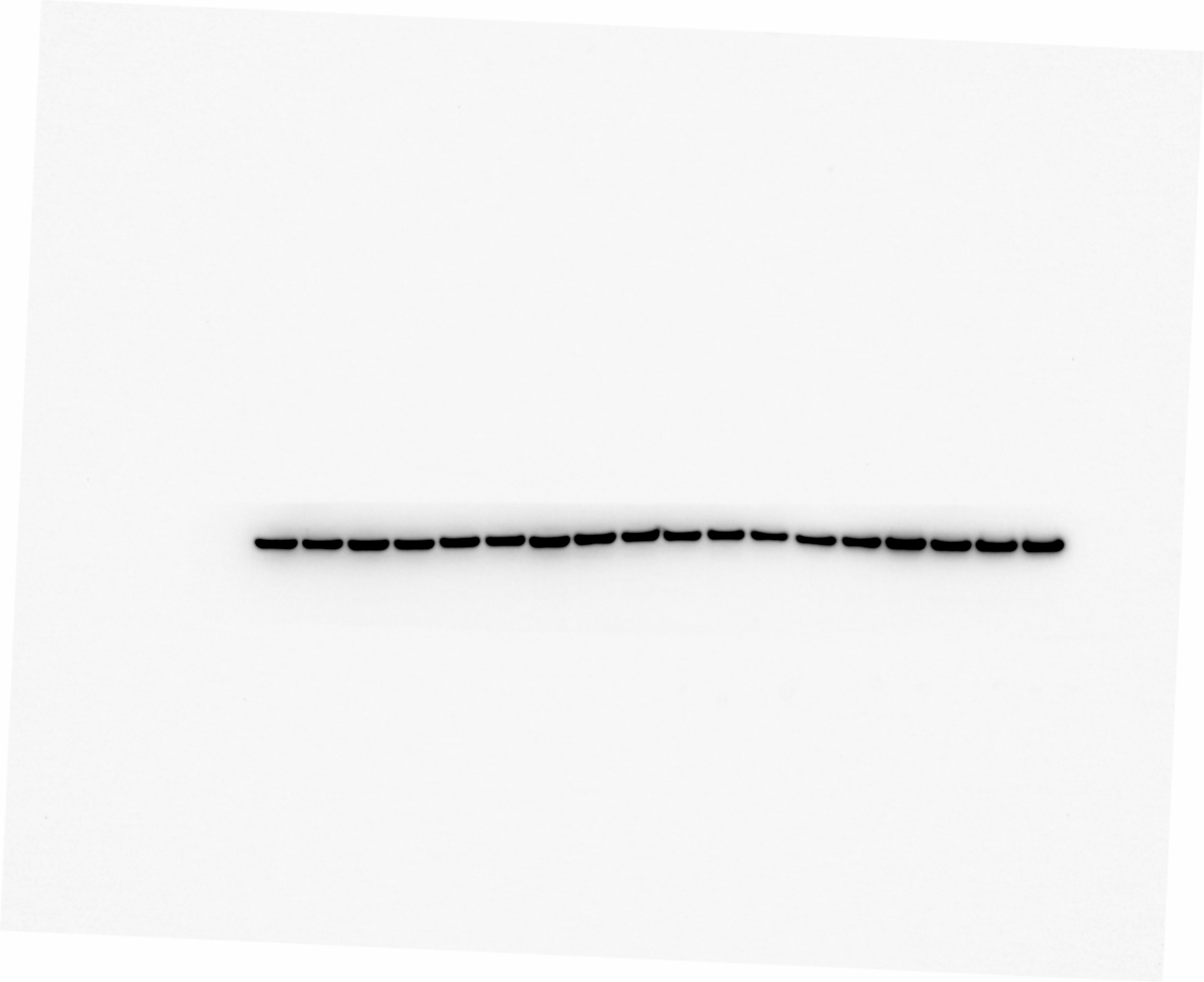

Supplement: Supplementary file 5 — Source data Fig. 4 [file 44319_2026_793_MOESM5_ESM.zip › Figure 4/Fig4A/IRF3.tif]

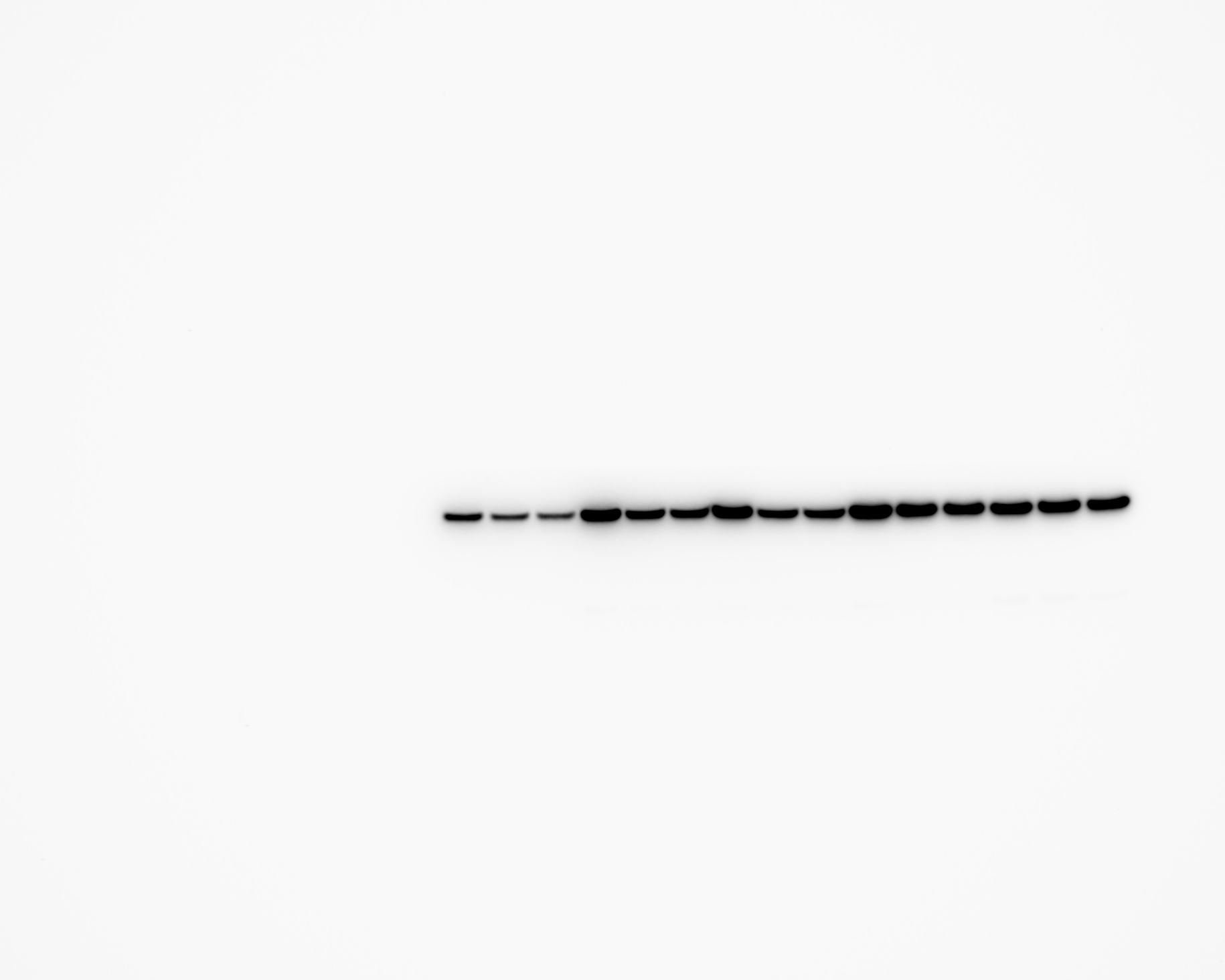

Supplement: Supplementary file 5 — Source data Fig. 4 [file 44319_2026_793_MOESM5_ESM.zip › Figure 4/Fig4A/STING.tif]

Figure 4D\_uncropped blots

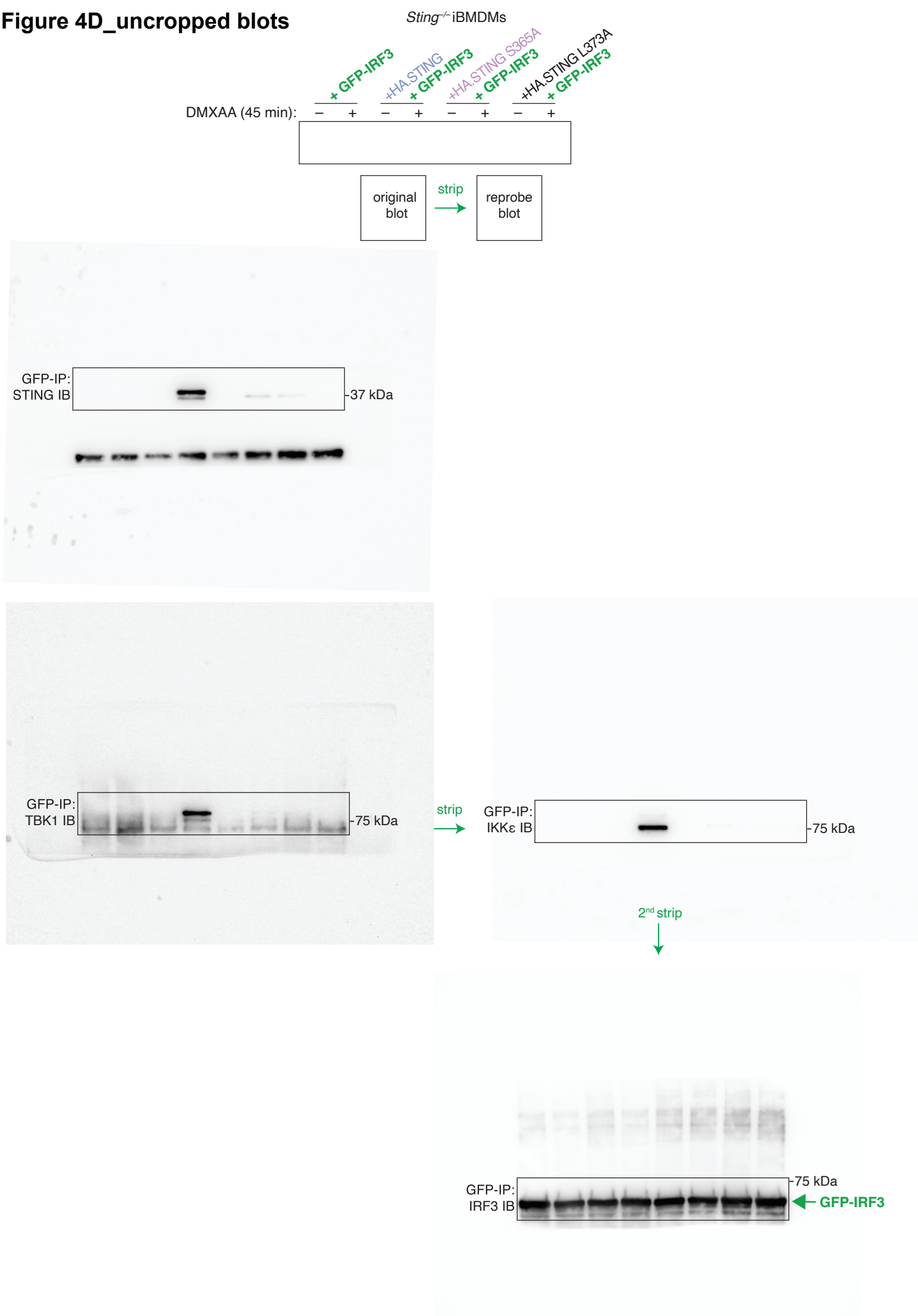

Supplement: Supplementary file 5 — Source data Fig. 4 [file 44319_2026_793_MOESM5_ESM.zip › Figure 4/Fig4D/Fig4D_uncropped blots.pdf]

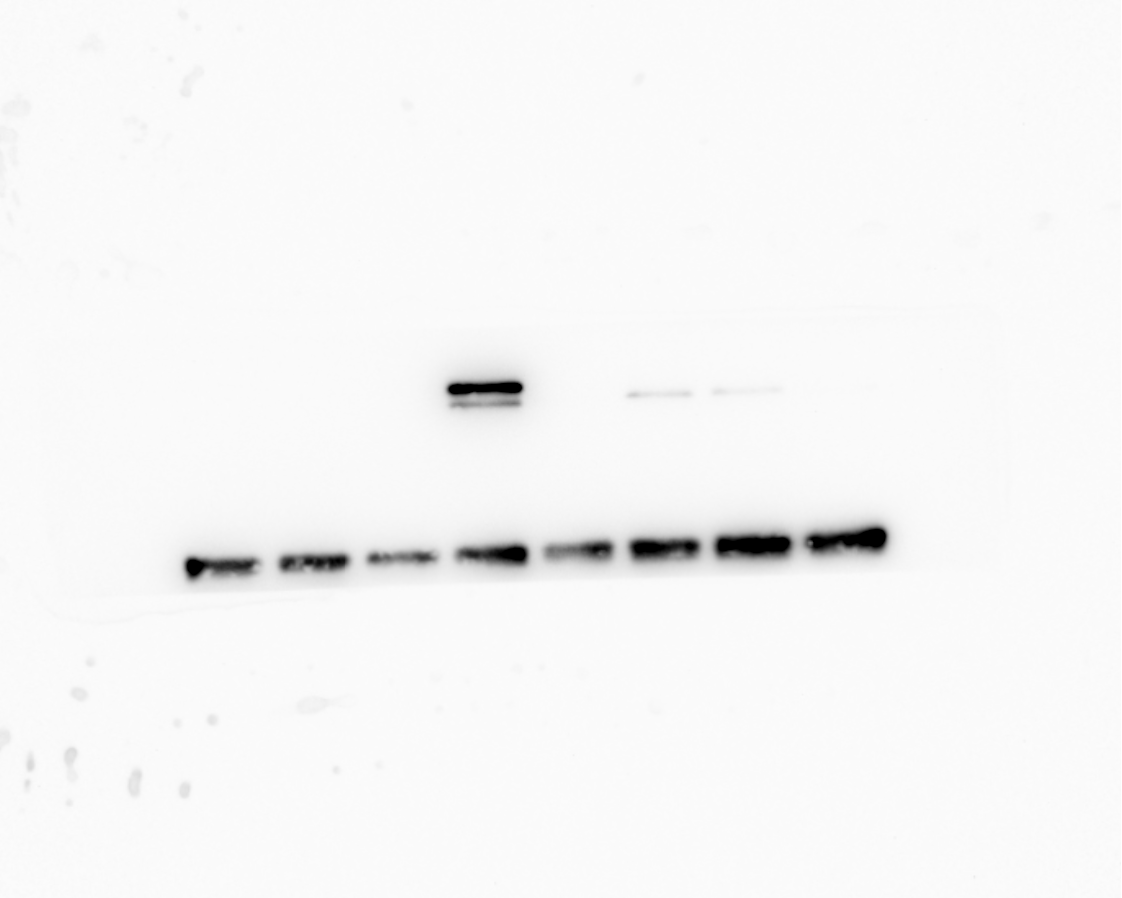

Supplement: Supplementary file 5 — Source data Fig. 4 [file 44319_2026_793_MOESM5_ESM.zip › Figure 4/Fig4D/GFP IP STING IB.tif]

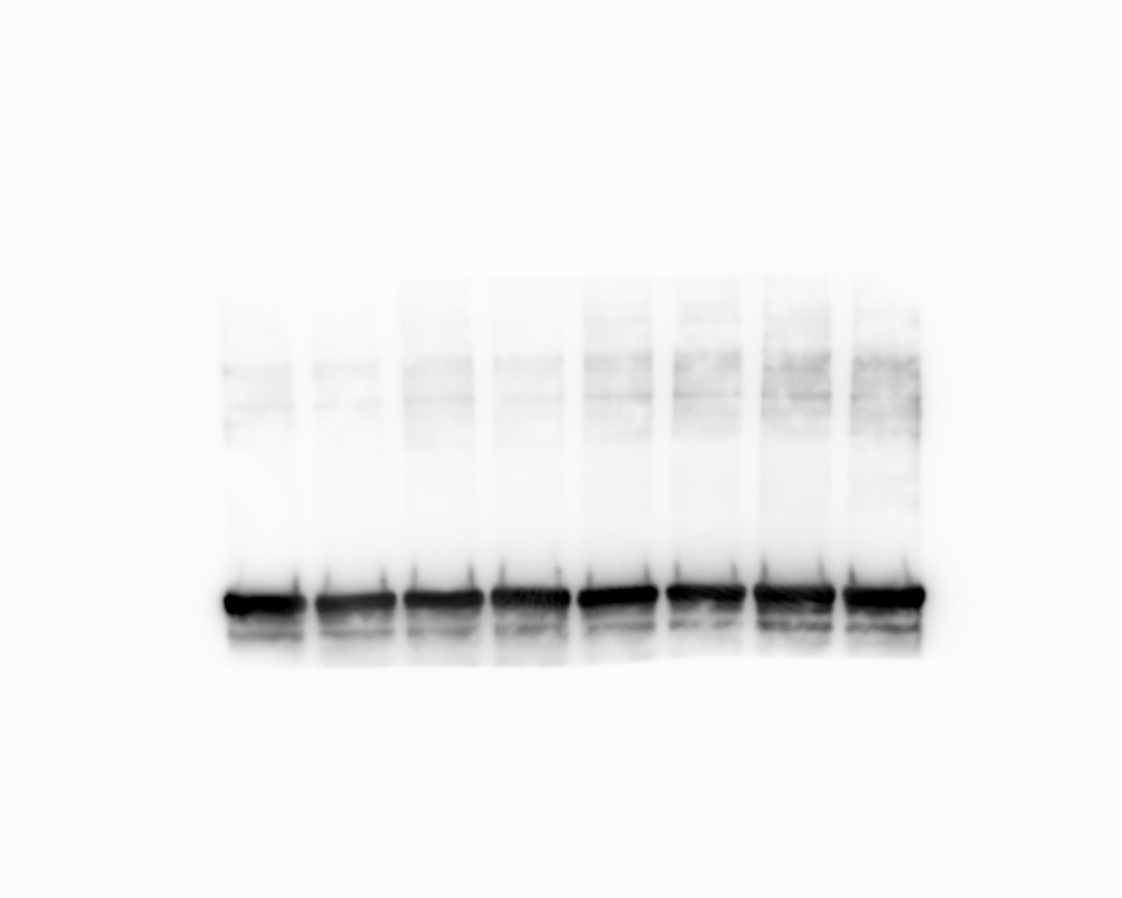

Supplement: Supplementary file 5 — Source data Fig. 4 [file 44319_2026_793_MOESM5_ESM.zip › Figure 4/Fig4D/GFP IP IRF3 IB.tif]

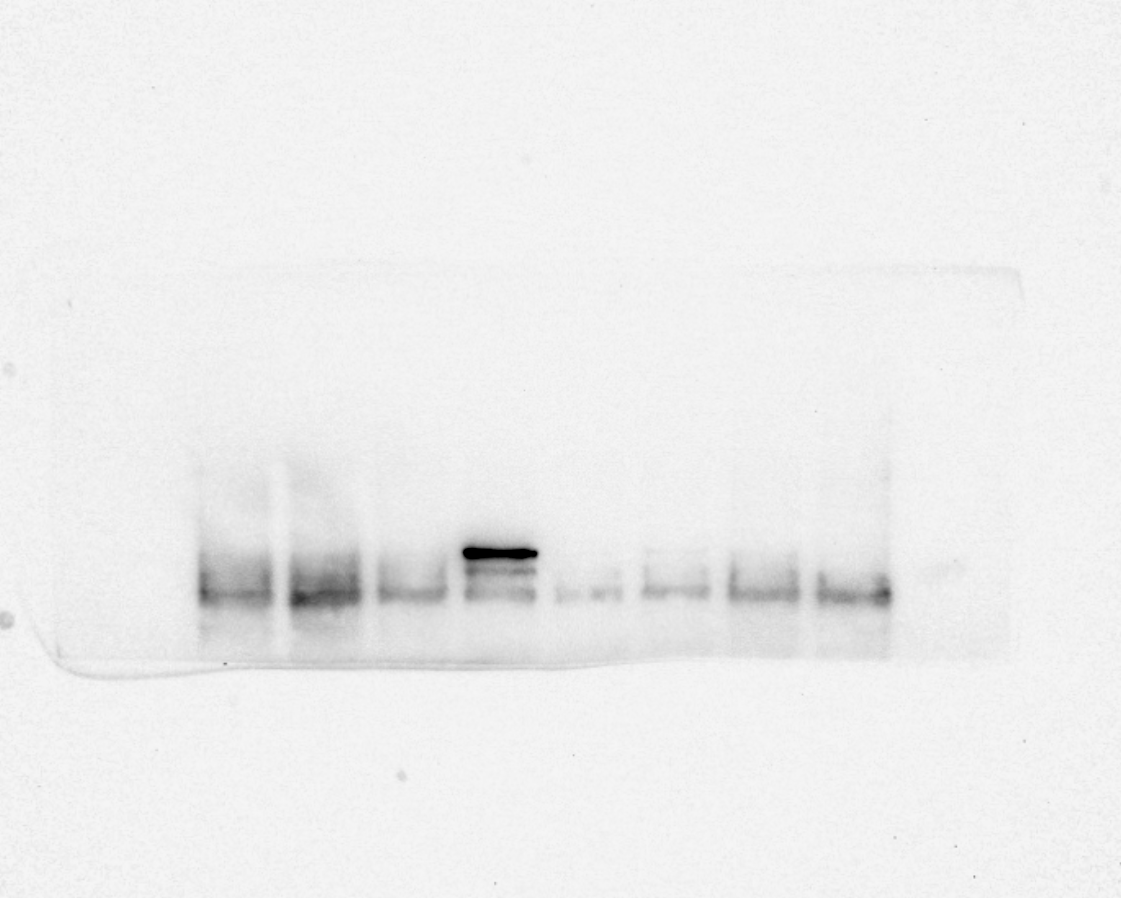

Supplement: Supplementary file 5 — Source data Fig. 4 [file 44319_2026_793_MOESM5_ESM.zip › Figure 4/Fig4D/GFP IP TBK1 IB.tif]

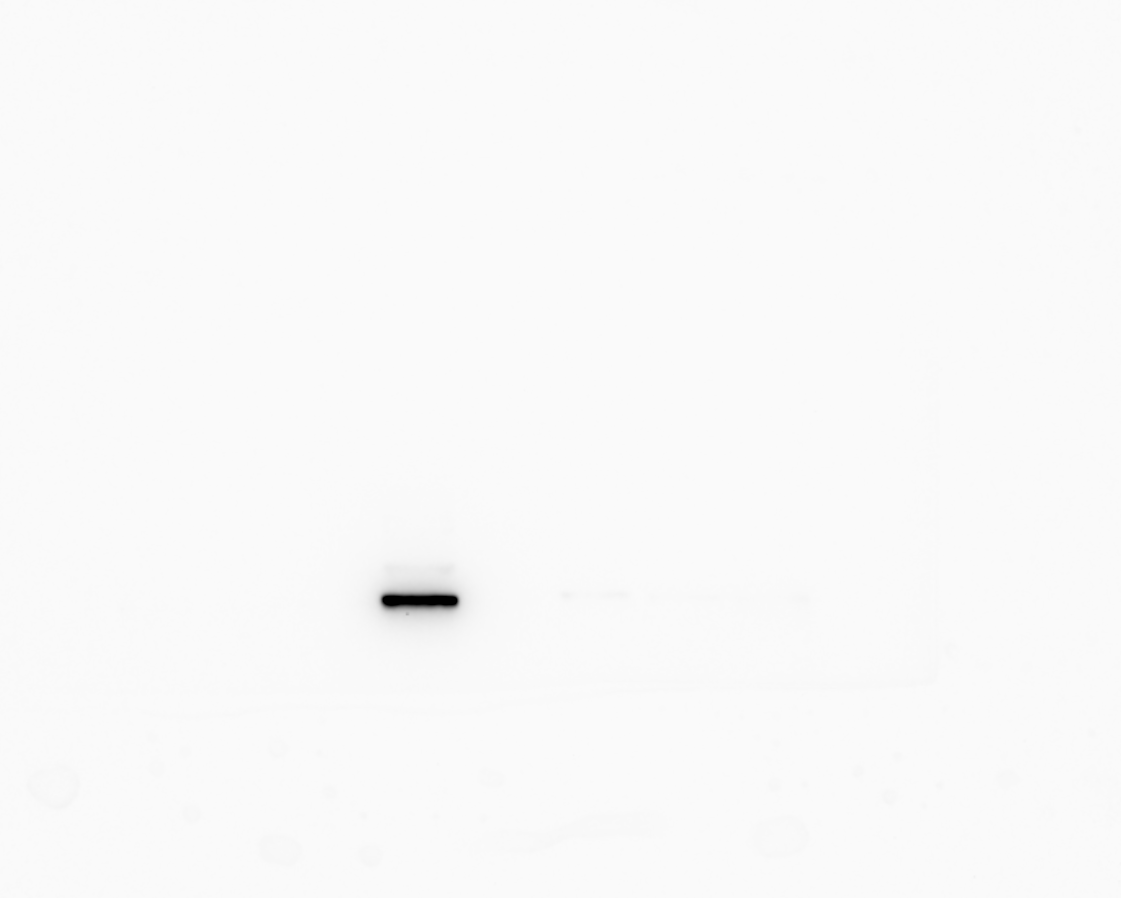

Supplement: Supplementary file 5 — Source data Fig. 4 [file 44319_2026_793_MOESM5_ESM.zip › Figure 4/Fig4D/GFP IP IKKe IB.tif]

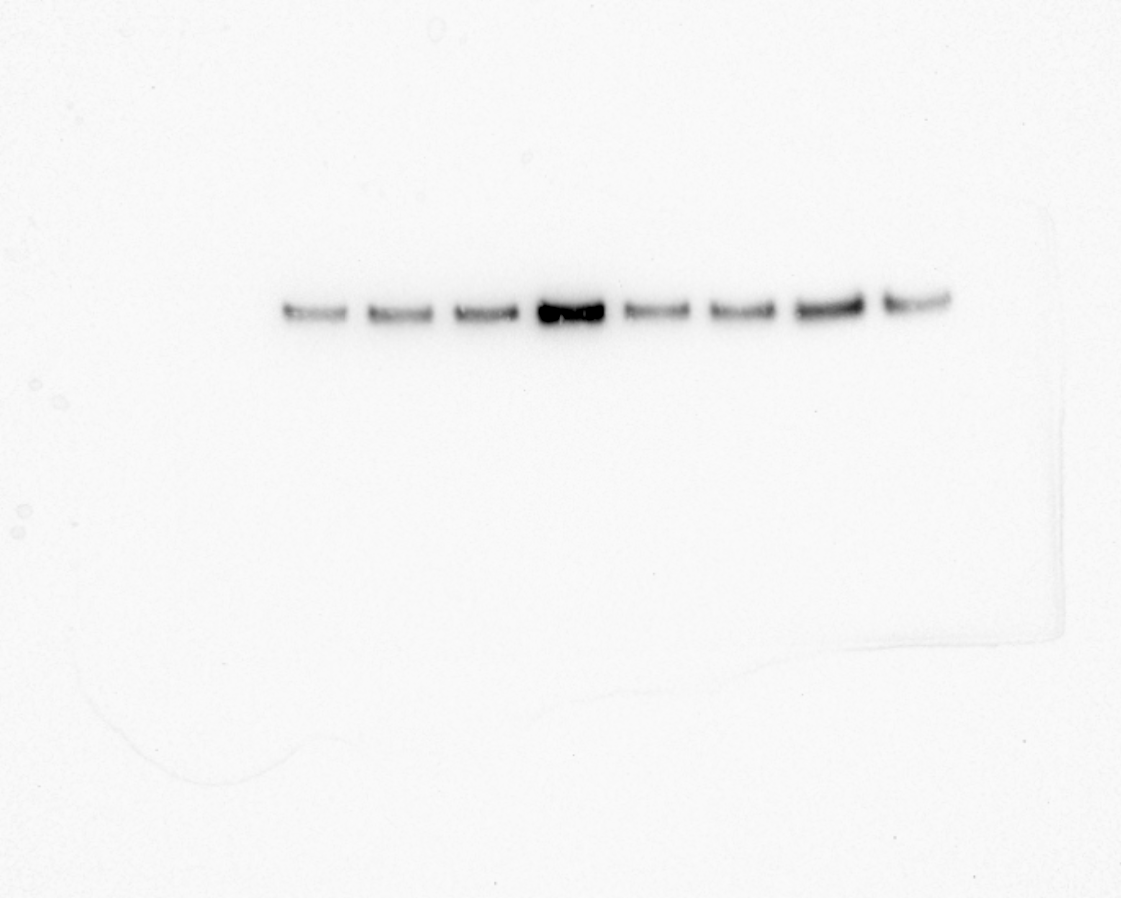

Supplement: Supplementary file 6 — Source data Fig. 5 [file 44319_2026_793_MOESM6_ESM.zip › Figure 5/Fig5D/P-cFos.tif]

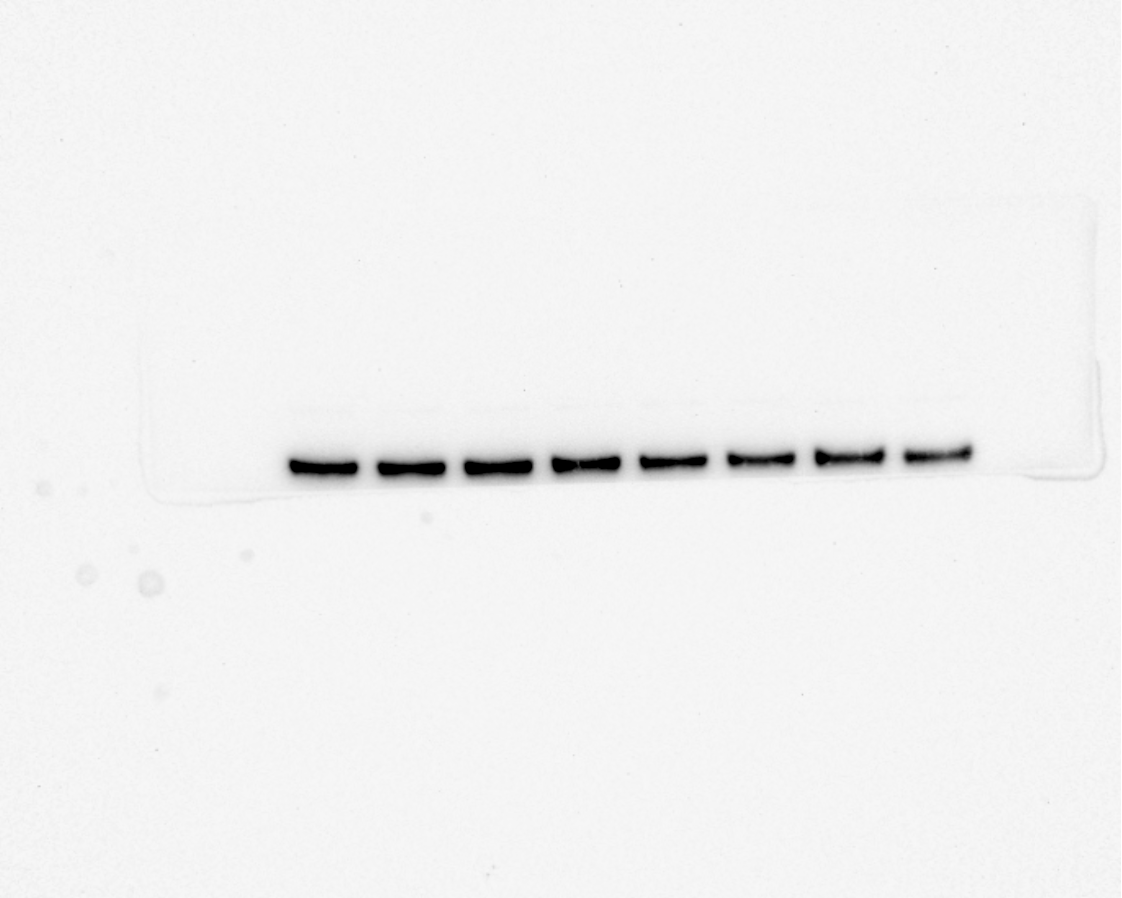

Supplement: Supplementary file 6 — Source data Fig. 5 [file 44319_2026_793_MOESM6_ESM.zip › Figure 5/Fig5D/p65.tif]

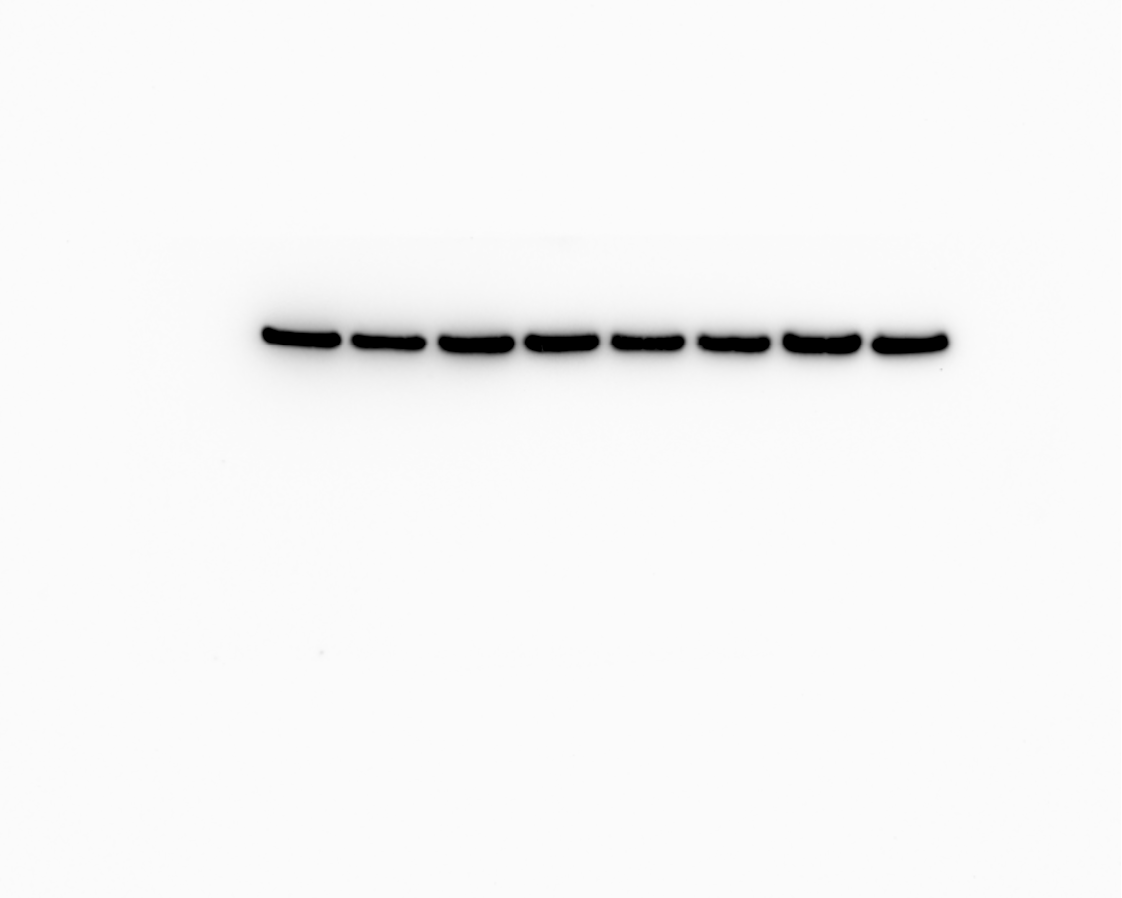

Supplement: Supplementary file 6 — Source data Fig. 5 [file 44319_2026_793_MOESM6_ESM.zip › Figure 5/Fig5D/ACTIN.tif]

Figure 5D\_uncropped blots

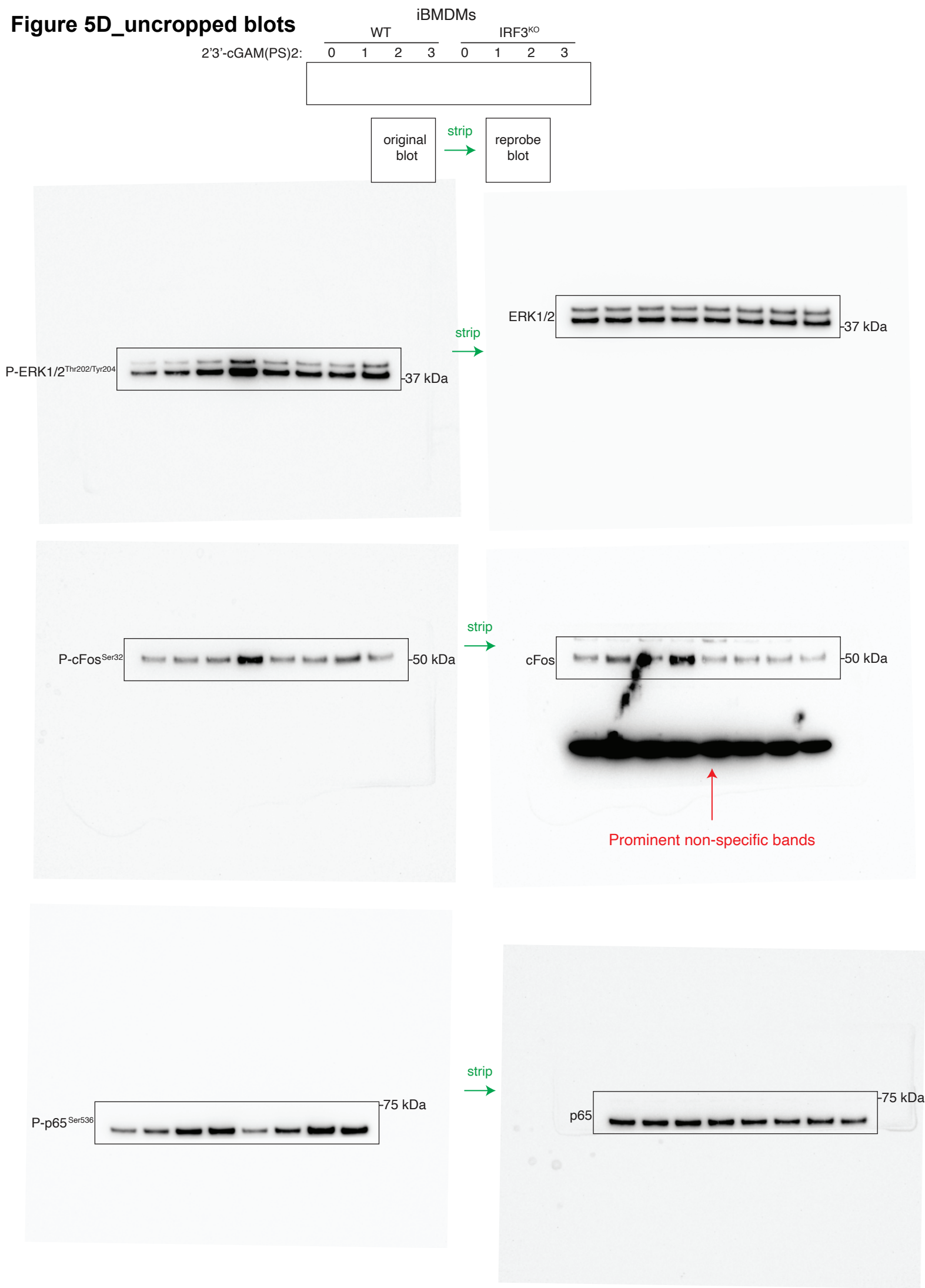

Figure 5D\_uncropped blots

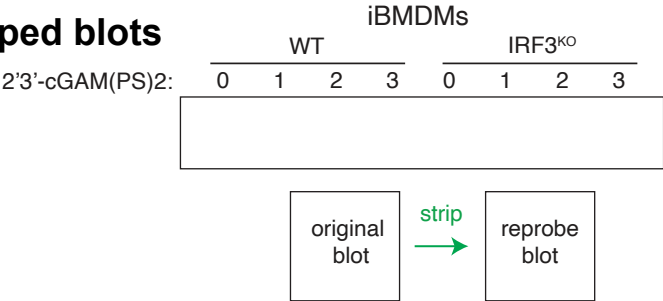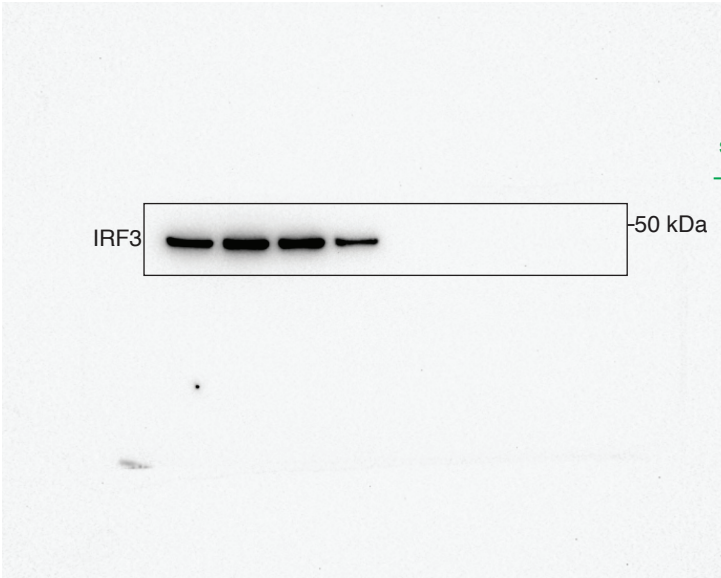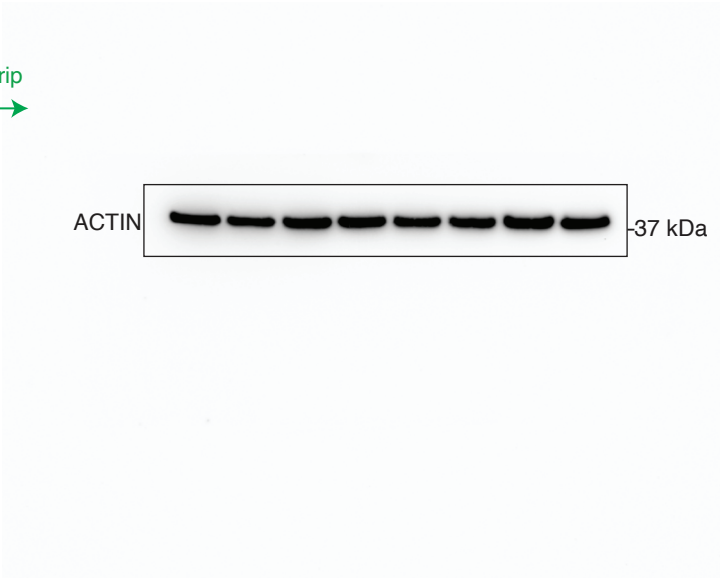

Supplement: Supplementary file 6 — Source data Fig. 5 [file 44319_2026_793_MOESM6_ESM.zip › Figure 5/Fig5D/Fig5D_uncropped blots.pdf]

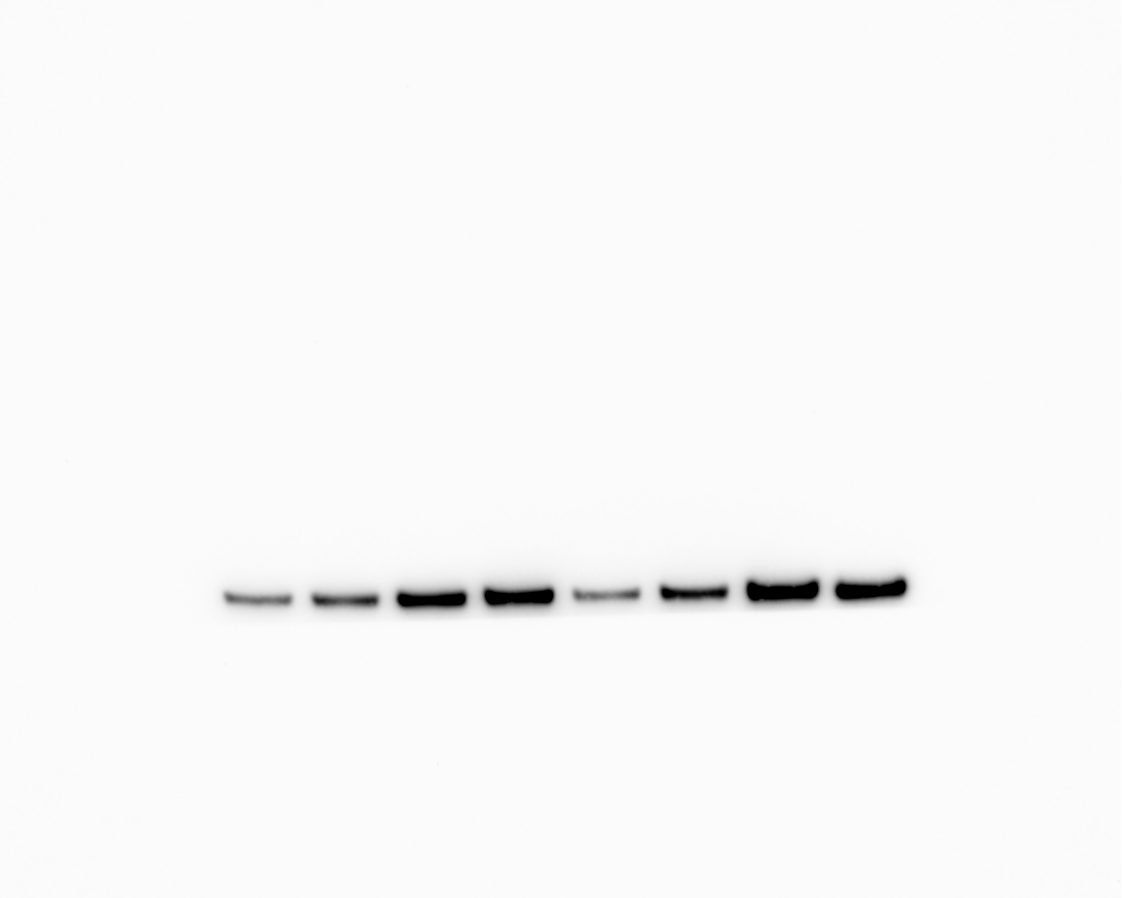

Supplement: Supplementary file 6 — Source data Fig. 5 [file 44319_2026_793_MOESM6_ESM.zip › Figure 5/Fig5D/P-p65.tif]

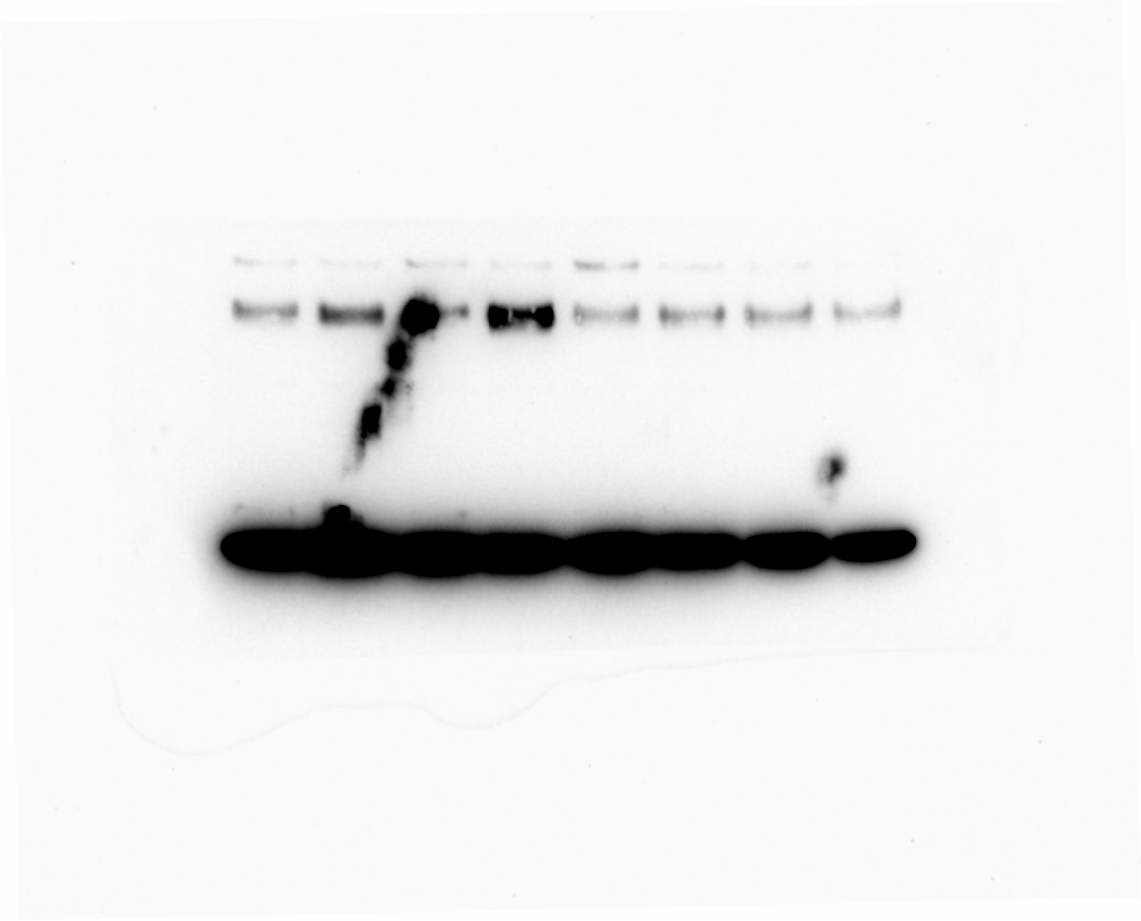

Supplement: Supplementary file 6 — Source data Fig. 5 [file 44319_2026_793_MOESM6_ESM.zip › Figure 5/Fig5D/cFos.tif]

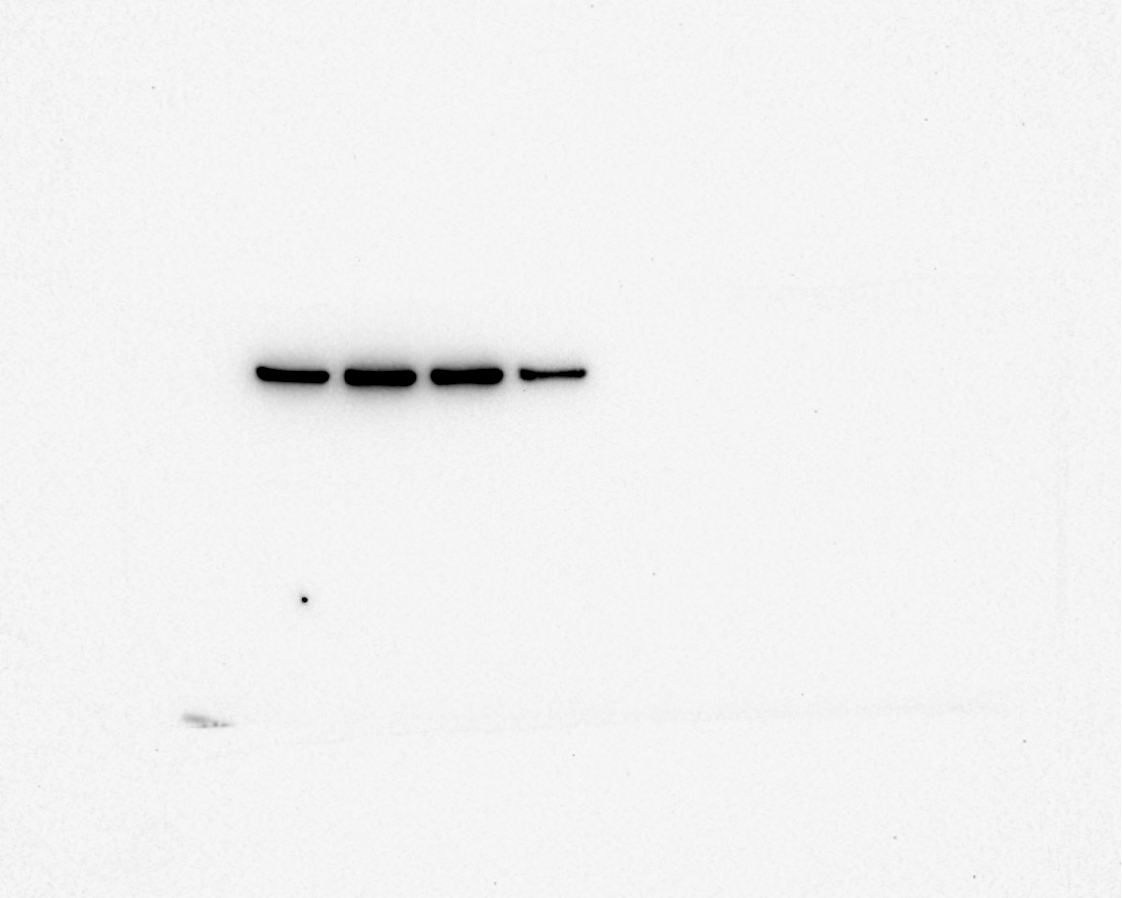

Supplement: Supplementary file 6 — Source data Fig. 5 [file 44319_2026_793_MOESM6_ESM.zip › Figure 5/Fig5D/IRF3.tif]

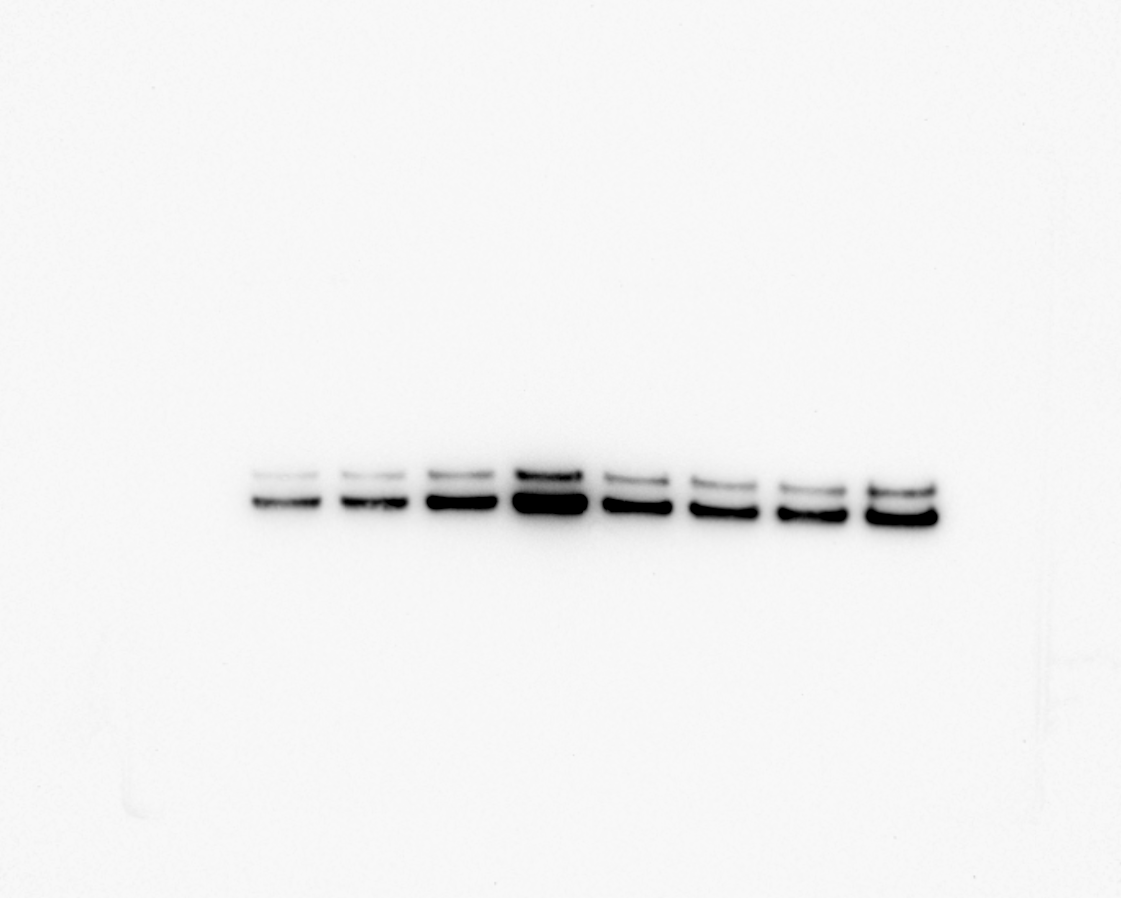

Supplement: Supplementary file 6 — Source data Fig. 5 [file 44319_2026_793_MOESM6_ESM.zip › Figure 5/Fig5D/P-ERK1:2.tif]

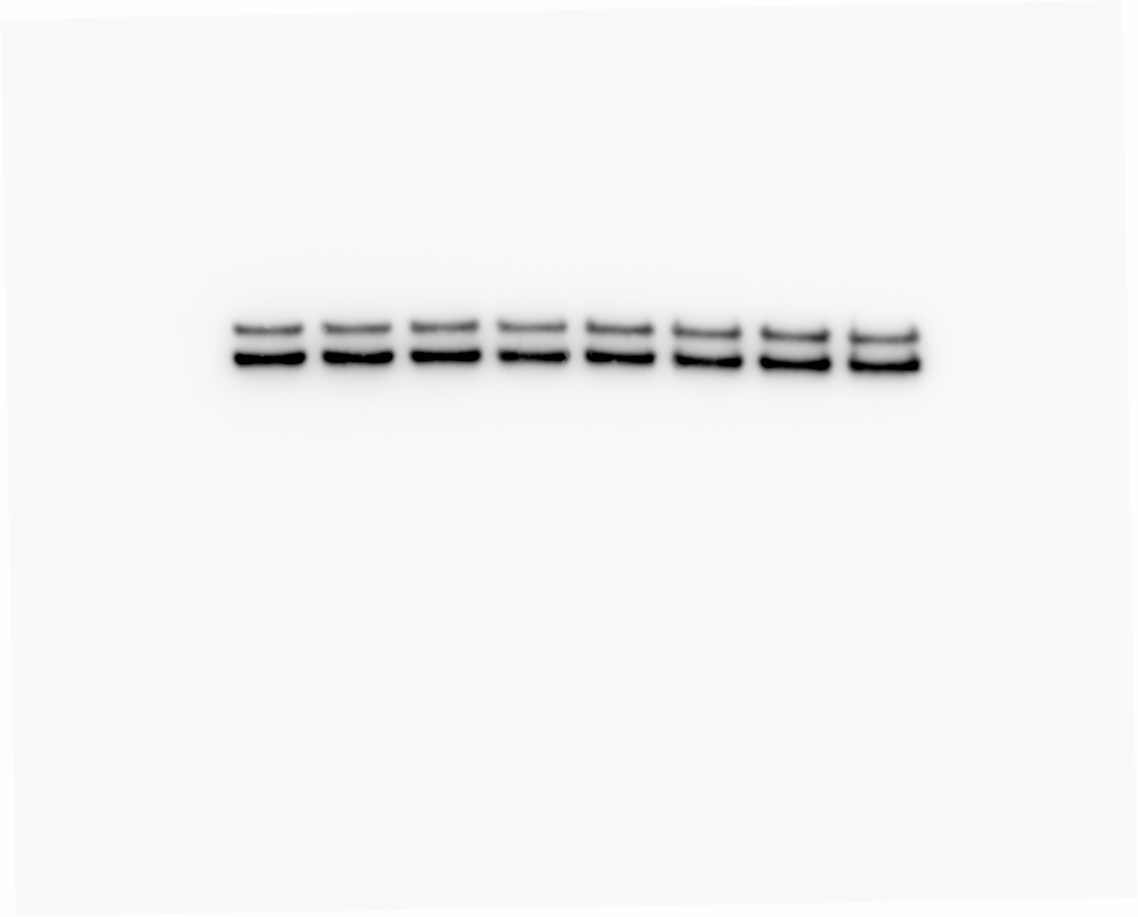

Supplement: Supplementary file 6 — Source data Fig. 5 [file 44319_2026_793_MOESM6_ESM.zip › Figure 5/Fig5D/ERK1:2.tif]

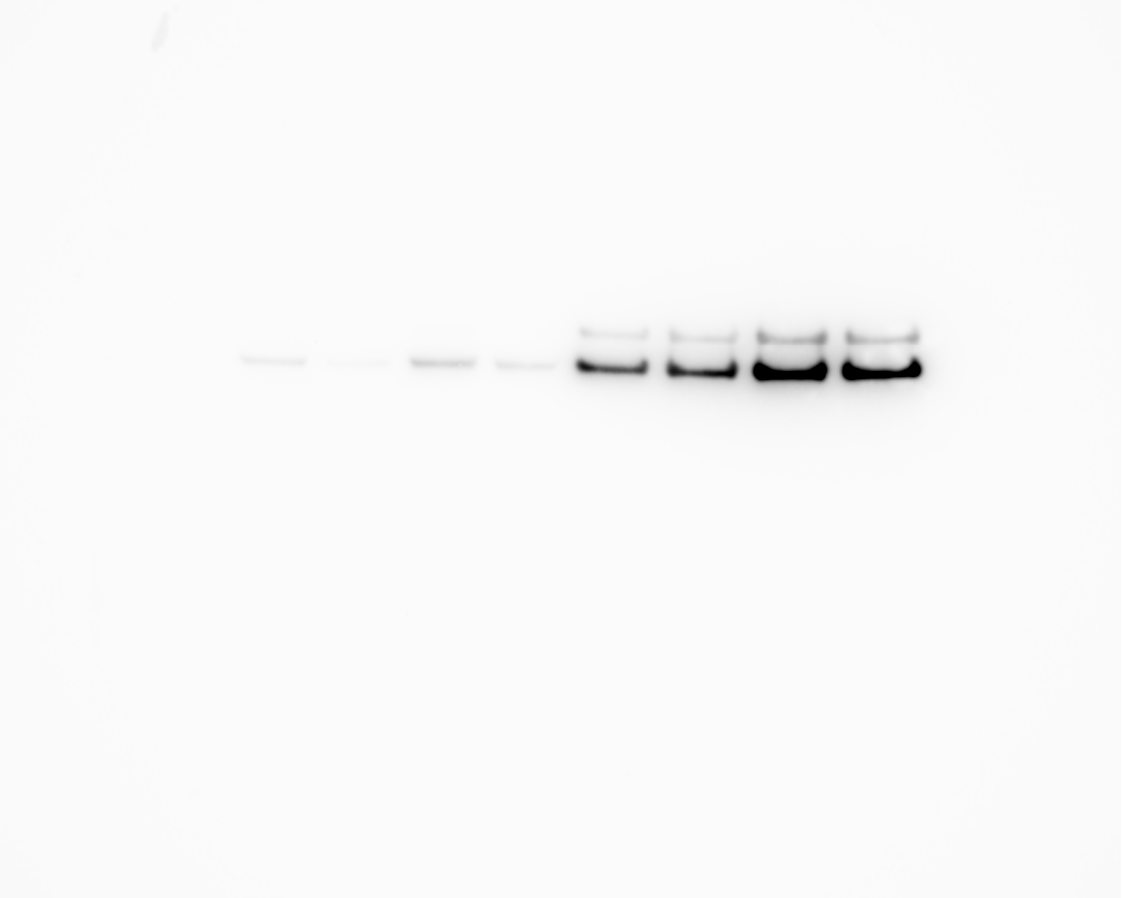

Supplement: Supplementary file 6 — Source data Fig. 5 [file 44319_2026_793_MOESM6_ESM.zip › Figure 5/Fig5C/LAMINA:C.tif]

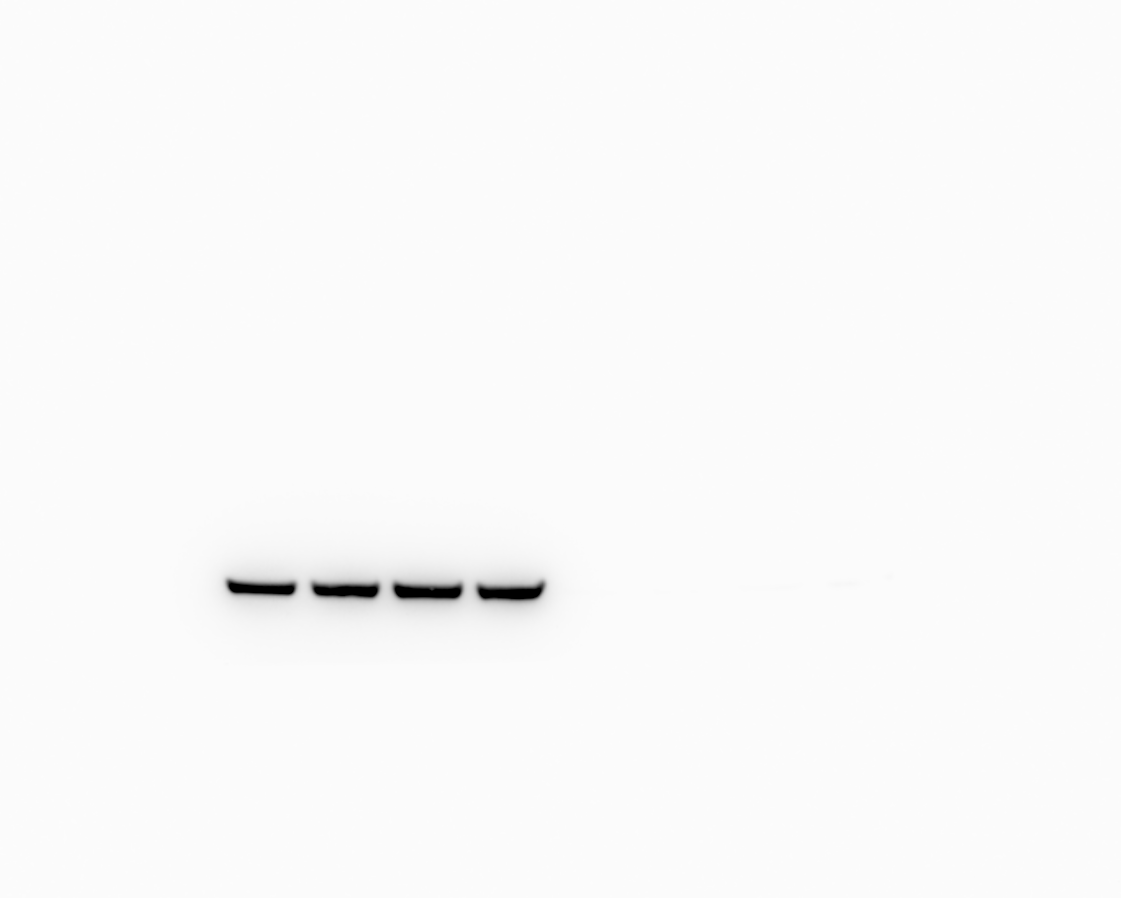

Supplement: Supplementary file 6 — Source data Fig. 5 [file 44319_2026_793_MOESM6_ESM.zip › Figure 5/Fig5C/HSP70.tif]

Figure 5C\_uncropped blots

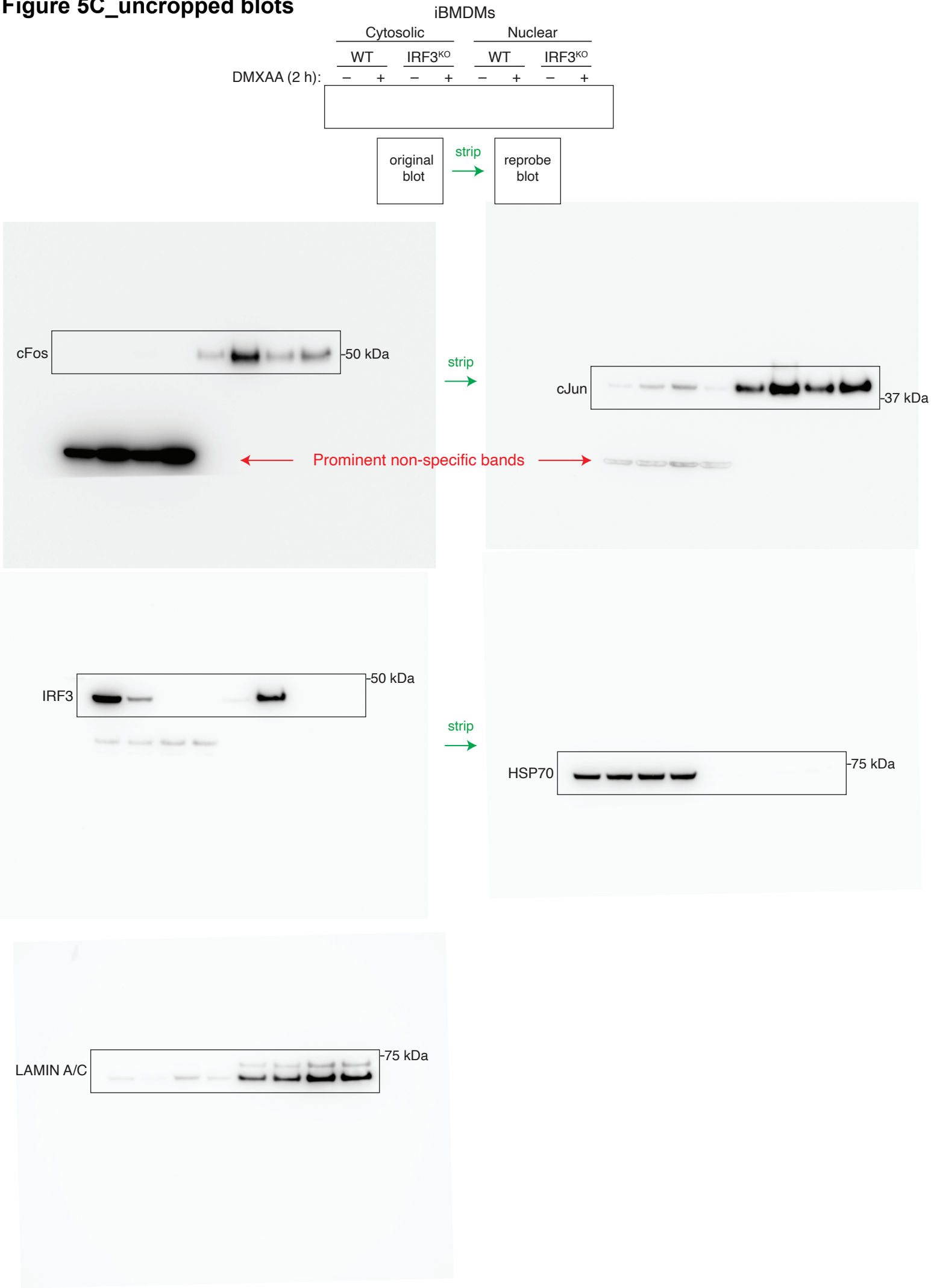

Supplement: Supplementary file 6 — Source data Fig. 5 [file 44319_2026_793_MOESM6_ESM.zip › Figure 5/Fig5C/Fig5C_uncropped blots.pdf]

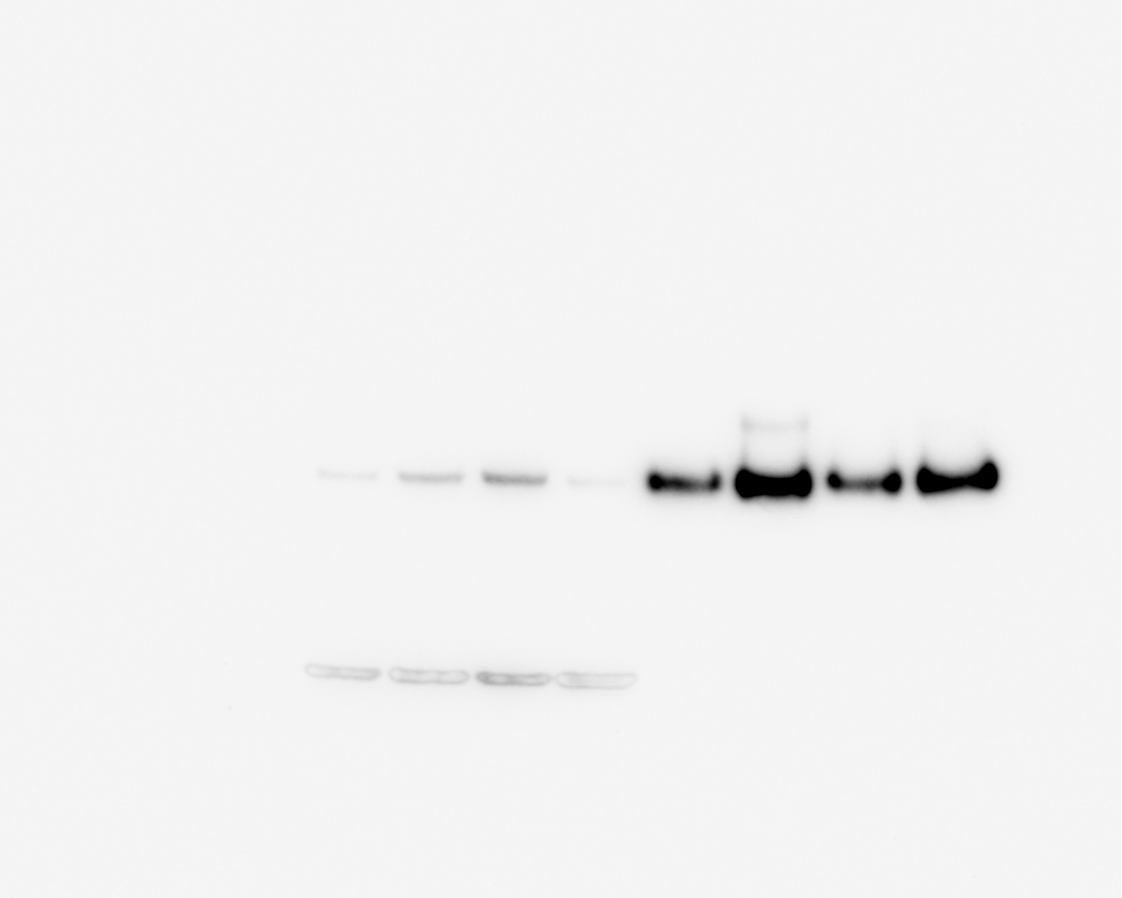

Supplement: Supplementary file 6 — Source data Fig. 5 [file 44319_2026_793_MOESM6_ESM.zip › Figure 5/Fig5C/cJun.tif]

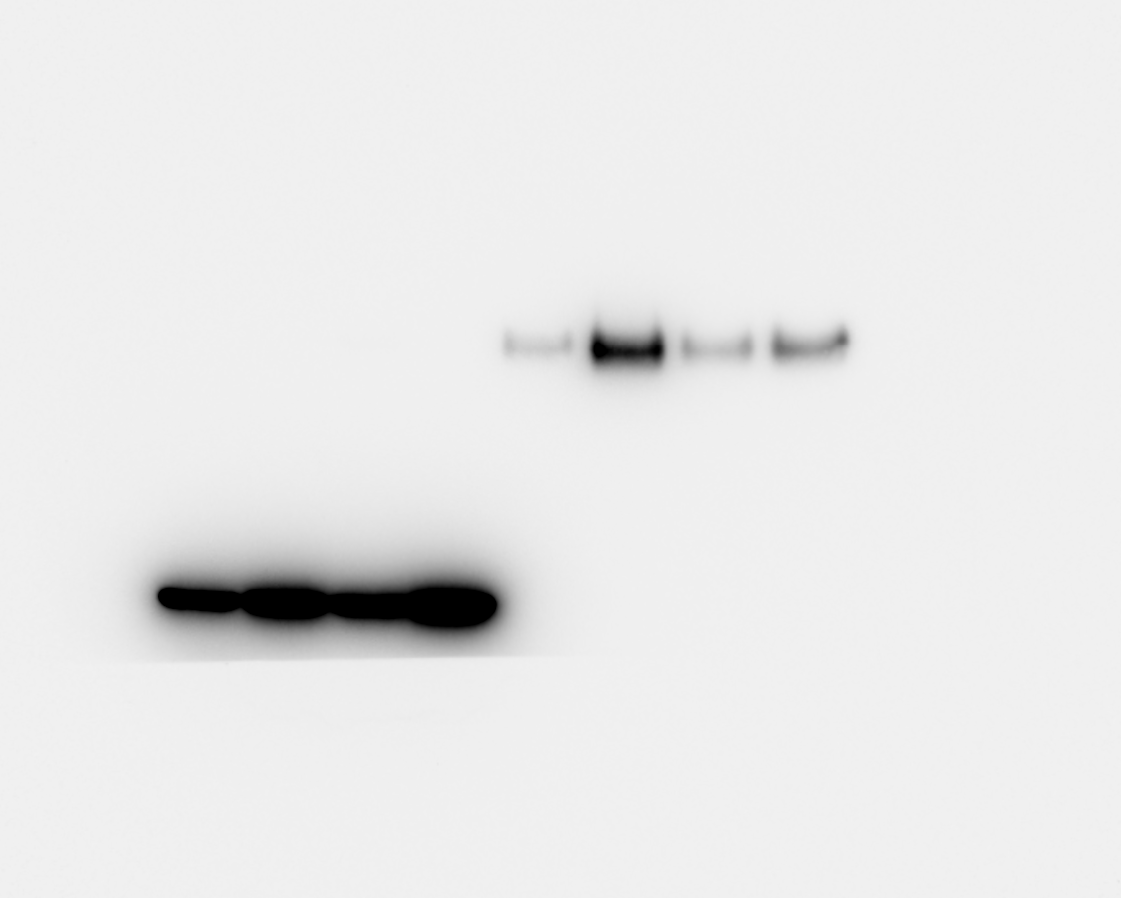

Supplement: Supplementary file 6 — Source data Fig. 5 [file 44319_2026_793_MOESM6_ESM.zip › Figure 5/Fig5C/cFos.tif]

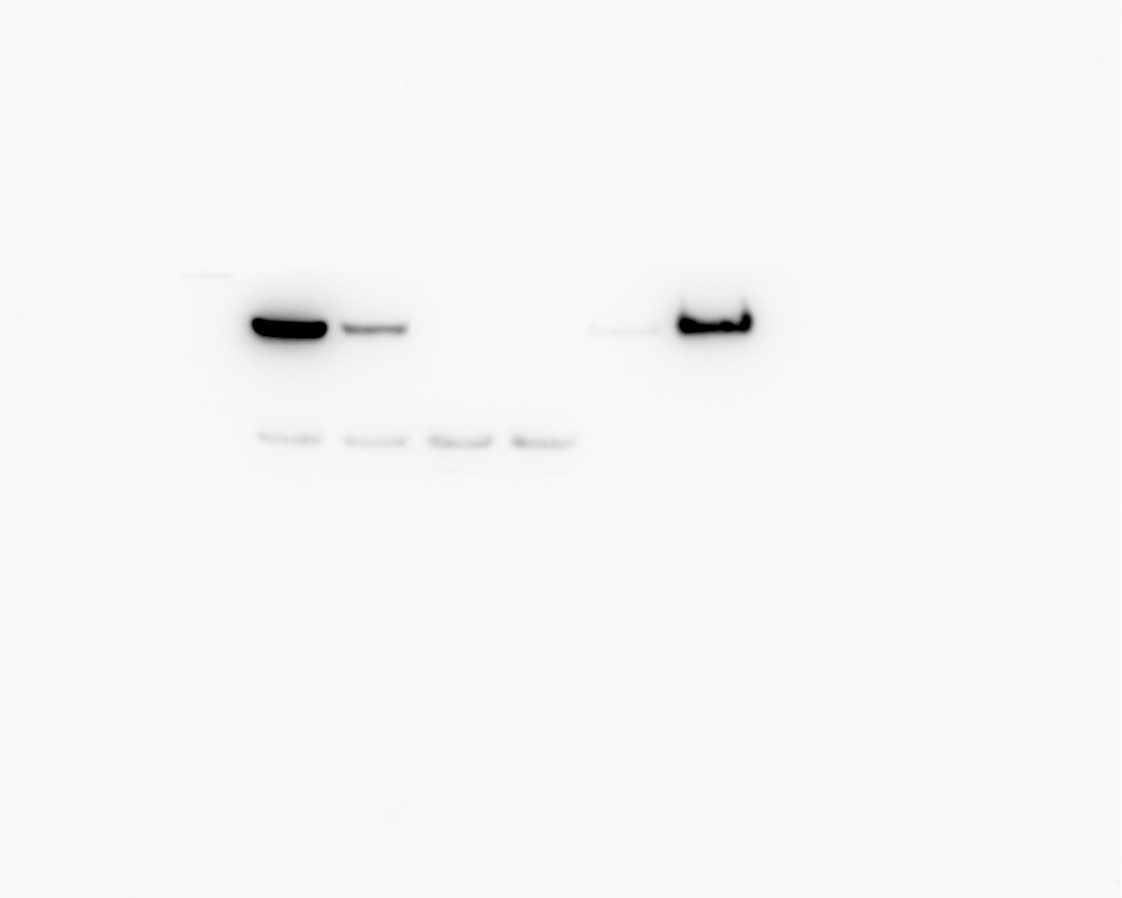

Supplement: Supplementary file 6 — Source data Fig. 5 [file 44319_2026_793_MOESM6_ESM.zip › Figure 5/Fig5C/IRF3.tif]
