# Supplementary material for: Noncanonical IRF3 function mediates STING-dependent pro-inflammatory cytokine production in macrophages
Source: EMBO Rep. 2026 May 8;27(11):2865–92. doi: 10.1038/s44319-026-00793-6 (PMC13260819; doi:10.1038/s44319-026-00793-6)

Figure 3D_uncropped blots

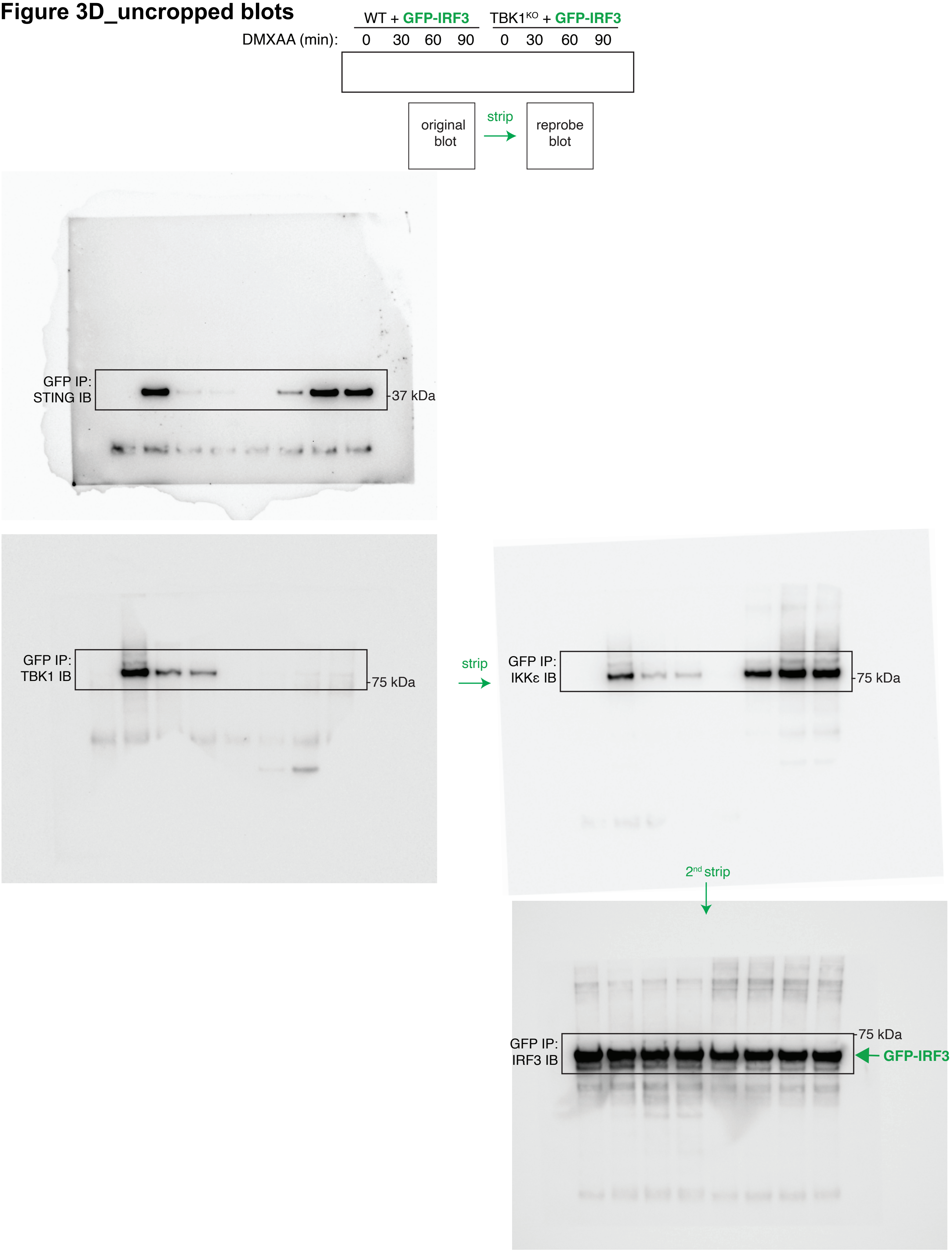

Figure 3D_uncropped blots

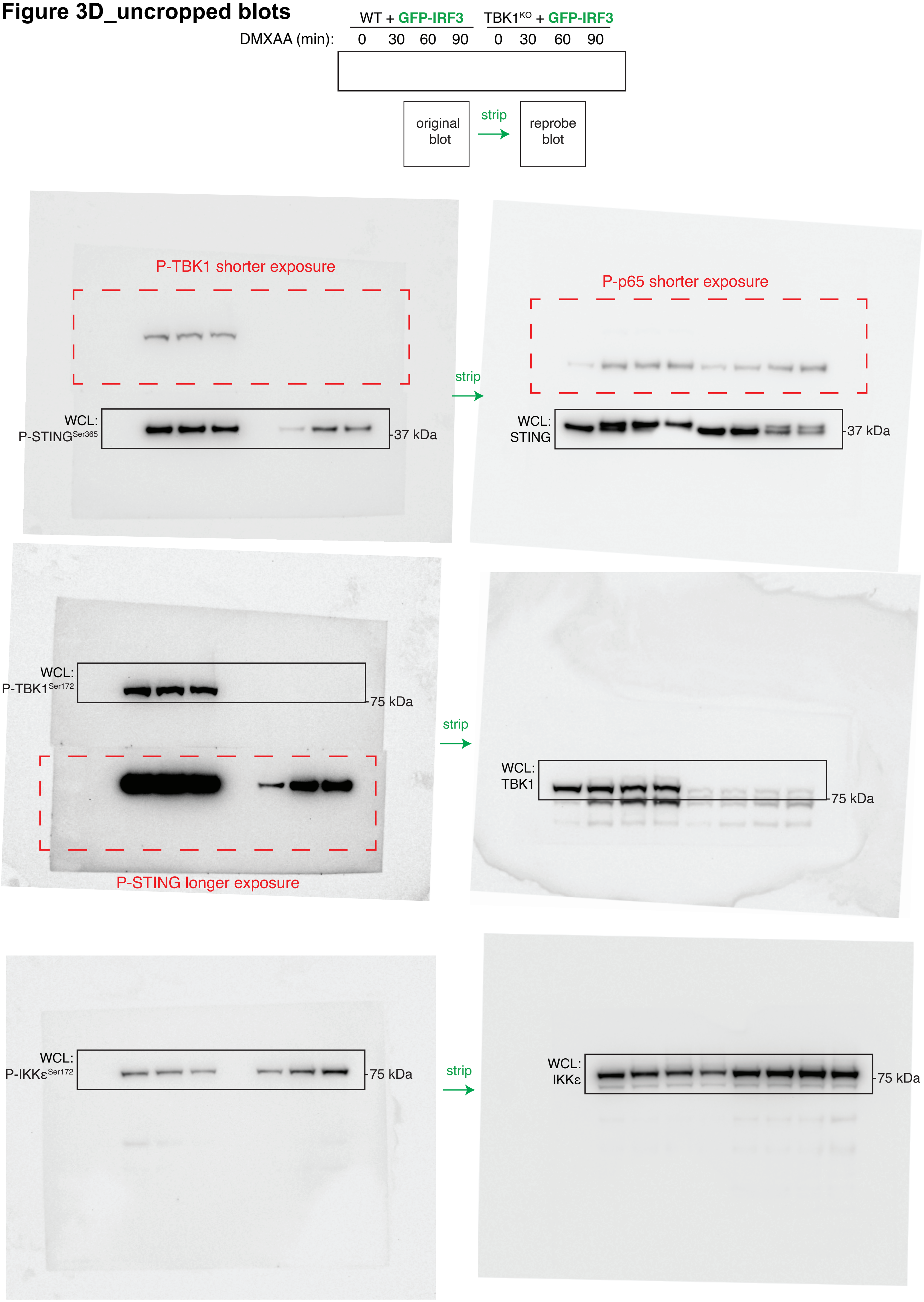

Figure 3D_uncropped blots

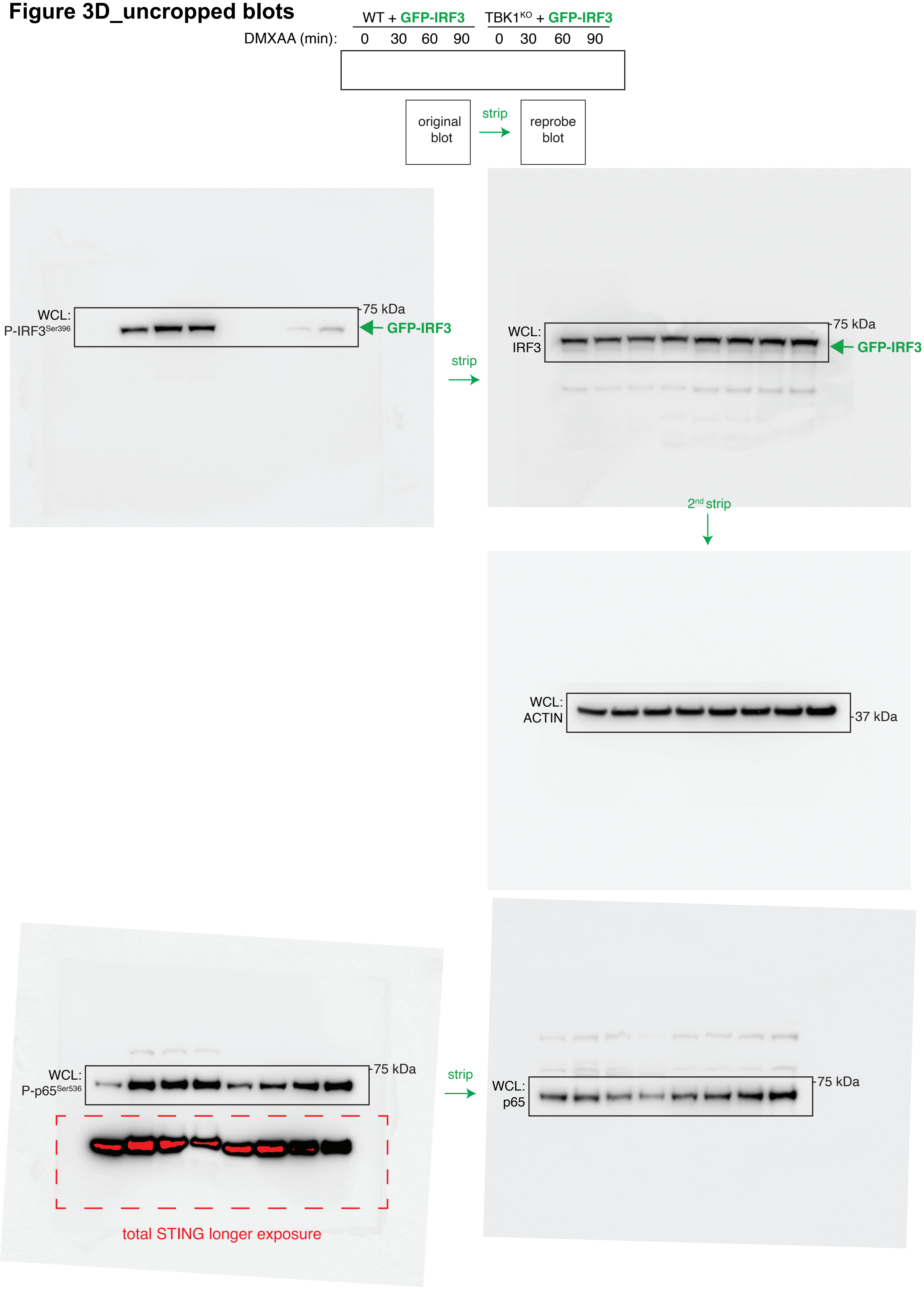

Supplement: Supplementary file 4 — Source data Fig. 3 [file 44319_2026_793_MOESM4_ESM.zip › Figure 3/Fig3D/Fig3D_uncropped blots.pdf]
