# Supplementary material for: Noncanonical IRF3 function mediates STING-dependent pro-inflammatory cytokine production in macrophages
Source: EMBO Rep. 2026 May 8;27(11):2865–92. doi: 10.1038/s44319-026-00793-6 (PMC13260819; doi:10.1038/s44319-026-00793-6)
Supplement: Supplementary file 7 — Expanded View Figures [file 44319_2026_793_MOESM7_ESM.pdf]

Expanded View Figures

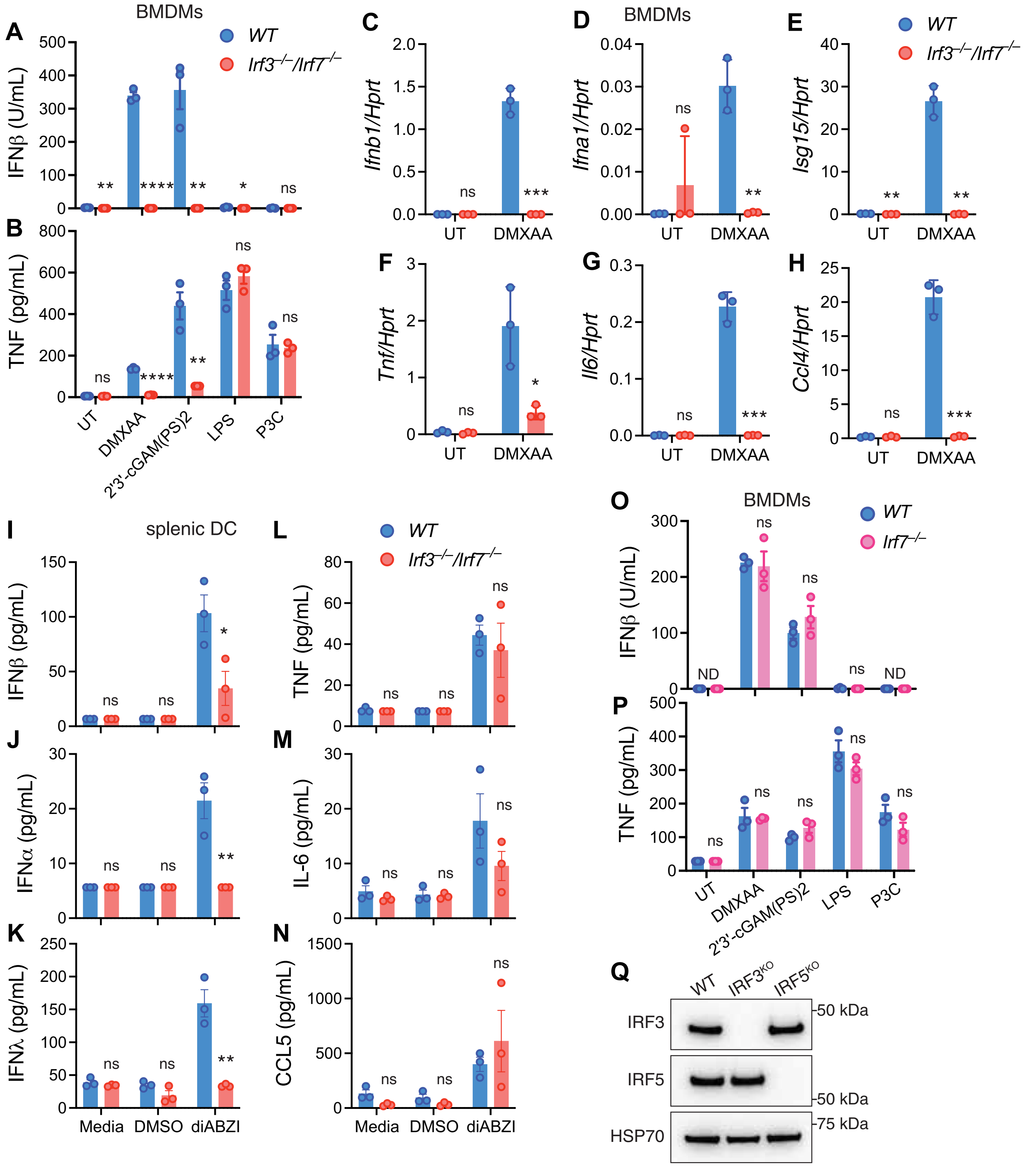

 © The Author(s)

Figure EV1. Lack of IRF3, but not IRF7, impacts the ability of macrophages to produce IFNs and inflammatory cytokines.

(**A**, **B**) Wild-type (WT) and *Irf3*$^{-/-}$/*Irf7*$^{-/-}$ primary BMDMs were left untreated (UT) or stimulated with 50 µg/mL DMXAA, 10 µg/mL 2′3′-cGAM(PS)2, 200 ng/mL LPS or 200 ng/mL Pam3CysK4 (P3C) for 4 h before cell supernatants were collected and levels of secreted IFNβ and TNF were measured by ELISA. Data shown as mean ± SEM from three combined biological experiments. Statistical analysis between genotypes for each condition group was performed using unpaired Student's *t* test. *$P < 0.05$ (**A** $P = 0.027$), **$P < 0.01$ (**A** $P = 0.0025$, $P = 0.0034$; **B** $P = 0.0042$), ****$P < 0.0001$ (**B** $P = 2.4839E\text{-}06$). ns= non-significant (**A** $P = 0.37$; **B** $P = 0.28$, $P = 0.32$, $P = 0.76$). (**C–H**) Wild-type (WT) and *Irf3*$^{-/-}$/*Irf7*$^{-/-}$ primary BMDMs were left untreated (UT) or stimulated with 50 µg/mL DMXAA for 4 h before cells for RNA isolation and the expression of IFN and pro-inflammatory genes were analysed by qPCR as indicated. Data shown as mean ± SEM from three combined biological experiments. Statistical analysis between genotypes for each condition group was performed using unpaired Student's *t* test. *$P < 0.05$ (**F** $P = 0.02$), **$P < 0.01$ (**D** $P = 0.0011$), ***$P < 0.001$ (**C** $P = 0.0001$, **E** $P = 0.0.0005$, $P = 0.00022$; **G** $P = 0.00011$; **H** $P = 0.00015$). ns= non-significant (**C** $P = 0.66$; **D** $P = 0.37$; **F** $P = 0.3$; **G** $P = 0.86$; **H** $P = 0.92$). (**I–N**) Primary bulk splenic DCs from wild-type (WT) and *Irf3*$^{-/-}$/*Irf7*$^{-/-}$ mice were left in fresh media or stimulated with DMSO control or 0.2 µM diABZI for 18 h before cell supernatants were collected to measure levels of secreted cytokines by LEGENDplex™ assay. Data shown as mean ± SEM from three combined biological experiments. Statistical analysis between genotypes for each condition group was performed using unpaired Student's *t* test. *$P < 0.05$ (**I** $P = 0.04$), **$P < 0.01$ (**J** $P = 0.008$; **K** $P = 0.004$). ns= non-significant (**K** $P = 0.31$, $P = 0.11$; **L** $P = 0.37$; **M** $P = 0.28$, $P = 0.76$, $P = 0.22$; **N** $P = 0.12$, $P = 0.5$). (**O**, **P**) Wild-type (WT) and *Irf7*$^{-/-}$ primary BMDMs were left untreated (UT) or stimulated with 50 µg/mL DMXAA, 10 µg/mL 2′3′-cGAM(PS)2, 200 ng/mL LPS or 200 ng/mL Pam3CysK4 (P3C) for 4 h before cell supernatants were collected and levels of secreted IFNβ and TNF were measured by ELISA. Data shown as mean ± SEM from three combined biological experiments. Statistical analysis between genotypes for each condition group was performed using unpaired Student's *t* test. ns= non-significant (**O** $P = 0.83$, $P = 0.27$, $P = 0.37$; **P** $P = 0.15$, $P = 0.79$, $P = 0.14$, $P = 0.24$, $P = 0.16$). ND not determined. (**Q**) Wild-type (WT), IRF3^{KO} and IRF5^{KO} iBMDMs were lysed for immunoblot with the indicated antibodies. Data shown is representative of three biological experiments.

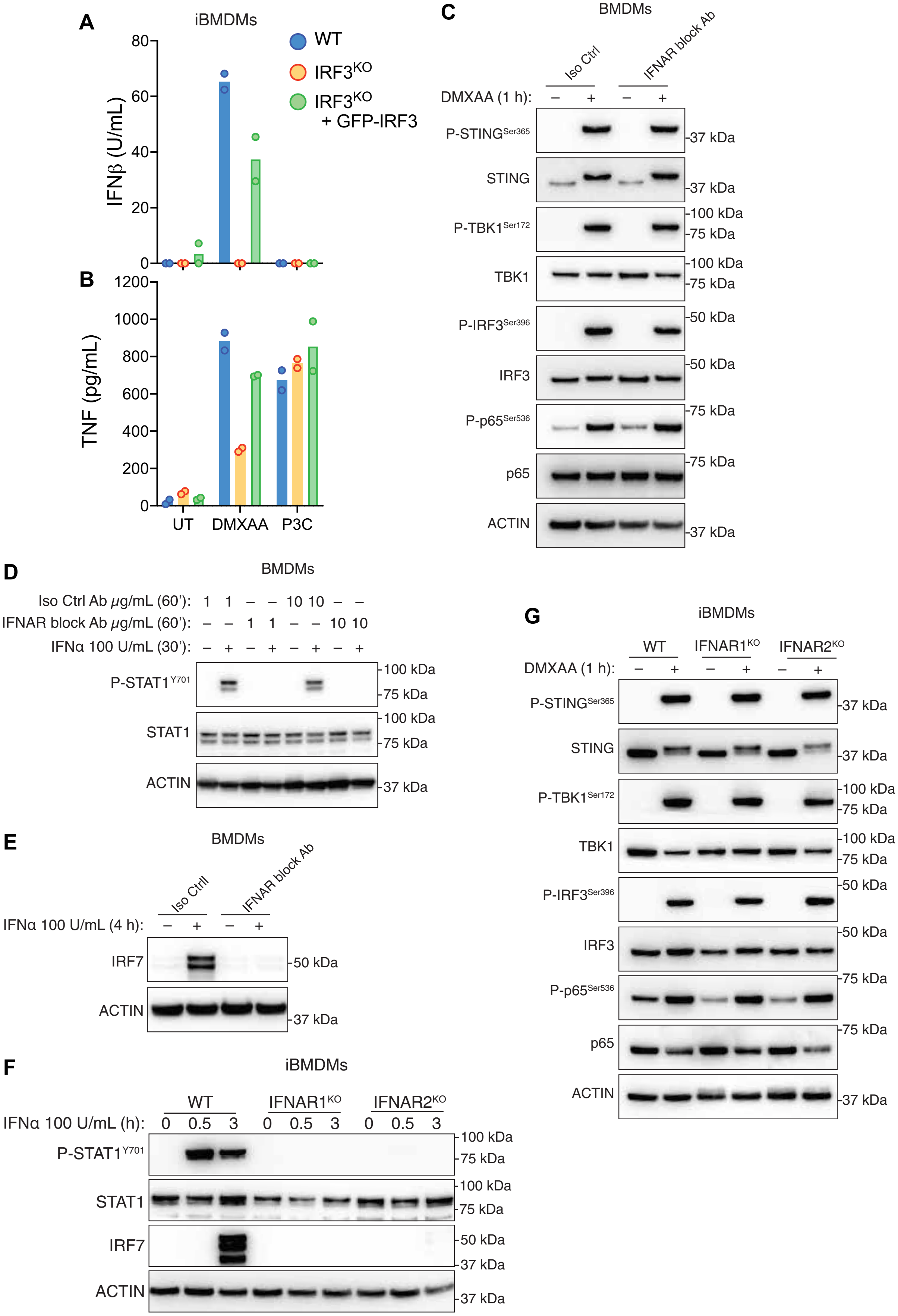
A
iBMDMs
WT
IRF3KO
IRF3KO + GFP-IRF3
IFNβ (U/mL)
B
TNF (pg/mL)
UT
DMXAA
P3C
C
BMDMs
Iso Ctrl
IFNAR block Ab
DMXAA (1 h):
P-STING Ser365
STING
P-TBK1 Ser172
TBK1
P-IRF3 Ser396
IRF3
P-p65 Ser536
p65
ACTIN
D
BMDMs
Iso Ctrl Ab μg/mL (60'): 1 1 – – 10 10 – –
IFNAR block Ab μg/mL (60'): – – 1 1 – – 10 10
IFNα 100 U/mL (30'): – + – + – + – +
P-STAT1 Y701
STAT1
ACTIN
E
BMDMs
Iso Ctrl
IFNAR block Ab
IFNα 100 U/mL (4 h):
IRF7
ACTIN
F
iBMDMs
WT
IFNAR1KO
IFNAR2KO
IFNα 100 U/mL (h): 0 0.5 3 0 0.5 3 0 0.5 3
P-STAT1 Y701
STAT1
IRF7
ACTIN
G
iBMDMs
WT
IFNAR1KO
IFNAR2KO
DMXAA (1 h):
P-STING Ser365
STING
P-TBK1 Ser172
TBK1
P-IRF3 Ser396
IRF3
P-p65 Ser536
p65
ACTIN

 © The Author(s)

Figure EV2. Blocking or deletion of IFNAR abolishes type I IFN responses.

(**A-B**) Wild-type (WT), IRF3^{KO} and IRF3^{KO} expressing GFP-IRF3 were left untreated (UT) or stimulated with 50 μg/mL DMXAA or 200 ng/mL Pam3CysK4 (P3C) for 4 h before cell supernatants were collected and levels of secreted IFNβ and TNF were measured by ELISA. Data shown as mean from two biological experiments. (**C**) Wild-type (WT) primary BMDMs were pre-treated for 1 h with 10 μg/mL isotype control antibody or IFNAR blocking antibody before being left untreated (–) or stimulated for 1 h with 50 μg/mL DMXAA (+). Cells were then lysed for immunoblot with the indicated antibodies. Data shown is representative of three biological experiments. (**D**) Wild-type (WT) primary BMDMs were pre-treated for 1 h with an isotype control antibody or IFNAR blocking antibody as indicated. Cells were then left untreated (–) or stimulated for 30 min with 100 U/mL IFNα (+). Cells were then lysed for immunoblot with the indicated antibodies. Data shown is representative of 3 biological experiments. (**E**) Wild-type (WT) primary BMDMs were pre-treated for 1 h with 10 μg/mL isotype control antibody or IFNAR blocking antibody. Cells were then left untreated (–) or stimulated for 4 h with 100 U/mL IFNα (+) before they were lysed for immunoblot with the indicated antibodies. Data shown is representative of three biological experiments. (**F**) Wild-type (WT), IFNAR1^{KO} and IFNAR2^{KO} iBMDMs were left untreated (0) or stimulated for 0.5 or 3 h with 100 U/mL IFNα before cells were lysed for immunoblot with the indicated antibodies. Data shown is representative of three biological experiments. (**G**) Wild-type (WT), IFNAR1^{KO} and IFNAR2^{KO} iBMDMs were left untreated (–) or stimulated for 1 h with 50 μg/mL DMXAA (+). Cells were then lysed for immunoblot with the indicated antibodies. Data shown is representative of three biological experiments.

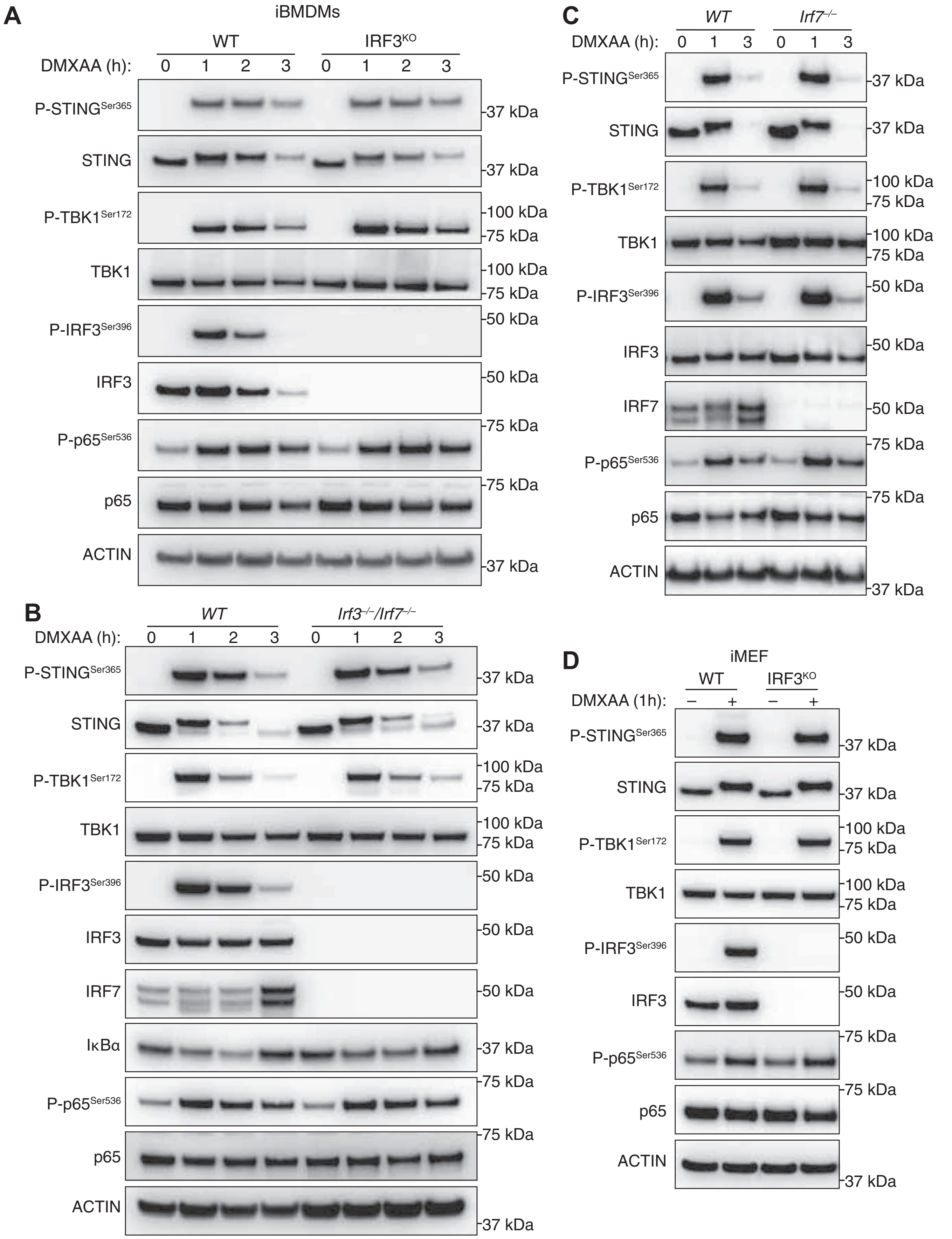
A
iBMDMs
WT
IRF3KO
DMXAA (h): 0 1 2 3 0 1 2 3
P-STINGSer365
STING
P-TBK1Ser172
TBK1
P-IRF3Ser396
IRF3
P-p65Ser536
p65
ACTIN
37 kDa
37 kDa
100 kDa
75 kDa
100 kDa
75 kDa
50 kDa
50 kDa
75 kDa
75 kDa
37 kDa
B
WT
Irf3−/−/Irf7−/−
DMXAA (h): 0 1 2 3 0 1 2 3
P-STINGSer365
STING
P-TBK1Ser172
TBK1
P-IRF3Ser396
IRF3
IRF7
IκBα
P-p65Ser536
p65
ACTIN
37 kDa
37 kDa
100 kDa
75 kDa
100 kDa
75 kDa
50 kDa
50 kDa
50 kDa
37 kDa
75 kDa
75 kDa
37 kDa
C
WT
Irf7−/−
DMXAA (h): 0 1 3 0 1 3
P-STINGSer365
STING
P-TBK1Ser172
TBK1
P-IRF3Ser396
IRF3
IRF7
P-p65Ser536
p65
ACTIN
37 kDa
37 kDa
100 kDa
75 kDa
100 kDa
75 kDa
50 kDa
50 kDa
50 kDa
75 kDa
75 kDa
37 kDa
D
iMEF
WT
IRF3KO
DMXAA (1h): − + − +
P-STINGSer365
STING
P-TBK1Ser172
TBK1
P-IRF3Ser396
IRF3
P-p65Ser536
p65
ACTIN
37 kDa
37 kDa
100 kDa
75 kDa
100 kDa
75 kDa
50 kDa
50 kDa
75 kDa
75 kDa
37 kDa

© The Author(s)

Figure EV3. STING signalling is unchanged in the absence of IRF3 and/or IRF7.

(**A**) Wild-type (WT) and IRF3^{KO} iBMDMs were left untreated (0) or stimulated for 1, 2 or 3 h with 50 μg/mL DMXAA. Cells were then lysed for immunoblot with the indicated antibodies. Data shown is representative of 3 biological experiments. (**B**) Wild-type (WT) and *Irf3*$^{-/-}$/*Irf7*$^{-/-}$ primary BMDMs were left untreated (0) or stimulated for 1, 2 or 3 h with 50 μg/mL DMXAA. Cells were then lysed for immunoblot with the indicated antibodies. Data shown is representative of three biological experiments. (**C**) Wild-type (*WT*) and *Irf7*$^{-/-}$ primary BMDMs were left untreated (0) or stimulated for 1 and 3 h with 50 μg/mL DMXAA. Cells were then lysed for immunoblot with the indicated antibodies. Data shown is representative of three biological experiments. (**D**) Wild-type (WT) and IRF3^{KO} iMEFs were left untreated (–) or stimulated (+) with 50 μg/mL DMXAA for 1 h. Cells were then lysed for immunoblot with the indicated antibodies. Data shown is representative of three biological experiments.

© The Author(s)

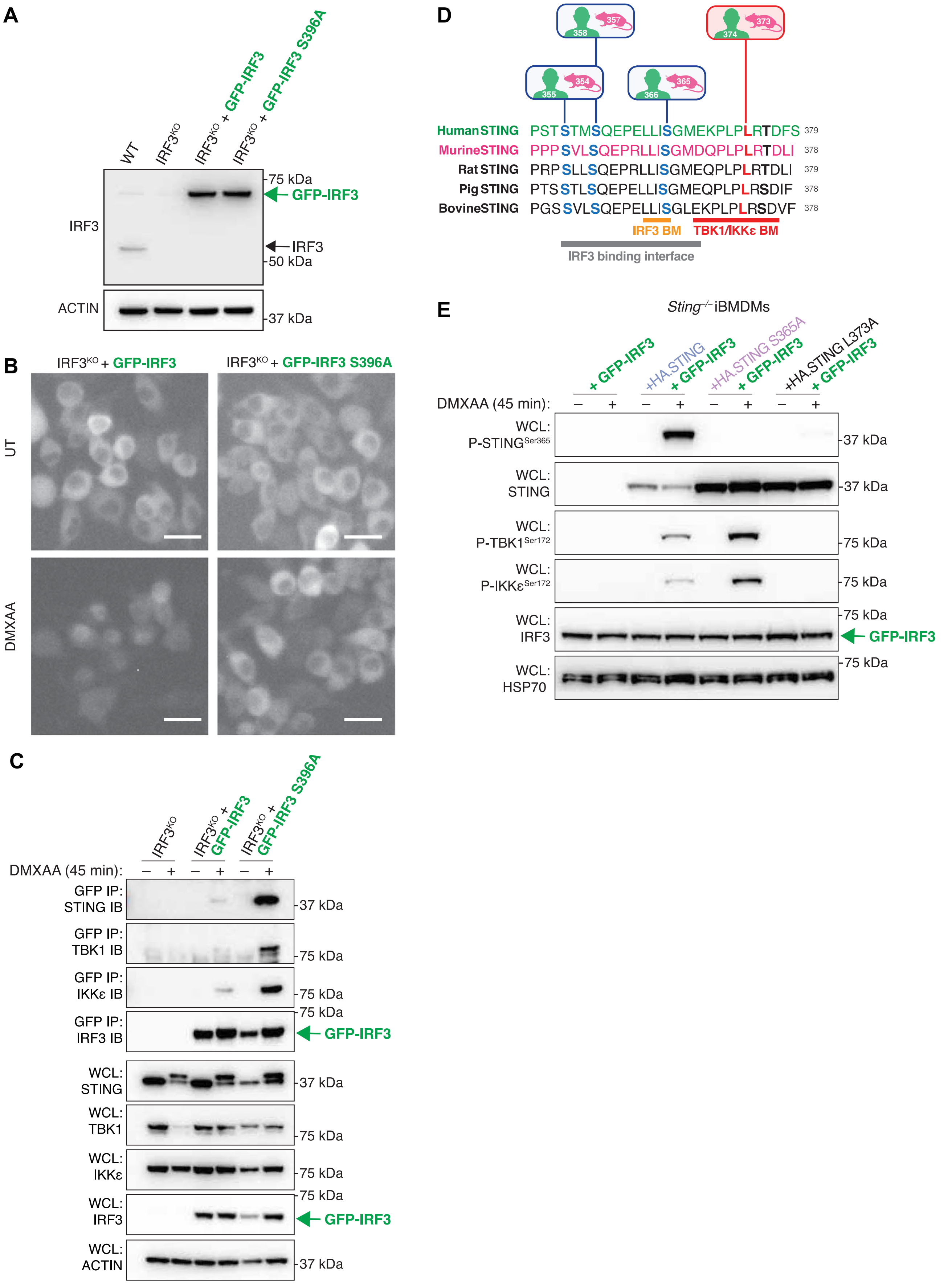
A
WT
IRF3KO
IRF3KO + GFP-IRF3
IRF3KO + GFP-IRF3 S396A
IRF3
75 kDa
GFP-IRF3
IRF3
50 kDa
ACTIN
37 kDa
B
IRF3KO + GFP-IRF3
IRF3KO + GFP-IRF3 S396A
UT
DMXAA
C
IRF3KO
IRF3KO + GFP-IRF3
IRF3KO + GFP-IRF3 S396A
DMXAA (45 min): – + – + – +
GFP IP: STING IB
37 kDa
GFP IP: TBK1 IB
75 kDa
GFP IP: IKKε IB
75 kDa
GFP IP: IRF3 IB
75 kDa
GFP-IRF3
WCL: STING
37 kDa
WCL: TBK1
75 kDa
WCL: IKKε
75 kDa
WCL: IRF3
75 kDa
GFP-IRF3
WCL: ACTIN
37 kDa
D
358 357
374 373
355 354
366 365
Human STING PSTSTMSQEPELLISGMEKPLPLRTDFS 379
Murine STING PPPSVLSQEPRLLISGMDQPLPLRTDLI 378
Rat STING PRPSLLSQEPRLLISGMEQPLPLRTDLI 379
Pig STING PTSSTLSQEPELLISGMEQPLPLRSDIF 378
Bovine STING PGSSVLSQEPELLISGLEKPLPLRSDVF 378
IRF3 BM
TBK1/IKKε BM
IRF3 binding interface
E
Sting−/− iBMDMs
+ GFP-IRF3
+HA.STING + GFP-IRF3
+HA.STING S365A + GFP-IRF3
+HA.STING L373A + GFP-IRF3
DMXAA (45 min): – + – + – + – +
WCL: P-STINGSer365
37 kDa
WCL: STING
37 kDa
WCL: P-TBK1Ser172
75 kDa
WCL: P-IKKεSer172
75 kDa
WCL: IRF3
75 kDa
GFP-IRF3
WCL: HSP70
75 kDa

 © The Author(s)

Figure EV4. The role of IRF3 in non-IFN STING responses is independent of its canonical function as a transcription factor for IFNs.

(**A**) Wild-type (WT) iBMDMs, IRF3^{KO} iBMDMs and those expressing either GFP-IRF3 or GFP-IRF3 S396A were lysed for immunoblot with the indicated antibodies. Data shown is representative of three biological experiments. (**B**) IRF3^{KO} iBMDMs expressing GFP-IRF3 or GFP-IRF3 S396A were left untreated (UT) or stimulated with 50 µg/mL DMXAA for 4 h before IRF3 localisation was examined by fluorescence microscopy. Scale bar = 40 µm. Data shown is representative of three biological experiments. (**C**) IRF3^{KO} iBMDMs expressing GFP-IRF3 or GFP-IRF3 S396A were left untreated (–) or stimulated for 45 min with 50 µg/mL DMXAA (+). Cells were lysed and a portion of the whole cell lysate (WCL) underwent immunoblot with the indicated antibodies. The remaining lysate underwent GFP immunoprecipitation (IP) before immunoblotting with the indicated antibodies. Data representative of 2 biological experiments. (**D**) Multiple sequence alignment of part of the CTT from human (green), murine (pink), rat (black), pig (black) and bovine (black) STING. Conserved phospho-serine (S) residues are highlighted in blue, the conserved lysine (L) residue at position 374 (human STING) is highlighted in red, the threonine (T) residue at position 376 (human STING) previously identified to be phosphorylated is highlighted in black (serine residues in pig and bovine). Red line indicates the TBK1/IKKε binding motif (BM); orange line indicates the IRF3 BM; grey line indicates the IRF3 binding interface. (**E**) *Sting1*$^{-/-}$ iBMDMs expressing GFP-IRF3 as well as either HA.STING, HA.STING S365A or HA.STING L373A were left untreated (–) or stimulated for 45 min with 50 µg/mL DMXAA (+). Cells were lysed and a portion of the whole cell lysate (WCL) underwent immunoblot with the indicated antibodies. Data representative of three biological experiments.

© The Author(s)

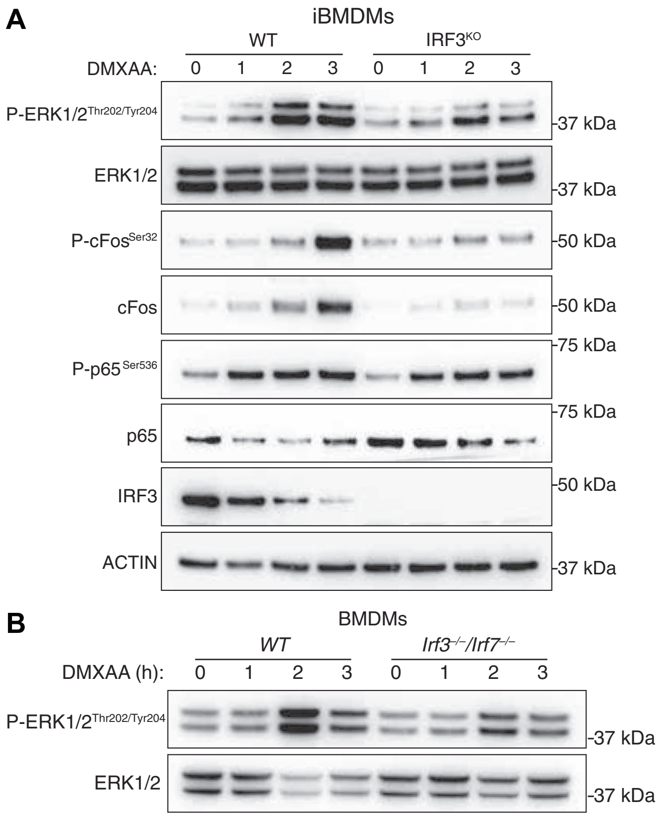

Figure EV5. Loss of IRF3 affects ERK1/2 and cFOS activity.

(**A**) Wild-type (WT) and IRF3^{KO} iBMDMs were left untreated (0) or stimulated for 1, 2 or 3 h with 50 μg/mL DMXAA before cells were lysed for immunoblot with the indicated antibodies. Data shown is representative of two biological experiments. (**B**) Wild-type (WT) and *Irf3*$^{-/-}$/*Irf7*$^{-/-}$ primary BMDMs were left untreated (0) or stimulated for 1, 2 and 3 h with 50 μg/mL DMXAA. Cells were then lysed for immunoblot with the indicated antibodies. Data shown is representative of three biological experiments.

© The Author(s)
