# Supplementary material for: Noncanonical IRF3 function mediates STING-dependent pro-inflammatory cytokine production in macrophages
Source: EMBO Rep. 2026 May 8;27(11):2865–92. doi: 10.1038/s44319-026-00793-6 (PMC13260819; doi:10.1038/s44319-026-00793-6)
Supplement: Supplementary file 1 — Peer Review File [file 44319_2026_793_MOESM1_ESM.pdf]

EMBO reports

Noncanonical IRF3 function mediates STING-dependent pro-inflammatory cytokine production in macrophages

Katherine Balka, Olivia Lamb, Rajan Venkatraman, Zoe Magill, Le Tri Hoang, Ryker Dumbrill, Kate McArthur, Nicole de Weerd, Paul Hertzog, Peter Crack, Linda Wakim, Kim Good-Jacobson, Mireille LAHOUD, Meredith O'Keeffe, and Dominic De Nardo

Corresponding author(s): Dominic De Nardo (dominic.denardo@monash.edu)

Review Timeline:

Submission Date:	5th Aug 25
Editorial Decision:	15th Aug 25
Revision Received:	12th Mar 26
Editorial Decision:	15th Apr 26
Revision Received:	20th Apr 26
Accepted:	21st Apr 26

Editor: Martina Rembold / Achim Breiling

Transaction Report:

(Note: With the exception of the correction of typographical or spelling errors that could be a source of ambiguity, letters and reports are not edited. Depending on transfer agreements, referee reports obtained elsewhere may or may not be included in this compilation. Referee reports are anonymous unless the Referee chooses to sign their reports.)

1st Editorial Decision **15th Aug 2025**

Dear Dr. De Nardo,

We have now received the reports from the three referees that were asked to evaluate your study, which can be found at the end of this email. As Martina is traveling, I took over the manuscript for the time being.

As you will see, the referees think that these findings are of interest. However, they have several comments, concerns, and suggestions, indicating that a major revision of the manuscript is necessary to allow publication of the study in EMBO reports. As the reports are below, and all the referee concerns need to be addressed, I will not detail them here. It seems central though, as indicated by referees #1 and #2, to add data that rule out IFN-I-mediated effects in the present study.

Given the constructive referee comments, I would thus like to invite you to revise your manuscript with the understanding that the concerns of the referees must be addressed in the revised manuscript and/or in a detailed point-by-point response. Acceptance of your manuscript will depend on a positive outcome of a second round of review. It is EMBO reports policy to allow a single round of revision only and acceptance of the manuscript will therefore depend on the completeness of your responses included in the next, final version of the manuscript.

Revised manuscripts should be submitted within three months of a request for revision. Please contact me to discuss the revision (also by video chat) if you have questions or comments regarding the revision, or should you need additional time.

When submitting your revised manuscript, please also carefully review the instructions that follow below.

PLEASE NOTE THAT upon resubmission revised manuscripts are subjected to an initial quality control prior to exposition to re-review. Upon failure in the initial quality control, the manuscripts are sent back to the authors, which may lead to delays. Frequent reasons for such a failure are the lack of the data availability section (please see below) and the presence of statistics based on n=2 (the authors are then asked to present scatter plots or provide more data points).

When submitting your revised manuscript, we will require:

1) a .docx formatted version of the final manuscript text (including legends for main figures, EV figures and tables), but without the figures included. Figure legends should be compiled at the end of the manuscript text.

2) individual production quality figure files as .eps, .tif, .jpg (one file per figure), of main figures and EV figures. Please upload these as separate, individual files upon re-submission.

The Expanded View format, which will be displayed in the main HTML of the paper in a collapsible format, has replaced the Supplementary information. For a report you can submit up to 5 main figures and up to 5 as Expanded View. Please follow the nomenclature Figure EV1, Figure EV2 etc. The figure legend for these should be included in the main manuscript document file in a section called Expanded View Figure Legends after the main Figure Legends section. Additional Supplementary material should be supplied as a single pdf file labeled Appendix. The Appendix should have page numbers and needs to include a table of content on the first page (with page numbers) and legends for all content. Please follow the nomenclature Appendix Figure Sx, Appendix Table Sx etc. throughout the text, and also label the figures and tables according to this nomenclature.

For more details, please refer to our guide to authors:
http://www.embopress.org/page/journal/14693178/authorguide#manuscriptpreparation

Please consult our guide for figure preparation:
http://wol-prod-cdn.literatumonline.com/pb-assets/embo-site/EMBOPress_Figure_Guidelines_061115-1561436025777.pdf

See also the guidelines for figure legend preparation:
https://www.embopress.org/page/journal/14693178/authorguide#figureformat

3) a .docx formatted letter INCLUDING the reviewers' reports and your detailed point-by-point responses to their comments. As part of the EMBO Press transparent editorial process, the point-by-point response is part of the Review Process File (RPF), which will be published alongside your paper.

4) a complete author checklist, which you can download from our author guidelines (https://www.embopress.org/page/journal/14693178/authorguide). Please insert page numbers in the checklist to indicate where the requested information can be found in the manuscript. The completed author checklist will also be part of the RPF.

Please also follow our guidelines for the use of living organisms, and the respective reporting guidelines:
http://www.embopress.org/page/journal/14693178/authorguide#livingorganisms

5) that primary datasets produced in this study (e.g. RNA-seq, ChIP-seq, structural and array data) are deposited in an appropriate public database. If no primary datasets have been deposited, please also state this in a dedicated section (e.g. 'No primary datasets have been generated and deposited'), see below.

See also: http://embor.embopress.org/authorguide#datadeposition

Please remember to provide a reviewer password if the datasets are not yet public.

The accession numbers and database should be listed in a formal "Data Availability" section that follows the model below. This is now mandatory (like the COI statement). Please note that the Data Availability Section is restricted to new primary data that are part of this study. This section is mandatory. As indicated above, if no primary datasets have been deposited, please state this in this section

Data availability

The datasets produced in this study are available in the following databases:

- RNA-Seq data: Gene Expression Omnibus GSE46843 (https://www.ncbi.nlm.nih.gov/geo/query/acc.cgi?acc=GSE46843)
- [data type]: [name of the resource] [accession number/identifier/doi] ([URL or identifiers.org/DATABASE:ACCESSION])

*** Note - All links should resolve to a page where the data can be accessed. ***

Moreover, I have these editorial requests:

6) We now request the publication of original source data with the aim of making primary data more accessible and transparent to the reader. You will receive a separate email with instructions for providing source data with your revised manuscript, including information how to upload and organize the files.

7) Our journal encourages inclusion of *data citations in the reference list* to directly cite datasets that were re-used and obtained from public databases. Data citations in the article text are distinct from normal bibliographical citations and should directly link to the database records from which the data can be accessed. In the main text, data citations are formatted as follows: "Data ref: Smith et al, 2001" or "Data ref: NCBI Sequence Read Archive PRJNA342805, 2017". In the Reference list, data citations must be labeled with "[DATASET]". A data reference must provide the database name, accession number/identifiers and a resolvable link to the landing page from which the data can be accessed at the end of the reference. Further instructions are available at: http://www.embopress.org/page/journal/14693178/authorguide#referencesformat

8) Regarding data quantification and statistics, please make sure that the number "n" for how many independent experiments were performed, their nature (biological versus technical replicates), the bars and error bars (e.g. SEM, SD) and the test used to calculate p-values is indicated in the respective figure legends (also for EV and Appendix figures). Please also check that all the p-values are explained in the legend, and that these fit to those shown in the figure. Please provide statistical testing where applicable. Please avoid the phrase 'independent experiment', but clearly state if these were biological or technical replicates. Please also indicate (e.g. with n.s.) if testing was performed, but the differences are not significant. In case n=2, please show the data as separate datapoints without error bars and statistics. See also:
http://www.embopress.org/page/journal/14693178/authorguide#statisticalanalysis

If n<5, please show single datapoints for diagrams. Please add to each legend (main, and EV figures, where applicable) a 'Data Information' section (or name the provided section like this) explaining the statistics used or providing information regarding replicates and scales. See:

https://www.embopress.org/page/journal/14693178/authorguide#figureformat

9) Please add scale bars of similar style and thickness to all microscopic images, using clearly visible black or white bars (depending on the background). Please place these in the lower right corner of the images themselves. Please do not write on or near the bars in the image but define the size in the respective figure legend.

10) Please also note our reference format:
http://www.embopress.org/page/journal/14693178/authorguide#referencesformat

11) We updated our journal's competing interests policy in January 2022 and request authors to consider both actual and perceived competing interests. Please review the policy https://www.embopress.org/competing-interests and update your competing interests if necessary. Please name this section 'Disclosure and Competing Interests Statement' and put it after the Acknowledgements section.

12) We now use CRediT to specify the contributions of each author in the journal submission system. CRediT replaces the

author contribution section. Please use the free text box to provide more detailed descriptions and do NOT provide your final manuscript text file with an author contributions section. See also our guide to authors: https://www.embopress.org/page/journal/14693178/authorguide#authorshipguidelines

13) All Materials and Methods need to be described in the main text using our 'Structured Methods' format, which is required for all research articles. According to this format, the Methods section should include a Reagents and Tools Table (listing key reagents, experimental models, software, and relevant equipment and including their sources and relevant identifiers), uploaded as separate file, and a Methods section in which we encourage the authors to describe their methods using a step-by-step protocol format with bullet points, to facilitate the adoption of the methodologies across labs. More information on how to adhere to this format as well as downloadable templates (.doc) for the Reagents and Tools Table can be found in our author guidelines (section 'Structured Methods'):

https://www.embopress.org/page/journal/14693178/authorguide#manuscriptpreparation

14) For a Scientific Report we require that results and discussion sections are combined in a single chapter called "Results & Discussion". Please do this for your manuscript. For more details please refer to our guide to authors: http://www.embopress.org/page/journal/14693178/authorguide#researcharticleguide

Then please order the manuscript sections like this, using only these names:
Title page - Abstract - Keywords - Introduction - Results & Discussion - Methods - Data availability section - Acknowledgements (please put here all the funding information) - Disclosure and Competing Interests Statement - References - Figure legends - Expanded View Figure legends

15) Please make sure that all the funding information is also entered into the online submission system and that it is complete and similar to the one in the acknowledgement section of the manuscript text file.

We would also welcome the submission of cover suggestions, or motifs to be used by our Graphics Illustrator in designing a cover.

As part of the EMBO publication's Transparent Editorial Process, EMBO reports publishes online a Review Process File (RPF) to accompany accepted manuscripts. This File will be published in conjunction with your paper and will include the referee reports, your point-by-point response and all pertinent correspondence relating to the manuscript.

You are able to opt out of this by letting the editorial office know (emboreports@embo.org). If you do opt out, the Review Process File link will point to the following statement: "No Review Process File is available with this article, as the authors have chosen not to make the review process public in this case."

I look forward to seeing a revised form of your manuscript when it is ready.

Achim Breiling
Senior Editor
EMBO Reports

Referee #1:

In this manuscript, Katherine R. Balka et al. report that IRF3 mediates STING-dependent pro-inflammatory cytokine production through a noncanonical mechanism. The study proposes that IRF3 interacts with STING independently of TBK1, mediated by STING S365 phosphorylation, thereby revealing an IRF3 function distinct from IFN signaling and NF-κB activation. However, a series of recent studies demonstrate that IRF3 inhibits inflammatory signaling in macrophages during viral infection (doi:10.1126/sciadv.adn2858; doi:10.3390/v15071579; doi:10.1073/pnas.2121385119), which partially diminishes the novelty of the current findings. Additionally, the study's logical framework requires strengthening. The following major and minor concerns should be addressed to enhance the rigor and impact of the work.

Major comments:

1. Previous studies have constructed a global IRF3 knockout mouse model (Irf3-/-) using genetic engineering techniques, and also has confirmed that IRF3 expression can be completely absent in all cells (doi: 10.1016/j.immuni.2016.04.009, doi: 10.1126/sciadv.adn2858). Why the authors used the Irf3-/-/Irf7-/- mice to explore the role of IRF3 in inflammatory cytokine production, even using the cells from Irf7-/- mice to exclude NEK7 effect? Why not directly use Irf3-/- mice to examine the role of

IRF3 in the study?
2. Previous studies have reported that non-viral stimuli, such as cGAMP and LPS suppress inflammatory target genes in Irf3−/− iBMDMs, while Pam3csk4 does not (doi: 10.1073/pnas.2121385119). It seems different from the findings in this study. The authors should explain or discuss this issue.
3. The repression of IRF3-mediated NF-κB activity (RIKA) attenuates viral activation of NF-κB and the resultant inflammatory gene induction. The authors should exclude the effect of RIKA to make the findings more convincing.
4. We strongly advice the authors examine the noncanonical role in pro-inflammatory cytokine production using the Ifnar-deficient mice or cells to eliminate the IFN signaling functions.
5. The authors showed that IRF3 interacted with STING in this study. The domains mediating IRF3-STING binding, particularly whether IRF3 interacts with the STING C-terminal tail (containing phosphorylated S365/S366), should be specified.
6. STING-deficient iBMDMs were used to analyze STING mutants, but simultaneous TBK1 deficiency is necessary to conclusively explain IRF3-STING-IKKε association in the absence of TBK1.
7. Line184, why the authors propose the IRF3-STING interaction is involved in alternative serine phospho-sites in the STING CTT. The authors should explain it.

Minor comments:
1. Abstract section should be brief as well as efficient.
2. Why the unit of IFN-β secretion in several figures is U/ml, not pg/ml ?
3. Please label non-significant (ns) data in figures where statistical tests were applied.
4. Fig. 2E, the phosphorylation of p65 also occur in the untreated cells, which may make the data not convincing enough. The authors should pay attention to this point.
5. Fig. 3A and EV Figure 3, the images of GFP-IRF3 obtained via immunofluorescence seemed black and white, which can not reflect the location of IRF3. We strongly advise to use green fluorescence. Besides, the images should be more clear and distinct.
6. EV Figure 4: Remove data from previous studies unless essential; irrelevant reuse may confuse readers.
7. Line163, the description for EV Figure 3A-B is not consistent with the figure, as there is no A or B in the corresponding figure.
8. Define all abbreviations at first use (e.g., "dendritic cells (DCs)" at Line 118).
9. There are numerous grammatical mistakes in the manuscript ("iMEF" → "iMEFs" (Lines 125, 366); "PamCys" → "Pam3CSK4" (Line 206). Typos: "Student's t-test" (Lines 163, 450-451; remove hyphen). We encourage the authors to have the text edited to resolve pervasive grammatical errors.

Referee #2:

Summary
In this study, an unexpected requirement for IRF3 is reported for the induction of pro-inflammatory cytokines downstream of STING activation. Beyond its canonical role in type I interferon (IFN-I) induction, the authors propose that STING-mediated recruitment and activation of IRF3 is also essential for the expression of NF-κB target genes such as TNFα and IL-6 in bone marrow-derived macrophages (BMDMs). It's been further speculated that IRF3 may have an additional scaffolding function in promoting STING-induced NF-κB responses.

While the work addresses an interesting and mechanistically important question, a key conceptual caveat undermines the central conclusion. In its current form, the data do not definitively support an IFN-I-independent role for IRF3 in NF-κB-dependent gene expression.

Wu et al. (2020) demonstrated, using IFNAR1-/- BMDMs, that TNFα and IL-6 expression downstream of STING activation is actually largely dependent on IFNs-I. In that work, cGAMP-induced TNFα and IL-6 expression was strongly reduced in IFNAR1-/- cells, suggesting an indirect requirement via IFN-I signaling. A similar relationship exists for nucleic acid-sensing TLRs, where IRF5 activation is also cooperating with NF-κB for TNFα/IL-6 induction downstream of TLR7/9 stimulation (PMID: 39856058).

1. To rule out IFN-I-mediated effects in the present study, the authors should perform stimulation assays in WT BMDMs treated with DMXAA or cGAMP in the presence of IFNAR-blocking antibodies. If TNFα/IL-6 induction would be reduced (similarly to Wu et al.), then IFN-I signaling contributes to the expression of these cytokines. Efficient IFNAR blocking should be confirmed by absence of ISG induction.

2. Additionally, the authors should measure expression of other pro-inflammatory, IFN-I-independent genes-such as IL-12p40 or IL-1α (per Wu et al.)-to see whether these are also reduced in IRF3-deficient cells.

The current control exp that authors provided in Fig 2C to rule out an IFN-dependence does not fully address these concerns. In Fig 2C, the authors attempt to complement IRF3 KO cells with exogenous IFNβ/α. However, the 16-hour gap between IFN-I addition and STING stimulation does not effectively test for direct IFN-dependence in TNFα/IL-6 transcription. The temporal mismatch likely fails to mimic endogenous IFN-I production kinetics and does not exclude IFN-I-mediated effects.

Without these additional experiments, the main mechanistic claim-that IRF3 directly promotes NF-κB gene expression independent of IFN-I-remains unproven. As such, the current evidence is insufficient for publication in EMBO reports.

Minor comments:
• Choice of knockout model: The authors use IRF3/IRF7 double KO BMDMs but then perform extensive controls to rule out a contribution of IRF7. Why not use IRF3 KO macrophages directly for a cleaner genetic system?
• Fig. 2D: The IκBα blot in IRF3/7 KO BMDMs shows reduced degradation compared to WT. This point is glossed over but may be biologically relevant. Quantification across the 3 independent repeats should be provided. Furthermore, has NF-κB nuclear translocation been assessed in these cells?
• Fig. 3: Signaling data for TBK1 KO is missing. It would be important to know whether TBK1 KO cells phenocopy the TNFα/IL-6 defect seen in IRF3/7 KO cells.
• Fig. 4B: The authors should include co-immunoprecipitation data of IRF3 with various STING mutants (e.g., S365A, L373A, S354A, S357A) to validate the proposed recruitment model. This would provide valuable cross-validation and mechanistic depth.
• Discussion: The rationale for concluding a scaffolding function of IRF3 is unclear. This hypothesis needs to be better justified based on the presented data.

Typos:
- Line 139: remove "any" or "a"
- Line 182: remove "via"
- Line 192: include "S357A"
- Line 283: replace "indispensible" with "indispensable"

Overall assessment:
This work addresses an interesting mechanistic question about STING-IRF3-NF-κB crosstalk. However, without rigorous exclusion of IFN-I-dependent mechanisms, the main conclusion is premature. Addressing the above points-particularly the IFNAR-blockade experiments and inclusion of IFN-I-independent cytokine readouts-will be essential for the manuscript to meet the conceptual and experimental standards of EMBO reports.

Referee #3:

In this manuscript, the authors provided the clear evidence of non-canonical IRF3 function mediating STING-dependent pro-inflammatory cytokine production in macrophages. Although several papers have already addressed the role of IRF3 in the production of pro-inflammatory cytokines, this study is distinct in showing that IRF3 can be associated with STING in the absence of TBK1 (Figure 3). This finding is important to open the new way to understand the non-canonical role of IRF3. The experiments/results are overall robust and compelling. The manuscript is well-written and cites proper publications.

Comments:
(1) Since IRF3 appears dispensable for pro-inflammatory cytokine production in dendritic cells, the title should be revised in that way. I propose adding "in macrophage" to the title.

(2) STING dimer can be multimerized in the trans-Golgi network (PMID: 38212328), which may be linked to non-canonical IRF3 function as scaffold. This paper can be included in "Discussion".

(3) line 150: "maker" should read "marker".

EMBOR-2025-62361V3_Response to reviewers

Editor comments are shown in *italics* and our responses are shown in blue.

We thank the reviewers for their time and helpful feedback on our manuscript. We believe we have addressed all the major concerns and in doing so greatly improved the quality of our manuscript and the impact of the findings.

In particular, we addressed the two major concerns of Reviewers #1 and #2:

i) use of single IRF3 KO macrophages, confirming the phenotype is IRF3 dependent;

ii) examined the effects of IFNs using an IFNAR1 blocking antibody and IFNAR1 and IFNAR KO macrophages, the results of which strongly suggest the phenotype of IRF3-deficient cells is independent of changes in global IFN levels.

During the revision of our manuscript, we made additional key discoveries that provide further mechanistic insight:

- Loss of IRF3 does not impact NF-κB nuclear translocation (new **Fig.2G**);
- The role of IRF3 is independent of its phosphorylation at S396A and therefore its dimerization and nuclear translocation (new **Fig.3E-F** and **Fig.EV4A-C**);
- IRF5 does not play a role in STING-mediated pro-inflammatory cytokine production (new **Fig.5A-B**);
- IRF3 mediates the activation of a STING-induced Erk1/2–cFos signalling arm (new **Fig.5A-B**).

Of note, during the preparation of our revised manuscript a new research paper was published in *Nature Immunology* from the laboratory of Søren Paludan that strongly supports our general findings (Zhang *et al*, 2025 – PMID: 40973797). While some mechanistic details differ between the studies, the major finding that IRF3 plays a central and direct role in STING-mediated pro-inflammatory cytokine responses independently of type I IFN signalling is clearly consistent. This complementary work supports the validity and timely nature of our study.

Referee #1:

In this manuscript, Katherine R. Balka et al. report that IRF3 mediates STING-dependent pro-inflammatory cytokine production through a noncanonical mechanism. The study proposes

that IRF3 interacts with STING independently of TBK1, mediated by STING S365 phosphorylation, thereby revealing an IRF3 function distinct from IFN signaling and NF-κB activation. However, a series of recent studies demonstrate that IRF3 inhibits inflammatory signaling in macrophages during viral infection (doi:10.1126/sciadv.adn2858; doi:10.3390/v15071579; doi:10.1073/pnas.2121385119), which partially diminishes the novelty of the current findings. Additionally, the study's logical framework requires strengthening. The following major and minor concerns should be addressed to enhance the rigor and impact of the work.

Major comments:

1. *Previous studies have constructed a global IRF3 knockout mouse model (Irf3-/-) using genetic engineering techniques, and also has confirmed that IRF3 expression can be completely absent in all cells (doi: 10.1016/j.immuni.2016.04.009, doi: 10.1126/sciadv.adn2858). Why the authors used the Irf3-/-/Irf7-/- mice to explore the role of IRF3 in inflammatory cytokine production, even using the cells from Irf7-/- mice to exclude NEK7 effect? Why not directly use Irf3-/- mice to examine the role of IRF3 in the study?*

We entirely agree with the reviewer and thank them for this important suggestion. To this end, we generated IRF3-deficient immortalised BMDMs ($IRF3^{KO}$ iBMDMs) using CRISPR/Cas9 gene editing (**Fig. EV1Q**). Consistent with our data from primary cells and iMEFs, we observed that the loss of IRF3 from macrophages was sufficient to reduce STING-induced IFNβ (**Fig.1H**), as well as TNF cytokine secretion (**Fig.1I**). While STING-induced TNF production was significantly reduced in $IRF3^{KO}$ iBMDMs, TNF levels were comparable to those from WT iBMDMs in response to TLR1/2 activation with Pam3Cysk4 (P3C) (**Fig.1I**). In addition, the $IRF3^{KO}$ iBMDMs were utilised to generate the following new data: **Fig.2F-G, Fig.3E-F, Fig.5A-D, Fig.EV2A-B, Fig.EV3A, Fig.EV4A-C** and **Fig.EV5A**. The phenotype we observed in our $IRF3^{KO}$ iBMDMs is consistent with observations made in the recent publication by Zhang *et al*. where the authors used primary murine IRF3-deficient macrophages as well as several human IRF3 KO cell lines (Zhang *et al*, 2025 – PMID: 40973797).

2. *Previous studies have reported that non-viral stimuli, such as cGAMP and LPS suppress inflammatory target genes in Irf3−/− iBMDMs, while Pam3csk4 does not (doi: 10.1073/pnas.2121385119). It seems different from the findings in this study. The authors should explain or discuss this issue.*

We appreciate the reviewer's suggestion to consider the potential contribution of RIKA to the observed phenotype. Our study focuses on STING agonist–driven signalling in macrophages, and not on viral activation of NF-κB, which is the context in which RIKA has previously been described to operate. Importantly, the defect we observe is selective for STING activation and is not seen following stimulation of multiple TLR pathways, arguing against a general repression of NF-κB activity that would be expected if RIKA were broadly working in this model.

3. *The repression of IRF3-mediated NF-κB activity (RIKA) attenuates viral activation of NF-κB and the resultant inflammatory gene induction. The authors should exclude the effect of RIKA to make the findings more convincing.*

While we appreciate the suggestion, given that our data argue against a role for RIKA (see **Reviewer #1, point 2**), experimentally excluding RIKA would not provide additional mechanistic insight.

4. *We strongly advice the authors examine the noncanonical role in pro-inflammatory cytokine production using the Ifnar-deficient mice or cells to eliminate the IFN signaling functions.*

We thank the reviewer for this important suggestion. To further examine the role of IFN on our observed phenotype we took two main approaches: *i)* use of an IFNAR1 blocking antibody; and, *ii)* generation of IFNAR1^{KO} and IFNAR2 ^{KO} iBMDMs.

Firstly, we treated primary BMDMs with a well characterised interferon-α/β receptor (IFNAR)1 blocking antibody or an isotype control counterpart prior to activation of STING. In the presence of IFNAR1 blocking we observed no reduction in STING- nor TLR2-induced TNF secretion (**Fig.2D**). We further observed no differences in DMXAA-induced STING signalling responses with IFNAR1 blocking antibody treatment (**Fig.EV2B**). Importantly, we saw that IFNα-induced STAT1 phosphorylation and ISG induction (shown by IRF7 expression) were completely abolished by IFNAR1 blocking antibody treatment, demonstrating efficient inhibition of type I IFN responses under these conditions (**Fig.EV2C-D**).

Secondly, we examined the role of IFN signalling genetically, by deleting either IFNAR1 or IFNAR2 in iBMDMs. The efficacy of gene deletion was indicated by an absence of both STAT1 phosphorylation and IRF7 upregulation in response to IFNα stimulation from both IFNAR1^{KO} and IFNAR2^{KO} iBMDMs (**Fig.EV2E**). While we observed no differences in STING signalling responses between WT, IFNAR1^{KO} and IFNAR2 ^{KO} iBMDMs (**Fig.EV2F**), we observed that

iBMDMs lacking either IFNAR1 or IFNAR2 displayed small yet significant reductions in STING-induced TNF production (**Fig.2E**). Of note, significant reductions were also observed in basal TNF levels and TNF induced by TLR1/2 activation (**Fig.2E**). This differs to our findings from both IRF3^{KO} iBMDMs and primary BMDMs activated in the presence of IFNAR1 blocking antibody, where TLR1/2-induced TNF remained unchanged (**Fig.1I** and **Fig.2D**). This strongly suggests that the reductions seen in PRR-driven TNF are likely due to intrinsic differences in cells lacking IFNARs, which are known to have altered macrophage homeostasis and immune responses (Gough *et al*, 2012 – PMID: 22365663).

Together these data therefore suggest that the role of IRF3 in STING-induced cytokine production is independent of any effects on global IFN responses.

5. *The authors showed that IRF3 interacted with STING in this study. The domains mediating IRF3-STING binding, particularly whether IRF3 interacts with the STING C-terminal tail (containing phosphorylated S365/S366), should be specified.*

As stated in the original manuscript and displayed in **Fig.EV4D** (previously Fig.4A), in addition to S365 (366 in human), residues S354 (S355 in human) and S357 (S358 in human) are located within the described IRF3 binding interface (Zhao *et al*, 2016 – PMID: 27302953) and are highly conserved in mammals.

6. *STING-deficient iBMDMs were used to analyze STING mutants, but simultaneous TBK1 deficiency is necessary to conclusively explain IRF3-STING-IKKε association in the absence of TBK1.*

We have previously published that TBK1 and IKKε can redundantly mediate non-IFN STING responses (Balka et al, 2020 – PMID: 32268090; and Venkatraman et al, 2024 – PMID: 39262777). Here, we show that in the absence of TBK1, IRF3 can still form a stable signalling complex with STING and IKKε (**Fig.3D**). Furthermore, in new data we demonstrate that the STING complex (STING–TBK1–IKKε–IRF3) only forms in cells expressing WT STING and not STING S365A (**Fig.4D**). Hence, we don't believe that removing TBK1 from these cell models will provide any further mechanistic insights.

7. *Line184, why the authors propose the IRF3-STING interaction is involved in alternative serine phospho-sites in the STING CTT. The authors should explain it.*

We and others have identified several additional residues to S365 within the STING CTT that become phosphorylated upon activation (Balka *et al.*, 2023 – PMID: 37139896; Konno *et al*, 2013 – PMID: 24119841). As the known IRF3–STING binding interface is known to contain two other serine phospho-sites i.e. S354 and S357, we hypothesised that perhaps one of these may be more important for this noncanonical role of IRF3. However, as the data in Fig.4 demonstrates, IRF3 recruitment to STING is indeed primarily mediated via the phosphorylation of S365, which is subsequently crucial for driving type I IFNs responses as well as mediating the production of pro-inflammatory cytokines.

Minor comments:

1. *Abstract section should be brief as well as efficient.*

The abstract is in line with the requirements for EMBO Reports.

2. *Why the unit of IFN-β secretion in several figures is U/ml, not pg/ml ?*

We apologise for the lack of clarity about this in the original manuscript. The IFN-β concentrations are measured in U/mL for ELISA measurements and in pg/mL for Legendplex assay measurements, consistent with the specific standards utilised for each assay. This has now been explicitly stated in the Methods of the revised manuscript. As these units reflect different measurements, the values obtained from ELISA and Legendplex assays were not directly compared.

3. *Please label non-significant (ns) data in figures where statistical tests were applied.*

We thank the reviewer for pointing this out and have now added 'ns' symbols to all data analysed. In addition, we have added symbols to data where the protein levels were 'not determined' (ND).

4. *Fig. 2E, the phosphorylation of p65 also occur in the untreated cells, which may make the data not convincing enough. The authors should pay attention to this point.*

Many cell types, including MEFs, monocytes and macrophages have basal signalling that mediates their survival, proliferation and maturation. These homeostatic responses are mediated via continuous low-level activation of MAPKs and NF-κB pathways, amongst others. Hence, it is normal to observe some basal activation by western blot in unstimulated cells.

This is also why these basal phosphorylation events are observed in STING KO macrophages (**Fig.4A**).

5. *Fig. 3A and EV Figure 3, the images of GFP-IRF3 obtained via immunofluorescence seemed black and white, which can not reflect the location of IRF3. We strongly advise to use green fluorescence. Besides, the images should be more clear and distinct.*

We understand the reviewers concern, however the images displayed are in fact of GFP-IRF3. In fluorescence microscopy, images are acquired as single-channel intensity (grayscale) data, which can subsequently be displayed using pseudocolouring. As white on black has the highest perceived contrast for the human eye, we chose to display the images as greyscale, which is considered best practice, rather than use green pseudocolour. Indeed, "*When only one fluorophore/wavelength/channel is shown in a figure, grayscale, which has the best contrast, is favourable*" (Schmied & Jambor, 2020 – PMID: 33708381). The GFP images were obtained using an EVOS™ XL Core Imaging System with the EVOS GFP Light Cube (Ex: 470/22 nm; Em: 510/42 nm). As this is a widefield microscope, in each image the entire field of view is illuminated at once. Hence, what is seen is the sum of all planes and this leads to reduced resolution compared to some other imaging approaches, such as confocal microscopy. In this case, to assess if GFP-IRF3 is either in the cytoplasm or nucleus the resolution is entirely sufficient.

6. *EV Figure 4: Remove data from previous studies unless essential; irrelevant reuse may confuse readers.*

We entirely agree that the addition of this data may be confusing and have now removed it from the revised manuscript.

7. *Line163, the description for EV Figure 3A-B is not consistent with the figure, as there is no A or B in the corresponding figure.*

This has now been corrected, thank you.

8. *Define all abbreviations at first use (e.g., "dendritic cells (DCs)" at Line 118).*

This has been done. Of note, the first use of dendritic cells (DCs) was in fact in the introduction of the manuscript (line 83).

9. *There are numerous grammatical mistakes in the manuscript ("iMEF" → "iMEFs" (Lines 125, 366); "PamCys" → "Pam3CSK4" (Line 206). Typos: "Student's t-test" (Lines 163, 450-451; remove hyphen). We encourage the authors to have the text edited to resolve pervasive grammatical errors.*

We thank the reviewing for pointing out these oversights. These errors have now been rectified. In the revised manuscript

----

Referee #2:

Summary

In this study, an unexpected requirement for IRF3 is reported for the induction of pro-inflammatory cytokines downstream of STING activation. Beyond its canonical role in type I interferon (IFN-I) induction, the authors propose that STING-mediated recruitment and activation of IRF3 is also essential for the expression of NF-κB target genes such as TNFα and IL-6 in bone marrow-derived macrophages (BMDMs). It's been further speculated that IRF3 may have an additional scaffolding function in promoting STING-induced NF-κB responses.

While the work addresses an interesting and mechanistically important question, a key conceptual caveat undermines the central conclusion. In its current form, the data do not definitively support an IFN-I-independent role for IRF3 in NF-κB-dependent gene expression.

Wu et al. (2020) demonstrated, using IFNAR1-/- BMDMs, that TNFα and IL-6 expression downstream of STING activation is actually largely dependent on IFNs-I. In that work, cGAMP-induced TNFα and IL-6 expression was strongly reduced in IFNAR1-/- cells, suggesting an indirect requirement via IFN-I signaling. A similar relationship exists for nucleic acid-sensing TLRs, where IRF5 activation is also cooperating with NF-κB for TNFα/IL-6 induction downstream of TLR7/9 stimulation (PMID: 39856058).

1. *To rule out IFN-I-mediated effects in the present study, the authors should perform stimulation assays in WT BMDMs treated with DMXAA or cGAMP in the presence of IFNAR-blocking antibodies. If TNFα/IL-6 induction would be reduced (similarly to Wu et al.), then IFN-I signaling contributes to the expression of these cytokines. Efficient IFNAR blocking should be confirmed by absence of ISG induction.*

We thank the reviewer for this important suggestion. To further examine the role of IFN on our observed phenotype we took two main approaches: *i)* use of an IFNAR1 blocking antibody; and, *ii)* generation of IFNAR1^{KO} and IFNAR2 ^{KO} iBMDMs.

Firstly, we treated primary BMDMs with a well characterised interferon-α/β receptor (IFNAR)1 blocking antibody or an isotype control counterpart prior to activation of STING. In the presence of IFNAR1 blocking we observed no reduction in STING- nor TLR2-induced TNF secretion (**Fig.2D**). We further observed no differences in DMXAA-induced STING signalling responses with IFNAR1 blocking antibody treatment (**Fig.EV2B**). Importantly, we saw that IFNα-induced STAT1 phosphorylation and ISG induction (shown by IRF7 expression) were completely abolished by IFNAR1 blocking antibody treatment, demonstrating efficient inhibition of type I IFN responses under these conditions (**Fig.EV2C-D**).

Secondly, we examined the role of IFN signalling genetically, by deleting either IFNAR1 or IFNAR2 in iBMDMs. The efficacy of gene deletion was indicated by an absence of both STAT1 phosphorylation and IRF7 upregulation in response to IFNα stimulation from both IFNAR1^{KO} and IFNAR2^{KO} iBMDMs (**Fig.EV2E**). While we observed no differences in STING signalling responses between WT, IFNAR1^{KO} and IFNAR2^{KO} iBMDMs (**Fig.EV2F**), we observed that iBMDMs lacking either IFNAR1 or IFNAR2 displayed small yet significant reductions in STING-induced TNF production (**Fig.2E**). Of note, significant reductions were also observed in basal TNF levels and TNF induced by TLR1/2 activation (**Fig.2E**). This differs to our findings from both IRF3^{KO} iBMDMs and primary BMDMs activated in the presence of IFNAR1 blocking antibody, where TLR1/2-induced TNF remained unchanged (**Fig.1I** and **Fig.2D**). This strongly suggests that the reductions seen in PRR-driven TNF are likely due to intrinsic differences in cells lacking IFNARs, which are known to have altered macrophage homeostasis and immune responses (Gough *et al*, 2012 – PMID: 22365663). Important to note is that while in *Wu et al*., the authors observed decreases in STING-induced cytokine expression in IFNAR1-deficient BMDMs, they did not assess cytokine expression in response to activation of any other PRRs. Hence, it is not possible to say if the effect they observed was specific to STING or not.

Together our data suggest that the role of IRF3 in STING-induced cytokine production is independent of any effects on global IFN responses.

***2.** Additionally, the authors should measure expression of other pro-inflammatory, IFN-I-independent genes-such as IL-12p40 or IL-1α (per Wu et al.)-to see whether these are also reduced in IRF3-deficient cells.*

Based on our findings using the IFNAR1 blocking antibody and from IFNAR1^{KO} and IFNAR2^{KO} iBMDMs (see above – **Reviewer #2, point 1**), we don't believe the reductions in STING-induced pro-inflammatory cytokine production from IRF3 KO cells is due to any changes in global IFN levels. Hence, we did not examine the expression of IL-12p40 or IL-1α in this setting.

The current control exp that authors provided in Fig 2C to rule out an IFN-dependence does not fully address these concerns. In Fig 2C, the authors attempt to complement IRF3 KO cells with exogenous IFNβ/α. However, the 16-hour gap between IFN-I addition and STING stimulation does not effectively test for direct IFN-dependence in TNFα/IL-6 transcription. The temporal mismatch likely fails to mimic endogenous IFN-I production kinetics and does not exclude IFN-I-mediated effects.

Without these additional experiments, the main mechanistic claim-that IRF3 directly promotes NF-κB gene expression independent of IFN-I-remains unproven. As such, the current evidence is insufficient for publication in EMBO reports.

Minor comments:

3. *Choice of knockout model: The authors use IRF3/IRF7 double KO BMDMs but then perform extensive controls to rule out a contribution of IRF7. Why not use IRF3 KO macrophages directly for a cleaner genetic system?*

We entirely agree with the reviewer and thank them for this important suggestion. To this end, we generated IRF3-deficient immortalised BMDMs (IRF3^{KO} iBMDMs) using CRISPR/Cas9 gene editing (**Fig. EV1Q**). Consistent with our data from primary cells and iMEFs, we observed that the loss of IRF3 from macrophages was sufficient to reduce STING-induced IFNβ (**Fig.1H**), as well as TNF cytokine secretion (**Fig.1I**). While STING-induced TNF production was significantly reduced in IRF3^{KO} iBMDMs, TNF levels were comparable to those from WT iBMDMs in response to TLR1/2 activation with Pam3Cysk4 (P3C) (**Fig.1I**). In addition, the IRF3^{KO} iBMDMs were utilised to generate the following new data: **Fig.2F-G, Fig.3E-F, Fig.5A-D, Fig.EV2A-B, Fig.EV3A, Fig.EV4A-C** and **Fig.EV5A**. The phenotype we observed in our IRF3^{KO} iBMDMs is consistent with observations made in the recent publication by Zhang *et al*. where the authors used primary murine IRF3-deficient macrophages as well as several human IRF3 KO cell lines (Zhang *et al,* 2025 – PMID: 40973797).

***4.** Fig. 2D: The IκBα blot in IRF3/7 KO BMDMs shows reduced degradation compared to WT. This point is glossed over but may be biologically relevant. Quantification across the 3 independent repeats should be provided.*

We thank the reviewer for this keen observation and agree that the levels of IκBα degradation appear reduced in the experiment presented in **Fig.EV3B** (previously Fig.2D). However, we believe this observation is simply due to a loading error i.e. more protein loaded in the *Irf3*$^{-/-}$/*Irf7*$^{-/-}$ samples. Indeed, we subsequently compared NF-κB translocation between WT and IRF3^{KO} iBMDMs and observed no defects, further suggesting this was not a biological effect due to IRF3 loss (see below – **Reviewer #2, point 5**).

***5.** Furthermore, has NF-κB nuclear translocation been assessed in these cells?*

We thank the reviewer for this insightful suggestion and examined NF-κB nuclear translocation in macrophages lacking IRF3. Consistent with comparable phosphorylation of the NF-κB p65 subunit observed in cell lysates from IRF3^{KO} iBMDMs (**Fig.2F** and **Fig.EV3A**), we observed no difference in the amount of p65 that translocated to the nucleus in IRF3^{KO} iBMDMs following STING activation (**Fig.2G**). Similarly, the nuclear translocation of the NF-κB p50 subunit (generated from the cleavage of p105 in the cytoplasm) also appeared normal in cells lacking IRF3 (**Fig.2G**). These data therefore further support that the role of IRF3 in STING-induced cytokine production is independent of canonical NF-κB signalling.

Our data thus far suggests the role of IRF3 in pro-inflammatory cytokine production downstream of STING is independent of NF-κB activity. IRF5 has previously been shown to cooperate with other transcription factors downstream of nucleic acid sensing TLRs to drive pro-inflammatory cytokines in monocytes and macrophages (Heinz *et al*, 2020 – PMID: 32433612). The role of IRF5 in STING-mediated cytokine production is currently unknown. We therefore generated IRF5^{KO} iBMDMs (**Fig.EV1Q**) and examined TNF and IFNβ secretion from WT, IRF3^{KO} and IRF5^{KO} iBMDMs following STING and TLR7 activation (using R848). Consistent with our previous data, IRF3^{KO} iBMDMs displayed a significant reduction in TNF levels (**Fig.5C**), as well as a complete loss of IFNβ production (**Fig.5D**). In contrast, we observed no reduction in STING-induced TNF nor IFNβ from macrophages lacking IRF5. Importantly, TNF production following TLR7 activation was greatly reduced in IRF5^{KO} iBMDMs, but not those lacking IRF3 further demonstrating IRF5 functional deficiency. This data clearly demonstrates that IRF5 is not involved in cytokine production from macrophages downstream of STING.

Examining the translocation of NF-κB further inspired us to look at other important transcription factors that might be in play in observed phenotype in the absence of IRF3. We have previously found that STING induces the activation of members of the mitogen-activated protein kinase (MAPK) family (Venkatraman *et al.*, 2024 – PMID: 39262777). MAPKs control the activity of components of the activating protein (AP)-1 transcriptional complex (e.g. cFos and cJun) that work in concert with NF-κB to drive pro-inflammatory cytokines. We therefore examined nuclear translocation of cFos and cJun in response to STING activation. Of note, while cJun translocation was comparable between WT and $IRF3^{KO}$ iBMDMs, we observed a clear defect in the ability of cFos to accumulate in the nucleus of $IRF3^{KO}$ iBMDMs following STING activation (**Fig.5C**). In the absence of activation cFos is highly unstable, however phosphorylation enhances both its protein stability and nuclear localization (Murphy *et al*, 2002 – PMID: 12134156). Consistent with defective cFos translocation, we also observed reduced STING-mediated cFos phosphorylation in cell lysates from $IRF3^{KO}$ iBMDMs compared to their WT counterparts (**Fig.5D**). Similarly, phosphorylation of extracellular signal-regulated kinase (ERK)1/2, which is known to mediate cFos phosphorylation (Gilley *et al*, 2009 – PMID: 19249353), was also greatly reduced in the absence of IRF3 following STING activation, while NF-κB p65 phosphorylation remained intact (**Fig.5D** and **Fig.EV5**). Together these data demonstrate that the reduction in STING pro-inflammatory cytokines observed from $IRF3^{KO}$ iBMDMs may be driven by a defect in the ERK1/2–cFos signalling module.

6. *Fig. 3: Signaling data for TBK1 KO is missing. It would be important to know whether TBK1 KO cells phenocopy the TNFα/IL-6 defect seen in IRF3/7 KO cells.*

We have now examined TNF in this setting finding that the lack of TBK1 also led to reduced TNF secretion (**Fig.3C**), albeit not as pronounced as the decrease observed in IFNβ production (**Fig.3B**). This is most likely due to the compensatory role of IKKε in STING-mediated non-IFN responses (Balka *et al*, 2020 – PMID: 32268090; and Venkatraman *et al*, 2024 – PMID: 39262777).

7. *Fig. 4B: The authors should include co-immunoprecipitation data of IRF3 with various STING mutants (e.g., S365A, L373A, S354A, S357A) to validate the proposed recruitment model. This would provide valuable cross-validation and mechanistic depth.*

We agree with the reviewer that interaction data would be valuable here. To examine the interactions IRF3 and STING in this model we took several approaches. Firstly, we utilised

endogenous STING IP and HA IP approaches that we have successfully utilised in the past to examine STING interactions with TBK1 and IKKε in murine macrophages (Venkatraman *et al*, 2024 – PMID: 39262777 and Balka *et al.*, 2023 – PMID: 37139896). However, in both cases while we were able to observe interactions between STING and TBK1/IKKε, we could not detect any IRF3. We next attempted endogenous IRF3 IPs (Rabbit monoclonal anti-IRF3 (D83B9) antibody, Cell Signaling Technology, Cat# 4302), however we were unable to detect STING nor TBK1/IKKε. Finally, as we were able to capture IRF3–STING complex interactions via the exogenous expression of GFP-IRF3 in combination with GFP IPs (**Fig.3D**), we expressed GFP-IRF3 in *Sting*$^{-/-}$ iBMDMs, as well as those expressing WT, S365A or L373A versions of STING. While IRF3, STING, TBK1 and IKKε interacted in cells expressing WT STING, no IRF3 interaction was observed in those expressing STING S365A (**Fig.4D**). As expected, we did not observe any interaction of IRF3 in *Sting*$^{-/-}$ iBMDMs expressing STING L373A (**Fig.4D**), nor STING phosphorylation at S365 (**Fig.EV4E**).

8. *Discussion: The rationale for concluding a scaffolding function of IRF3 is unclear. This hypothesis needs to be better justified based on the presented data.*

We apologise that this was unclear in the original version of the manuscript and concede this notion may have been unduly overstated. We have therefore modified the discussion as follows: "*These observations raise the possibility that IRF3 may also function as a scaffold to facilitate STING-driven pro-inflammatory cytokine production by promoting ERK1/2-cFos activity, independently of canonical NF-κB. However, this model requires further investigation.*"

9. *Typos:*

- Line 139: remove "any" or "a"

- Line 182: remove "via"

- Line 192: include "S357A"

- Line 283: replace "indispensible" with "indispensable"

These errors have been amended in the revised version, thank you.

Overall assessment:

This work addresses an interesting mechanistic question about STING-IRF3-NF-κB crosstalk. However, without rigorous exclusion of IFN-I-dependent mechanisms, the main conclusion is premature. Addressing the above points-particularly the IFNAR-blockade experiments and

inclusion of IFN-I-independent cytokine readouts-will be essential for the manuscript to meet the conceptual and experimental standards of EMBO reports.

Referee #3:

In this manuscript, the authors provided the clear evidence of non-canonical IRF3 function mediating STING-dependent pro-inflammatory cytokine production in macrophages. Although several papers have already addressed the role of IRF3 in the production of pro-inflammatory cytokines, this study is distinct in showing that IRF3 can be associated with STING in the absence of TBK1 (Figure 3). This finding is important to open the new way to understand the non-canonical role of IRF3. The experiments/results are overall robust and compelling. The manuscript is well-written and cites proper publications.

Comments:

***1.** Since IRF3 appears dispensable for pro-inflammatory cytokine production in dendritic cells, the title should be revised in that way. I propose adding "in macrophage" to the title.*

We thank the reviewer for this suggestion and have amended the title.

***2.** STING dimer can be multimerized in the trans-Golgi network (PMID: 38212328), which may be linked to non-canonical IRF3 function as scaffold. This paper can be included in "Discussion".*

We agree that the importance of palmitoylation and cholesterol in STING clustering is worth mentioning and have added this to the discussion.

***3.** line 150: "maker" should read "marker".*

This has been amended in the revised version, thank you.

1st Revision - Editorial Decision **15th Apr 2026**

Manuscript number: EMBOR-2025-62361V3
Title: Noncanonical IRF3 function mediates STING-dependent pro-inflammatory cytokine production in macrophages
Author(s): Katherine Balka, Olivia Lamb, Rajan Venkatraman, Zoe Magill, Le Tri Hoang, Ryker Dumbrill, Kate McArthur, Nicole de Weerd, Paul Hertzog, Peter Crack, Linda Wakim, Kim Good-Jacobson, Mireille LAHOUD, Meredith O'Keeffe, and Dominic De Nardo

Dear Dom,

I had already sent you the positive referee reports (copied again below) and now follow up with a formal 'accept in principle' decision letter, as promised. There is actually not much to ask for from the editorial side - your paper was almost perfectly formatted.

These are the points that require changes:

- Regarding the Author Contributions, we now use CRediT to specify the contributions of each author in the journal submission system. CRediT replaces the author contribution section, which therefore needs to be removed from the manuscript text. You can use the free text box in our system if you wish to provide more detailed descriptions. See also guide to authors https://www.embopress.org/page/journal/14693178/authorguide#authorshipguidelines.

- The information on funding provided in the manuscript and the online manuscript tracking system must be congruent. Currently, the following funders are listed in the manuscript but are missing from the system as separate entries: Australian and New Zealand Society of Immunology (ASI) Breakthrough Immunology Award, Monash Silver Jubilee Postgraduate Research scholarship and a Monash Graduate Excellence Scholarship, Australian Government Research Training Program Scholarships; The Comments box should not be used please.

- Please provide a callout for Fig. 5AB.

- The nomenclature of EV figure legends and individual Figure files is not correct: it needs to be Figure EV1, etc. instead of EV Figure 1, etc.

- Please describe your findings in the abstract in present tense.

- Finally, EMBO Reports papers are accompanied online by
A) a short (1-2 sentences) summary of the findings and their significance,
B) 2-3 bullet points highlighting key results and
C) a schematic summary figure that provides a sketch of the major findings (not a data image).
Please provide the summary figure as a separate file in PNG or JPG format at a size of 550x300 pixels (width x height). Please note that the size is rather small and that text needs to be readable at the final size. Please send us this information along with the revised manuscript.

Once you have made these minor revisions, please use the following link to submit your corrected manuscript:

Link Not Available

If all remaining corrections have been attended to, you will then receive an official decision letter from the journal accepting your manuscript for publication in the next available issue of EMBO reports. This letter will also include details of the further steps you need to take for the prompt inclusion of your manuscript in our next available issue.

Thank you for your contribution to EMBO reports.

Best regards,

Martina

Martina Rembold, PhD
Senior Editor
EMBO reports

================

Referee #1:

The authors have addressed my concerns. No further comments.

Referee #2:

The authors addressed all comments thoroughly and added new data that provide further mechanistic insight into the IFN-independent role of IRF3, including the involvement of ERK1/2-cFos signaling.
One minor point to correct during proofs: in Figure 3B-C, the annotation appears incomplete, as one of the colors is missing.

All minor editorial requests have been addressed by the authors.

Dr. Dominic De Nardo
Monash University
Biochemistry and Molecular Biology
Room 256, Level 2, Building 75
15 Innovation Walk
Clayton, VIC 3800
Australia

Dear Dom,

Thank you for implementing the final minor revisions. I am very pleased to accept your manuscript for publication in the next available issue of EMBO reports. Thank you for your contribution to our journal.

Your manuscript will be processed for publication by EMBO Press. It will be copy edited and you will receive page proofs prior to publication. Please note that you will be contacted by Springer Nature Author Services to complete licensing and payment information.

You may qualify for financial assistance for your publication charges - either via a Springer Nature fully open access agreement or an EMBO initiative. Check your eligibility: https://link.springer.com/journal/44319/how-to-publish-with-us

Should you be planning a Press Release on your article, please get in contact with embo_production@springernature.com as early as possible in order to coordinate publication and release dates.

If you have any questions, please do not hesitate to contact the Editorial Office. Thank you for your contribution to EMBO Reports.

Kind regards,

Martina

Martina Rembold, PhD
Senior Editor
EMBO reports

>>> Please note that it is EMBO Reports policy for the transcript of the editorial process (containing referee reports and your response letter) to be published as an online supplement to each paper. If you do NOT want this, you will need to inform the Editorial Office via email immediately. More information is available here: https://link.springer.com/partners/embo-press/editorial-policies#Peer%20review

EMBO Press Author Checklist

	USEFUL LINKS FOR COMPLETING THIS FORM
Corresponding Author Name: Dominic De Nardo	[The EMBO Journal - Author Guidelines](#)
Journal Submitted to: EMBO Reports	[EMBO Reports - Author Guidelines](#)
Manuscript Number: EMBOR-2025-62361V3	[Molecular Systems Biology - Author Guidelines](#)
	[EMBO Molecular Medicine - Author Guidelines](#)

Reporting Checklist for Life Science Articles (updated January
This checklist is adapted from Materials Design Analysis Reporting (MDAR) Checklist for Authors. MDAR establishes a minimum set of requirements in transparent reporting in the life sciences (see Statement of Task: 10.31222/osf.io/9sm4x). Please follow the journal's guidelines in preparing your
Please note that a copy of this checklist will be published alongside your article.

Abridged guidelines for figures
1. Data
The data shown in figures should satisfy the following conditions:
- ➞ the data were obtained and processed according to the field's best practice and are presented to reflect the results of the experiments in an accurate and unbiased manner.
- ➞ ideally, figure panels should include only measurements that are directly comparable to each other and obtained with the same assay.
- ➞ plots include clearly labeled error bars for independent experiments and sample sizes. Unless justified, error bars should not be shown for technical
- ➞ if n<5, the individual data points from each experiment should be plotted. Any statistical test employed should be justified.
- ➞ Source Data should be included to report the data underlying figures according to the guidelines set out in the authorship guidelines on Data

2. Captions
Each figure caption should contain the following information, for each panel where they are relevant:
- ➞ a specification of the experimental system investigated (eg cell line, species name).
- ➞ the assay(s) and method(s) used to carry out the reported observations and measurements.
- ➞ an explicit mention of the biological and chemical entity(ies) that are being measured.
- ➞ an explicit mention of the biological and chemical entity(ies) that are altered/varied/perturbed in a controlled manner.
- ➞ the exact sample size (n) for each experimental group/condition, given as a number, not a range;
- ➞ a description of the sample collection allowing the reader to understand whether the samples represent technical or biological replicates (including how many animals, litters, cultures, etc.).
- ➞ a statement of how many times the experiment shown was independently replicated in the laboratory.
- ➞ definitions of statistical methods and measures:
  - common tests, such as t-test (please specify whether paired vs. unpaired), simple χ2 tests, Wilcoxon and Mann-Whitney tests, can be unambiguously identified by name only, but more complex techniques should be described in the methods section;
  - are tests one-sided or two-sided?
  - are there adjustments for multiple comparisons?
  - exact statistical test results, e.g., P values = x but not P values < x;
  - definition of 'center values' as median or average;
  - definition of error bars as s.d. or s.e.m.

Please complete ALL of the questions below.
Select "Not Applicable" only when the requested information is not relevant for your study.

Materials

Newly Created Materials	Information included in the manuscript?	In which section is the information available? (Reagents and Tools Table, Materials and Methods, Figures, Data Availability Section)
New materials and reagents need to be available; do any restrictions apply?	Yes	Reagents & Tools Table, Materials & Methods

Antibodies	Information included in the manuscript?	In which section is the information available? (Reagents and Tools Table, Materials and Methods, Figures, Data Availability Section)
For **antibodies** provide the following information:<br>- Commercial antibodies: RRID (if possible) or supplier name, catalogue number and or/clone number<br>- Non-commercial: RRID or citation	Yes	Reagents & Tools Table, Materials & Methods

DNA and RNA sequences	Information included in the manuscript?	In which section is the information available? (Reagents and Tools Table, Materials and Methods, Figures, Data Availability Section)
**Short novel DNA or RNA including primers, probes**: provide the sequences.	Yes	Reagents & Tools Table

Cell materials	Information included in the manuscript?	In which section is the information available? (Reagents and Tools Table, Materials and Methods, Figures, Data Availability Section)
**Cell lines:** Provide species information, strain. Provide accession number in repository **OR** supplier name, catalog number, clone number, and/**OR** RRID.	Yes	Reagents & Tools Table
**Primary cultures:** Provide species, strain, sex of origin, genetic modification status.	Yes	Materials & Methods
Report if the cell lines were recently **authenticated** (e.g., by STR profiling) and tested for mycoplasma contamination.	Yes	Materials & Methods

Experimental animals	Information included in the manuscript?	In which section is the information available? (Reagents and Tools Table, Materials and Methods, Figures, Data Availability Section)
**Laboratory animals or Model organisms:** Provide species, strain, sex, age, genetic modification status. Provide accession number in repository **OR** supplier name, catalog number, clone number, **OR** RRID.	Yes	Reagents & Tools Table, Materials & Methods
**Animal observed in or captured from the field:** Provide species, sex, and age where possible.	Not Applicable	
Please detail **housing and husbandry conditions**.	Not Applicable	

Plants and microbes	Information included in the manuscript?	In which section is the information available? (Reagents and Tools Table, Materials and Methods, Figures, Data Availability Section)
**Plants:** provide species and strain, ecotype and cultivar where relevant, unique accession number if available, and source (including location for collected wild specimens).	Not Applicable	
**Microbes:** provide species and strain, unique accession number if available, and source.	Not Applicable	

Human research participants	Information included in the manuscript?	In which section is the information available? (Reagents and Tools Table, Materials and Methods, Figures, Data Availability Section)
If collected and within the bounds of privacy constraints report on age, sex and gender or ethnicity for all study participants.	Not Applicable	

Core facilities	Information included in the manuscript?	In which section is the information available? (Reagents and Tools Table, Materials and Methods, Figures, Data Availability Section)
If your work benefited from core facilities, was their service mentioned in the acknowledgments section?	Yes	Acknowledgments

Design

Study protocol	Information included in the manuscript?	In which section is the information available? (Reagents and Tools Table, Materials and Methods, Figures, Data Availability Section)
If study protocol has been **pre-registered, provide DOI in the manuscript**. For clinical trials, provide the trial registration number **OR** cite DOI.	Not Applicable	
Report the **clinical trial registration number** (at ClinicalTrials.gov or equivalent), where applicable.	Not Applicable	

Laboratory protocol	Information included in the manuscript?	In which section is the information available? (Reagents and Tools Table, Materials and Methods, Figures, Data Availability Section)
Provide DOI OR other citation details if **external detailed step-by-step protocols** are available.	Not Applicable	

Experimental study design and statistics	Information included in the manuscript?	In which section is the information available? (Reagents and Tools Table, Materials and Methods, Figures, Data Availability Section)
Include a statement about **sample size** estimate even if no statistical methods were used.	Not Applicable	
Were any steps taken to minimize the effects of subjective bias when allocating animals/samples to treatment (e.g. **randomization procedure**)? If yes, have they been described?	Not Applicable	
Include a statement about **blinding** even if no blinding was done.	Not Applicable	
Describe **inclusion/exclusion criteria** if samples or animals were excluded from the analysis. Were the criteria pre-established?<br><br>If sample or data points were omitted from analysis, report if this was due to attrition or intentional exclusion and provide justification.	Not Applicable	
For every figure, are **statistical tests** justified as appropriate? Do the data meet the assumptions of the tests (e.g., normal distribution)? Describe any methods used to assess it. Is there an estimate of variation within each group of data? Is the variance similar between the groups that are being statistically compared?	Yes	Figure Legends

Sample definition and in-laboratory replication	Information included in the manuscript?	In which section is the information available? (Reagents and Tools Table, Materials and Methods, Figures, Data Availability Section)
In the figure legends: state number of times the experiment was **replicated** in laboratory.	Yes	Figure Legends
In the figure legends: define whether data describe **technical or biological replicates**.	Yes	Figure Legends

Ethics

Ethics	Information included in the manuscript?	In which section is the information available? (Reagents and Tools Table, Materials and Methods, Figures, Data Availability Section)
Studies involving **human participants**: State details of **authority granting ethics approval** (IRB or equivalent committee(s), provide reference number for approval.	Not Applicable	
Studies involving **human participants**: Include a statement confirming that **informed consent** was obtained from all subjects and that the experiments conformed to the principles set out in the WMA Declaration of Helsinki and the Department of Health and Human Services Belmont Report.	Not Applicable	
Studies involving **human participants:** For publication of **patient photos**, include a statement confirming that consent to publish was obtained.	Not Applicable	
Studies involving experimental **animals**: State details of **authority granting ethics approval** (IRB or equivalent committee(s), provide reference number for approval. Include a statement of compliance with ethical regulations.	Yes	Materials & Methods
Studies involving **specimen and field samples:** State if relevant **permits** obtained, provide details of authority approving study; if none were required, explain why.	Not Applicable	

Dual Use Research of Concern (DURC)	Information included in the manuscript?	In which section is the information available? (Reagents and Tools Table, Materials and Methods, Figures, Data Availability Section)
Could your study fall under dual use research restrictions? Please check biosecurity documents and list of **select agents and toxins** (CDC): https://www.selectagents.gov/sat/list.htm	Not Applicable	
If you used a select agent, is the security level of the lab appropriate and reported in the manuscript?	Not Applicable	
If a study is subject to dual use research of concern regulations, is the name of the **authority granting approval and reference number** for the regulatory approval provided in the manuscript?	Not Applicable	

Reporting

The MDAR framework recommends adoption of discipline-specific guidelines, established and endorsed through community initiatives. Journals have their own policy about requiring specific guidelines and recommendations to complement MDAR.

Adherence to community standards	Information included in the manuscript?	In which section is the information available? (Reagents and Tools Table, Materials and Methods, Figures, Data Availability Section)
State if relevant guidelines or checklists (e.g., **ICMJE, MIBBI, ARRIVE, PRISMA**) have been followed or provided.	Not Applicable	
For **tumor marker prognostic studies,** we recommend that you follow the **REMARK** reporting guidelines (see link list at top right). See author guidelines, under 'Reporting Guidelines'. Please confirm you have followed these guidelines.	Not Applicable	
For **phase II and III randomized controlled trials**, please refer to the **CONSORT** flow diagram (see link list at top right) and submit the CONSORT checklist (see link list at top right) with your submission. See author guidelines, under 'Reporting Guidelines'. Please confirm you have submitted this list.	Not Applicable	

Data Availability

Data availability	Information included in the manuscript?	In which section is the information available? (Reagents and Tools Table, Materials and Methods, Figures, Data Availability Section)
Have **primary datasets** been deposited according to the journal's guidelines (see 'Data Deposition' section) and the respective accession numbers provided in the Data Availability Section?	Not Applicable	
Were **human clinical and genomic datasets** deposited in a public access-controlled repository in accordance to ethical obligations to the patients and to the applicable consent agreement?	Not Applicable	
Are **computational models** that are central and integral to a study available without restrictions in a machine-readable form? Were the relevant accession numbers or links provided?	Not Applicable	
If publicly available data were reused, provide the respective **data citations in the reference list**.	Not Applicable	
